# iMethyl-PseAAC: Identification of Protein Methylation Sites via a Pseudo Amino Acid Composition Approach

**DOI:** 10.1155/2014/947416

**Published:** 2014-05-22

**Authors:** Wang-Ren Qiu, Xuan Xiao, Wei-Zhong Lin, Kuo-Chen Chou

**Affiliations:** ^1^Computer Department, Jingdezhen Ceramic Institute, Jingdezhen 333046, China; ^2^Information School, ZheJiang Textile & Fashion College, Ningbo 315211, China; ^3^Gordon Life Science Institute, Boston, MA 02478, USA; ^4^Center of Excellence in Genomic Medicine Research (CEGMR), King Abdulaziz University, Jeddah 21589, Saudi Arabia

## Abstract

Before becoming the native proteins during the biosynthesis, their polypeptide chains created by ribosome's translating mRNA will undergo a series of “product-forming” steps, such as cutting, folding, and posttranslational modification (PTM). Knowledge of PTMs in proteins is crucial for dynamic proteome analysis of various human diseases and epigenetic inheritance. One of the most important PTMs is the Arg- or Lys-methylation that occurs on arginine or lysine, respectively. Given a protein, which site of its Arg (or Lys) can be methylated, and which site cannot? This is the first important problem for understanding the methylation mechanism and drug development in depth. With the avalanche of protein sequences generated in the postgenomic age, its urgency has become self-evident. To address this problem, we proposed a new predictor, called iMethyl-PseAAC. In the prediction system, a peptide sample was formulated by a 346-dimensional vector, formed by incorporating its physicochemical, sequence evolution, biochemical, and structural disorder information into the general form of pseudo amino acid composition. It was observed by the rigorous jackknife test and independent dataset test that iMethyl-PseAAC was superior to any of the existing predictors in this area.

## 1. Introduction

**Figure 1:**
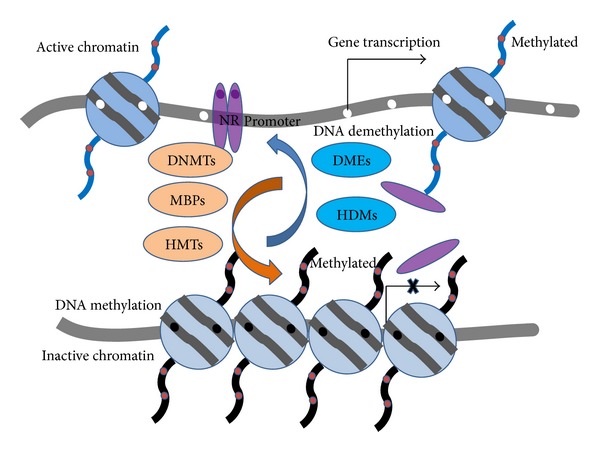
Schematic drawing to show the involvement of the Arg-methylation and Lys-methylation in gene regulation (adapted from [13] with permission).

Posttranslational modifications (PTMs) of proteins are crucial for understanding the dynamic proteome and various signaling pathways or networks in cells. As one of the most important PTMs, protein methylation typically occurs on arginine (Arg) or lysine (Lys) residues in the protein sequence [1]. In fact, there are growing evidences indicating that protein Arg-methylation is capable of providing important regulatory mechanisms for gene expression in a wide variety of biological contexts [2] and that Lys-methylation is correlated with either gene activation or repression depending on the site and degree of methylation [3]. Owing to their important roles in gene regulation (Figure 1), the Arg-methylation and Lys-methylation as well as their regulatory enzymes are implicated in a variety of human disease states, such as cancer [4], coronary heart disease [5], multiple sclerosis [6], rheumatoid arthritis [7], and neurodegenerative disorders [8]. Furthermore, epigenetic inheritance due to methylation can occur through either DNA methylation or protein methylation. Many researches on humans have shown that repeated high-level activation of the body's stress system (particularly in early childhood) could alter methylation processes, leading to changes in the chemistry of the individual's DNA. The chemical changes could disable genes and prevent the brain from properly regulating its response to stress. Researchers and clinicians have drawn a link between this neurochemical dysregulation and the development of chronic health problems such as depression [9], obesity [10], diabetes [11], and hypertension [12]. Therefore, it would certainly provide very useful information or clues for drug discovery to study and analyze the mechanisms that govern these basic epigenetic phenomena.

Although the full extent of regulatory roles of protein methylation is still under elusive investigation, many efforts have been made to determine the methylation sites with experimental approaches, such as mutagenesis of potential methylated residues, methylation-specific antibodies [14], and mass spectrometry [15, 16]. The results obtained from these experimental methods have not only provided reliable methylation sites but also indicated that the Arg-methylation and Lys-methylation were closely correlated with the local downstream and upstream residues from the central Arg and Lys, respectively. Unfortunately, even if the number of local residues was limited at *ξ* = 5, 6, or  7 for both downstream and upstream, it is by no means easy to determine all the methylation sites. This is because the number of possible peptide sequence *N* thus formed from 20 amino acids runs into

(1) N=202ξ=102ξlog⁡(20)={1.0240×1013,when  ξ=54.0960×1015,when  ξ=61.6384×1018,when  ξ=7,

which is an astronomical figure for any of the above three cases! It would be exhausting to purely utilize the experimental approaches to determine the large-scale methylation sites. With the avalanche of protein sequences generated in the postgenomic age, it is highly desired to develop automated methods for rapidly and reliably identifying the methylation sites in proteins.

Actually, considerable efforts have been made in this regard. For instance, Daily et al. [17] developed a method for predicting Arg- and Lys-methylation sites using Support Vector Machine (SVM) based on the hypothesis that PTMs preferentially occurred in intrinsically disordered regions [18]. Chen et al. [19] built a web server called MeMo for identifying methylation sites by using the orthogonal binary coding scheme to formulate the protein sequence fragments and SVM to operate the prediction. Using Bi-profile Bayes feature extraction approach, Shao et al. [20] developed a predictor called BPB-PPMS to identify protein methylation sites. Meanwhile, Shien et al. [21] proposed a methylation site prediction method called MASA, in which both sequence information and structural characteristics, such as accessible surface area (ASA) and secondary structure of residues surrounding the methylation sites, were taken into account. Two years later, another method in this area was presented by Hu et al. [22] using the feature selection approach and nearest neighbor algorithm. Recently, Shi et al. [23] developed a method called PMeS to improve the prediction of protein methylation sites based on an enhanced feature encoding scheme and SVM. Although each of the aforementioned methods has its own merit and did play a role in stimulating the development of this area, they all need improvement from one or more of the following aspects: (i) the benchmark dataset used by the previous investigators needs to be updated by incorporating some new and experiment-confirmed data, or improved by removing redundancy and duplicate sequences; (ii) further enhancing the prediction quality by introducing the state-of-the-art machine learning techniques; (iii) making the formulation of all the statistical samples purely based on the sequence information alone because some of the existing methods also needed the structural information that was not always available and hence would unavoidably suffer from some limitation; and (iv) establishing user-friendly and public-accessible web servers because most of the existing methods did not have any web server whatsoever or the web server did not work.

The present study was initiated with an attempt to develop a new predictor for identifying protein methylation sites by focusing on the abovementioned four aspects.

According to a recent comprehensive review [24] and demonstrated by a series of recent publications (see, e.g., [25–28]), to establish a really useful statistical predictor for a protein or peptide system, we need to consider the following procedures: (i) construct or select a valid benchmark dataset to train and test the predictor; (ii) formulate the protein or peptide samples with an effective mathematical expression that can truly reflect their intrinsic correlation with the target to be predicted; (iii) introduce or develop a powerful algorithm (or engine) to operate the prediction; (iv) properly perform cross-validation tests to objectively evaluate the anticipated accuracy of the predictor; and (v) establish a user-friendly web server for the predictor that is accessible to the public. Below, let us describe how to deal with these steps one by one.

## 2. Materials and Methods

### 2.1. Benchmark Dataset

To develop a statistical predictor, it is fundamentally important to establish a reliable and stringent benchmark dataset to train and test the predictor. If the benchmark dataset contains some errors, the predictor trained by it must be unreliable and the accuracy tested by it would be completely meaningless. The benchmark dataset used by Hu et al. [22] contained many duplicate peptide sequences and self-conflicting data. As shown in Part I of the Online Supporting Information S1 available online at http://dx.doi.org/10.1155/2014/947416, of the 180 samples in their positive Arg-methylation learning dataset, 5 were duplicates; of the 2,171 negative learning dataset, 64 were duplicates; of the 10 samples in their positive Arg-methylation testing dataset, 3 were duplicates; of the 206 samples in the negative testing dataset, 46 were duplicates. Similarly, as shown in Part II of the supporting information, of the 262 samples in their positive Lys-methylation learning dataset, 3 were duplicates; of the 2,569 negative learning dataset, 506 were duplicates; of the 48 samples in their positive Lys-methylation testing dataset, 24 were duplicates; of the 243 samples in the negative testing dataset, 111 were duplicates. Also, in their benchmark dataset [22], there were many self-conflicting samples. As shown in Part I of the Online Supporting Information S2, of the 2,351 samples in their learning dataset for Arg-methylation, 8 occur in both positive and negative subsets. Similarly, as shown in Part II of the supporting information, of the 2,831 samples in their learning dataset for Lys-methylation, 60 occur in both positive and negative subsets. Of the 291 samples in their testing dataset for Lys-methylation, 5 occur in both positive and negative subsets. Therefore, the first important thing is to construct a new and reliable benchmark dataset by getting rid of all the duplicates or self-conflicting sequence data. The concrete procedures can be summarized as follows.

**Figure 2:**
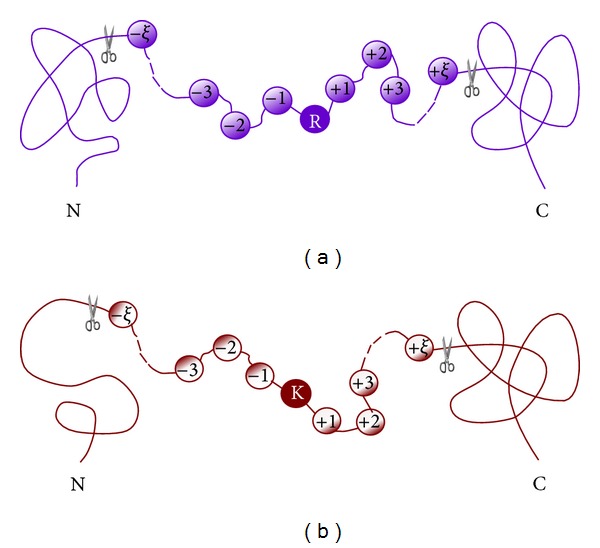
Schematic drawing to show the Chou's peptide formulation for studying (a) Arg-methylation and (b) Lys-methylation (adapted from [32, 33] with permission).

In this study the benchmark dataset was derived from the Swiss-Prot database (version 2013_06). Collected were those proteins that had clear experimental annotations about their Arg-methylation and Lys-methylation sites. For facilitating description later, let us adopt the Chou's peptide formulation that was used for studying HIV protease cleavage sites [29, 30], specificity of GalNAc-transferase [31], and signal peptide cleavage sites [32]. According to Chou's scheme, a peptide with Arg (namely R in its single-letter code) or Lys (namely K) located at its center (Figure 2) can be expressed as

(2) $$\begin{array}{c} P\left(R\right)={\text{R}}_{-\xi }^{}{\text{R}}_{-\left(\xi -1\right)}^{}\cdots {\text{R}}_{-2}^{}{\text{R}}_{-1}^{}R{\text{R}}_{+1}^{}{\text{R}}_{+2}^{}\cdots {\text{R}}_{+\left(\xi -1\right)}^{}{\text{R}}_{+\xi }^{}\\ P\left(K\right)={\text{R}}_{-\xi }^{}{\text{R}}_{-\left(\xi -1\right)}^{}\cdots {\text{R}}_{-2}^{}{\text{R}}_{-1}^{}K{\text{R}}_{+1}^{}{\text{R}}_{+2}^{}\cdots {\text{R}}_{+\left(\xi -1\right)}^{}{\text{R}}_{+\xi }^{}, \end{array}$$

where the subscript *ξ* is an integer (cf. (1)), R_−*ξ*_ represents the *ξ*th downstream amino acid residue from the center, R_*ξ*_ the *ξ*th upstream amino acid residue, and so forth (Figures 2(a) and 2(b)). Peptides **P**(*R*) and **P**(*K*) with the profile of (2) can be further classified into the following categories:

(3) P(R)∈{Arg-methylation  peptide,if  its  center  is  a  methylation  sitenon-Arg-methylation  peptide,otherwise,P(R)∈{Lys-methylation  peptide,if  its  center  is  a  methylation  sitenon-Lys-methylation  peptide,otherwise,

where ∈ represents “a member of” in the set theory.

As pointed out in a comprehensive review [34], there is no need to separate a benchmark dataset into a training dataset and a testing dataset for validating a prediction method if it is tested by the jackknife or subsampling (K-fold) cross-validation because the outcome thus obtained is actually from a combination of many different independent dataset tests. Thus, the benchmark dataset for the current study can be formulated as

(4) $$\begin{array}{c} S_{\text{R}}=S_{\text{R}}^{+}\cup S_{\text{R}}^{-}\\ S_{\text{K}}=S_{\text{K}}^{+}\cup S_{\text{K}}^{-}, \end{array}$$

where *S*_R_ is the benchmark dataset for Arg-methylation, *S*_K_ is the benchmark dataset for Lys-methylation, ∪ is the symbol for “union” in the set theory, *S*_R_^+^ contains the samples for Arg-methylation peptides only, *S*_R_^−^  contains the samples for non-Arg-methylation peptides only (cf. (3)), and so forth.

After some preliminary trials and also considering the treatment by the previous investigators [17–20, 22, 23], we chose *ξ* = 5 (cf. (2)) to construct the samples for the benchmark datasets *S*_R_ and *S*_K_, respectively. The detailed procedure was as follows. If the upstream or downstream in a protein was less than 5, the lacking residues were filled with the same residue of its closest neighbor. The peptide samples thus obtained were subject to a screening procedure to winnow those that were identical to any other. Excluded from our benchmark dataset were also those that were self-conflict, namely, occurring in both methylation group and nonmethylation group.

Finally, we obtained 1,481 peptide samples for *S*_R_, of which 185 samples were of Arg-methylation belonging to the positive dataset *S*_R_^+^, and 1,296 samples of non-Arg-methylation belonging to the negative dataset *S*_R_^−^. The Arg-methylation sites and their corresponding (2*ξ* + 1) = 11 amino acids along the protein chain are given in the Online Supporting Information S3. Similarly, we also obtained 1,884 peptide samples for *S*_K_, of which 226 samples were of Lys-methylation belonging to the positive dataset *S*_K_^+^, and 1,518 samples of non-Lys-methylation belonging to the negative dataset *S*_K_^−^. The Lys-methylation sites and their corresponding (2*ξ* + 1) = 11 amino acids along the protein chain are given in the Online Supporting Information S4.

### 2.2. Sample Formulation

One of the most important but also most difficult problems in computational biology is how to formulate a biological sequence with a discrete model or a vector, yet still keep considerable sequence order information. This is because all the existing operation engines, such as “Correlation Angle” method [35–37], “Optimization Approach” [38], “Component Coupled” algorithm [39, 40], “Covariance Discriminant” or CD algorithm [41–44], “Neural Network” algorithm [45, 46], Support Vector Machine or SVM algorithm [27, 47], “Random Forest” algorithm [48], “Conditional Random Field” algorithm [44], “Nearest Neighbor” algorithm [49], “K-Nearest Neighbor” or KNN algorithm [50], “Optimized Evidence-Theoretic K-Nearest Neighbor” or OET-KNN algorithm [51], and “Fuzzy K-Nearest Neighbor” algorithm [26, 52], can only handle vector but not sequence samples. However, a vector defined in a discrete model may completely lose all the sequence-order information [53]. Therefore, in developing a statistical method for predicting the attribute of a peptide in protein, an important task is to formulate the peptide with a vector that can truly reflect its key feature by incorporating some of its sequence information.

To realize this, various feature vectors (see, e.g., [26, 44, 54–64]) were proposed to express proteins or peptides by extracting their different features into the pseudo amino acid composition [53, 65] or Chou's PseAAC [66–68] or general form of PseAAC [24, 69].

According to [24], the general form of PseAAC for a protein or peptide **P** can be formulated by

(5) $$\begin{array}{c} P={\left[\begin{array}{cccccc} \psi_{1} & \psi_{2} & \cdots & \psi_{u} & \cdots & \psi_{\Omega } \end{array}\right]}^{T}, \end{array}$$

where **T** is the transpose operator, while *Ω* an integer to reflect the vector's dimension. The value of *Ω* as well as the components *ψ*_*u*_  (*u* = 1,2,…, *Ω*) in (5) will depend on how to extract the desired information from the protein or peptide sequence. Below, let us describe how to extract the useful information from the benchmark datasets *S*_R_ and *S*_K_ to define the peptide samples via (5). Actually, we are to approach this problem from the following four aspects: (i) position specific scoring matrices (PSSM), (ii) grey-PSSM approach, (iii) amino acid factors (AAF), and (iv) disorder score (DS).

Biology is a natural science with historic dimension. All biological species have developed beginning from a very limited number of ancestral species. It is true for protein sequence as well [70]. Their evolution involves changes of single residues, insertions and deletions of several residues [71], gene doubling, and gene fusion. To incorporate this kind of evolution information into (5), let us consider the following.

According to [72], the sequence evolution information for a peptide with 11 amino acid residues can be expressed by a 11 × 20 matrix, as given by

(6) $$\begin{array}{c} P_{\text{PSSM}}^{0}=\left[\begin{array}{cccc} m_{1,1}^{0} & m_{1,2}^{0} & \cdots & m_{1,20}^{0}\\ m_{2,1}^{0} & m_{2,2}^{0} & \cdots & m_{2,20}^{0}\\ \vdots & \vdots & \ddots & \vdots \\ m_{11,1}^{0} & m_{11,2}^{0} & \cdots & m_{11,20}^{0} \end{array}\right], \end{array}$$

where  *m*_*i*,*j*_^0^ represents the original score of amino acid residue in the *i*th  (*i* = 1, 2,…, 11) sequential position of the peptide that is being changed to amino acid type *j*  (*j* = 1, 2,…, 20) during the evolution process. Here, the numerical codes 1, 2, …, 20 are used to denote the 20 native amino acid types according to the alphabetical order of their single character codes [73]. The 11 × 20 scores in (6) were generated by using PSI-BLAST [72] to search the UniProtKB/Swiss-Prot database (Release 2011_05) through three iterations with 0.001 as the *E*-value cutoff for multiple sequence alignment against the sequence of the peptide **P**. In order to make every element in (6) within the range of 0-1, a conversion was performed through the standard sigmoid function to make it become

(7) $$\begin{array}{c} P_{\text{PSSM}}=\left[\begin{array}{cccc} m_{1,1}^{} & m_{1,2}^{} & \cdots & m_{1,20}^{}\\ m_{2,1}^{} & m_{2,2}^{} & \cdots & m_{2,20}^{}\\ \vdots & \vdots & \ddots & \vdots \\ m_{11,1}^{} & m_{11,2}^{} & \cdots & m_{11,20}^{} \end{array}\right], \end{array}$$

where

(8) $$\begin{array}{c} m_{i,j}^{}=\frac{1}{1+e^{-m_{i,j}^{0}}}\;\left(1\leq i\leq 11,1\leq j\leq 20\right). \end{array}$$

Now let us use each of 11 × 20 = 220 elements in (7) to represent the 1st 220 components of (5),

(9) $$\begin{array}{c} \psi_{u}^{}=\{\begin{array}{cc} m_{1,1}^{} & \text{when}\;u=1\\ \vdots & \vdots \\ m_{11,1}^{} & \text{when}\;u=11\\ \vdots & \vdots \\ m_{1,20}^{} & \text{when}\;u=210\\ \vdots & \vdots \\ m_{11,20}^{} & \text{when}\;u=220. \end{array} \end{array}$$

Next, let us use the grey model approach to extract more useful information from (7) to define some additional components in (5). According to the grey system theory [74], if the information of a system investigated is fully known, it is called a “white system;” if completely unknown, a “black system;” if partially known, a “grey system”. The model developed on the basis of such a theory is called “grey model,” which is a kind of nonlinear and dynamic model formulated by a differential equation. The grey model is particularly useful for solving complicated problems that are lack of sufficient information, or need to process uncertain information and to reduce random effects of acquired data. Following the same approach as done by Lin et al. [25], besides the 220 components as defined in the above equation, we can add the following 3 × 20 = 60 additional components for (5):

(10) $$\begin{array}{c} \psi_{u}^{}=\{\begin{array}{cc} a_{1}^{1} & \text{when}\;u=221\\ a_{2}^{1} & \text{when}\;u=222\\ b_{}^{1} & \text{when}\;u=223\\ \vdots & \vdots \\ a_{1}^{20} & \text{when}\;u=278\\ a_{2}^{20} & \text{when}\;u=279\\ b_{}^{20} & \text{when}\;u=280, \end{array} \end{array}$$

where

(11) $$\begin{array}{c} \left[\begin{array}{c} a_{1}^{j}\\ a_{2}^{j}\\ b^{j} \end{array}\right]={\left(B_{j}^{T}B_{j}^{}\right)}^{-1}B_{j}^{T}U_{j}\;\left(j=1,2,\ldots ,20\right). \end{array}$$

In the above equation

(12) $$\begin{array}{c} B_{j}^{}=\left[\begin{array}{ccc} -m_{2,j}^{} & -m_{1,j}^{}-0.5m_{2,j}^{} & 1\\ -m_{3,j}^{} & -\overset{2}{\underset{i=1}{\sum }}m_{i,j}^{}-0.5m_{3,j}^{} & 1\\ \vdots & \vdots & \vdots \\ -m_{k,j}^{} & -\overset{k-1}{\underset{i=1}{\sum }}m_{i,j}^{}-0.5m_{k,j}^{} & 1\\ \vdots & \vdots & \vdots \\ -m_{11,j}^{} & -\overset{11-1}{\underset{i=1}{\sum }}m_{i,j}^{}-0.5m_{11,j}^{} & 1 \end{array}\right],\\ U_{j}^{}=\left[\begin{array}{c} m_{2,j}^{}-m_{1,j}^{}\\ m_{3,j}^{}-m_{2,j}^{}\\ \vdots \\ \begin{array}{c} m_{k,j}^{}-m_{k-1,j}^{}\\ \begin{array}{c} \vdots \\ m_{11,j}^{}-m_{10,j}^{} \end{array} \end{array} \end{array}\right]. \end{array}$$

The structure and function of proteins are largely dependent on the composition of various physicochemical properties of the 20 amino acids. These properties were described with the following five factors by Atchley et al. [75, 76]: (i) polarity (AAF-1), (ii) secondary structure (AAF-2), (iii) molecular volume (AAF-3), (iv) codon diversity (AAF-4), and (v) electrostatic charge (AAF-5). They were used to predict posttranslational modification sites [22, 77, 78]. Thus, using the AAIndex data [79, 80], we can add 5 × 11 = 55 components for (5) as formulated below

(13) $$\begin{array}{c} \psi_{u}^{}=\{\begin{array}{cc} r_{1}^{1} & \text{when}\;u=281\\ \vdots & \vdots \\ r_{5}^{1} & \text{when}\;u=285\\ \vdots & \vdots \\ r_{1}^{11} & \text{when}\;u=331\\ \vdots & \vdots \\ r_{5}^{11} & \text{when}\;u=335, \end{array} \end{array}$$

where *r*_*k*_^*l*^  (*k* = 1,2,…, 5; *l* = 1,2,…, 11) is the *k*th AAindex for the *l*th amino acid residue of the peptide concerned as given in Table 1 [76].

The functional importance of the disordered regions in proteins has been increasingly recognized [81, 82] and used to predict protein structures and functions [81, 83, 84]. According to Sickmeier et al. [85], they also play various roles in signaling and regulation by multiple binding of proteins and high-specificity low affinity interactions. To incorporate this kind of information into the PaeAAC of (5), the following 11 components were defined:

(14) $$\begin{array}{c} \psi_{u}^{}=\{\begin{array}{cc} {@}_{}^{1} & \text{when}\;u=336\\ {@}_{}^{2} & \text{when}\;u=337\\ \vdots & \vdots \\ {@}_{}^{11} & \text{when}\;u=346, \end{array} \end{array}$$

where @^*l*^ is the disorder score calculated by VSL2 [86] for the *l*th  (*l* = 1,2,…, 11) amino residue on the peptide sample.

Finally, we obtained the PseAAC with *Ω* = 346 components (cf. (5)**)**, of which 220 were defined by (9), 60 by (10), 55 by (13), and 11 by (14). And such 346-D feature vector was used to represent the peptide samples for further study.

### 2.3. Operation Engine

In this study, we used the SVM (Support Vector Machine) [87, 88] as the operation engine for conducting predictions. SVM is a powerful and popular method for pattern recognition that has been successfully used in the realm of bioinformatics (see, e.g., [64, 89–91]. The basic idea of SVM is to transform the data into a high dimensional feature space and then determine the optimal separating hyperplane using a kernel function. To handle a multiclass problem, “one-versus-one (OVO)” and “one-versus-rest (OVR)” are generally applied to extend the traditional SVM. For a brief formulation of SVM and how it works, see the papers [89, 92]. For more details about SVM, see a monograph [93].

The SVM software used in this paper was downloaded from the LIBSVM package [94], which provided a simple interface. Due to its advantages, the users can easily perform classification prediction by properly selecting the built-in parameters *c* and *γ*. In order to maximize the performance of the SVM algorithm, the two parameters in the RBF kernel were preliminarily optimized through a grid search strategy, as briefed as follows. As indicated in (9), (10), (13), and (14), each peptide sample in the current study was a 346-D vector containing *Ω* = 220 + 60 + 55 + 11 = 346 components. These 346 components were used as the input for each of the peptide samples investigated. The class values were set to 1 for methylation sites and −1 for nonmethylation sites. The threshold used to identify the positive (methylation) or negative (nonmethylation) peptide was set to 0 by default. For this kind of two-group classification, SVM would separate the classes with a surface that maximizes the margin between them. Because the ratio between the numbers of samples in the two groups was about one to seven (the samples in *S*_R_^+^ were 185, and the samples in *S*_R_^−^ were 1296, while the samples in *S*_K_^+^ were 226, and the samples in *S*_K_^−^ were 1518), the negative datasets were randomly divided into seven subsets for *S*_R_^−^ and *S*_K_^−^, respectively. During training process, the jackknife operations were conducted on such 14 datasets to optimize the SVM parameters using the search function SVMcgForClass, which was downloaded from http://www.matlabsky.com/.

The predictor obtained via the aforementioned procedures is called iMethyl-PseAAC.

How to properly and quantitatively measure the quality of a new predictor [95] and how to make it user-friendly for the public are the two key issues that have important impacts on its application value [96]. Below, let us address these two problems.

### 2.4. A Set of Metrics for Examining Prediction Quality

In literature the following four metrics are often used for examining the performance quality of a predictor

(15) $$\begin{array}{c} \text{Sn}=\frac{\text{TP}}{\text{TP}+\text{FN}}\\ \text{Sp}=\frac{\text{TN}}{\text{TN}+\text{FP}}\\ \text{Acc}=\frac{\text{TP}+\text{TN}}{\text{TP}+\text{TN}+\text{FP}+\text{FN}}\\ \text{MCC}=\frac{\left(\text{TP}\times \text{TN}\right)-\left(\text{FP}\times \text{FN}\right)}{\sqrt{\left(\text{TP}+\text{FP}\right)\left(\text{TP}+\text{FN}\right)\left(\text{TN}+\text{FP}\right)\left(\text{TN}+\text{FN}\right)}}, \end{array}$$

where TP represents the number of the true positive; TN, the number of the true negative; FP, the number of the false positive; FN, the number of the false negative; Sn, the sensitivity; Sp, the specificity; Acc, the accuracy; and MCC, the Mathew's correlation coefficient. To most biologists, however, the four metrics as formulated in (15) are not quite intuitive and easy to understand, particularly for the Mathew's correlation coefficient. Here let us adopt the formulation proposed recently in [27, 44] based on the symbols introduced by Chou [33] in predicting signal peptides. According to the formulation, the same four metrics can be written as

(16) $$\begin{array}{c} \text{Sn}=1-\frac{N_{-}^{+}}{N^{+}}\\ \text{Sp}=1-\frac{N_{+}^{-}}{N^{-}}\\ \text{Acc}=1-\frac{N_{-}^{+}+N_{+}^{-}}{N^{+}+N^{-}}\\ \text{MCC}=\frac{1-\left(N_{-}^{+}/N_{}^{+}+N_{+}^{-}/N^{-}\right)}{\sqrt{\left(1+\left(N_{+}^{-}-N_{-}^{+}\right)/N^{+}\right)\left(1+\left(N_{-}^{+}-N_{+}^{-}\right)/N^{-}\right)}}, \end{array}$$

where *N* ^+^ is the total number of the Arg-methylation (or Lys-methylation) peptides investigated, while *N*_−_^+^ is the number of the peptides incorrectly predicted as the non-Arg-methylation peptides, and *N* ^−^ is the total number of the non-Arg-methylation investigated, while *N*_+_^−^ is the number of the non-Arg-methylation incorrectly predicted as the Arg-methylation peptides [97].

Now, it is crystal clear from (16) that when *N*_−_^+^ = 0 meaning none of the Arg-methylation peptides was incorrectly predicted to be a non-Arg-methylation peptide, we have the sensitivity Sn = 1. When *N*_−_^+^ = *N* ^+^ meaning that all the Arg-methylation peptides were incorrectly predicted to be the non-Arg-methylation peptides, we have the sensitivity Sn = 0. Likewise, when *N*_+_^−^ = 0 meaning none of the non-Arg-methylation peptides was incorrectly predicted to be the Arg-methylation peptide, we have the specificity Sp = 1, whereas *N*_+_^−^ = *N* ^−^ meaning all the non-Arg-methylation peptides were incorrectly predicted as the Arg-methylation peptides, we have the specificity Sp = 0. When *N*_−_^+^ = *N*_+_^−^ = 0 meaning that none of Arg-methylation peptides in the positive dataset and none of the non-Arg-methylation peptides in the negative dataset was incorrectly predicted, we have the overall accuracy Acc = 1 and MCC = 1; when *N*_−_^+^ = *N* ^+^ and *N*_+_^−^ = *N* ^−^ meaning that all the Arg-methylation peptides in the positive dataset and all the non-Arg-methylation peptides in the negative dataset were incorrectly predicted, we have the overall accuracy Acc = 0 and MCC = −1, whereas when *N*_−_^+^ = *N* ^+^/2 and *N*_+_^−^ = *N* ^−^/2 we have Acc = 0.5 and MCC = 0 meaning no better than random prediction. As we can see from the above discussion based on (16), the meanings of sensitivity, specificity, overall accuracy, and Mathew's correlation coefficient have become much more intuitive and easier-to-understand.

### 2.5. Web Server and User Guide

For the convenience of the vast majority of biological scientists, a web server for iMethyl-PseAAC was established. Here, let us provide a step-by-step guide on how to use the web server to get the desired results without the need to follow the mathematic equations that were presented just for the integrity in developing the predictor.

**Step 1**

**Figure 3:**
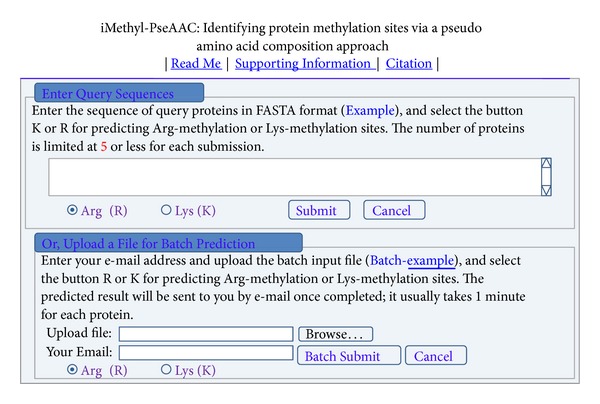
A semiscreenshot to show the top page of iMethyl-PseAAC. Its web-site address is at http://www.jci-bioinfo.cn/iMethyl-PseAAC.

Open the web server at http://www.jci-bioinfo.cn/iMethyl-PseAAC and you will see the top page of the predictor on your computer screen, as shown in Figure 3. Click on the* Read Me* button to see a brief introduction about iMethyl-PseAAC predictor and the caveat when using it.

**Step 2**

Either type or copy/paste the sequences of query proteins into the input box located at the center of Figure 3. The input should be in the FASTA format; only the 20 native amino acid codes are allowed in the protein sequences. Click the* Example* button to see the input format.

**Step 3**

Check on the “Arg” button for predicting the Arg-methylation sites, or “Lys” button for the Lys-methylation sites.

**Step 4**

Click the* Submit* button to see the predicted result. For example, if you use the sequences of the two query proteins in the* Example* window as the input and check the Arg button on, after clicking the* Submit* button, you will see the following predicted results. The total number of Arg (R) in the 1st protein (P62805) is 14, and the Arg at the sequence positions 4 and 41 (highlighted in red) is the methylation site, but the Arg at all the other 12 sites is not. The total number of Arg (R) in the 2nd protein (P68431) is 18, and the Arg at the sequence positions 3, 9, and 18 (highlighted in red) is the methylation site, but the Arg at all the other 15 sites is not. However, if you check the Lys button for the two query proteins, after clicking the* Submit* button, you will see that the total number of Lys (K) in the 1st protein (P62805) is 11, and the Lys at the sequence positions 13, 17, and 21 (highlighted in red) is the methylation site, but the Lys at all the other 8 sites is not, and that the total number of Lys (K) in the 2nd protein (P68431) is 13, of which, except the sequence position 116, the Lys at all the other 12 positions is the methylation site. A comparison of these predicted results with the experimental observations will be given in the Results and Discussion section. It takes about 30 seconds for the above computation before the predicted results appear on the computer screen; the more number of query proteins and longer of each sequence, the more time it is usually needed. The number of proteins is limited at 5 or less for each such direct submission.

**Step 5**

As shown on the lower panel of Figure 3, you may also choose the batch prediction by entering your e-mail address and your desired batch input file (in FASTA format) via the “Browse” button. To see the sample of batch input file, click on the button* Batch-example*. After clicking the button* Batch-submit*, you will see “Your batch job is under computation; once the results are available, you will be notified by e-mail.”

**Step 6**

Click the Citation button to see the relevant papers that document the detailed development and algorithm of iMethyl-PseAAC.

**Step 7**

Click on the Supporting Information button to download the benchmark dataset used to train and test the iMethyl-PseAAC predictor.

*Caveat. *To obtain the predicted result with the anticipated success rate, the entire sequence of the query protein rather than its fragment should be used as an input.

## 3. Results and Discussion

In statistical prediction, the following three cross-validation methods are often used to evaluate the anticipated accuracy of a predictor: independent dataset test, subsampling (K-fold cross-validation) test, and jackknife test [98]. However, as elucidated by a comprehensive review [24], among the three cross-validation methods, the jackknife test was deemed the least arbitrary and most objective because it could always yield a unique result for a given benchmark dataset and hence has been increasingly used and widely recognized by investigators to examine the accuracy of various predictors (see, e.g., [60, 61, 90, 99–101]). Therefore, in this study, we also adopted the jackknife test to examine the prediction quality of the iMethyl-PseAAC predictor.

It is instructive to point out that the number of positive samples and that of negative samples in the current benchmark dataset, for either Arg- or Lys-methylation system, are highly imbalanced. As shown in Online Supporting Information S3 and Online Supporting Information S4, the number of negative samples is about seven times the number of the positive samples. A general approach to treat this kind of highly sample-imbalanced system is to randomly separate the large set into several subsets and make each of them have about the same size of the small set.

The details for the subsets thus obtained for the current Arg-methylation and Lys-methylation systems are given in Online Supporting Information S5 and Online Supporting Information S6, respectively.

The jackknife rates achieved by iMethyl-PseAAC for the Arg-methylation system and Lys-methylation system are given in Tables 1 and 2, respectively. As we can see from the two tables, the average accuracy achieved by iMethyl-PseAAC for the Arg-methylation system was 76.19% and that for the Lys-methylation system was 70.74%. Meanwhile, we can also see that the corresponding MCCs (cf. (16)) were 52.74% and 41.66%, respectively, indicating that the prediction accuracy of iMethyl-PseAAC was quite stable, fully consistent with its sensitivity Sn and specificity Sp.

To further demonstrate its power, let us compare iMethyl-PseAAC with the existing predictors in this area. Only those predictors with a publicly accessible web server were qualified to be included in this study. Thus, the comparison will be made among the three predictors whose web servers are BPB-PPMS [20], PMeS [23], and iMethyl-PseAAC. Also, the best way to compare them is through practical application. To realize this, let us construct two independent datasets. One was for comparing the accuracy in identifying the Arg-methylation sites, and the other for Lys-methylation. The former contains 75 samples of which 20 are positive and 55 negative (see Online Supporting Information S7), while the latter contains 40 samples of which 14 are positive and 26 negative (see Online Supporting Information S8). To avoid the memory effect or bias in favor with iMethyl-PseAAC, none of the samples in the two independent datasets occurs in the datasets used to train the iMethyl-PseAAC predictor.

Listed in Tables 3 and 4 were the outcomes obtained by the three web-server predictors on the two independent datasets. As we can see from the two tables, the scores of the four metrics (cf. (16)) achieved by iMethyl-PseAAC were all remarkably higher than those by its counterparts except the rate of Sp for which iMethyl-PseAAC was tied with BPB-BPMS (see column 5 of Table 3) and about 11% lower than that of BPB-BPMS (see column 5 of Table 4). These results have clearly indicated that iMethyl-PseAAC is superior to its counterparts in predicting the Arg-methylation and Lys-methylation sites in proteins.

Finally, it is instructive to present an in-depth analysis to compare the experimental results with those reported in Step 4 of the “Web Server and User Guide.” According to experimental observations, the protein (P62805) has 103 amino acid residues and 14 Arg sites, of which only the 1st Arg (or the one located at the sequence position 4) is methylated, while all the other 13 Arg residues (or those located at the sequence positions 18, 20, 24, 36, 37, 40, 41, 46, 56, 68, 79, 93, and 96) are not methylated. Thus, we have *N* ^+^ = 1 and *N* ^−^ = 13 (cf. (16)). Since none of methylated Arg sites was incorrectly predicted as nonmethylated site and only one of the 13 nonmethylated Arg sites was incorrectly predicted as methylated sites, we have *N*_−_^+^ = 0 and *N*_+_^−^ = 1. Substituting these data into (16), we obtain Sn = 1, Sp = 0.92, Acc = 0.93, and MCC = 0.68.

The 2nd protein (P68431) has 136 amino acid residues and 18 Arg residues, of which the first three Arg residues (or those located at the sequence positions 3, 9, and 18) are methylated according to experimental observations. Thus, we have *N* ^+^ = 3 and *N* ^−^ = 15. Since none of the 3 methylated Arg sites was incorrectly predicted as nonmethylated and none of the 15 nonmethylated Arg sites was incorrectly predicted as methylated, we have *N*_−_^+^ = 0 and *N*_+_^−^ = 0. Substituting these data into (16), we obtain Sn = 1, Sp = 1, Acc = 1, and MCC = 1, meaning that the predicted result by iMethyl-PseAAC in the aforementioned Step 4 for protein (P68431) is perfectly correct.

Similar analysis can also be extended for the Lys-methylation. For example, the protein (P62805) has 11 Lys sites, of which only the 5th Lys (or the one located at the sequence position 21) was the methylated and all the other Lys residues (or those located at the sequence positions 6, 9, 13, 17, 32, 45, 60, 78, 80, and 92) were not according to experimental observations. Accordingly, its 3rd and 4th Lys residues were overpredicted by iMethyl-PseAAC as methylated. Thus we have *N* ^+^ = 1, *N* ^−^ = 10, *N*_−_^+^ = 0, and *N*_+_^−^ = 2. Substituting these data into (16), we obtain Sn = 1, Sp = 0.80, Acc = 0.82, and MCC = 0.63.

The 2nd protein (P68431) has 13 Lys residues, of which only the 3rd Lys (or the one located at sequence position 15) and 12th Lys (or the one located at the sequence position 116) are not methylated while all the other Lys residues (or those located at 5, 10, 19, 24, 28, 37, 38, 57, 65, 80, and 123) are methylated according to experimental observations. Thus, we have *N* ^+^ = 11 and *N* ^−^ = 2. Since none of the 11 methylated Lys sites was incorrectly predicted as nonmethylated site and only one of the 2 nonmethylated Lys sites was incorrectly predicted as the methylated site, we have *N*_−_^+^ = 0 and *N*_+_^−^ = 1. Substituting these data into (16), we obtain Sn = 1, Sp = 0.5, Acc = 0.92, and MCC = 0.68.

## 4. Conclusion

To timely acquire the information of Arg- and Lys-methylation sites in proteins is important for studying epigenetic inheritance in depth, analyzing various human diseases, and developing new drugs. It is anticipated that the iMethyl-PseAAC predictor may become a very useful high throughput tool in this regard. Its user-friendly web server and the step-by-step guide can help users easily to get their desired data.

## Supplementary Material

S1: An analysis of the benchmark dataset used in Hu et al. (Biopolymers, 2011, 95, 763-771)
S2: List of self-conflict samples in the benchmark dataset used in Hu et al. (Biopolymers, 2011, 95, 763-771)
S3: Benchmark dataset used for studying the Arg-methylation. It contains 1,481 samples, of which 185 are positive and 1,296 negative. These data were extracted from UniProtKB/Swiss-Prot database (version UniProt release 2013_06).
S4: Benchmark dataset used for studying the Lys-methylation. It contains 1,884 samples, of which 226 are positive and 1,518 negative. These data were extracted from UniProtKB/Swiss-Prot database (version UniProt release 2013_06).
S5: Seven negative subsets for studying Arg-methylation. Each subset contains 185 negative samples randomly taken from the 1,296 negative samples in Online Supporting Information S3 except for the 6th subset, which contains 186 samples. None of the samples in one subset occurs in any other subset.
S6: Seven negative subsets for studying Lys-methylation. Each subset contains 217 negative samples randomly taken from the 1,518 negative samples in Online Supporting Information S4 except for the 5th subset, which only contains 216 samples. None of the samples in one subset occurs in any other subset.
S7: Independent dataset for studying the Arg-methylation. It contains 75 samples, of which 20 are positive and 55 negative. None of the samples listed here occurs in Online Supporting Information S3.
S8: Independent dataset for studying the Lys-methylation. It contains 40 samples, of which 14 are positive and 26 negative. None of the samples listed here occurs in Online Supporting Information S4.
S9: The code for encoding the peptides investigated in this paper.

## Figures and Tables

**Table 1:** The metrics rates obtained by the jackknife test on the Arg-methylation system, where the positive dataset contains 185 samples (see Online Supporting Information S3), while the negative dataset consists of seven subsets with each containing 185 samples except for the 6th subset that contains 186 samples (see Online Supporting Information S5).

Negative subset	Acc (%)	MCC	Sn (%)	Sp (%)
1	72.16	0.45	64.32	80.00
2	78.65	0.57	79.46	77.84
3	73.24	0.47	66.49	80.00
4	78.71	0.57	78.38	79.03
5	77.30	0.55	70.81	83.78
6	75.68	0.51	75.68	75.68
7	77.57	0.56	67.57	87.57

Average	76.19	0.53	71.81	80.56

**Table 2:** The metrics rates obtained by the jackknife test on the Lys-methylation system, where the positive dataset contains 226 samples (see Online Supporting Information S4), while the negative dataset consists of seven subsets with each containing 217 samples except for the 5th subset that contains 216 samples (see Online Supporting Information S6).

Negative subset	Acc (%)	MCC	Sn (%)	Sp (%)
1	73.36	0.47	72.57	74.19
2	65.01	0.30	63.27	66.82
3	71.11	0.42	70.35	71.89
4	67.27	0.35	63.72	70.97
5	73.76	0.48	73.01	74.54
6	72.23	0.45	64.16	80.65
7	72.46	0.45	73.01	71.89

Average	70.74	0.42	68.58	72.99

**Table 3:** Comparison of iMethyl-PseAAC with the existing web-server predictors when tested for identifying Arg-methylation sites by the independent dataset (see Online Supporting Information S7).

Predictor	Acc (%)	MCC	Sn (%)	Sp (%)
PMeS^a^	76.00	0.45	70.00	78.18
BPB-PPMS^b^	93.33	0.83	85.00	96.36
iMethyl-PseAAC	97.33	0.94	100.00	96.36

^a^From [23].

^b^From [20].

**Table 4:** Comparison of iMethyl-PseAAC with the existing web-server predictors when tested for identifying Lys-methylation sites by the independent dataset (see Online Supporting Information S8).

Predictor	Acc (%)	MCC	Sn (%)	Sp (%)
PMeS^a^	65.00	0.35	78.57	57.69
BPB-PPMS^b^	70.00	0.36	64.29	73.08
iMethyl-PseAAC	75.00	0.60	100.00	61.54

^a^See footnote a of Table 3.

^b^See footnote b of Table 3.
