# Supplementary material for: iMethyl-PseAAC: Identification of Protein Methylation Sites via a Pseudo Amino Acid Composition Approach
Source: Biomed Res Int. 2014 May 22;2014:947416. doi: 10.1155/2014/947416 (PMC4054830; doi:10.1155/2014/947416)
Supplement: Supplementary file 1 — S1: An analysis of the benchmark dataset used in Hu et al. (Biopolymers, 2011, 95, 763-771) S2: List of self-conflict samples in the benchmark dataset used in Hu et al. (Biopolymers, 2011, 95, 763-771) S3: Benchmark dataset used for studying the Arg-methylation. It contains 1,481 samples, of which 185 are positive and 1,296 negative. These data were extracted from UniProtKB/Swiss-Prot database (version UniProt release 2013_06). S4: Benchmark dataset used for studying the Lys-methylation. It contains 1,884 samples, of which 226 are positive and 1,518 negative. These data were extracted from UniProtKB/Swiss-Prot database (version UniProt release 2013_06). S5: Seven negative subsets for studying Arg-methylation. Each subset contains 185 negative samples randomly taken from the 1,296 negative samples in Online Supporting Information S3 except for the 6th subset, which contains 186 samples. None of the samples in one subset occurs in any other subset. S6: Seven negative subsets for studying Lys-methylation. Each subset contains 217 negative samples randomly taken from the 1,518 negative samples in Online Supporting Information S4 except for the 5th subset, which only contains 216 samples. None of the samples in one subset occurs in any other subset. S7: Independent dataset for studying the Arg-methylation. It contains 75 samples, of which 20 are positive and 55 negative. None of the samples listed here occurs in Online Supporting Information S3. S8: Independent dataset for studying the Lys-methylation. It contains 40 samples, of which 14 are positive and 26 negative. None of the samples listed here occurs in Online Supporting Information S4. S9: The code for encoding the peptides investigated in this paper. [file 947416.f1.zip › S1.pdf]

Online Supporting Information S1. An analysis of the benchmark dataset used in Hu et al. (Biopolymers, 2011, 95, 763-771)

I. Arg-methylation dataset

(**A**) Of the 180 samples in their learning positive dataset, the following 5 pairs contain a duplicate sequence

Pair #	Protein	Site	Protein	Site	Duplicate sequence
1	P02686	241	P02688	131	SQGKGRGLSLS
2	P08199	656	P08199	666	GGFGGRGGGRG
3	P08199	660	P08199	674	GRGGGRGGFGG
4	P04256	194	P09651	194	ASSSQRGRSGS
5	Q92804	483	Q92804	570	GYGGDRGGYGG

(**B**) Of the 2,171 samples in their learning negative dataset, the following 64 pairs contain a duplicate sequence

Pair #	Protein	Site	Protein	Site	Duplicate sequence
1	P02686	139	P02688	6	MASQKRPSQRH
2	P02686	143	P02688	10	KRPSQRHGSKY
3	P02686	159	P02688	26	TMDHARHGFLP
4	P02686	165	P02688	32	HGFLPRHRDTG
5	P02686	167	P02688	34	FLPRHRDTGIL
6	P02686	231	P02688	121	NIVTPRTPPPS
7	P02686	247	P02688	137	GLSLSRFSWGA
8	P02686	264	P02688	154	FGYGGRASDYK
9	P02686	293	P02688	184	FKLGGRDSRSG
10	P02686	296	P02688	187	GGRDSRSGSPM
11	P02686	303	P02688	194	GSPMARR----
12	P02686	304	P02688	195	SPMARR-----
13	Q07955	210	Q07955	212	SRSRSRSRSRS
14	Q07955	210	Q07955	214	SRSRSRSRSRS
15	P04264	199	P08729	110	FIDKVRFLEQQ
16	P04256	14	P09651	14	EPEQLRKLFIG
17	P04256	31	P09651	31	TDESLRSHFEQ
18	P04256	47	P09651	47	DCVVMRDPNTK
19	P04256	53	P09651	53	DPNTKRSRGFG
20	P04256	55	P09651	55	NTKRSRGFGFV
21	P04256	75	P09651	75	AAMNARPHKVD
22	P04256	82	P09651	82	HKVDGRVVEPK
23	P04256	88	P09651	88	VVEPKRAVSRE
24	P04256	92	P09651	92	KRAVSREDSQR
25	P04256	97	P09651	97	REDSQRPGAHL
26	P04256	122	P09651	122	EEHHLRDYFEQ

27	P04256	140	P09651	140	EIMTDRGSGKK
28	P04256	146	P09651	146	GSGKKRGFAFV
29	P04256	196	P09651	196	SSQRGRSGSGN
30	P04256	218	P09651	218	NDNFGRGGNFS
31	P04256	232	P09651	232	GFGGSRGGGGY
32	P04256	284	P09651	336	GNFGGRSSGPY
33	P04256	300	P09651	352	YFAKPRNQGGY
34	P04256	318	P09651	370	SYGSGRRF---
35	P04256	319	P09651	371	YGSGRRF----
36	P11940	44	Q13310	44	SIRVCRDMITR
37	P11940	49	Q13310	49	RDMITRRSLGY
38	P11940	50	Q13310	50	DMITRRSLGYA
39	P11940	67	Q13310	67	PADAERALDTM
40	P11940	89	Q13310	89	IMWSQRDPSLR
41	P11940	94	Q13310	94	RDPSLRKSGVG
42	P11940	166	Q13310	166	MLLNDRKVFVG
43	P11940	172	Q13310	172	KVFVGRFKSRK
44	P11940	176	Q13310	176	GRFKSRKEREA
45	P11940	179	Q13310	179	KSRKEREAELG
46	P11940	565	Q13310	574	QMLGERLFPLI
47	P11940	604	Q13310	613	SPESLRSKVDE
48	Q13151	139	P22626	153	QSGKKRGFGFV
49	P70372	277	P26378	330	NVKVIRDFNTN
50	Q92804	422	Q92804	450	GYGGDRSGGGY
51	Q92804	459	Q92804	467	GYGGDRGGGYG
52	Q92804	459	Q92804	475	GYGGDRGGGYG
53	Q92804	459	Q92804	490	GYGGDRGGGYG
54	P31942	84	P31943	179	ERIGHRYIEIF
55	Q92804	422	Q92804	553	GYGGDRSGGGY
56	Q92804	459	Q92804	562	GYGGDRGGGYG
57	Q06787	245	P51114	245	NIQQARKVPGV
58	Q13283	13	Q9UN86	13	PLLVGREFVRQ
59	Q13283	17	Q9UN86	17	GREFVRQYYTL
60	Q13283	78	Q9UN86	78	CHTKIRHVDAH
61	P04256	14	P51991	35	EPEQLRKLFIG
62	P04256	82	P51991	103	HKVDGRVVEPK
63	P04256	88	P51991	109	VVEPKRAVSRE
64	Q13283	407	Q9UN86	401	FRGEVRLNVEE

(**C**) Of the 10 samples in their testing positive dataset, the following 3 pairs contain a duplicate sequence

Pair #	Protein	Site	Protein	Site	Duplicate sequence
1	P81558	107	P02687	106	SQGKGRGLSLS
2	P81558	107	P83487	108	SQGKGRGLSLS
3	P81558	107	P06906	107	SQGKGRGLSLS

(**D**) Of the 206 samples in their testing negative dataset, the following 46 pairs contain a duplicate sequence

Pair #	Protein	Site	Protein	Site	Duplicate sequence
1	P25274	5	P81558	5	-ASQKRPSQRH
2	P25274	9	P81558	9	KRPSQRHGSKY
3	P25274	25	P81558	25	TMDHARHGFLP
4	P25274	31	P81558	31	HGFLPRHRDTG
5	P25274	25	P02687	23	TMDHARHGFLP
6	P25274	31	P02687	29	HGFLPRHRDTG
7	P25274	33	P02687	31	FLPRHRDTGIL
8	P25274	54	P81558	53	RGAPKRGSGKD
9	P25274	54	P02687	52	RGAPKRGSGKD
10	P81558	64	P02687	63	GHHAARTTHYG
11	P25274	95	P81558	97	NIVTPRTPPPS
12	P25274	95	P02687	96	NIVTPRTPPPS
13	P81558	113	P02687	112	GLSLSRFSWGA
14	P25274	157	P81558	160	FKLGGRDSRSG
15	P25274	160	P81558	163	GGRDSRSGSPM
16	P25274	157	P02687	158	FKLGGRDSRSG
17	P25274	167	P81558	170	GSPMARR----
18	P25274	160	P02687	161	GGRDSRSGSPM
19	P25274	168	P81558	171	SPMARR-----
20	P25274	167	P02687	168	GSPMARR----
21	P25274	5	P83487	5	-ASQKRPSQRH
22	P25274	168	P02687	169	SPMARR-----
23	P25274	9	P83487	9	KRPSQRHGSKY
24	P25274	5	P06906	5	-ASQKRPSQRH
25	P25274	25	P83487	25	TMDHARHGFLP
26	P25274	9	P06906	9	KRPSQRHGSKY
27	P25274	31	P83487	31	HGFLPRHRDTG
28	P25274	25	P06906	25	TMDHARHGFLP
29	P25274	33	P83487	33	FLPRHRDTGIL
30	P25274	31	P06906	31	HGFLPRHRDTG
31	P25274	33	P06906	33	FLPRHRDTGIL
32	P81558	64	P83487	65	GHHAARTTHYG
33	P25274	54	P06906	54	RGAPKRGSGKD
34	P25274	95	P06906	97	NIVTPRTPPPS
35	P81558	113	P83487	114	GLSLSRFSWGA
36	P81558	113	P06906	113	GLSLSRFSWGA
37	P02687	129	P06906	130	FGYGGRASDYK
38	P25274	157	P83487	161	FKLGGRDSRSG
39	P25274	157	P06906	160	FKLGGRDSRSG
40	P25274	160	P83487	164	GGRDSRSGSPM
41	P25274	160	P06906	163	GGRDSRSGSPM
42	P25274	167	P83487	171	GSPMARR----

43	P25274	167	P06906	170	GSPMARR----
44	P25274	168	P83487	172	SPMARR-----
45	P25274	168	P06906	171	SPMARR-----
46	O09032	323	Q15717	277	NVKVIRDFNTN

II. Lys-methylation dataset

(**A**) Of the 262 samples in their learning positive dataset, the following 3 pairs contain a duplicate sequence

Pair #	Protein	Site	Protein	Site	Duplicate sequence
1	P81558	107	P02687	106	SQGKGKGLSLS
2	P81558	107	P83487	108	SQGKGKGLSLS
3	P81558	107	P06906	107	SQGKGKGLSLS

(**B**) Of the 2,569 samples in their learning negative dataset, the following 506 pairs contain a duplicate sequence

Pair #	Protein	Site	Protein	Site	Duplicate sequence
1	P00042	85	P00043	86	YIPGTKMAFGG
2	P00042	17	P00044	17	GATLFKTRCLQ
3	P00042	28	P00044	28	CHTVEKGGPHK
4	P00042	33	P00044	33	KGGPHKVGPNL
5	P00042	85	P00044	85	YIPGTKMAFGG
6	P00042	92	P00044	92	AFGGLKKEKDR
7	P00042	93	P00044	93	FGGLKKEKDRN
8	P00042	95	P00044	95	GLKKEKDRNDL
9	P00047	19	P00048	16	GANLFKTRCAQ
10	P00042	85	P00048	84	YIPGTKMAFGG
11	P00051	19	P00060	19	GEKIFKTKCAQ
12	P00051	21	P00060	21	KIFKTKCAQCH
13	P00051	81	P00060	81	LLNPKKYIPGT
14	P00051	87	P00060	87	YIPGTKMVFPG
15	P00051	107	P00060	107	LIAYLKEATA-
16	P00060	12	P00061	12	PPGNPKAGEKI
17	P00060	16	P00061	16	PKAGEKIFKTK
18	P00051	19	P00061	19	GEKIFKTKCAQ
19	P00051	21	P00061	21	KIFKTKCAQCH
20	P00051	30	P00061	30	CHTVDKGAGHK
21	P00060	35	P00061	35	KGAGHKEGPNL
22	P00051	81	P00061	81	LLNPKKYIPGT
23	P00051	87	P00061	87	YIPGTKMVFPG
24	P00060	95	P00061	95	FPGLKKPQERA

25	P00051	107	P00061	107	LIAYLKEATA-
26	P00060	12	P00062	12	PPGNPKAGEKI
27	P00060	16	P00062	16	PKAGEKIFKTK
28	P00051	30	P00062	30	CHTVDKGAGHK
29	P00051	35	P00062	35	KGAGHKQGPNL
30	P00060	61	P00062	61	YSAANKNMAVN
31	P00051	81	P00062	81	LLNPKKYIPGT
32	P00051	87	P00062	87	YIPGTKMVFPG
33	P00051	95	P00062	95	FPGLKKPQDRA
34	P00051	19	P00063	19	GEKIFKTKCAQ
35	P00051	21	P00063	21	KIFKTKCAQCH
36	P00051	30	P00063	30	CHTVDKGAGHK
37	P00051	35	P00063	35	KGAGHKQGPNL
38	P00060	61	P00063	61	YSAANKNMAVN
39	P00051	81	P00063	81	LLNPKKYIPGT
40	P00051	87	P00063	87	YIPGTKMVFPG
41	P00051	95	P00063	95	FPGLKKPQDRA
42	P00051	21	P00068	21	KIFKTKCAQCH
43	P00051	81	P00068	81	LLNPKKYIPGT
44	P00051	87	P00068	87	YIPGTKMVFPG
45	P00051	95	P00068	95	FPGLKKPQDRA
46	P00051	19	P00070	20	GEKIFKTKCAQ
47	P00051	21	P00070	22	KIFKTKCAQCH
48	P00060	30	P00070	31	CHTVEKGAGHK
49	P00051	35	P00070	36	KGAGHKQGPNL
50	P00051	81	P00070	82	LLNPKKYIPGT
51	P00051	87	P00070	88	YIPGTKMVFPG
52	P00060	95	P00070	96	FPGLKKPQERA
53	P00068	19	P00073	19	GAKIFKTKCAQ
54	P00051	21	P00073	21	KIFKTKCAQCH
55	P00051	81	P00073	81	LLNPKKYIPGT
56	P00051	87	P00073	87	YIPGTKMVFPG
57	P00051	95	P00073	95	FPGLKKPQDRA
58	P22342	7	P00076	7	DAERGKKLFES
59	P22342	8	P00076	8	AERGKKLFESR
60	P22342	52	P00076	52	YSNANKNAAIV
61	P22342	71	P00076	71	FLENPKKYVPG
62	P22342	72	P00076	72	LENPKKYVPGT
63	P22342	78	P00076	78	YVPGTKMAFAG
64	P22342	98	P00076	98	IIAYMKTLKD-
65	P22342	101	P00076	101	YMKTLKD----
66	P00077	84	P00078	84	LENPKKFMPGT
67	P00077	90	P00078	90	FMPGTKMSFAG
68	P25079	8	P00876	8	PQTETKASVGF
69	P25079	18	P00876	18	FKAGVKEYKLT
70	P25079	21	P00876	21	GVKEYKLTYYT
71	P25079	32	P00876	32	PEYQTKDTDIL
72	P25079	81	P00876	81	SLDRYKGRCYR

73	P25079	94	P00876	94	RVVGEKDQYIA
74	P25079	128	P00876	128	NVFGFKALRAL
75	P25079	161	P00876	161	QVERDKLNKYG
76	P25079	164	P00876	164	RDKLNKYGRPL
77	P25079	175	P00876	175	LGCTIKPKLGL
78	P25079	177	P00876	177	CTIKPKLGLSA
79	P25079	183	P00876	183	LGLSAKNYGRA
80	P25079	201	P00876	201	GLDFTKDDENV
81	P25079	236	P00876	236	ETGEIKGHYLN
82	P25079	316	P00876	316	FRVLAKALRMS
83	P25079	334	P00876	334	GTVVGKLEGER
84	P25079	429	P00876	429	LEACVKARNEG
85	P25079	463	P00876	463	ACEVWKEIVFN
86	P25079	8	P27063	6	PQTETKASVGF
87	P25079	8	P27065	8	PQTETKASVGF
88	P25079	18	P27065	18	FKAGVKEYKLT
89	P25079	21	P27065	21	GVKEYKLTYYT
90	P25079	32	P27065	32	PEYQTKDTDIL
91	P25079	81	P27065	81	SLDRYKGRCYR
92	P25079	94	P27065	94	RVVGEKDQYIA
93	P25079	128	P27065	128	NVFGFKALRAL
94	P00876	146	P27065	146	PPAYVKTFQGP
95	P25079	161	P27065	161	QVERDKLNKYG
96	P25079	164	P27065	164	RDKLNKYGRPL
97	P25079	175	P27065	175	LGCTIKPKLGL
98	P25079	177	P27065	177	CTIKPKLGLSA
99	P25079	183	P27065	183	LGLSAKNYGRA
100	P25079	201	P27065	201	GLDFTKDDENV
101	P25079	236	P27065	236	ETGEIKGHYLN
102	P00876	252	P27065	252	CEEMIKRAVFA
103	P00876	305	P27065	305	VIDRQKNHGIH
104	P25079	316	P27065	316	FRVLAKALRMS
105	P25079	334	P27065	334	GTVVGKLEGER
106	P25079	429	P27065	429	LEACVKARNEG
107	P00876	450	P27065	450	IREACKWSPEL
108	P25079	463	P27065	463	ACEVWKEIVFN
109	P00876	477	P27065	477	VDVLDK-----
110	P25079	8	P27066	8	PQTETKASVGF
111	P25079	128	P27066	128	NVFGFKALRAL
112	P25079	161	P27066	161	QVERDKLNKYG
113	P25079	164	P27066	164	RDKLNKYGRPL
114	P25079	175	P27066	175	LGCTIKPKLGL
115	P25079	177	P27066	177	CTIKPKLGLSA
116	P25079	183	P27066	183	LGLSAKNYGRA
117	P25079	201	P27066	201	GLDFTKDDENV
118	P25079	236	P27066	236	ETGEIKGHYLN
119	P25079	252	P27066	252	CEEMMKRAVFA
120	P25079	305	P27066	305	VIDRQKNHGMH

121	P25079	334	P27066	334	GTVVGKLEGER
122	P21251	14	P02598	14	QIAEFKEAFSL
123	P21251	22	P02598	22	FSLFDKDGDGT
124	P21251	31	P02598	31	GTITTKELGTV
125	P02993	20	P02994	20	HVDSGKSTTTG
126	P02993	41	P02994	41	KRTIEKFEKEA
127	P02993	55	P02994	55	GKGSFKYAWVL
128	P02993	62	P02994	62	AWVLDKLKAER
129	P02993	64	P02994	64	VLDKLKAERER
130	P02993	100	P02994	100	HRDFIKNMITG
131	P02993	255	P02994	253	LQDVYKIGGIG
132	P43286	63	P43287	61	TVIGYKIQSDT
133	P02993	313	P02994	311	VGFNVKNVSVK
134	P43286	144	P43287	142	GVGFVKAFQSS
135	P43286	241	P43287	239	AVIYNKSKPWD
136	P43286	243	P43287	241	IYNKSKPWDDH
137	P43286	276	P43287	274	RASGSKSLGSF
138	P25886	3	P47914	3	---MAKSKNHT
139	P25886	15	P47914	15	HNQSRKWHRNG
140	P25886	22	P47914	22	HRNGIKKPRSQ
141	P25886	23	P47914	23	RNGIKKPRSQR
142	P25886	33	P47914	33	RYESLKGVDPK
143	P25886	38	P47914	38	KGVDPKFLRNM
144	P25886	47	P47914	47	NMRFAKKHNKK
145	P25886	48	P47914	48	MRFAKKHNKKG
146	P25886	51	P47914	51	AKKHNKKGLKK
147	P25886	52	P47914	52	KKHNKKGLKKM
148	P25886	55	P47914	55	NKKGLKKMQAN
149	P25886	56	P47914	56	KKGLKKMQANN
150	P25886	73	P47914	73	RAEAIKALVKP
151	P21251	14	P04464	14	QIAEFKEAFSL
152	P21251	78	P04464	78	MARKMKDTDSE
153	P00219	73	P55907	74	HPASEKKADPV
154	P00219	74	P55907	75	PASEKKADPVN
155	P00219	101	P55907	102	AAIDVKPP---
156	O60814	6	P57053	6	MPEPAKSAPAP
157	O60814	12	P57053	12	SAPAPKKGSKK
158	O60814	13	P57053	13	APAPKKGSKKA
159	O60814	16	P57053	16	PKKGSKKAVTK
160	O60814	17	P57053	17	KKGSKKAVTKA
161	O60814	21	P57053	21	KKAVTKAQKKD
162	O60814	35	P57053	35	RKRSRKESYSV
163	O60814	44	P57053	44	SVYVYKVLKQV
164	O60814	117	P57053	117	VSEGTKAVTKY
165	O60814	121	P57053	121	TKAVTKYTSAK
166	O60814	126	P57053	126	KYTSAK-----
167	P25079	8	P04992	8	PQTETKASVGF
168	P25079	18	P04992	18	FKAGVKEYKLT

169	P25079	21	P04992	21	GVKEYKLTYYT
170	O15819	57	P59226	54	LREIRKYQKST
171	P25079	128	P04992	128	NVFGFKALRAL
172	P00876	146	P04992	146	PPAYVKTFQGP
173	P25079	161	P04992	161	QVERDKLNKYG
174	P25079	164	P04992	164	RDKLNKYGRPL
175	O15819	119	P59226	116	CAIHAKRVTIM
176	P25079	175	P04992	175	LGCTIKPKLGL
177	P25079	177	P04992	177	CTIKPKLGLSA
178	P25079	183	P04992	183	LGLSAKNYGRA
179	P25079	201	P04992	201	GLDFTKDDENV
180	P25079	252	P04992	252	CEEMMKRAVFA
181	P00876	305	P04992	305	VIDRQKNHGIH
182	P25079	316	P04992	316	FRVLAKALRMS
183	P25079	334	P04992	334	GTVVGKLEGER
184	P00876	450	P04992	450	IREACKWSPEL
185	P59226	65	P61830	65	ELLIRKLPFQR
186	P00876	477	P04992	477	VDVLDK-----
187	P39476	21	P61991	21	DISKIKKVWRV
188	P39476	22	P61991	22	ISKIKKVWRVG
189	P39476	28	P61991	28	VWRVGKMISFT
190	P39476	40	P61991	40	DEGGGKTGRGA
191	P39476	49	P61991	49	GAVSEKDAPKE
192	P39476	53	P61991	53	EKDAPKELLQM
193	P21251	14	P62157	14	QIAEFKEAFSL
194	P21251	22	P62157	22	FSLFDKDGDGT
195	P21251	31	P62157	31	GTITTKELGTV
196	P21251	76	P62157	76	TMMARKMKDTD
197	P21251	78	P62157	78	MARKMKDTDSE
198	P21251	95	P62157	95	FRVFDKDGNGY
199	P21251	14	P62184	14	QIAEFKEAFSL
200	P21251	22	P62184	22	FSLFDKDGDGT
201	P21251	31	P62184	31	GTITTKELGTV
202	P21251	76	P62184	76	TMMARKMKDTD
203	P21251	78	P62184	78	MARKMKDTDSE
204	P62784	6	P62787	6	MSGRGKGGKGL
205	P62784	9	P62787	9	RGKGGKGLGKG
206	P62784	13	P62787	13	GKGLGKGGAKR
207	P62784	17	P62787	17	GKGGAKRHRKV
208	P62784	32	P62787	32	IQGITKPAIRR
209	P62784	45	P62787	45	RRGGVKRISGL
210	P62784	92	P62787	92	VVYALKRQGRT
211	P62784	6	P62803	6	MSGRGKGGKGL
212	P62784	9	P62803	9	RGKGGKGLGKG
213	P62784	13	P62803	13	GKGLGKGGAKR
214	P62784	17	P62803	17	GKGGAKRHRKV
215	P62784	32	P62803	32	IQGITKPAIRR
216	P62784	45	P62803	45	RRGGVKRISGL

217	O60814	6	P06899	6	MPEPAKSAPAP
218	P62784	60	P62803	60	TRGVLKVFLEN
219	O60814	12	P06899	12	SAPAPKKGSKK
220	O60814	13	P06899	13	APAPKKGSKKA
221	P62784	80	P62803	80	EHAKRKTVTAM
222	O60814	16	P06899	16	PKKGSKKAVTK
223	P62784	92	P62803	92	VVYALKRQGRT
224	O60814	17	P06899	17	KKGSKKAVTKA
225	O60814	21	P06899	21	KKAVTKAQKKD
226	O60814	24	P06899	24	VTKAQKKDGKK
227	O60814	25	P06899	25	TKAQKKDGKKR
228	O60814	28	P06899	28	QKKDGKKRKRS
229	O60814	29	P06899	29	KKDGKKRKRSR
230	O60814	31	P06899	31	DGKKRKRSRKE
231	O60814	86	P06899	86	LAHYNKRSTIT
232	O60814	117	P06899	117	VSEGTKAVTKY
233	O60814	121	P06899	121	TKAVTKYTSAK
234	O60814	126	P06899	126	KYTSAK-----
235	P59226	15	P07041	15	KSTGGKAPRKQ
236	P02993	20	P68104	20	HVDSGKSTTTG
237	P02993	30	P68104	30	GHLIYKCGGID
238	P02993	41	P68104	41	KRTIEKFEKEA
239	P61830	38	P07041	38	TGGVKKPHRYK
240	P61830	43	P07041	43	KPHRYKPGTVA
241	P02993	55	P68104	55	GKGSFKYAWVL
242	P59226	65	P07041	65	ELLIRKLPFQR
243	P02993	62	P68104	62	AWVLDKLKAER
244	P02993	64	P68104	64	VLDKLKAERER
245	P02993	100	P68104	100	HRDFIKNMITG
246	P02993	129	P68104	129	EAGISKNGQTR
247	P02993	146	P68104	146	YTLGVKQLIVG
248	P02993	154	P68104	154	IVGVNKMDSTE
249	P02993	255	P68104	255	LQDVYKIGGIG
250	P59226	15	P08898	15	KSTGGKAPRKQ
251	P02994	288	P68104	290	VTTEVKSVEMH
252	P02993	313	P68104	313	VGFNVKNVSVK
253	P07041	57	P08898	57	IRRYQKSTELL
254	O15819	119	P08898	116	CAIHAKRVTIM
255	P59226	123	P08898	123	VTIMPKDIQLA
256	P59226	15	P08903	15	KSTGGKAPRKQ
257	P59226	38	P08903	38	TGGVKKPHRFR
258	P07041	57	P08903	57	IRRYQKSTELL
259	P59226	65	P08903	65	ELLIRKLPFQR
260	P59226	80	P08903	80	IAQDFKTDLRF
261	O15819	119	P08903	116	CAIHAKRVTIM
262	P59226	123	P08903	123	VTIMPKDIQLA
263	P59226	38	P68429	38	TGGVKKPHRFR
264	O15819	57	P68429	54	LREIRKYQKST

265	P59226	57	P68429	57	IRKYQKSTELL
266	P59226	65	P68429	65	ELLIRKLPFQR
267	P59226	80	P68429	80	IAQDFKTDLRF
268	O15819	119	P68429	116	CAIHAKRVTIM
269	P59226	123	P68429	123	VTIMPKDIQLA
270	P59226	15	P68432	15	KSTGGKAPRKQ
271	P07472	30	P0A7K2	30	SAMEEKFGVSA
272	P02393	66	P0A7K2	60	FDVILKAAGAN
273	P07041	57	P68432	57	IRRYQKSTELL
274	P59226	65	P68432	65	ELLIRKLPFQR
275	P59226	80	P68432	80	IAQDFKTDLRF
276	O15819	119	P68432	116	CAIHAKRVTIM
277	P59226	123	P68432	123	VTIMPKDIQLA
278	P02393	127	P0A7K2	121	AEVEVK-----
279	P19984	116	P68696	116	ANVVEKLADYL
280	P16403	46	P10412	46	SELITKAVAAS
281	P16403	52	P10412	52	AVAASKERSGV
282	P16403	63	P10412	63	SLAALKKALAA
283	P16403	64	P10412	64	LAALKKALAAA
284	P16403	75	P10412	75	GYDVEKNNSRI
285	P16403	81	P10412	81	NNSRIKLGLKS
286	P16403	85	P10412	85	IKLGLKSLVSK
287	P16403	90	P10412	90	KSLVSKGTLVQ
288	P16403	97	P10412	97	TLVQTKGTGAS
289	P16403	106	P10412	106	ASGSFKLNKKA
290	P16403	109	P10412	109	SFKLNKKAASG
291	P16403	110	P10412	110	FKLNKKAASGE
292	P62784	6	P82888	6	MSGRGKGGKGL
293	P62784	9	P82888	9	RGKGGKGLGKG
294	P62784	13	P82888	13	GKGLGKGGAKR
295	P62784	32	P82888	32	IQGITKPAIRR
296	P62784	45	P82888	45	RRGGVKRISGL
297	P62784	60	P82888	60	TRGVLKVFLEN
298	P62784	92	P82888	92	VVYALKRQGRT
299	P16403	152	P10412	152	PKKSAKKTPKK
300	P16403	153	P10412	153	KKSAKKTPKKA
301	P16403	156	P10412	156	AKKTPKKAKKP
302	P16403	157	P10412	157	KKTPKKAKKPA
303	P16403	159	P10412	159	TPKKAKKPAAA
304	P68432	38	P84229	38	TGGVKKPHRYR
305	P07041	57	P84229	57	IRRYQKSTELL
306	P59226	65	P84229	65	ELLIRKLPFQR
307	O15819	119	P84229	116	CAIHAKRVTIM
308	P59226	123	P84229	123	VTIMPKDIQLA
309	P16403	196	P10412	197	KAVKPKAAKPK
310	P07041	57	P84249	57	IRRYQKSTELL
311	P59226	65	P84249	65	ELLIRKLPFQR
312	O15819	119	P84249	116	CAIHAKRVTIM

313	P59226	123	P84249	123	VTIMPKDIQLA
314	P00060	12	Q0DI31	13	PPGNPKAGEKI
315	P00060	16	Q0DI31	17	PKAGEKIFKTK
316	P00051	19	Q0DI31	20	GEKIFKTKCAQ
317	P00051	21	Q0DI31	22	KIFKTKCAQCH
318	P00051	30	Q0DI31	31	CHTVDKGAGHK
319	P00051	35	Q0DI31	36	KGAGHKQGPNL
320	P00051	81	Q0DI31	82	LLNPKKYIPGT
321	P00051	87	Q0DI31	88	YIPGTKMVFPG
322	P00060	95	Q0DI31	96	FPGLKKPQERA
323	P59226	15	Q16695	15	KSTGGKAPRKQ
324	P68432	37	Q16695	37	ATGGVKKPHRY
325	P68432	38	Q16695	38	TGGVKKPHRYR
326	P07041	57	Q16695	57	IRRYQKSTELL
327	P59226	65	Q16695	65	ELLIRKLPFQR
328	P59226	80	Q16695	80	IAQDFKTDLRF
329	P59226	123	Q16695	123	VTIMPKDIQLA
330	O60814	6	Q16778	6	MPEPAKSAPAP
331	O60814	12	Q16778	12	SAPAPKKGSKK
332	O60814	13	Q16778	13	APAPKKGSKKA
333	O60814	16	Q16778	16	PKKGSKKAVTK
334	O60814	17	Q16778	17	KKGSKKAVTKA
335	O60814	21	Q16778	21	KKAVTKAQKKD
336	O60814	24	Q16778	24	VTKAQKKDGKK
337	O60814	25	Q16778	25	TKAQKKDGKKR
338	O60814	28	Q16778	28	QKKDGKKRKRS
339	O60814	29	Q16778	29	KKDGKKRKRSR
340	O60814	31	Q16778	31	DGKKRKRSRKE
341	P06899	35	Q16778	35	RKRSRKESYSI
342	P06899	44	Q16778	44	SIYVYKVLKQV
343	O60814	86	Q16778	86	LAHYNKRSTIT
344	O60814	117	Q16778	117	VSEGTKAVTKY
345	P59226	15	Q42681	15	KSTGGKAPRKQ
346	O15819	57	Q42681	53	LREIRKYQKST
347	P59226	80	Q42681	79	IAQDFKTDLRF
348	O15819	119	Q42681	115	CAIHAKRVTIM
349	P59226	123	Q42681	122	VTIMPKDIQLA
350	P68104	3	Q5VTE0	3	---MGKEKTHI
351	P68104	5	Q5VTE0	5	-MGKEKTHINI
352	P02993	20	Q5VTE0	20	HVDSGKSTTTG
353	P02993	30	Q5VTE0	30	GHLIYKCGGID
354	P02993	41	Q5VTE0	41	KRTIEKFEKEA
355	P68104	44	Q5VTE0	44	IEKFEKEAAEM
356	P68104	51	Q5VTE0	51	AAEMGKGSFKY
357	P02993	62	Q5VTE0	62	AWVLDKLKAER
358	P02993	64	Q5VTE0	64	VLDKLKAERER
359	P68104	79	Q5VTE0	79	DISLWKFETSK
360	P68104	84	Q5VTE0	84	KFETSKYYVTI

361	P02993	100	Q5VTE0	100	HRDFIKNMITG
362	P02993	129	Q5VTE0	129	EAGISKNGQTR
363	P02993	146	Q5VTE0	146	YTLGVKQLIVG
364	P02993	154	Q5VTE0	154	IVGVNKMDSTE
365	P68104	172	Q5VTE0	172	YEEIVKEVSTY
366	P68104	179	Q5VTE0	179	VSTYIKKIGYN
367	P68104	180	Q5VTE0	180	STYIKKIGYNP
368	P68104	212	Q5VTE0	212	NMPWFKGWKVT
369	P68104	215	Q5VTE0	215	WFKGWKVTRKD
370	P68104	219	Q5VTE0	219	WKVTRKDGNAS
371	P68104	244	Q5VTE0	244	TRPTDKPLRLP
372	P02993	255	Q5VTE0	255	LQDVYKIGGIG
373	P68104	273	Q5VTE0	273	ETGVLKPGMVV
374	P02994	288	Q5VTE0	290	VTTEVKSVEMH
375	P68104	318	Q5VTE0	318	KNVSVKDVRRG
376	P68104	330	Q5VTE0	330	VAGDSKNDPPM
377	P68104	371	Q5VTE0	371	AHIACKFAELK
378	P68104	376	Q5VTE0	376	KFAELKEKIDR
379	P68104	378	Q5VTE0	378	AELKEKIDRRS
380	P68104	385	Q5VTE0	385	DRRSGKKLEDG
381	P68104	386	Q5VTE0	386	RRSGKKLEDGP
382	P68104	392	Q5VTE0	392	LEDGPKFLKSG
383	P68104	395	Q5VTE0	395	GPKFLKSGDAA
384	P68104	408	Q5VTE0	408	DMVPGKPMCVE
385	P68104	439	Q5VTE0	439	AVGVIKAVDKK
386	P68104	443	Q5VTE0	443	IKAVDKKAAGA
387	P68104	444	Q5VTE0	444	KAVDKKAAGAG
388	P68104	450	Q5VTE0	450	AAGAGKVTKSA
389	P68104	453	Q5VTE0	453	AGKVTKSAQKA
390	P68104	457	Q5VTE0	457	TKSAQKAQKAK
391	P68104	460	Q5VTE0	460	AQKAQKAK---
392	P68104	462	Q5VTE0	462	KAQKAK-----
393	P68104	3	Q71V39	3	---MGKEKTHI
394	P68104	5	Q71V39	5	-MGKEKTHINI
395	P02993	20	Q71V39	20	HVDSGKSTTTG
396	P59226	15	P10651	15	KSTGGKAPRKQ
397	P02993	30	Q71V39	30	GHLIYKCGGID
398	P02993	41	Q71V39	41	KRTIEKFEKEA
399	P68104	44	Q71V39	44	IEKFEKEAAEM
400	P68104	51	Q71V39	51	AAEMGKGSFKY
401	P68432	38	P10651	38	TGGVKKPHRYR
402	P07041	57	P10651	57	IRRYQKSTELL
403	P02993	62	Q71V39	62	AWVLDKLKAER
404	P59226	65	P10651	65	ELLIRKLPFQR
405	P02993	64	Q71V39	64	VLDKLKAERER
406	P59226	80	P10651	80	IAQDFKTDLRF
407	P02993	100	Q71V39	100	HRDFIKNMITG
408	P21251	14	P11118	14	QIAEFKEAFSL

409	P02993	129	Q71V39	129	EAGISKNGQTR
410	P21251	22	P11118	22	FSLFDKDGDGT
411	P02993	146	Q71V39	146	YTLGVKQLIVG
412	P21251	31	P11118	31	GTITTKELGTV
413	P02993	154	Q71V39	154	IVGVNKMDSTE
414	P02993	179	Q71V39	179	VSAYIKKIGYN
415	P02993	180	Q71V39	180	SAYIKKIGYNP
416	P68104	244	Q71V39	244	TRPTDKPLRLP
417	P02993	255	Q71V39	255	LQDVYKIGGIG
418	P02993	290	Q71V39	290	ITTEVKSVEMH
419	P02993	313	Q71V39	313	VGFNVKNVSVK
420	P68104	371	Q71V39	371	AHIACKFAELK
421	P68104	376	Q71V39	376	KFAELKEKIDR
422	P68104	378	Q71V39	378	AELKEKIDRRS
423	P68104	453	Q71V39	453	AGKVTKSAQKA
424	Q13310	30	P11940	30	AMLYEKFSPAG
425	Q13310	95	P11940	95	DPSLRKSGVGN
426	Q13310	108	P11940	108	IKNLDKSIDNK
427	P19984	91	Q95VF7	91	GVITVKTSKAI
428	Q13310	113	P11940	113	KSIDNKALYDT
429	Q13310	129	P11940	129	NILSCKVVCDE
430	P19984	116	Q95VF7	116	ANVVEKLADYL
431	Q13310	167	P11940	167	LLNDRKVFVGR
432	Q13310	174	P11940	174	FVGRFKSRKER
433	Q13310	177	P11940	177	RFKSRKEREAE
434	Q13310	268	P11940	268	VGRAQKKVERQ
435	O60814	12	Q99877	12	SAPAPKKGSKK
436	Q13310	348	P11940	348	PEEATKAVTEM
437	O60814	13	Q99877	13	APAPKKGSKKA
438	O60814	16	Q99877	16	PKKGSKKAVTK
439	O60814	17	Q99877	17	KKGSKKAVTKA
440	O60814	21	Q99877	21	KKAVTKAQKKD
441	Q13310	568	P11940	559	PPQEQKQMLGE
442	O60814	24	Q99877	24	VTKAQKKDGKK
443	O60814	25	Q99877	25	TKAQKKDGKKR
444	Q13310	615	P11940	606	ESLRSKVDEAV
445	O60814	28	Q99877	28	QKKDGKKRKRS
446	O60814	29	Q99877	29	KKDGKKRKRSR
447	O60814	31	Q99877	31	DGKKRKRSRKE
448	O60814	35	Q99877	35	RKRSRKESYSV
449	O60814	44	Q99877	44	SVYVYKVLKQV
450	P39476	9	P13123	9	VKFKYKGEEKE
451	O60814	86	Q99877	86	LAHYNKRSTIT
452	O60814	117	Q99877	117	VSEGTKAVTKY
453	Q16778	121	Q99877	121	TKAVTKYTSSK
454	Q16778	126	Q99877	126	KYTSSK-----
455	P39476	49	P13123	48	GAVSEKDAPKE
456	O60814	12	Q99880	12	SAPAPKKGSKK

457	O60814	13	Q99880	13	APAPKKGSKKA
458	O60814	16	Q99880	16	PKKGSKKAVTK
459	O60814	17	Q99880	17	KKGSKKAVTKA
460	O60814	21	Q99880	21	KKAVTKAQKKD
461	O60814	24	Q99880	24	VTKAQKKDGKK
462	O60814	25	Q99880	25	TKAQKKDGKKR
463	O60814	28	Q99880	28	QKKDGKKRKRS
464	O60814	29	Q99880	29	KKDGKKRKRSR
465	O60814	31	Q99880	31	DGKKRKRSRKE
466	O60814	35	Q99880	35	RKRSRKESYSV
467	O60814	44	Q99880	44	SVYVYKVLKQV
468	O60814	86	Q99880	86	LAHYNKRSTIT
469	O60814	117	Q99880	117	VSEGTKAVTKY
470	Q16778	121	Q99880	121	TKAVTKYTSSK
471	Q16778	126	Q99880	126	KYTSSK-----
472	P40283	36	Q9FFC0	31	AEKKPKAGKKL
473	P40283	39	Q9FFC0	34	KPKAGKKLPKE
474	P40283	69	Q9FFC0	64	KIYIFKVLKQV
475	P40283	72	Q9FFC0	67	IFKVLKQVHPD
476	P40283	83	Q9FFC0	78	IGISSKAMGIM
477	P40283	111	Q9FFC0	106	LARYNKKPTIT
478	P40283	112	Q9FFC0	107	ARYNKKPTITS
479	P40283	142	Q9FFC0	137	VSEGTKAVTKF
480	P40283	146	Q9FFC0	141	TKAVTKFTSS-
481	P40283	7	Q9LZT0	7	APKAEKKPAEK
482	P40283	8	Q9LZT0	8	PKAEKKPAEKK
483	P40283	23	Q9LZT0	18	KPVEEKSKAEK
484	P40283	25	Q9LZT0	20	VEEKSKAEKAP
485	P40283	28	Q9LZT0	23	KSKAEKAPAEK
486	P40283	33	Q9LZT0	28	KAPAEKKPKAG
487	P40283	34	Q9LZT0	29	APAEKKPKAGK
488	P40283	36	Q9LZT0	31	AEKKPKAGKKL
489	P40283	39	Q9LZT0	34	KPKAGKKLPKE
490	P40283	40	Q9LZT0	35	PKAGKKLPKEA
491	P40283	43	Q9LZT0	38	GKKLPKEAGAG
492	P40283	51	Q9LZT0	46	GAGGDKKKKMK
493	P40283	52	Q9LZT0	47	AGGDKKKKMKK
494	P40283	53	Q9LZT0	48	GGDKKKKMKKK
495	P40283	54	Q9LZT0	49	GDKKKKMKKKS
496	P40283	56	Q9LZT0	51	KKKKMKKKSVE
497	P40283	57	Q9LZT0	52	KKKMKKKSVET
498	P40283	58	Q9LZT0	53	KKMKKKSVETY
499	P40283	64	Q9LZT0	59	SVETYKIYIFK
500	P40283	69	Q9LZT0	64	KIYIFKVLKQV
501	P40283	72	Q9LZT0	67	IFKVLKQVHPD
502	P40283	83	Q9LZT0	78	IGISSKAMGIM
503	P40283	111	Q9LZT0	106	LARYNKKPTIT
504	P40283	112	Q9LZT0	107	ARYNKKPTITS

505	P40283	142	Q9LZT0	137	VSEGTKAVTKF
506	P40283	146	Q9LZT0	141	TKAVTKFTSS-

(**C**) Of the 48 samples in their testing positive dataset, the following 24 pairs contain a duplicate sequence

Pair #	Protein	Site	Protein	Site	Duplicate sequence
1	P00052	80	P62772	80	YLLNPKKYIPG
2	P59169	10	P09988	10	KQTARKSTGGK
3	P00052	94	P62772	94	VFPGLKKPQDR
4	P58876	47	P62807	47	VYKVLKQVHPD
5	P58876	58	P62807	58	TGISSKAMGIM
6	P62154	116	P11121	116	TNLGEKLTDEE
7	P58876	109	P62807	109	PGELAKHAVSE
8	P59169	5	Q6LCW8	5	-MARTKQTARK
9	P59169	10	Q6LCW8	10	KQTARKSTGGK
10	P09988	37	Q6LCW8	36	ATGGVKKPHRY
11	P58876	47	P23527	47	VYKVLKQVHPD
12	P58876	58	P23527	58	TGISSKAMGIM
13	P58876	109	P23527	109	PGELAKHAVSE
14	P58876	47	Q8N257	47	VYKVLKQVHPD
15	P58876	58	Q8N257	58	TGISSKAMGIM
16	P58876	109	Q8N257	109	PGELAKHAVSE
17	P59169	5	P50564	5	-MARTKQTARK
18	P59169	10	P50564	10	KQTARKSTGGK
19	P58876	47	Q93079	47	VYKVLKQVHPD
20	P58876	58	Q93079	58	TGISSKAMGIM
21	Q6LCW8	28	P50564	28	TKAARKTPATG
22	P58876	109	Q93079	109	PGELAKHAVSE
23	P09988	37	P50564	36	ATGGVKKPHRY
24	P62154	116	Q95NR9	116	TNLGEKLTDEE

(**D**) Of the 243 samples in their testing negative dataset, the following 111 pairs contain a duplicate sequence

Pair #	Protein	Site	Protein	Site	Duplicate sequence
1	P00052	19	P62772	19	GEKIFKTKCAQ
2	P00052	21	P62772	21	KIFKTKCAQCH
3	P00052	30	P62772	30	CHTVDKGAGHK
4	P62154	31	P07463	31	GTITTKELGTV
5	P00052	35	P62772	35	KGAGHKQGPNL
6	P00052	81	P62772	81	LLNPKKYIPGT
7	P00052	87	P62772	87	YIPGTKMVFPG
8	P59169	15	P09988	15	KSTGGKAPRKQ

9	P00052	95	P62772	95	FPGLKKPQDRA
10	P59169	38	P09988	38	TGGVKKPHRYR
11	P59169	65	P09988	65	ELLIRKLPFQR
12	P58876	21	P62807	21	KKAVTKAQKKD
13	P59169	80	P09988	80	IAQDFKTDLRF
14	P58876	24	P62807	24	VTKAQKKDGKK
15	P58876	25	P62807	25	TKAQKKDGKKR
16	P58876	28	P62807	28	QKKDGKKRKRS
17	P62154	14	P11121	14	QIAEFKEAFSL
18	P58876	29	P62807	29	KKDGKKRKRSR
19	P62154	22	P11121	22	FSLFDKDGDGT
20	P58876	31	P62807	31	DGKKRKRSRKE
21	P62154	31	P11121	31	GTITTKELGTV
22	P58876	35	P62807	35	RKRSRKESYSV
23	P62154	76	P11121	76	TMMARKMKDTD
24	P58876	44	P62807	44	SVYVYKVLKQV
25	P62154	78	P11121	78	MARKMKDTDSE
26	P62154	95	P11121	95	FRVFDKDGNGF
27	P58876	86	P62807	86	LAHYNKRSTIT
28	P62154	149	P11121	149	TMMTSK-----
29	P58876	117	P62807	117	VSEGTKAVTKY
30	P58876	121	P62807	121	TKAVTKYTSSK
31	P58876	126	P62807	126	KYTSSK-----
32	P62807	12	P23527	12	SAPAPKKGSKK
33	P59169	15	Q6LCW8	15	KSTGGKAPRKQ
34	P62807	13	P23527	13	APAPKKGSKKA
35	P62807	16	P23527	16	PKKGSKKAVTK
36	P62807	17	P23527	17	KKGSKKAVTKA
37	P58876	21	P23527	21	KKAVTKAQKKD
38	P58876	24	P23527	24	VTKAQKKDGKK
39	P58876	25	P23527	25	TKAQKKDGKKR
40	P59169	54	Q6LCW8	53	LREIRKYQKST
41	P58876	28	P23527	28	QKKDGKKRKRS
42	P59169	57	Q6LCW8	56	IRKYQKSTELL
43	P58876	29	P23527	29	KKDGKKRKRSR
44	P59169	65	Q6LCW8	64	ELLIRKLPFQR
45	P58876	31	P23527	31	DGKKRKRSRKE
46	P59169	80	Q6LCW8	79	IAQDFKTDLRF
47	P59169	116	Q6LCW8	115	CAIHAKRVTIM
48	P59169	123	Q6LCW8	122	VTIMPKDIQLA
49	P62807	12	Q8N257	12	SAPAPKKGSKK
50	P58876	86	P23527	86	LAHYNKRSTIT
51	P62807	13	Q8N257	13	APAPKKGSKKA
52	P62807	16	Q8N257	16	PKKGSKKAVTK
53	P58876	117	P23527	117	VSEGTKAVTKY
54	P62807	17	Q8N257	17	KKGSKKAVTKA
55	P58876	121	P23527	121	TKAVTKYTSSK
56	P58876	21	Q8N257	21	KKAVTKAQKKD

57	P58876	126	P23527	126	KYTSSK-----
58	P58876	24	Q8N257	24	VTKAQKKDGKK
59	P58876	25	Q8N257	25	TKAQKKDGKKR
60	P04717	18	P27064	18	FKAGVKDYKLT
61	P04717	21	P27064	21	GVKDYKLTYYT
62	P23527	44	Q8N257	44	SIYVYKVLKQV
63	P04717	128	P27064	128	NVFGFKALRAL
64	P04717	161	P27064	161	QVERDKLNKYG
65	P58876	86	Q8N257	86	LAHYNKRSTIT
66	P04717	164	P27064	164	RDKLNKYGRPL
67	P04717	175	P27064	175	LGCTIKPKLGL
68	P58876	117	Q8N257	117	VSEGTKAVTKY
69	P04717	177	P27064	177	CTIKPKLGLSA
70	P58876	121	Q8N257	121	TKAVTKYTSSK
71	P04717	183	P27064	183	LGLSAKNYGRA
72	P58876	126	Q8N257	126	KYTSSK-----
73	P04717	201	P27064	201	GLDFTKDDENV
74	P23527	6	Q93079	6	MPDPAKSAPAP
75	P62807	12	Q93079	12	SAPAPKKGSKK
76	P04717	236	P27064	236	ETGEIKGHYLN
77	P62807	13	Q93079	13	APAPKKGSKKA
78	P62807	16	Q93079	16	PKKGSKKAVTK
79	P04717	305	P27064	305	VIDRQKNHGMH
80	P62807	17	Q93079	17	KKGSKKAVTKA
81	P58876	21	Q93079	21	KKAVTKAQKKD
82	P04717	334	P27064	334	GTVVGKLEGER
83	P58876	24	Q93079	24	VTKAQKKDGKK
84	P58876	25	Q93079	25	TKAQKKDGKKR
85	P58876	28	Q93079	28	QKKDGKKRKRS
86	P04717	463	P27064	463	ACEVWKEIKFE
87	P58876	29	Q93079	29	KKDGKKRKRSR
88	P58876	31	Q93079	31	DGKKRKRSRKE
89	P58876	35	Q93079	35	RKRSRKESYSV
90	P58876	44	Q93079	44	SVYVYKVLKQV
91	P59169	15	P50564	15	KSTGGKAPRKQ
92	Q6LCW8	24	P50564	24	KQLATKAARKT
93	P58876	86	Q93079	86	LAHYNKRSTIT
94	P58876	117	Q93079	117	VSEGTKAVTKY
95	P58876	121	Q93079	121	TKAVTKYTSSK
96	P59169	54	P50564	53	LREIRKYQKST
97	P58876	126	Q93079	126	KYTSSK-----
98	P59169	57	P50564	56	IRKYQKSTELL
99	P62154	14	Q95NR9	14	QIAEFKEAFSL
100	P59169	65	P50564	64	ELLIRKLPFQR
101	P62154	22	Q95NR9	22	FSLFDKDGDGT
102	P59169	80	P50564	79	IAQDFKTDLRF
103	P62154	31	Q95NR9	31	GTITTKELGTV
104	P62154	76	Q95NR9	76	TMMARKMKDTD

105	P59169	123	P50564	122	VTIMPKDIQLA
106	P62154	78	Q95NR9	78	MARKMKDTDSE
107	P62154	95	Q95NR9	95	FRVFDKDGNGF
108	P62807	12	P58876	12	SAPAPKKGSKK
109	P62807	13	P58876	13	APAPKKGSKKA
110	P62807	16	P58876	16	PKKGSKKAVTK
111	P62807	17	P58876	17	KKGSKKAVTKA
