# Supplementary material for: iMethyl-PseAAC: Identification of Protein Methylation Sites via a Pseudo Amino Acid Composition Approach
Source: Biomed Res Int. 2014 May 22;2014:947416. doi: 10.1155/2014/947416 (PMC4054830; doi:10.1155/2014/947416)
Supplement: Supplementary file 1 — S1: An analysis of the benchmark dataset used in Hu et al. (Biopolymers, 2011, 95, 763-771) S2: List of self-conflict samples in the benchmark dataset used in Hu et al. (Biopolymers, 2011, 95, 763-771) S3: Benchmark dataset used for studying the Arg-methylation. It contains 1,481 samples, of which 185 are positive and 1,296 negative. These data were extracted from UniProtKB/Swiss-Prot database (version UniProt release 2013_06). S4: Benchmark dataset used for studying the Lys-methylation. It contains 1,884 samples, of which 226 are positive and 1,518 negative. These data were extracted from UniProtKB/Swiss-Prot database (version UniProt release 2013_06). S5: Seven negative subsets for studying Arg-methylation. Each subset contains 185 negative samples randomly taken from the 1,296 negative samples in Online Supporting Information S3 except for the 6th subset, which contains 186 samples. None of the samples in one subset occurs in any other subset. S6: Seven negative subsets for studying Lys-methylation. Each subset contains 217 negative samples randomly taken from the 1,518 negative samples in Online Supporting Information S4 except for the 5th subset, which only contains 216 samples. None of the samples in one subset occurs in any other subset. S7: Independent dataset for studying the Arg-methylation. It contains 75 samples, of which 20 are positive and 55 negative. None of the samples listed here occurs in Online Supporting Information S3. S8: Independent dataset for studying the Lys-methylation. It contains 40 samples, of which 14 are positive and 26 negative. None of the samples listed here occurs in Online Supporting Information S4. S9: The code for encoding the peptides investigated in this paper. [file 947416.f1.zip › S2.pdf]

Online Supporting Information S2. List of self-conflict samples in the benchmark dataset used in Hu et al. (Biopolymers, 2011, 95, 763-771)

I. Arg-methylation dataset

(**A**) Of the $(180+2171)=2351$ samples in their learning dataset for Arg-methylation, the following 8 samples occur in both positive and negative subsets

Pair #	Positive		Negative		Conflict sample
	Protein	Site	Protein	Site	
1	Q92804	483	Q92804	498	GYGGDRGGYGG
2	Q92804	483	Q92804	505	GYGGDRGGYGG
3	Q92804	483	Q92804	512	GYGGDRGGYGG
4	Q92804	483	Q92804	519	GYGGDRGGYGG
5	P22509	28	P35637	248	GRGGGRGGRGG
6	Q01844	596	P35637	491	GRGGDRGGFRG
7	P09651	206	P04256	206	NFGGGRGGGFG
8	P09651	225	P04256	225	GNFSGRGGFGG

(**B**) Of the $(10+206)=216$ samples in their testing dataset for Arg-methylation, no any sample occur in both positive and negative subsets

II. Lys-methylation dataset

(**A**) Of the $(262+2569)=2831$ samples in their learning dataset for Lys-methylation, the following 60 samples occur in both positive and negative subsets

Pair #	Positive		Negative		Conflict sample
	Protein	Site	Protein	Site	
1	P00042	79	P00043	80	LENPKKYIPGT
2	P00042	79	P00046	78	LENPKKYIPGT
3	P00042	79	P00048	78	LENPKKYIPGT
4	P02993	36	P02994	36	CGGIDKRTIEK
5	O60814	109	P40283	134	PGELAKHAVSE
6	P39476	61	P61991	61	LQMLEKQKK--
7	P39476	63	P61991	63	MLEKQKK----
8	P39476	64	P61991	64	LEKQKK-----
9	O15819	5	P07041	5	-MARTKQTARK
10	P61830	19	P07041	19	GKAPRKQLASK
11	P61830	24	P07041	24	KQLASKAARKS
12	P61830	28	P07041	28	SKAARKSAPST
13	P02993	36	P68104	36	CGGIDKRTIEK
14	P61830	37	P07041	37	STGGVKKPHRY

15	P59226	19	P08898	19	GKAPRKQLATK
16	P59226	24	P08898	24	KQLATKAARKS
17	P59226	19	P08903	19	GKAPRKQLATK
18	P59226	24	P08903	24	KQLATKAARKS
19	P59226	37	P08903	37	ATGGVKKPHRF
20	P59226	24	P68429	24	KQLATKAARKS
21	P59226	37	P68429	37	ATGGVKKPHRF
22	O15819	5	P68432	5	-MARTKQTARK
23	P59226	19	P68432	19	GKAPRKQLATK
24	P59226	24	P68432	24	KQLATKAARKS
25	P59226	19	P84229	19	GKAPRKQLATK
26	P59226	24	P84229	24	KQLATKAARKS
27	P59226	19	P84249	19	GKAPRKQLATK
28	P59226	24	P84249	24	KQLATKAARKS
29	O15819	5	Q16695	5	-MARTKQTARK
30	P59226	10	Q16695	10	KQTARKSTGGK
31	P59226	19	Q16695	19	GKAPRKQLATK
32	P59226	19	Q42681	19	GKAPRKQLATK
33	P02993	36	Q5VTE0	36	CGGIDKRTIEK
34	O15819	5	P10651	5	-MARTKQTARK
35	P61830	19	P10651	19	GKAPRKQLASK
36	P02993	36	Q71V39	36	CGGIDKRTIEK
37	O60814	109	Q9FFC0	129	PGELAKHAVSE
38	O60814	109	Q9LZT0	129	PGELAKHAVSE
39	P02994	30	P02993	30	GHLIYKCGGID
40	P49949	101	P00219	101	AAIDVKPP---
41	P61830	15	P59226	15	KSTGGKAPRKQ
42	P61830	80	P59226	80	IAQDFKTDLRF
43	P08898	80	P59226	80	IAQDFKTDLRF
44	P68429	15	P59226	15	KSTGGKAPRKQ
45	P82888	80	P62787	80	EHARRKTVTAM
46	P84229	15	P59226	15	KSTGGKAPRKQ
47	P84229	37	P68432	37	ATGGVKKPHRY
48	P84229	80	P59226	80	IAQDFKTDLRF
49	P84249	15	P59226	15	KSTGGKAPRKQ
50	P84249	38	P68432	38	TGGVKKPHRYR
51	P84249	80	P59226	80	IAQDFKTDLRF
52	Q42681	36	P68432	37	ATGGVKKPHRY
53	Q42681	37	P68432	38	TGGVKKPHRYR
54	Q5VTE0	55	P02993	55	GKGSFKYAWVL
55	P10651	37	P68432	37	ATGGVKKPHRY
56	Q71V39	55	P02993	55	GKGSFKYAWVL
57	Q9SE35	143	Q9SE35	110	RILSSKKSGSY
58	Q9SE35	165	Q9SE35	110	RILSSKKSGSY
59	Q9SE35	184	Q9SE35	110	RILSSKKSGSY
60	Q9SE35	222	Q9SE35	110	RILSSKKSGSY

(**B**) Of the $(48+243)=291$ samples in their testing dataset for Lys-methylation, the following 5 samples occur in both positive and negative subsets

Pair #	Positive		Negative		Conflict sample
	Protein	Site	Protein	Site	
1	P59169	5	P09988	5	-MARTKQTARK
2	P59169	19	Q6LCW8	19	GKAPRKQLATK
3	P59169	19	P50564	19	GKAPRKQLATK
4	Q6LCW8	37	P59169	38	TGGVKKPHRYR
5	P50564	37	P59169	38	TGGVKKPHRYR
