# Supplementary material for: iMethyl-PseAAC: Identification of Protein Methylation Sites via a Pseudo Amino Acid Composition Approach
Source: Biomed Res Int. 2014 May 22;2014:947416. doi: 10.1155/2014/947416 (PMC4054830; doi:10.1155/2014/947416)
Supplement: Supplementary file 1 — S1: An analysis of the benchmark dataset used in Hu et al. (Biopolymers, 2011, 95, 763-771) S2: List of self-conflict samples in the benchmark dataset used in Hu et al. (Biopolymers, 2011, 95, 763-771) S3: Benchmark dataset used for studying the Arg-methylation. It contains 1,481 samples, of which 185 are positive and 1,296 negative. These data were extracted from UniProtKB/Swiss-Prot database (version UniProt release 2013_06). S4: Benchmark dataset used for studying the Lys-methylation. It contains 1,884 samples, of which 226 are positive and 1,518 negative. These data were extracted from UniProtKB/Swiss-Prot database (version UniProt release 2013_06). S5: Seven negative subsets for studying Arg-methylation. Each subset contains 185 negative samples randomly taken from the 1,296 negative samples in Online Supporting Information S3 except for the 6th subset, which contains 186 samples. None of the samples in one subset occurs in any other subset. S6: Seven negative subsets for studying Lys-methylation. Each subset contains 217 negative samples randomly taken from the 1,518 negative samples in Online Supporting Information S4 except for the 5th subset, which only contains 216 samples. None of the samples in one subset occurs in any other subset. S7: Independent dataset for studying the Arg-methylation. It contains 75 samples, of which 20 are positive and 55 negative. None of the samples listed here occurs in Online Supporting Information S3. S8: Independent dataset for studying the Lys-methylation. It contains 40 samples, of which 14 are positive and 26 negative. None of the samples listed here occurs in Online Supporting Information S4. S9: The code for encoding the peptides investigated in this paper. [file 947416.f1.zip › S3.pdf]

Online Supporting Information S3. Benchmark dataset used for studying the Arg-methylation. It contains 1,481 samples, of which 185 are positive and 1,296 negative. These data were extracted from UniProtKB/Swiss-Prot database (version UniProt release 2013_06).

(A) List of 185 positive Arg-methylation sites and sequence

Sample #	Protein ID	Site	Sequences
1	Q9NR22	73	EEMTSRDYYFD
2	Q9UBL3	296	SSGKGRGAKRK
3	Q92934	94	EPSPFRGRSRS
4	Q92934	96	SPFRGRSRSAP
5	Q7Q2B7	478	DAQGARNAVNL
6	P11911	198	YEDISRGLQGT
7	Q09472	2142	QAGVQRAGLPQ
8	P03372	260	IRKDRRGGRML
9	Q01844	300	SGPDNRGRGRG
10	Q01844	302	PDNRGRGRGGF
11	Q01844	304	NRGRGRGGFDR
12	Q01844	309	RGGFDRGGMSR
13	Q01844	314	RGGMSRGGRGG
14	Q01844	317	MSRGGRGGGRG
15	Q01844	321	GRGGGRGGMGS
16	Q01844	455	PMNSMRGGLPP
17	Q01844	464	PPREGRGMPPP
18	Q01844	471	MPPPLRGGPGG
19	Q01844	490	GRMGGRGGDRG
20	Q01844	494	GRGGDRGGFPP
21	Q01844	500	GGFPPRGPRGS
22	Q01844	503	PPRGPRGSRGN
23	Q01844	506	GPRGSRGNPSG
24	Q01844	563	PPGGDRGRGGP
25	Q01844	565	GGDRGRGGPGG
26	Q01844	572	GPGGMRGGRGG
27	Q01844	575	GMRGGRGGLMD
28	Q01844	581	GGLMDRGGPGG
29	Q01844	589	PGGMFRGGRGG
30	Q01844	592	MFRGGRGGDRG
31	Q01844	596	GRGGDRGGFRG
32	Q01844	600	DRGGFRGGRGM
33	Q01844	603	GFRGGRGMDRG
34	Q01844	607	GRGMDRGGFGG
35	Q01844	615	FGGGRRGGPGG
36	Q01844	633	QMGGRRGGRGG
37	Q01844	636	GRRGGRGGPGK
38	P22509	8	PGFSPRGGGFG
39	P22509	15	GGFGGRGGFGD
40	P22509	21	GGFGDRGGRGG
41	P22509	24	GDRGGRGGGRG
42	P22509	28	GRGGGRGGRGG

43	P22509	31	GGRGGRGGFGG
44	P35637	242	GGYEPRGRGGG
45	P35637	244	YEPRGRGGGRG
46	P35637	251	GGRGGRGGMGG
47	P35637	259	MGGSDRGGFNK
48	P35637	377	RADFNRGGGNG
49	P35637	383	GGGNGRGGRGR
50	P35637	386	NGRGGRGRGGP
51	P35637	388	RGGRGRGGPMG
52	P35637	394	GGPMGRGGYGG
53	P35637	407	SGGGGRGGFPS
54	P35637	473	YGDDRRGGRGG
55	P35637	476	DRRGGRGGYDR
56	P35637	481	RGGYDRGGYRG
57	P35637	485	DRGGYRGRGGD
58	P35637	487	GGYRGRGGDRG
59	P35637	495	DRGGFRGGRGG
60	P35637	498	GFRGGRGGGDR
61	P35637	503	RGGGDRGGFGP
62	P03347	387	NFRNQRKMVKC
63	P03347	409	NCRAPRKKGCW
64	P68431	3	MMMMARTKQTA
65	P68431	18	GGKAPRKQLAT
66	P62805	4	MMMSGRGKGGK
67	P17096	26	TEKRGRGRPRK
68	P17096	58	TPKRPRGRPKG
69	P17096	60	KRPRGRPKGSK
70	P31942	121	RPIGGRGGYYG
71	O60506	444	MPPPTRGRGRG
72	O60506	510	GAAPSRGRGAA
73	O60506	518	GAAPPRGRAGY
74	O60506	536	SARGVRGARGG
75	O60506	539	GVRGARGGAQQ
76	Q07666	45	LPHRSRGGGGG
77	Q07666	52	GGGGSRGGARA
78	Q07666	304	PVPRGRGVGPP
79	P08199	656	GGFGGRGGGRG
80	P08199	660	GRGGGRGGFGG
81	P08199	670	GRGGGRGGGRG
82	P08199	680	GGFGGRGRGGF
83	P08199	682	FGGRGRGGFGG
84	P08199	688	GGFGGRGGFRG
85	P08199	692	GRGGFRGGRGG
86	P08199	695	GFRGGRGGGGG
87	Q28165	238	DESLFRGRQIK
88	Q28165	259	ISTTDRGFPRA
89	Q28165	263	DRGFPRARYRA
90	Q28165	265	GFPRARYRART
91	Q28165	267	PRARYRARTTN
92	Q28165	269	ARYRARTTNYN
93	Q28165	277	NYNSSRSRFYS
94	Q28165	279	NSSRSRFYSGF

95	Q28165	287	SGFNSRPRGRV
96	Q28165	289	FNSRPRGRVYR
97	Q28165	291	SRPRGRVYRGR
98	Q28165	294	RGRVYRGRARA
99	Q28165	296	RVYRGRARATS
100	Q28165	298	YRGRARATSWY
101	O75925	303	RAKGIRNPDHS
102	Q96PU8	242	PPAALRTPTPA
103	Q92804	206	SSGGDRGGFKN
104	Q92804	528	GGDRSRGGYGG
105	Q92804	535	GYGGDRGGGSG
106	Q92804	570	GYGGDRGGYGG
107	P09867	194	ASSSQRGRSGS
108	O00267	681	SAGGQRGGFGS
109	O00267	696	SGGMSRGRGRR
110	O00267	698	GMSRGRGRRDN
111	P69697	52	YGRKKRRQRRR
112	P69697	53	GRKKRRQRRRP
113	Q15554	17	GRAAGRRASRS
114	Q9NR22	58	PSCPGRGKMSK
115	P15056	671	IFMVGRGYLSP
116	Q5TAQ9	204	LHFNQRGTWLA
117	Q96EY1	58	TSCGPRALLTL
118	Q96EY1	238	MDTCERCNGKG
119	Q96EY1	293	PCVVCRGAGQA
120	P06746	83	ATGKLRKLEKI
121	P06746	152	EKRIPREEMLQ
122	Q92997	27	PLPAERVTLAD
123	Q92997	212	QSSASRLMRRH
124	Q92997	342	WDPSPRGCFTL
125	Q92997	698	ELTASRQSFRM
126	Q60487	4	VVVGGRGRGRG
127	Q60487	6	VGGRGRGRGTA
128	Q60487	8	GRGRGRGTAAA
129	Q54G21	314	ANLLQRVAKLG
130	Q9R1E0	248	SGKSPRRRAAS
131	Q9R1E0	250	KSPRRRAASMD
132	Q13283	435	GPGGPRGGLGG
133	P31943	233	FERMRRGAYGG
134	P31942	287	MGGYGRDGMDN
135	O60506	496	FQVGARGRGGR
136	P04264	82	SISVARGGGRG
137	P08729	20	AAFSGRGAQVR
138	Q07666	310	GVGPPRGALVR
139	Q07666	315	RGALVRGTPVR
140	Q07666	320	RGTPVRGAITR
141	Q07666	325	RGAITRGATVT
142	Q07666	340	PPPTVRGAPAP
143	Q36736	45	LSPEMREHYEK
144	P02687	106	SQGKGRGLSLS
145	P15720	106	MQAKGRGLSLT
146	P25274	105	SQGKGRGTVLS

147	Q8IZS5	113	ATEVSREDDDR
148	P11940	493	QTMGPRPAAAA
149	Q9JMB7	14	ARGRARGQETV
150	Q9JMB7	49	GDLVGRGRQRG
151	Q9JMB7	371	QPKRRRGPGGT
152	Q8CDG1	74	LVSMFRGMGLD
153	Q8CDG1	95	VPPLGRGVLGR
154	Q86WR7	252	QPKAPRFPSNI
155	Q86WR7	320	RVARGRGLPGP
156	P09651	206	NFGGGRGGGFG
157	P09651	225	GNFSGRGGFGG
158	P22626	203	SSRSGRGGNFG
159	P22626	213	GFGDSRGGGGN
160	P51991	52	TDDSLREHFEK
161	Q99729	245	YGSGGRGNRNR
162	P14678	147	MTPQGRGTVAA
163	Q14524	513	HLSLTRGLSRT
164	Q14524	526	KPRSSRGSIFT
165	Q14524	680	ELEESRHKCPP
166	P23246	681	GGQGPRGMGPG
167	P68363	339	TIKTKRSIQFV
168	P07437	318	VAAVFRGRMSM
169	Q15554	18	RAAGRRASRSS
170	Q86V81	220	TRRGTRGGARG
171	O08583	218	TRRGTRGGSRG
172	P39748	19	APSAIRENDIK
173	P39748	100	GELAKRSERRA
174	P39748	104	KRSERRAEAEK
175	P39748	192	SPVLMRHLTAS
176	P68433	9	TKQTARKSTGG
177	P31269	140	EPLSARRGDCP
178	Q8CDG1	100	RGVLGRGLSAN
179	Q8CDG1	163	ASSIGRGMDKP
180	Q8CDG1	549	SNELTRWGLSL
181	P04049	563	IFMVGRGYASP
182	P46783	158	RGGFGRGRGQP
183	P46783	160	GFGRGRGQPPQ
184	O76922	13	VIARGRGRGRK
185	O76922	15	ARGRGRGRKPN

(B) List of 1,296 negative Arg-methylation sites and sequence

Seqence #	Protein ID	Site	Sequences
1	Q9NR22	229	VAIEDRQYKDF
2	Q9UBL3	324	LFSAQRLPPHG
3	Q92934	72	VEIRSRHSSYP
4	Q7Q2B7	222	IDLPERVDVII
5	P11911	9	GLEALRALPLL
6	P11911	118	CGTYLRVRNPV

7	Q09472	86	LSELLRSGSSP
8	Q09472	354	HKCQRREQANG
9	Q09472	408	WKNCTRHDCPV
10	Q09472	580	ITQDLRNHLVH
11	Q09472	604	ALKDRRMENLV
12	Q09472	649	LEEKRRTRLQK
13	Q09472	1015	TETEERSTELK
14	Q09472	1076	ESLPFRQPVDP
15	Q09472	1324	GEVTVRVVHAS
16	Q09472	1410	RPKCLRTAVYH
17	Q09472	1462	IPKPKRLQEWY
18	P03372	28	LEPLNRPQLKI
19	P03372	158	NSDNRRQGGRE
20	P03372	234	IDKNRRKSCQA
21	P03372	256	MKGGIRKDRRG
22	P03372	503	QQQHQRLAQLL
23	P03372	515	ILSHIRHMSNK
24	Q01844	614	GFGGGRRGGPG
25	Q01844	632	EQMGGRRGGRG
26	P22509	76	GGGRGRGGGRG
27	P22509	224	VIEDARHPHKY
28	P35637	213	YGQQDRGGRGR
29	P35637	269	KFGGPRDQGSR
30	P35637	274	RDQGSRHDSEQ
31	P35637	328	NLYTDRETGKL
32	P03347	299	RDYVDRFYKTL
33	P03347	305	FYKTLRAEQAS
34	P03347	429	KDCTERQANFL
35	P03347	452	NFLQSRPEPTA
36	P03347	502	PLTSLRSLFGN
37	P68431	54	LREIRRYQKST
38	P68431	73	FQRLVREIAQD
39	P68431	117	AIHAKRVTIMP
40	P68431	130	IQLARRIRGER
41	P62805	24	HRKVLRDNIQG
42	P62805	68	LENVIRDAVTY
43	P62805	96	LKRQGRTLYGF
44	P17096	84	PGRKPRGRPKK
45	P31942	19	SDGTVRLRGLP
46	P31942	21	GTVRLRGLPFG
47	P31942	105	YDPPRRLLGQR
48	P31942	222	PLNPIRVHIDI
49	O60506	303	TAAQARRRLMS
50	O60506	305	AQARRRLMSGK
51	O60506	446	PPTRGRGRGGR
52	Q07666	13	AARMSRSSGRS
53	Q07666	43	PPLPHRSRGGG
54	Q07666	291	VPVRGRGAAPP
55	Q07666	331	GATVTRGVPPP
56	P08199	394	KVRAARTLLAK
57	P08199	604	RIVTDRETGSS
58	O75925	63	KELYRRRFPQK

59	O75925	257	ITSLVRLSTTV
60	O75925	579	LLHSSRFFPYT
61	Q92804	101	GSQGGRAPSYD
62	Q92804	164	HTQDDRRDVSR
63	Q92804	165	TQDDRRDVSRY
64	Q92804	185	SQGGGRGRGGY
65	Q92804	321	SFATRRPEFMR
66	Q92804	372	NMNFARRNSCN
67	Q92804	415	GRGGDRGGYGG
68	Q92804	431	GYGGDRSSGGG
69	Q92804	459	GYGGDRGGGYG
70	Q92804	580	GKMGGRNDYRN
71	Q92804	584	GRNDYRNDQRN
72	P09867	82	HKVDGRVVEPK
73	P09867	88	VVEPKRAVSRE
74	O00267	17	EEDSERSSDGE
75	O00267	30	EVDEERRSAAG
76	O00267	130	LQNLWRDQREE
77	O00267	368	GNRYSRKGFLF
78	O00267	571	KVVTVRHQAVT
79	O00267	641	FVCKTRHLVLA
80	O00267	652	GGSKPRDVTNF
81	O00267	783	YGSGSRTPMYG
82	P69697	7	EPVDPRLEPWK
83	P69697	56	KRRQRRRPPQG
84	Q15554	28	SGRARRGRHEP
85	Q15554	30	RARRGRHEPGL
86	Q15554	47	GAGEARLEEAV
87	Q15554	69	ALRAFRGSRYG
88	Q15554	72	AFRGSRYGDFR
89	Q15554	192	TTQKLRNDLLN
90	Q15554	356	QPKNKRMTISR
91	P15056	188	KALMMRGLIPE
92	P15056	260	LFQGFRCQTCG
93	P15056	347	IPQPFRPADED
94	P15056	360	NQFGQRDRSSS
95	P15056	426	GPQRERKSSSS
96	Q5TAQ9	161	PALRERELGSS
97	Q96EY1	187	PEELFRKIFGE
98	Q96EY1	272	GPFVMRSTCRR
99	Q96EY1	276	MRSTCRRCGGR
100	Q96EY1	281	RRCGGRGSIII
101	Q96EY1	303	AKQKKRVMIPV
102	Q96EY1	364	LGGTARAQGLY
103	P06746	89	KLEKIRQDDTS
104	P06746	126	TLEDLRKNEDK
105	Q92997	215	ASRLMRRHKRR
106	Q92997	220	RRHKRRRRKQK
107	Q54G21	660	KMMESRNEAIT
108	Q54G21	708	INQLKRDYNTL
109	Q54G21	827	TLETERSALSS
110	Q54G21	841	LVENTRLETES

111	Q9R1E0	19	FEPLPRQRSCT
112	Q9R1E0	313	STFRPRTSSNA
113	Q9R1E0	551	TSAMNRLTPVK
114	Q9R1E0	586	CNGYGRMGVLH
115	Q13283	106	NNQALRRFMQT
116	Q13283	107	NQALRRFMQTF
117	Q13283	283	PASQPRPESKP
118	P31943	230	GAGFERMRRGA
119	P04264	30	INYQRRTTSSS
120	P04264	65	GGFGSRSLVNL
121	P04264	199	FIDKVRFLEQQ
122	P04264	343	SMDNNRSLDLD
123	P08729	48	GASRPRVAVRS
124	P08729	305	DLRNTRNEISE
125	P15720	10	KRSSFRHGSKM
126	P15720	48	FFGGDRHVPRR
127	P15720	96	NIVSPRTPPPM
128	P15720	162	GGSGSRPGSRS
129	P25274	128	FGYGGRAADYK
130	Q8IZS5	61	EKKVIRHDMPD
131	Q8IZS5	67	HDMPDRTLAAH
132	Q8IZS5	77	HQQKFRLPASA
133	P11940	153	QEAAERAIEKM
134	P11940	166	MLLNDRKVFVG
135	P11940	186	AELGARAKEFT
136	P11940	287	QMKQDRITRYQ
137	P11940	331	MMEGGRSKGFG
138	Q9JMB7	6	MTGRARARARG
139	Q9JMB7	36	GYIPPRPQQSP
140	Q9JMB7	51	LVGRGRQRGMV
141	Q9JMB7	84	ERGGRRRDFHD
142	Q9JMB7	147	EARRLRSALLF
143	Q8CDG1	177	FGLTARDPPRL
144	Q86WR7	140	AGKEHRKQDAE
145	Q86WR7	204	QKISERMAGNE
146	Q86WR7	221	PFREGRPGEWR
147	P09651	265	YSGGSRGYGSG
148	P22626	185	HNAEVRKALSR
149	P51991	239	GGNFGRGGNFG
150	Q99729	30	EASRGRGWTGA
151	P14678	228	PPPGMRGPPPP
152	Q14524	179	VKILARGFCLH
153	Q14524	340	CPEGYRCLKAG
154	Q14524	367	FLALFRLMTQD
155	P23246	399	FGPIERAVVIV
156	P68363	105	ANNYARGHYTI
157	P68363	264	LVPYPRIHFPL
158	P68363	320	CCLLYRGDVVP
159	P07437	77	TMDSVRSGPFG
160	P07437	162	EEYPDRIMNTF
161	P07437	282	GSQQYRALTVP
162	P07437	359	CDIPPRGLKMA

163	Q86V81	20	LNRSQRGGRGG
164	Q86V81	174	VPLDGRPMNIQ
165	Q86V81	197	AQSVNRGGMTR
166	Q86V81	202	RGGMTRNRGAG
167	O08583	56	GGGPMRNRPAI
168	O08583	203	GMTRNRGSGGF
169	P39748	70	MGMFYRTIRMM
170	P31269	207	HARSTRKKRCP
171	P31269	233	NMYLTRDRRYE
172	P31269	241	RYEVARLLNLT
173	P04049	100	CCAVFRLLHEH
174	P04049	143	THNFARKTFLK
175	P04049	231	VSSQHRYSTPH
176	P04049	282	IEDAIRSHSES
177	P04049	318	PAQRERAPVSG
178	P04049	333	NKIRPRGQRDS
179	P46783	8	MPKKNRIAIYE
180	P46783	80	GIQYLRDYLHL
181	P46783	96	PATLRRSRPET
182	P46783	105	ETGRPRPKGLE
183	P46783	116	GERPARLTRGE
184	P46783	128	DRDTYRRSAVP
185	O76922	87	SVRGRRLITDL
186	Q9NR22	13	RCLLLRRKMAE
187	Q9NR22	14	CLLLRRKMAEN
188	Q9UBL3	211	ECMTTRQRPGK
189	Q9UBL3	231	TMSKERDVFLV
190	Q9UBL3	315	TTKKARSDPLF
191	Q9UBL3	374	PGDLYRACLYE
192	Q9UBL3	411	GYSMVRASHGV
193	Q9UBL3	592	KDLTYRPMSDM
194	Q92934	116	GRELRRMSDEF
195	Q92934	131	KKGLPRPKSAG
196	Q7Q2B7	84	ELVLFRFASRG
197	Q7Q2B7	88	FRFASRGDCQQ
198	Q7Q2B7	95	DCQQFRSLLHK
199	Q7Q2B7	341	EKDLHRIQIDV
200	Q7Q2B7	447	KNPYFRYTGAP
201	P11911	47	LGEEARLTCEN
202	P11911	96	VNKNHRGLYWC
203	P11911	137	EGTKNRIITAE
204	Q09472	1104	LSTIKRKLDTG
205	Q09472	1133	AWLYNRKTSRV
206	Q09472	1197	YSYQNRYHFCE
207	Q09472	1392	PPNQRRVYISY
208	Q09472	1494	QATEDRLTSAK
209	P03372	37	KIPLERPLGEV
210	P03372	142	SGYTVREAGPP
211	P03372	241	SCQACRLRKCY
212	P03372	436	TSSRFRMMNLQ
213	Q01844	392	VKMNKRTGQPM
214	P22509	56	GGGGFRGRGGG

215	P22509	84	GRGGKRGNQSG
216	P35637	521	EHRQDRRERPY
217	P03347	275	LNKIVRMYSPT
218	P03347	403	EGHTARNCRAP
219	P68431	129	DIQLARRIRGE
220	P62805	40	IRRLARRGGVK
221	P62805	79	TEHAKRKTVTA
222	P31942	93	IFRSSRSEIKG
223	P31942	129	YYGAGRGSMYD
224	P31942	261	NNMQHRYIELF
225	P31942	301	YGSVGRMGMGN
226	P31942	323	LGGYGRGGGGS
227	O60506	103	VMKTYRQREKQ
228	O60506	229	NNHEIRSGKHI
229	O60506	451	RGRGGRGGYGY
230	O60506	504	GGRGARGAAPS
231	O60506	557	GARGGRGGNVG
232	O60506	565	NVGGKRKADGY
233	Q07666	10	DDPAARMSRSS
234	Q07666	204	GKGSMRDKAKE
235	Q07666	436	VKGAYREHPYG
236	P08199	343	AVVDVRTGTNR
237	P08199	421	DALEIRLVSQD
238	P08199	474	GQRQERTGKNS
239	Q28165	17	GAAGGRGSGPG
240	Q28165	25	GPGRRRHLVPG
241	Q28165	78	EPPRPRAPPGA
242	Q28165	200	CGSVNRVTILC
243	Q28165	248	KVIPKRTNRPG
244	O75925	276	TAEIGRNYSMA
245	O75925	528	LIQDYRHPFHM
246	Q96PU8	106	RILGPRGLTAK
247	Q92804	221	RDYGPRTDADS
248	Q92804	320	VSFATRRPEFM
249	Q92804	341	GRGGYRGRGGF
250	Q92804	349	GGFQGRGGDPK
251	Q92804	388	RPEDSRPSGGD
252	Q92804	406	GERGYRGRGGR
253	Q92804	422	GYGGDRSGGGY
254	P09867	31	TDESLRSHFEQ
255	P09867	196	SSQRGRSGSGN
256	P09867	300	YFAKPRNQGGY
257	O00267	31	VDEERRSAAGS
258	O00267	65	EEDDDRPPKKP
259	O00267	319	DRIKARMSLKD
260	O00267	346	DAEKIRSLGGD
261	O00267	516	LKVLPRDLQLC
262	O00267	582	RKKDNRFAVAL
263	O00267	711	IGQTVRISQGP
264	O00267	798	LQDGSRTPHYG
265	O00267	831	PNTPSRAEEEY
266	O00267	1049	ILGEDREATGV

267	O00267	1066	EDGIVRMDLDE
268	P69697	78	PTSQSRGDPTG
269	Q15554	13	SDGSGRAAGRR
270	Q15554	77	RYGDFRQIRDI
271	Q15554	102	VSRLLRVMQCL
272	P15056	239	THNFVRKTFFT
273	Q5TAQ9	11	SSTDGRTDLAN
274	Q5TAQ9	151	ALPRPRWQALP
275	Q5TAQ9	177	EACGARVFVQR
276	Q5TAQ9	182	RVFVQRFRLQH
277	Q96EY1	102	ILGVPRNASQK
278	Q96EY1	332	IFITFRVQKSP
279	P06746	102	INFLTRVSGIG
280	P06746	137	LNHHQRIGLKY
281	P06746	258	RRIDIRLIPKD
282	Q92997	145	SAQRERPRRRD
283	Q92997	167	AKGERRREPGG
284	Q60487	105	MKEDGRLLASK
285	Q54G21	180	VANGDRVSIKY
286	Q54G21	230	GVIGMRKSAKR
287	Q54G21	793	EESLKRLKLDM
288	Q54G21	1425	IDNSPRQTTTE
289	Q9R1E0	249	GKSPRRRAASM
290	Q9R1E0	487	AQPNSRVLGQN
291	Q9R1E0	534	ASVNGRTLPHV
292	Q13283	159	EEPEERQQTPE
293	Q13283	296	QIPPQRPQRDQ
294	P31943	16	FVVKVRGLPWS
295	P31943	52	YTREGRPSGEA
296	P31943	192	SRAEVRTHYDP
297	P31943	217	RPGAGRGYNSI
298	P04264	37	TSSSTRRSGGG
299	P04264	386	QITAGRHGDSV
300	P08729	30	RLSSARPGGLG
301	P08729	46	GLGASRPRVAV
302	P08729	52	PRVAVRSAYGG
303	P08729	64	VGAGIREVTIN
304	P08729	187	DEINHRTAAEN
305	P08729	276	MAKCSRAEAEA
306	P08729	313	ISEMNRAIQRL
307	P08729	317	NRAIQRLQAEI
308	Q36736	14	SAKLDRLDQEF
309	P02687	41	LDSLGRFFGSD
310	P15720	78	RSQHGRPGDDN
311	P15720	166	SRPGSRSGSPV
312	P25274	9	KRPSQRHGSKY
313	P25274	49	FFSSDRGAPKR
314	Q8IZS5	31	MVKEDREATEG
315	Q8IZS5	142	QDESGREETLN
316	Q8IZS5	177	LEEFQRSQKTI
317	P11940	89	IMWSQRDPSLR
318	P11940	206	DMDDERLKDLF

319	P11940	278	QTELKRKFEQM
320	P11940	311	DDERLRKEFSP
321	Q9JMB7	83	AERGGRRRDFH
322	Q9JMB7	145	LMEARRLRSAL
323	Q9JMB7	160	EDLIGRCHAFD
324	Q8CDG1	9	VRPLFRGPTPV
325	Q8CDG1	88	RPPSKREVPPL
326	Q8CDG1	111	MVRKDREEPRS
327	Q8CDG1	156	LLPLGRAASSI
328	Q86WR7	6	MPVTHRKSDAS
329	Q86WR7	22	TSPSCRLRAFS
330	Q86WR7	173	PDPPRRELRAP
331	Q86WR7	176	PRRELRAPSPP
332	Q86WR7	265	TNGAAREPRRT
333	P22626	15	LERKKREKEQF
334	P22626	60	DPASKRSRGFG
335	P22626	228	PGSNFRGGSDG
336	P22626	238	GYGSGRGFGDG
337	P51991	9	KPPPGRPQPDS
338	P51991	113	KRAVSREDSVK
339	P51991	143	EEYNLRDYFEK
340	P51991	226	GNFMGRGGNFG
341	Q99729	248	GGRGNRNRGNR
342	Q99729	250	RGNRNRGNRGS
343	P14678	221	PPPGMRPPPPG
344	P23246	7	SRDRFRSRGGG
345	P23246	18	GGGFHRRGGGG
346	P23246	25	GGGGGRGGLHD
347	P68363	215	YDICRRNLDIE
348	P68363	243	ITASLRFDGAL
349	P68363	308	VKCDPRHGKYM
350	P07437	213	YDICFRTLKLT
351	P07437	241	VTTCLRFPGQL
352	P07437	276	APLTSRGSQQY
353	P07437	306	AACDPRHGRYL
354	P07437	380	QELFKRISEQF
355	Q86V81	63	RPAIARGAAGG
356	Q86V81	79	PAPYSRPKQLP
357	Q86V81	144	YDRSGRSLGTA
358	O08583	189	IDTQRRPAQSI
359	P39748	211	EFHLSRILQEL
360	P39748	239	YCESIRGIGPK
361	P39748	332	RLSKSRQGSTQ
362	P39748	355	LSSAKRKEPEP
363	P31269	128	GLPSSRPYGIK
364	P31269	141	PLSARRGDCPT
365	P04049	59	TSNTIRVFLPN
366	P04049	223	RESVSRMPVSS
367	P46783	55	QSLKSRGYVKE
368	O76922	86	GSVRGRRLITD
369	O76922	123	FKVLKRPNWTI
370	O76922	146	ATRLRRSFLYE

371	Q9NR22	368	NAKNVRDLDFT
372	Q9UBL3	181	LTWQSRTQDEH
373	Q9UBL3	380	ACLYERVLLAL
374	Q9UBL3	417	ASHGVRKGAWY
375	Q9UBL3	437	PDTAARLGWSQ
376	Q9UBL3	622	EVDGRRSPPWE
377	Q92934	112	AQRYGRELRRM
378	Q92934	142	TATQMRQSSSW
379	Q92934	149	SSSWTRVFQSW
380	Q92934	157	QSWWDRNLGRG
381	Q7Q2B7	40	QGMAVRLLQEG
382	Q7Q2B7	67	TTHLSRGHTSH
383	Q7Q2B7	299	NLVALRDAAMK
384	P11911	162	LLFRKRWQNEK
385	Q09472	15	PPSAKRPKLSS
386	Q09472	370	NLPHCRTMKNV
387	Q09472	504	SPQGMRPMSNM
388	Q09472	626	ESANNRAEYYH
389	Q09472	1187	LCTIPRDATYY
390	Q09472	1297	RLPSTRLGTFL
391	Q09472	1342	PGMKARFVDSG
392	P03372	183	SAKETRYCAVC
393	P03372	269	MLKHKRQRDDG
394	Q01844	486	GGPMGRMGGRG
395	P22509	38	GFGGGRGGFGG
396	P22509	58	GGFRGRGGGGG
397	P22509	74	QSGGGRGRGGG
398	P22509	106	GVFICRGKEDA
399	P35637	216	QDRGGRGRGGS
400	P35637	234	GGGYNRSSGGY
401	P35637	372	SFATRRADFNR
402	P35637	518	SRGEHRQDRRE
403	P03347	20	RWEKIRLRPGG
404	P03347	22	EKIRLRPGGKK
405	P68431	41	VKKPHRYRPGT
406	P68431	43	KPHRYRPGTVA
407	P68431	50	GTVALREIRRY
408	P68431	64	TELLIRKLPFQ
409	P68431	84	FKTDLRFQSSA
410	P62805	18	KGGAKRHRKVL
411	P62805	37	KPAIRRLARRG
412	P62805	46	RGGVKRISGLI
413	P17096	24	DGTEKRGRGRP
414	P17096	28	KRGRGRPRKQP
415	P31942	174	RMRDGRGMGGH
416	P31942	205	RGLPFRATEND
417	O60506	381	IHFDERDGAVK
418	O60506	526	AGYSQRGGPGS
419	O60506	554	GVRGARGGRGG
420	O60506	577	QPDSKRRQTNN
421	O60506	578	PDSKRRQTNNQ
422	Q07666	31	AHPSVRQTPSR

423	Q07666	282	VPEPSRGRGVP
424	Q07666	284	EPSRGRGVPVR
425	Q07666	346	GAPAPRARTAG
426	P08199	348	RTGTNRKFGYV
427	P08199	554	EGRTIRLELQG
428	P08199	599	GSVRARIVTDR
429	Q28165	23	GSGPGRRRHLV
430	O75925	248	PKRPSRPINIT
431	O75925	347	LTIPCRALTCS
432	O75925	417	TWAPMRSKKEV
433	O75925	507	ASPVSRTPSLP
434	Q96PU8	53	EISRVRKDMYN
435	Q96PU8	139	KEEQNRGKPNW
436	Q96PU8	205	LNGTYRDANIK
437	Q96PU8	256	IMPLIRQIQTA
438	Q96PU8	320	ATKVRRHDMRV
439	Q96PU8	324	RRHDMRVHPYQ
440	Q92804	216	NFGGHRDYGPR
441	Q92804	334	GSGGGRRGRGG
442	Q92804	335	SGGGRRGRGGY
443	Q92804	411	RGRGGRGGDRG
444	P09867	122	EEHHLRDYFEQ
445	P09867	140	EIMTDRGSGKK
446	P09867	284	GNFGGRSSGPY
447	O00267	71	PPKKPRHGGFI
448	O00267	190	KIGEERATAIS
449	O00267	198	AISLMRKFIAY
450	O00267	610	GPHSGREGEIR
451	O00267	615	REGEIRHLFRS
452	O00267	619	IRHLFRSFAFL
453	O00267	767	TSTYGRTPMYG
454	O00267	989	IQVKVRDTYLD
455	P69697	49	GISYGRKKRRQ
456	Q15554	41	GGPAERGAGEA
457	Q15554	274	PREPARQLRNP
458	P15056	199	CCAVYRIQDGE
459	P15056	362	FGQRDRSSSAP
460	P15056	384	IDDLIRDQGFR
461	P15056	435	SSSEDRNRMKT
462	Q5TAQ9	65	TSTESRGTDTE
463	Q5TAQ9	94	INDENRVHDRS
464	Q5TAQ9	117	EQPRRRVQRKR
465	Q5TAQ9	125	RKRANRDQDSS
466	Q5TAQ9	134	SSDDERALEDW
467	Q5TAQ9	149	TSALPRPRWQA
468	Q5TAQ9	159	ALPALRERELG
469	Q96EY1	25	PAISGRGARPP
470	Q96EY1	340	KSPVFRRDGAD
471	Q96EY1	341	SPVFRRDGADI
472	P06746	182	VCGSFRRGAES
473	Q92997	73	PCFNGRVVSWL
474	Q92997	105	PPPMERTGGIG

475	Q92997	200	DDSTSRFSSST
476	Q92997	219	MRRHKRRRRKQ
477	Q60487	16	AAAARREPGGA
478	Q60487	96	GVCANRYLAMK
479	Q60487	144	YVALKRTGQYK
480	Q54G21	235	RKSAKRILVIP
481	Q54G21	613	VQTLHRLVEDN
482	Q54G21	673	VAQMRRQIIDL
483	Q54G21	746	YALKIRAFKKS
484	Q54G21	1031	KDVIKRVTVEY
485	Q9R1E0	222	HSKFIRVQNEG
486	Q9R1E0	264	KFAKSRGRAAK
487	Q13283	32	PDMLHRFYGKN
488	Q13283	78	CHTKIRHVDAH
489	Q13283	302	PQRDQRVREQR
490	Q13283	304	RDQRVREQRIN
491	Q13283	317	PQRGPRPIREA
492	Q13283	331	GDIEPRRMVRH
493	Q13283	332	DIEPRRMVRHP
494	Q13283	370	NVVELRINSGG
495	P31943	49	RFIYTREGRPS
496	P31943	199	HYDPPRKLMAM
497	P04264	12	SRSGYRSGGGF
498	P04264	29	IINYQRRTTSS
499	P04264	239	FINNLRRRVDQ
500	P08729	13	PVFTSRSAAFS
501	P02687	129	FGYGGRASDYK
502	P02687	158	FKLGGRDSRSG
503	P15720	52	DRHVPRRGFGK
504	P15720	112	GLSLTRFSWGG
505	P25274	111	GTVLSRFSWGA
506	P11940	49	RDMITRRSLGY
507	P11940	67	PADAERALDTM
508	P11940	83	KGKPVRIMWSQ
509	P11940	94	RDPSLRKSGVG
510	P11940	240	FVSFERHEDAQ
511	Q9JMB7	8	GRARARARGRA
512	Q9JMB7	10	ARARARGRARG
513	Q9JMB7	53	GRGRQRGMVVG
514	Q9JMB7	95	LGVNTRQNLDH
515	Q9JMB7	193	NGEHVRITITL
516	Q8CDG1	21	PSQCVRMPGCW
517	Q8CDG1	31	WPQAPRPLEPA
518	Q8CDG1	50	RGLVFRKPEDS
519	Q8CDG1	206	VLTMERKEKEL
520	Q86WR7	186	PVEHPRLLRSV
521	P22626	62	ASKRSRGFGFV
522	P22626	99	KRAVAREESGK
523	P22626	147	EIITDRQSGKK
524	P51991	167	QSGKKRGFAFV
525	Q99729	28	VPEASRGRGWT
526	P14678	16	QHIDYRMRCIL

527	P14678	73	GLVLLRGENLV
528	P14678	108	GPGIGRAAGRG
529	P14678	209	GIPPGRGTPMG
530	Q14524	34	AEKQARGSTTL
531	Q14524	43	TLQESREGLPE
532	Q14524	222	ALRTFRVLRAL
533	Q14524	225	TFRVLRALKTI
534	Q14524	282	RHKCVRNFTAL
535	P23246	9	DRFRSRGGGGG
536	P23246	267	GGPGGRSEEKI
537	P23246	315	EDEFKRLFAKY
538	P23246	342	IKLESRALAEI
539	P68363	221	NLDIERPTYTN
540	P07437	262	MVPFPRLHFFM
541	Q86V81	29	GGGRGRGRAGS
542	Q86V81	73	GGGRNRPAPYS
543	Q86V81	155	DVHFERKADAL
544	Q86V81	204	GMTRNRGAGGF
545	O08583	38	GSQGGRGGAVQ
546	O08583	188	QIDTQRRPAQS
547	O08583	196	AQSINRGGMTR
548	P31269	106	AAPDGRYMRSW
549	P31269	109	DGRYMRSWLEP
550	P31269	248	LNLTERQVKIW
551	P31269	258	WFQNRRMKMKK
552	P04049	316	PVPAQRERAPV
553	O76922	11	NPVIARGRGRG
554	O76922	26	NVEANRGFAPS
555	O76922	189	LVTKSRAGENI
556	Q9NR22	95	LKDEVRTLTYR
557	Q9NR22	204	TVIFARDKWLK
558	Q9NR22	218	LMFPDRAALYV
559	Q9NR22	300	CLQIQRNDYVH
560	Q9UBL3	108	DEENGRQLGEV
561	Q9UBL3	160	NTYFLRKQANL
562	Q92934	109	LWAAQRYGREL
563	Q7Q2B7	261	KMYPSRGDLHV
564	P11911	125	RNPVPRPFLDM
565	P11911	160	TLLLFRKRWQN
566	Q09472	237	GQTGLRGPQPL
567	Q09472	353	AHKCQRREQAN
568	Q09472	466	PSSIERAYAAL
569	Q09472	754	MGYGPRMQQPS
570	Q09472	883	LHPPPRQTPTP
571	Q09472	915	PQQQPRSQQST
572	Q09472	1055	KPEELRQALMP
573	Q09472	1137	NRKTSRVYKYC
574	Q09472	1166	GYCCGRKLEFS
575	P03372	162	RRQGGRERLAS
576	P03372	243	QACRLRKCYEV
577	P03372	259	GIRKDRRGGRM
578	P03372	277	DDGEGRGEVGS

579	P03372	300	PLMIKRSKKNS
580	P03372	394	IGLVWRSMEHP
581	P03372	434	LATSSRFRMMN
582	P03372	548	MLDAHRLHAPT
583	Q01844	138	ATAPTRPQDGN
584	Q01844	461	GGLPPREGRGM
585	Q01844	518	GNVQHRAGDWQ
586	P22509	80	GRGGGRGGKRG
587	P22509	213	NLAKKRTNIIP
588	P22509	250	QPDQTRIVALN
589	P35637	371	VSFATRRADFN
590	P35637	472	NYGDDRRGGRG
591	P35637	514	GKMDSRGEHRQ
592	P03347	91	YCVHQRIEIKD
593	P03347	150	QAISPRTLNAW
594	P03347	380	TIMMQRGNFRN
595	P03347	464	PFLQSRPEPTA
596	P03347	476	PEESFRSGVET
597	P68431	70	KLPFQRLVREI
598	P62805	36	TKPAIRRLARR
599	P62805	41	RRLARRGGVKR
600	P62805	56	IYEETRGVLKV
601	P17096	73	GAAKTRKTTTT
602	P17096	86	RKPRGRPKKLE
603	P31942	116	PGPYDRPIGGR
604	P31942	137	MYDRMRRGGDG
605	P31942	232	IGADGRATGEA
606	O60506	60	SDLDERAIEAL
607	O60506	286	DKKKNRGFCFL
608	O60506	343	KVLFVRNLANT
609	O60506	520	APPRGRAGYSQ
610	Q07666	17	SRSSGRSGSMD
611	Q07666	213	KEEELRKGGDP
612	Q07666	289	RGVPVRGRGAA
613	Q07666	354	TAGIQRIPLPP
614	P08199	281	PAPGKRKKEMT
615	P08199	471	GEKGQRQERTG
616	P08199	551	MEIEGRTIRLE
617	P08199	597	FEGSVRARIVT
618	Q28165	24	SGPGRRRHLVP
619	Q28165	125	EAIKARVREME
620	O75925	28	LGYAGRNKHGR
621	O75925	163	SDNSQRFRETC
622	O75925	296	TVLLQRLRAKG
623	O75925	330	ATTSLRVSLLC
624	O75925	632	HTVTNRSSTDT
625	Q96PU8	43	FNHLERLLDEE
626	Q96PU8	101	FNFVGRILGPR
627	Q96PU8	330	VHPYQRIVTAD
628	Q96PU8	336	IVTADRAATGN
629	Q92804	153	SYHSQRENYSH
630	Q92804	169	RRDVSRYGEDN

631	Q92804	187	GGGRGRGGYDK
632	Q92804	195	YDKDGRGPMTG
633	Q92804	326	RPEFMRGGGSG
634	Q92804	373	MNFARRNSCNQ
635	Q92804	395	SGGDFRGRGYG
636	Q92804	441	GYSGDRSGGGY
637	Q92804	526	GYGGDRSRGGY
638	P09867	53	DPNTKRSRGFG
639	P09867	178	HNCEVRKALSK
640	P09867	218	NDNFGRGGNFS
641	O00267	123	DRSGARRLQNL
642	O00267	133	LWRDQREEELG
643	O00267	246	GVGNLRLGYWN
644	O00267	334	RKKFKRPPQRL
645	O00267	416	STGKEREHNFQ
646	O00267	747	TISVDRQRLTT
647	Q15554	25	SRSSGRARRGR
648	Q15554	66	FHEALRAFRGS
649	Q15554	89	QALLVRPLGKE
650	Q15554	200	LLNIIREKNLA
651	Q15554	270	VEKPPREPARQ
652	Q15554	361	RMTISRLVLEE
653	Q15554	490	VMIKDRWRTMK
654	Q5TAQ9	116	EEQPRRRVQRK
655	Q96EY1	8	ARCSTRWLLVV
656	Q96EY1	28	SGRGARPPREG
657	Q96EY1	319	DGQTVRMPVGK
658	Q96EY1	325	MPVGKREIFIT
659	P06746	40	KYNAYRKAASV
660	P06746	112	GPSAARKFVDE
661	P06746	253	KEYPHRRIDIR
662	P06746	299	NEYTIRPLGVT
663	P06746	328	IQWKYREPKDR
664	Q92997	231	VSRIERSSSFS
665	Q60487	63	IHPDGRVDGVR
666	Q60487	84	LQAEDRGVVSI
667	Q60487	131	NYNTYRSRKYS
668	Q9R1E0	21	PLPRQRSCTWP
669	Q9R1E0	98	AAAASRGLCGD
670	Q9R1E0	154	TSSSRRNAWGN
671	Q9R1E0	266	AKSRGRAAKKK
672	Q9R1E0	311	NWSTFRPRTSS
673	Q13283	299	PQRPQRDQRVR
674	Q13283	307	RVREQRINIPP
675	Q13283	335	PRRMVRHPDSH
676	P31943	81	ETMGHRYVEVF
677	P31943	114	NDGFVRLRGLP
678	P31943	224	YNSIGRGAGFE
679	P31943	232	GFERMRRGAYG
680	P31943	262	SDRFGRDLNYC
681	P04264	179	QKVKSREREQI
682	P04264	240	INNLRRRVDQL

683	P04264	241	NNLRRRVDQLK
684	P04264	403	ISELNRVIQRL
685	P08729	25	RGAQVRLSSAR
686	P08729	88	SLQRVRQEESE
687	P08729	161	QVDGGRLEAEL
688	P08729	226	EINFLRTLNET
689	Q36736	21	DQEFNRKMQEQ
690	P02687	9	KRPSQRSKYLA
691	P02687	63	GHHAARTTHYG
692	P15720	53	RHVPRRGFGKD
693	P25274	63	KDHAARTTHYG
694	P25274	77	QKSGHRPQDEN
695	Q8IZS5	91	GNEYGRNYFDP
696	Q8IZS5	118	REDDDRIFYNR
697	Q8IZS5	221	VTAVIRSRRYY
698	Q8IZS5	223	AVIRSRRYYRE
699	P11940	44	SIRVCRDMITR
700	P11940	172	KVFVGRFKSRK
701	Q9JMB7	125	FRLTSRPQWAL
702	Q8CDG1	39	EPAWGRAGPAG
703	Q8CDG1	108	SANMVRKDREE
704	Q8CDG1	115	DREEPRSSLPD
705	Q86WR7	231	RTPAARGPRSG
706	Q86WR7	268	AAREPRRTLSR
707	P51991	16	QPDSGRRRRRR
708	P51991	19	SGRRRRRRGEE
709	P51991	20	GRRRRRRGEEG
710	Q99729	194	PKLNKRRGFVF
711	Q99729	322	GKSQRRGGHQN
712	P14678	94	DTGIARVPLAG
713	P14678	112	GRAAGRGIPAG
714	Q14524	219	NVSALRTFRVL
715	Q14524	376	QDCWERLYQQT
716	Q14524	433	EEKEKRFQEAM
717	P23246	32	GLHDFRSPPPG
718	P23246	287	LSLLRRPGEKT
719	P23246	298	YTQRCRLFVGN
720	P23246	407	VIVDDRGRSTG
721	P07437	62	GKYVPRAILVD
722	P07437	390	FTAMFRRKAFL
723	Q86V81	31	GRGRGRAGSQG
724	Q86V81	47	AQAAARVNRGG
725	O08583	50	AARVNRGGGPM
726	P39748	29	KSYFGRKVAID
727	P39748	47	FLIAVRQGGDV
728	P39748	261	IEEIVRRLDPN
729	P39748	320	QFSEERIRSGV
730	P31269	210	STRKKRCPYTK
731	P31269	257	IWFQNRRMKMK
732	P04049	73	TVVNVRNGMSL
733	P04049	111	KGKKARLDWNT
734	P04049	218	TMRRMRESVSR

735	P04049	256	LSQRQRSTSTP
736	P46783	65	EQFAWRHFYWY
737	P46783	119	PARLTRGEADR
738	O76922	96	DLVYSRPPGMT
739	O76922	248	NLENFRMQLWP
740	O76922	291	LSDAIRDSDDY
741	Q9NR22	251	DMTCIRDVAMK
742	Q9UBL3	213	MTTRQRPGKMT
743	Q9UBL3	621	TEVDGRRSPPW
744	Q92934	20	SSSAERGLGPS
745	Q92934	115	YGRELRRMSDE
746	Q7Q2B7	146	TSTYQRAIYNN
747	Q7Q2B7	239	MLYNERMLETY
748	Q7Q2B7	308	MKEYFRQPIVD
749	Q7Q2B7	326	MAKSIRHTTNF
750	P11911	30	GCQALRVEGGP
751	P11911	55	CENNGRNPNIT
752	P11911	120	TYLRVRNPVPR
753	Q09472	200	SIGAGRGRQNM
754	Q09472	362	ANGEVRQCNLP
755	Q09472	424	NAGDKRNQQPI
756	Q09472	613	LVAYARKVEGD
757	Q09472	648	ELEEKRRTRLQ
758	Q09472	651	EKRRTRLQKQN
759	Q09472	695	DPSMIRGSVPN
760	Q09472	728	PPIVPRQTPPL
761	Q09472	838	SPVPSRTPTPH
762	Q09472	1067	LEALYRQDPES
763	Q09472	1234	EQFSKRKNDTL
764	Q09472	1281	LKKSARTRKEN
765	Q09472	1305	TFLENRVNDFL
766	Q09472	1311	VNDFLRRQNHP
767	Q09472	1312	NDFLRRQNHPE
768	Q09472	1391	PPPNQRRVYIS
769	Q09472	1405	SVHFFRPKCLR
770	P03372	211	KAFFKRSIQGH
771	P03372	233	TIDKNRRKSCQ
772	P03372	412	NLLLDRNQGKC
773	P03372	477	KDHIHRVLDKI
774	P03372	555	HAPTSRGGASV
775	Q01844	446	KVSLARKKPPM
776	Q01844	648	DKGEHRQERRD
777	Q01844	651	EHRQERRDRPY
778	P22509	64	GGGGGRGGGFQ
779	P22509	128	VYGEKRVSISE
780	P22509	204	SHRSGRDLINL
781	P22509	310	LEPYERDHAVV
782	P03347	15	GGELDRWEKIR
783	P03347	58	TSEGCRQILGQ
784	P03347	361	PGHKARVLAEA
785	P03347	406	TARNCRAPRKK
786	P03347	444	PSYKGRPGNFL

787	P17096	30	GRGRPRKQPPV
788	P31942	80	GKHKERIGHRY
789	P31942	200	HFVHMRGLPFR
790	O60506	142	VTTGQRKYGGP
791	O60506	192	PIWDLRLMMDP
792	O60506	203	LTGLNRGYAFV
793	O60506	366	FGKLERVKKLK
794	O60506	448	TRGRGRGGRGG
795	O60506	501	RGRGGRGARGA
796	O60506	546	GAQQQRGRGVR
797	Q07666	186	GNTIKRLQEET
798	Q07666	302	PPPVPRGRGVG
799	P08199	561	ELQGPRGSPNA
800	P08199	567	GSPNARSQPSK
801	Q28165	227	DKESVRTSLAL
802	Q28165	240	SLFRGRQIKVI
803	O75925	165	NSQRFRETCFA
804	Q96PU8	51	DEEISRVRKDM
805	Q96PU8	68	GSTEKRSAELP
806	Q96PU8	130	GKGSMRDKKKE
807	Q92804	337	GGRRGRGGYRG
808	Q92804	343	GGYRGRGGFQG
809	Q92804	545	GYGGDRSGGYG
810	P09867	75	AAMNARPHKVD
811	P09867	97	REDSQRPGAHL
812	P09867	146	GSGKKRGFAFV
813	O00267	119	VLDEDRSGARR
814	O00267	469	PAQELRKYFKM
815	O00267	552	VGVIVRLERET
816	O00267	555	IVRLERETFQV
817	O00267	668	APMSPRISSPM
818	O00267	700	SRGRGRRDNEL
819	O00267	701	RGRGRRDNELI
820	O00267	757	TVGSRRPGGMT
821	O00267	1005	QTGVIRSVTGG
822	P69697	55	KKRRQRRRPPQ
823	Q15554	27	SSGRARRGRHE
824	Q15554	99	EHTVSRLLRVM
825	Q15554	109	MQCLSRIEEGE
826	Q15554	492	IKDRWRTMKRL
827	P15056	95	DALQQREQQLL
828	P15056	178	CGVTVRDSLKK
829	P15056	443	MKTLGRRDSSD
830	Q5TAQ9	98	NRVHDRSEEEE
831	Q5TAQ9	122	RVQRKRANRDQ
832	Q5TAQ9	225	WDWVRRQPVLD
833	Q96EY1	41	GAWLSRKLSVP
834	Q96EY1	64	ALLTLRPGVSL
835	Q96EY1	151	SDEVKRKQYDA
836	Q96EY1	277	RSTCRRCGGRG
837	P06746	149	GDFEKRIPREE
838	P06746	254	EYPHRRIDIRL

839	Q92997	113	GIGDSRPPSFH
840	Q92997	149	ERPRRRDGPEH
841	Q92997	168	KGERRREPGGY
842	Q92997	216	SRLMRRHKRRR
843	Q92997	221	RHKRRRRKQKV
844	Q60487	121	CFFFERLESNN
845	Q60487	133	NTYRSRKYSSW
846	Q54G21	194	LENNQRVGSLF
847	Q54G21	210	SETPFRFVVGE
848	Q9R1E0	29	TWPLPRPEFNQ
849	Q9R1E0	606	GMFIERLDCDM
850	Q13283	17	GREFVRQYYTL
851	Q13283	132	HNDIFRYQDEV
852	Q13283	247	VQEDLRTFSWA
853	Q13283	314	NIPPQRGPRPI
854	P31943	75	ALKKDRETMGH
855	P31943	175	KKHKERIGHRY
856	P04264	8	RQFSSRSGYRS
857	P04264	38	SSSTRRSGGGG
858	P04264	181	VKSREREQIKS
859	P04264	223	VDTSTRTHNLE
860	P04264	267	MVEDYRNKYED
861	P04264	277	DEINKRTNAEN
862	P08729	136	SAKSSRLPDIF
863	P08729	302	HGDDLRNTRNE
864	P02687	31	FLPRHRDTGIL
865	P02687	96	NIVTPRTPPPS
866	P15720	30	RHGSPRHRDSG
867	P15720	42	LDSLGRFFGGD
868	P25274	150	QGTLSRLFKLG
869	Q8IZS5	132	LFDESRQGEPQ
870	Q8IZS5	195	LEQEIRSTSLH
871	P11940	265	QIYVGRAQKKV
872	Q9JMB7	80	LSLAERGGRRR
873	Q9JMB7	85	RGGRRRDFHDL
874	Q9JMB7	121	STNHFRLTSRP
875	Q9JMB7	174	LFLPKRLQHKV
876	Q86WR7	24	PSCRLRAFSRG
877	Q86WR7	40	RSSSSRSRSFT
878	Q86WR7	42	SSSRSRSFTLD
879	Q86WR7	172	TPDPPRRELRA
880	Q86WR7	234	AARGPRSGDPG
881	Q86WR7	269	AREPRRTLSRA
882	Q86WR7	273	RRTLSRAAVSV
883	P22626	38	TEESLRNYYEQ
884	P22626	89	HSIDGRVVEPK
885	P51991	18	DSGRRRRRRGE
886	P51991	68	DCVVMRDPQTK
887	P51991	74	DPQTKRSRGFG
888	P51991	246	GNFGGRGGYGG
889	Q99729	137	HRLDGRVIDPK
890	Q99729	171	TEEKIREYFGE

891	Q99729	321	YGKSQRRGGHQ
892	P14678	25	ILQDGRIFIGT
893	P14678	61	SKQAEREEKRV
894	P14678	65	EREEKRVLGLV
895	P14678	132	LAGPVRGVGGP
896	Q14524	104	GKTIFRFSATN
897	Q14524	190	AFTFLRDPWNW
898	Q14524	383	YQQTLRSAGKI
899	Q14524	451	EALTIRGVDTV
900	P23246	358	DDTPMRGRQLR
901	P68363	79	VIDEVRTGTYR
902	P68363	123	VLDRIRKLADQ
903	P68363	214	IYDICRRNLDI
904	P68363	402	LMYAKRAFVHW
905	P07437	156	LISKIREEYPD
906	P07437	320	AVFRGRMSMKE
907	Q86V81	58	GPIRNRPAIAR
908	Q86V81	71	AGGGGRNRPAP
909	Q86V81	141	AVHYDRSGRSL
910	Q86V81	190	IDAQRRPAQSV
911	O08583	70	AAGGGRNRPAP
912	O08583	201	RGGMTRNRGSG
913	P39748	103	AKRSERRAEAE
914	P39748	129	EKFTKRLVKVT
915	P39748	262	EEIVRRLDPNK
916	P31269	235	YLTRDRRYEVA
917	P04049	215	PSLTMRRMRES
918	P04049	216	SLTMRRMRESV
919	P46783	124	RGEADRDTYRR
920	P46783	153	TEFQFRGGFGR
921	O76922	80	SEGDPRGSVRG
922	O76922	132	TIYQYRVDFTP
923	O76922	145	EATRLRRSFLY
924	O76922	221	NLILRRAMEGL
925	O76922	233	LKLVSRYYYDP
926	Q9NR22	100	RTLTYRNSMYH
927	Q9UBL3	300	GRGAKRKQQDG
928	Q9UBL3	342	NKDGYRYILAE
929	Q9UBL3	388	LALHDRAPQLK
930	Q92934	39	SGKHHRQAPGL
931	Q92934	70	GAVEIRSRHSS
932	Q92934	98	FRGRSRSAPPN
933	Q92934	161	DRNLGRGSSAP
934	Q7Q2B7	112	SVFNLRTEDSS
935	Q7Q2B7	140	MQDFVRTSTYQ
936	Q09472	202	GAGRGRQNMQY
937	Q09472	397	HCASSRQIISH
938	Q09472	568	STTGIRKQWHE
939	Q09472	603	AALKDRRMENL
940	Q09472	705	NQMMPRITPQS
941	Q09472	1356	ESFPYRTKALF
942	P03372	157	PNSDNRRQGGR

943	P03372	271	KHKRQRDDGEG
944	P03372	287	SAGDMRAANLW
945	P03372	335	EYDPTRPFSEA
946	Q01844	292	GPGENRSMSGP
947	P22509	141	DKIEYRAWNPF
948	P22509	201	VEFSHRSGRDL
949	P22509	261	AHTFLRNGGHF
950	P22509	320	VVGVYRPPPKA
951	P35637	218	RGGRGRGGSGG
952	P35637	441	MNFSWRNECNQ
953	P03347	39	IVWASRELERF
954	P03347	43	SRELERFAVNP
955	P03347	76	GSEELRSLYNT
956	P03347	214	AAEWDRVHPVH
957	P03347	229	APGQMREPRGS
958	P03347	232	QMREPRGSDIA
959	P62805	20	GAKRHRKVLRD
960	P62805	93	VYALKRQGRTL
961	P17096	81	TTTPGRKPRGR
962	P31942	55	MDYQGRSTGEA
963	P31942	104	FYDPPRRLLGQ
964	P31942	110	RLLGQRPGPYD
965	P31942	169	DGFDDRMRDGR
966	O60506	130	KALLERTGYTL
967	O60506	171	VGKIPRDLFED
968	O60506	244	SVANNRLFVGS
969	O60506	304	AAQARRRLMSG
970	O60506	413	PPDQKRKERKA
971	O60506	478	DYHNYRGGYED
972	O60506	548	QQQRGRGVRGA
973	O60506	551	RGRGVRGARGG
974	Q07666	36	RQTPSRQPPLP
975	Q07666	159	MKLKERVLIPV
976	Q07666	348	PAPRARTAGIQ
977	Q07666	420	DWNGTRPSLKA
978	Q07666	429	KAPPARPVKGA
979	P08199	385	EKPKGRDSKKV
980	Q28165	76	EEEPPRPRAPP
981	Q28165	127	IKARVREMEEE
982	Q28165	251	PKRTNRPGIST
983	O75925	15	MVMSLRVSELQ
984	O75925	201	VQVQLRFCLSE
985	O75925	245	GVEPKRPSRPI
986	O75925	298	LLQRLRAKGIR
987	O75925	309	NPDHSRALIKE
988	O75925	341	PLGKMRLTIPC
989	O75925	622	SSNSLRESHSH
990	Q96PU8	170	EIKLKRAVEEV
991	Q96PU8	319	VATKVRRHDMR
992	Q92804	403	GYGGERGYRGR
993	Q92804	408	RGYRGRGGRGG
994	P09867	14	EPEQLRKLFIG

995	P09867	55	NTKRSRGFGFV
996	P09867	92	KRAVSREDSQR
997	P09867	232	GFGGSRGGGGY
998	O00267	310	LKMIPRIDYDR
999	O00267	365	IFEGNRYSRKG
1000	O00267	484	KVIAGRFEGDT
1001	O00267	733	TESTARVELHS
1002	O00267	749	SVDRQRLTTVG
1003	O00267	756	TTVGSRRPGGM
1004	P69697	57	RRQRRRPPQGS
1005	Q15554	21	GRRASRSSGRA
1006	Q15554	80	DFRQIRDIMQA
1007	Q15554	223	QQKMLRFLESH
1008	Q15554	277	PARQLRNPPTT
1009	Q15554	332	KNKRPRKDENE
1010	P15056	146	PTDVARSNPKS
1011	P15056	252	FCDFCRKLLFQ
1012	P15056	271	YKFHQRCSTEV
1013	P15056	354	ADEDHRNQFGQ
1014	P15056	437	SEDRNRMKTLG
1015	Q5TAQ9	115	EEEQPRRRVQR
1016	Q5TAQ9	168	LGSSARFVYEA
1017	P06746	183	CGSFRRGAESS
1018	P06746	283	FNKNMRAHALE
1019	Q92997	39	KGVLQRPSYKF
1020	Q92997	143	LVSAQRERPRR
1021	Q92997	147	QRERPRRRDGP
1022	Q92997	157	PEHATRLNGTA
1023	Q92997	166	TAKGERRREPG
1024	Q92997	222	HKRRRRKQKVS
1025	Q92997	228	KQKVSRIERSS
1026	Q60487	57	GGFFLRIHPDG
1027	Q54G21	339	NESNSRNSHSN
1028	Q54G21	631	VEKENRMVTLK
1029	Q9R1E0	147	LAGQPRKTSSS
1030	Q9R1E0	153	KTSSSRRNAWG
1031	Q9R1E0	177	SSAEKRLTLSQ
1032	Q9R1E0	211	WKNSIRHNLSL
1033	Q13283	398	KVLSNRPIMFR
1034	P31943	29	ADEVQRFFSDC
1035	P31943	188	IFKSSRAEVRT
1036	P04264	45	GGGGGRFSSCG
1037	P04264	251	KSDQSRLDSEL
1038	P04264	392	HGDSVRNSKIE
1039	P08729	253	SMDNSRSLDLD
1040	P02687	23	TMDHARHGFLP
1041	P02687	52	RGAPKRGSGKD
1042	P02687	78	KAQGHRPQDEN
1043	P02687	161	GGRDSRSGSPM
1044	P15720	6	MASQKRSSFRH
1045	P15720	32	GSPRHRDSGLL
1046	P15720	63	DIHAARASHVG

1047	P15720	73	GSIPQRSQHGR
1048	P11940	41	PILSIRVCRDM
1049	P11940	176	GRFKSRKEREA
1050	P11940	179	KSRKEREAELG
1051	P11940	272	QKKVERQTELK
1052	P11940	309	GIDDERLRKEF
1053	Q9JMB7	12	ARARGRARGQE
1054	Q8CDG1	45	AGPAGRGLVFR
1055	Q8CDG1	293	ELKSQRKTDDA
1056	Q86WR7	28	LRAFSRGGSLE
1057	Q86WR7	35	GSLESRSSSSR
1058	Q86WR7	218	PTSPFREGRPG
1059	Q86WR7	226	RPGEWRTPAAR
1060	P22626	12	TVPLERKKREK
1061	P22626	82	AAMAARPHSID
1062	P22626	129	EEHHLRDYFEE
1063	P22626	153	QSGKKRGFGFV
1064	P22626	190	RKALSRQEMQE
1065	P51991	17	PDSGRRRRRRG
1066	P51991	76	QTKRSRGFGFV
1067	P51991	161	EVMEDRQSGKK
1068	Q99729	110	NTGRSRGFGFI
1069	Q99729	133	DQKEHRLDGRV
1070	P14678	49	DCDEFRKIKPK
1071	P14678	181	PPPMGRGAPPP
1072	P14678	196	PPPGMRPPMGP
1073	Q14524	8	NFLLPRGTSSF
1074	Q14524	14	GTSSFRRFTRE
1075	Q14524	15	TSSFRRFTRES
1076	Q14524	122	FHPIRRAAVKI
1077	P23246	236	PKPPHRGGGEP
1078	P23246	286	NLSLLRRPGEK
1079	P23246	296	KTYTQRCRLFV
1080	P23246	360	TPMRGRQLRVR
1081	P23246	363	RGRQLRVRFAT
1082	P23246	376	AALSVRNLSPY
1083	P68363	84	RTGTYRQLFHP
1084	P68363	121	DLVLDRIRKLA
1085	P68363	156	SLLMERLSVDY
1086	P68363	229	YTNLNRLISQI
1087	P68363	422	EFSEAREDMAA
1088	P07437	121	VLDVVRKEAES
1089	P07437	251	LNADLRKLAVN
1090	P07437	309	DPRHGRYLTVA
1091	P07437	391	TAMFRRKAFLH
1092	Q86V81	17	IIKLNRSQRGG
1093	Q86V81	23	SQRGGRGGGRG
1094	Q86V81	27	GRGGGRGRGRA
1095	Q86V81	38	GSQGGRGGGAQ
1096	Q86V81	50	AARVNRGGGPI
1097	Q86V81	56	GGGPIRNRPAI
1098	O08583	47	VQAAARVNRGG

1099	P39748	327	RSGVKRLSKSR
1100	P31269	40	LGQPPRQAATL
1101	P31269	204	NWLHARSTRKK
1102	P31269	236	LTRDRRYEVAR
1103	P04049	40	QFGYQRRASDD
1104	P04049	41	FGYQRRASDDG
1105	P04049	89	KALKVRGLQPE
1106	P04049	191	DWSNIRQLLLF
1107	P04049	254	GSLSQRQRSTS
1108	P46783	95	VPATLRRSRPE
1109	P46783	103	RPETGRPRPKG
1110	P46783	129	RDTYRRSAVPP
1111	O76922	17	GRGRGRKPNNV
1112	Q9NR22	8	MKHSSRCLLLR
1113	Q9NR22	349	DYLTVRRGEEI
1114	Q9NR22	350	YLTVRRGEEIY
1115	Q9UBL3	398	KISDDRLTVVG
1116	Q9UBL3	461	FSYSWRSKKGT
1117	Q7Q2B7	206	NNLTDRIIVIA
1118	Q7Q2B7	318	DTFDIRICMAK
1119	Q7Q2B7	391	HWYQVRCLLQT
1120	Q7Q2B7	417	LAANQRQSYDV
1121	Q7Q2B7	535	ISSTGRQQQQQ
1122	P11911	111	NNILKRSCGTY
1123	Q09472	335	ADPEKRKLIQQ
1124	Q09472	1252	CTECGRKMHQI
1125	Q09472	1283	KSARTRKENKF
1126	Q09472	1292	KFSAKRLPSTR
1127	Q09472	1478	KAVSERIVHDY
1128	P03372	151	PPAFYRPNSDN
1129	P03372	164	QGGRERLASTN
1130	P03372	263	DRRGGRMLKHK
1131	P03372	352	TNLADRELVHM
1132	P03372	363	INWAKRVPGFV
1133	Q01844	268	QQSSFRQDHPS
1134	Q01844	330	GSAGERGGFNK
1135	Q01844	537	QNFAWRTECNQ
1136	P22509	46	FGGGGRGRGGG
1137	P22509	48	GGGRGRGGGGG
1138	P22509	98	MVEPHRHEGVF
1139	P22509	147	AWNPFRSKLAA
1140	P22509	230	HPHKYRMLIAM
1141	P35637	422	GGGQQRAGDWK
1142	P03347	264	GEIYKRWIILG
1143	P03347	286	SILDIRQGPKE
1144	P03347	294	PKEPFRDYVDR
1145	P03347	384	QRGNFRNQRKM
1146	P68431	27	ATKAARKSAPA
1147	P68431	53	ALREIRRYQKS
1148	P17096	56	VPTPKRPRGRP
1149	P31942	84	ERIGHRYIEIF
1150	P31942	90	YIEIFRSSRSE

1151	P31942	135	GSMYDRMRRGG
1152	P31942	138	YDRMRRGGDGY
1153	P31942	171	FDDRMRDGRGM
1154	O60506	105	KTYRQREKQGT
1155	O60506	416	QKRKERKAQRQ
1156	O60506	420	ERKAQRQAAKN
1157	O60506	498	VGARGRGGRGA
1158	O60506	512	APSRGRGAAPP
1159	O60506	533	GPGSARGVRGA
1160	Q07666	56	SRGGARASPAT
1161	P08199	391	DSKKVRAARTL
1162	P08199	458	AEIDGRSVSLY
1163	Q28165	172	MEADARSIYVG
1164	O75925	33	RNKHGRKHELL
1165	O75925	62	IKELYRRRFPQ
1166	O75925	64	ELYRRRFPQKI
1167	O75925	479	EPSAKRTCPSL
1168	Q96PU8	124	CKIMVRGKGSM
1169	Q96PU8	163	EDAQNRAEIKL
1170	Q96PU8	227	AQAAPRIITGP
1171	Q92804	175	YGEDNRGYGGS
1172	Q92804	383	QCNEPRPEDSR
1173	Q92804	397	GDFRGRGYGGE
1174	P09867	47	DCVVMRDPNTK
1175	O00267	124	RSGARRLQNLW
1176	O00267	280	PKSWVRLKRGI
1177	O00267	283	WVRLKRGIYKD
1178	O00267	315	RIDYDRIKARM
1179	O00267	329	DWFAKRKKFKR
1180	O00267	338	KRPPQRLFDAE
1181	O00267	494	TGLIVRVEENF
1182	O00267	577	HQAVTRKKDNR
1183	O00267	813	LHDGSRTPAQS
1184	Q15554	54	EEAVNRWVLKF
1185	Q15554	154	VVESSRKLVKE
1186	Q15554	330	ALKNKRPRKDE
1187	Q15554	482	YPFVNRTAVMI
1188	P15056	158	QKPIVRVFLPN
1189	P15056	166	LPNKQRTVVPA
1190	P15056	172	TVVPARCGVTV
1191	P15056	389	RDQGFRGDGGS
1192	P15056	424	SPGPQRERKSS
1193	P15056	444	KTLGRRDSSDD
1194	Q5TAQ9	33	GAEEGRETSSG
1195	Q5TAQ9	59	DGGPNRTSTES
1196	Q5TAQ9	120	RRRVQRKRANR
1197	Q5TAQ9	184	FVQRFRLQHGL
1198	Q5TAQ9	224	VWDWVRRQPVL
1199	Q96EY1	18	VVGTPRLPAIS
1200	Q96EY1	31	GARPPREGVVG
1201	Q92997	148	RERPRRRDGPE
1202	Q60487	15	TAAAARREPGG

1203	Q60487	68	RVDGVREKTDP
1204	Q54G21	12	EEYSNRNAASS
1205	Q54G21	320	VAKLGRATGFT
1206	Q54G21	672	NVAQMRRQIID
1207	Q54G21	759	DEKNARKSDIE
1208	Q54G21	800	KLDMVRIKKRN
1209	Q54G21	804	VRIKKRNDDKV
1210	Q9R1E0	83	SEDFARAPGCV
1211	Q9R1E0	324	STISGRLSPIM
1212	Q13283	13	PLLVGREFVRQ
1213	Q13283	63	QKEIHRKVMSQ
1214	Q13283	320	GPRPIREAGEQ
1215	P31943	44	GAQGIRFIYTR
1216	P31943	116	GFVRLRGLPFG
1217	P31943	150	VDFQGRSTGEA
1218	P31943	206	LMAMQRPGPYD
1219	P31943	212	PGPYDRPGAGR
1220	P31943	259	GFGSDRFGRDL
1221	P04264	86	ARGGGRGSGFG
1222	P08729	77	LLAPLRLDADP
1223	P08729	86	DPSLQRVRQEE
1224	P08729	149	QIAGLRGQLEA
1225	P08729	167	LEAELRSMQDV
1226	Q36736	53	YEKFERMIKEH
1227	P02687	29	HGFLPRHRDTG
1228	P02687	47	FFGSDRGAPKR
1229	P02687	112	GLSLSRFSWGA
1230	P15720	25	TTDHARHGSPR
1231	P25274	43	LDSIGRFFSSD
1232	Q8IZS5	105	EEINPRQCATE
1233	Q8IZS5	123	RIFYNRLTKLF
1234	Q8IZS5	224	VIRSRRYYREQ
1235	P11940	50	DMITRRSLGYA
1236	P11940	290	QDRITRYQGVN
1237	Q9JMB7	144	PLMEARRLRSA
1238	Q9JMB7	187	VFSQTRNGEHV
1239	Q9JMB7	218	YNIIFRRLLKI
1240	Q8CDG1	83	LDTAFRPPSKR
1241	Q8CDG1	140	SVGWSRMLGRG
1242	Q8CDG1	144	SRMLGRGSSEV
1243	Q8CDG1	181	ARDPPRLPQPP
1244	Q8CDG1	255	ECKSMRFGMLK
1245	Q8CDG1	326	YNVVFRRVMKL
1246	Q86WR7	189	HPRLLRSVPTP
1247	P22626	21	EKEQFRKLFIG
1248	P22626	54	DCVVMRDPASK
1249	P22626	95	VVEPKRAVARE
1250	P22626	200	EVQSSRSGRGG
1251	P22626	266	GYGGGRGGYGG
1252	P22626	325	NFGGSRNMGGP
1253	P51991	21	RRRRRRGEEGH
1254	P51991	96	AAMCARPHKVD

1255	P51991	214	SAGSQRGRGGG
1256	P51991	216	GSQRGRGGGSG
1257	Q99729	108	DPNTGRSRGFG
1258	Q99729	195	KLNKRRGFVFI
1259	Q99729	253	RNRGNRGSGGG
1260	P14678	18	IDYRMRCILQD
1261	P14678	172	QYPPGRGGPPP
1262	Q14524	18	FRRFTRESLAA
1263	Q14524	27	AAIEKRMAEKQ
1264	Q14524	53	EEEAPRPQLDL
1265	Q14524	121	PFHPIRRAAVK
1266	Q14524	277	FMGNLRHKCVR
1267	P23246	19	GGFHRRGGGGG
1268	P23246	44	GLNQNRGPMGP
1269	P23246	242	GGGEPRGGRQH
1270	P23246	245	EPRGGRQHHPP
1271	P23246	365	RQLRVRFATHA
1272	P68363	64	GKHVPRAVFVD
1273	P68363	373	LAKVQRAVCML
1274	P68363	390	AEAWARLDHKF
1275	P07437	46	DLQLDRISVYY
1276	P07437	86	FGQIFRPDNFV
1277	Q86V81	189	QIDAQRRPAQS
1278	O08583	58	GPMRNRPAIAR
1279	P39748	73	FYRTIRMMENG
1280	P39748	245	GIGPKRAVDLI
1281	P39748	322	SEERIRSGVKR
1282	P39748	339	GSTQGRLDDFF
1283	P31269	29	ELSVGRYAPGT
1284	P31269	166	SPPVDREKQPS
1285	P04049	67	LPNKQRTVVNV
1286	P04049	164	LLNGFRCQTCG
1287	P04049	275	LPVDSRMIEDA
1288	P04049	331	EKNKIRPRGQR
1289	P46783	98	TLRRSRPETGR
1290	P46783	113	GLEGERPARLT
1291	O76922	84	PRGSVRGRRLI
1292	O76922	143	DVEATRLRRSF
1293	O76922	220	LNLILRRAMEG
1294	O76922	260	YQTSIRQHEND
1295	O76922	278	CHKVMRTETLY
1296	O76922	302	QSTFKRAVMGM
