# Supplementary material for: iMethyl-PseAAC: Identification of Protein Methylation Sites via a Pseudo Amino Acid Composition Approach
Source: Biomed Res Int. 2014 May 22;2014:947416. doi: 10.1155/2014/947416 (PMC4054830; doi:10.1155/2014/947416)
Supplement: Supplementary file 1 — S1: An analysis of the benchmark dataset used in Hu et al. (Biopolymers, 2011, 95, 763-771) S2: List of self-conflict samples in the benchmark dataset used in Hu et al. (Biopolymers, 2011, 95, 763-771) S3: Benchmark dataset used for studying the Arg-methylation. It contains 1,481 samples, of which 185 are positive and 1,296 negative. These data were extracted from UniProtKB/Swiss-Prot database (version UniProt release 2013_06). S4: Benchmark dataset used for studying the Lys-methylation. It contains 1,884 samples, of which 226 are positive and 1,518 negative. These data were extracted from UniProtKB/Swiss-Prot database (version UniProt release 2013_06). S5: Seven negative subsets for studying Arg-methylation. Each subset contains 185 negative samples randomly taken from the 1,296 negative samples in Online Supporting Information S3 except for the 6th subset, which contains 186 samples. None of the samples in one subset occurs in any other subset. S6: Seven negative subsets for studying Lys-methylation. Each subset contains 217 negative samples randomly taken from the 1,518 negative samples in Online Supporting Information S4 except for the 5th subset, which only contains 216 samples. None of the samples in one subset occurs in any other subset. S7: Independent dataset for studying the Arg-methylation. It contains 75 samples, of which 20 are positive and 55 negative. None of the samples listed here occurs in Online Supporting Information S3. S8: Independent dataset for studying the Lys-methylation. It contains 40 samples, of which 14 are positive and 26 negative. None of the samples listed here occurs in Online Supporting Information S4. S9: The code for encoding the peptides investigated in this paper. [file 947416.f1.zip › S4.pdf]

**Online Supporting Information S4**. **Benchmark dataset used for studying the Lys-methylation.** It contains 1,884 samples, of which 226 are positive and 1,518 negative. These data were extracted from UniProtKB/Swiss-Prot database (version UniProt release 2013_06).

## (A) List of 226 positive Lys-methylation sites and sequence

| Seqence # | Protein ID | Site | Sequences |
|---|---|---|---|
| 1 | P05141 | 52 | ADKQYKGIIDC |
| 2 | Q9Y232 | 135 | PNNARKQISRS |
| 3 | P00042 | 61 | DANIKKAVEWS |
| 4 | P26358 | 70 | ETKLRKEELSE |
| 5 | O75530 | 66 | NAPGRKSWGKG |
| 6 | O75530 | 197 | AINELKFHPRD |
| 7 | O75530 | 268 | WRINSKRMMNA |
| 8 | O75530 | 284 | DYNPNKTNRPF |
| 9 | Q5VTE0 | 55 | GKGSFKYAWVL |
| 10 | Q5VTE0 | 165 | PPYSQKRYEEI |
| 11 | P0CE47 | 57 | NAPEEKARGIT |
| 12 | O75367 | 123 | RGSKGKLEAII |
| 13 | P62807 | 58 | TGISSKAMGIM |
| 14 | P68432 | 5 | MMARTKQTARK |
| 15 | P68432 | 10 | KQTARKSTGGK |
| 16 | P68432 | 28 | TKAARKSAPAT |
| 17 | P68432 | 37 | ATGGVKKPHRY |
| 18 | P68432 | 80 | IAQDFKTDLRF |
| 19 | P09988 | 15 | KSTGGKAPRKQ |
| 20 | P09988 | 28 | SKAARKAAPAT |
| 21 | P69150 | 28 | SKAARKSAPAT |
| 22 | P69150 | 37 | ATGGIKKPHRF |
| 23 | P59226 | 37 | ATGGVKKPHRF |
| 24 | O15819 | 10 | KQTARKSTGAK |
| 25 | O15819 | 28 | SKQAHKQTPVS |
| 26 | O15819 | 40 | SSGGVKKVHRF |
| 27 | O15819 | 83 | IAQEFKTDLRF |
| 28 | Q55BN9 | 28 | NKSSQKSFPST |
| 29 | Q55BN9 | 37 | STQGLKKTHRF |
| 30 | Q55BP0 | 37 | VNEVLKKTHRF |
| 31 | P59169 | 28 | TKAARKSAPTT |
| 32 | P59169 | 37 | TTGGVKKPHRY |
| 33 | P84249 | 28 | TKAARKSAPST |
| 34 | P84249 | 37 | STGGVKKPHRY |
| 35 | P84249 | 38 | TGGVKKPHRYR |
| 36 | P08898 | 28 | TKAARKSAPAS |
| 37 | P08898 | 37 | ASGGVKKPHRY |
| 38 | P61830 | 28 | SKAARKSAPST |
| 39 | P62803 | 21 | AKRHRKVLRDN |
| 40 | P04637 | 370 | HSSHLKSKKGQ |
| 41 | P04637 | 373 | HLKSKKGQSTS |
| 42 | P04637 | 382 | TSRHKKLMFKT |

| | | | |
|---|---|---|---|
| 43 | Q86L05 | 122 | LRQVPKLLGPG |
| 44 | Q7M1B9 | 105 | REIFDKVLAMA |
| 45 | P80322 | 20 | QISEFKMAFDM |
| 46 | P14909 | 203 | ISRDNKIILLS |
| 47 | P14909 | 385 | NEEVIKEGIQK |
| 48 | P39462 | 11 | LVEIGKPLSLQ |
| 49 | P39462 | 213 | AVEAAKRAGAD |
| 50 | P22498 | 116 | NENELKRLDEY |
| 51 | P22498 | 135 | YREIFKDLKSR |
| 52 | P22498 | 273 | QPLTDKDMEAV |
| 53 | P22498 | 311 | VRDDLKGRLDW |
| 54 | P22498 | 332 | VKRTEKGYVSL |
| 55 | P0C835 | 16 | KTPAGKEAELV |
| 56 | P80053 | 254 | GVINEKGIDVG |
| 57 | P80053 | 260 | GIDVGKAIEIK |
| 58 | P80053 | 372 | DEEAKKLIVDR |
| 59 | P80053 | 391 | YDYHQKKLEDH |
| 60 | P80053 | 392 | DYHQKKLEDHD |
| 61 | P13123 | 5 | MMVKVKFKYKG |
| 62 | P13123 | 7 | VKVKFKYKGEE |
| 63 | P61991 | 5 | MMATVKFKYKG |
| 64 | P61991 | 7 | ATVKFKYKGEE |
| 65 | P13125 | 7 | AKVRFKYKGEE |
| 66 | P13125 | 63 | LARAEKKKKKK |
| 67 | P13125 | 64 | ARAEKKKKKKK |
| 68 | P39476 | 61 | LQMLEKQKKKK |
| 69 | P39476 | 63 | MLEKQKKKKKK |
| 70 | P39476 | 64 | LEKQKKKKKKK |
| 71 | P26358 | 142 | TPRRSKSDGEA |
| 72 | P55907 | 30 | PVEPPKVLGIH |
| 73 | P81543 | 29 | PVENPKVLGIH |
| 74 | P81539 | 29 | YEPPTKLGIWG |
| 75 | P49949 | 29 | PVDPPKVLGIH |
| 76 | P49949 | 101 | AAIDVKPPPPP |
| 77 | P81542 | 30 | ADPPVKEKALG |
| 78 | P00219 | 29 | PVESPKVLGVH |
| 79 | O43524 | 46 | QASPAKPSGET |
| 80 | O43524 | 149 | SGQPRKCSSRR |
| 81 | O43524 | 230 | NEGTGKSSWWI |
| 82 | O43524 | 262 | SNKYTKSRGRA |
| 83 | O43524 | 271 | RAAKKKAALQT |
| 84 | O43524 | 290 | PSQLSKWPGSP |
| 85 | O43524 | 419 | FPYTTKGSGLG |
| 86 | Q08369 | 299 | CGLYMKLHGVP |
| 87 | P16403 | 34 | GGTPRKASGPP |
| 88 | P16403 | 187 | PKKAAKSAAKA |
| 89 | P10412 | 26 | KKKARKSAGAA |
| 90 | P16401 | 27 | KKATKKAAGAG |
| 91 | O75367 | 18 | TSRSAKAGVIF |
| 92 | P40283 | 4 | MMMAPKAEKKP |
| 93 | P62807 | 47 | VYKVLKQVHPD |
| 94 | P62807 | 109 | PGELAKHAVSE |

| | | | |
|---|---|---|---|
| 95 | P68432 | 19 | GKAPRKQLATK |
| 96 | P68432 | 24 | KQLATKAARKS |
| 97 | P68432 | 57 | IRRYQKSTELL |
| 98 | P68432 | 65 | ELLIRKLPFQR |
| 99 | P68432 | 123 | VTIMPKDIQLA |
| 100 | Q42681 | 28 | TKAARKTPATG |
| 101 | P09988 | 19 | GKAPRKQLASK |
| 102 | P09988 | 24 | KQLASKAARKA |
| 103 | P69150 | 57 | IRKYQKSTDLL |
| 104 | P69150 | 80 | IAHEFKAELRF |
| 105 | O15819 | 19 | AKVPRKHIGSK |
| 106 | O15819 | 24 | KHIGSKQAHKQ |
| 107 | Q55BN9 | 19 | AKVPRKHLGNK |
| 108 | Q55BN9 | 24 | KHLGNKSSQKS |
| 109 | Q55BP0 | 19 | AKVPRKHLSSK |
| 110 | Q55BP0 | 24 | KHLSSKSSFPS |
| 111 | P61830 | 24 | KQLASKAARKS |
| 112 | Q41811 | 21 | RKRHRKVLRDN |
| 113 | O14979 | 161 | SWDTSKKDLTE |
| 114 | Q14103 | 119 | KDYFSKFGEVV |
| 115 | P16862 | 180 | LAYRMKTTDTY |
| 116 | O00159 | 383 | RDALAKAVYSR |
| 117 | P13538 | 36 | KPFDAKSSVFV |
| 118 | Q96RI1 | 220 | IQCKSKRLRKN |
| 119 | P04637 | 372 | SHLKSKKGQST |
| 120 | P11940 | 299 | VNLYVKNLDDG |
| 121 | P06400 | 810 | YISPLKSPYKI |
| 122 | P06400 | 860 | SDRVLKRSAEG |
| 123 | P0CX43 | 47 | DPQRDKRFSGS |
| 124 | P47914 | 5 | MMAKSKNHTTH |
| 125 | Q969Q0 | 53 | YGGQTKPIFRK |
| 126 | P0CX27 | 40 | LFAQGKRRYDR |
| 127 | P0CX27 | 55 | FGGQTKPVFHK |
| 128 | P02393 | 77 | KIGVIKVVRAL |
| 129 | P02393 | 88 | TGLGLKEAKDK |
| 130 | P0A7K2 | 82 | TGLGLKEAKDL |
| 131 | P07472 | 84 | TGLGLKEAKAA |
| 132 | Q12962 | 189 | SRSKSKDRKYT |
| 133 | Q04207 | 310 | TYETFKSIMKK |
| 134 | P84747 | 2 | PPPPPKVSPRW |
| 135 | Q53688 | 27 | NLWYFKDLGVS |
| 136 | Q53688 | 105 | LMDVLKMGKKS |
| 137 | Q53688 | 108 | VLKMGKKSKYY |
| 138 | Q53688 | 310 | ISESIKKIKAQ |
| 139 | Q53688 | 326 | FSYEVKRLASQ |
| 140 | Q53688 | 467 | EEWKNKVEEWH |
| 141 | Q53688 | 508 | FKERIKQHMIK |
| 142 | Q53688 | 513 | KQHMIKSVREA |
| 143 | Q53688 | 519 | SVREAKINTSW |
| 144 | Q53688 | 638 | MYLTYKLLSLR |
| 145 | E5KWG9 | 117 | AGGFTKTWSFP |
| 146 | B6A879 | 150 | VGDKTKPKLSA |

| | | | |
|---|---|---|---|
| 147 | B6A879 | 235 | LPQIGKGHIVP |
| 148 | B6A879 | 405 | KAKLKKSNIDQ |
| 149 | B6A879 | 518 | FFDIVKNYMDA |
| 150 | B6A879 | 620 | LYNPGKEIYLK |
| 151 | B6A879 | 625 | KEIYLKSISEI |
| 152 | Q9L5X8 | 325 | SPFAGKEGKFV |
| 153 | Q9L5X8 | 394 | PEVIIKEEDGQ |
| 154 | Q6NC90 | 67 | ERLGGKVVWQG |
| 155 | Q6NC90 | 114 | YREAVKHRQAA |
| 156 | Q6NC90 | 129 | RLIRLKPLKPG |
| 157 | Q9I756 | 9 | VGFYGKLAGRG |
| 158 | D0ZRV8 | 216 | EAFWAKLDALV |
| 159 | Q6NEZ3 | 267 | VTEDNKASTSI |
| 160 | Q6NEZ3 | 440 | EFEIPKTLAPG |
| 161 | D1BQI7 | 3 | MMMMYKYITIL |
| 162 | D1BQI7 | 17 | GQIAQKLTATL |
| 163 | D1BQI7 | 63 | FQNPGKLEQAV |
| 164 | D1BQI7 | 95 | KALSRKNIRRV |
| 165 | D1BQI7 | 160 | YNDPEKTDYEL |
| 166 | D1BQI7 | 214 | GTHYDKPSFHH |
| 167 | Q9UKV3 | 654 | SSNSRKSLSPG |
| 168 | P18281 | 35 | NVTVKKVDRSS |
| 169 | P18281 | 72 | DVHVKKVDRGA |
| 170 | Q9SJU4 | 395 | EGMFVKGYTYY |
| 171 | Q9ZU52 | 387 | KGMFVKGYTYY |
| 172 | P62157 | 116 | TNLGEKLTDEE |
| 173 | P62152 | 95 | FRVFDKDGNGF |
| 174 | P11118 | 149 | KMMMSKKKKKK |
| 175 | P07463 | 116 | TNLGEKLTDDE |
| 176 | P04573 | 96 | ILRAFKVFDAN |
| 177 | P04573 | 117 | KFIMQKVGEEP |
| 178 | P00889 | 395 | EQGKAKNPWPN |
| 179 | P00044 | 78 | YLTNPKKYIPG |
| 180 | P00044 | 79 | LTNPKKYIPGT |
| 181 | P00063 | 80 | YLLNPKKYIPG |
| 182 | P00063 | 94 | VFPGLKKPQDR |
| 183 | P00078 | 4 | MMMPPKARAPL |
| 184 | P00078 | 83 | YLENPKKFMPG |
| 185 | P00077 | 4 | MMMPPKAREPL |
| 186 | P00043 | 79 | YLENPKKYIPG |
| 187 | P00076 | 85 | AFAGIKAKKDR |
| 188 | P00042 | 79 | LENPKKYIPGT |
| 189 | P00070 | 95 | VFPGLKKPQER |
| 190 | P00047 | 94 | AFGGLKKNKDR |
| 191 | P68105 | 36 | CGGIDKRTIEK |
| 192 | P68105 | 79 | DISLWKFETSK |
| 193 | P68105 | 318 | KNVSVKDVRRG |
| 194 | Q71V39 | 165 | PAYSEKRYDEI |
| 195 | P02993 | 79 | DIALWKFETAK |
| 196 | P02993 | 219 | WNIERKEGKAD |
| 197 | P02993 | 318 | KNVSVKELRRG |
| 198 | P02994 | 79 | DIALWKFETPK |

| | | | |
|---|---|---|---|
| 199 | Q96KQ7 | 185 | VHRARKTMSKP |
| 200 | P53991 | 118 | IPPGTKINSKG |
| 201 | P53991 | 124 | INSKGKEVPTA |
| 202 | P07041 | 80 | IAQDFKSDLRF |
| 203 | P10587 | 128 | VINPYKQLPIY |
| 204 | P13538 | 131 | TVNPYKWLPVY |
| 205 | P13538 | 552 | TDTSFKNKLYD |
| 206 | P68696 | 104 | GVYNEKIQPGT |
| 207 | P11416 | 347 | LEQPDKVDMLQ |
| 208 | P27063 | 12 | ASVGFKAGVKK |
| 209 | P27064 | 14 | ASVGFKAGVKD |
| 210 | P04717 | 14 | AKVGFKAGVKD |
| 211 | P04992 | 14 | ASVGFKAGVKE |
| 212 | P0A7J7 | 4 | MMMAKKVQAYV |
| 213 | P0A7J7 | 40 | IMEFCKAFNAK |
| 214 | Q5SLP6 | 3 | MMMMKKVVAVV |
| 215 | P0CX53 | 4 | MMMPPKFDPNE |
| 216 | P0CX53 | 11 | DPNEVKYLYLR |
| 217 | P62986 | 98 | KMICRKCYARL |
| 218 | P55072 | 315 | APKREKTHGEV |
| 219 | P05547 | 142 | RGKFIKPTLKK |
| 220 | P05547 | 146 | IKPTLKKVSKY |
| 221 | O95785 | 1162 | PPTARKMFPGL |
| 222 | B6A879 | 364 | NISSDKDGENY |
| 223 | B6A879 | 390 | GYSNRKEISIA |
| 224 | B6A879 | 400 | ACSGVKAKLKK |
| 225 | B6A879 | 474 | GIPMEKIHLGY |
| 226 | B6A879 | 498 | TRQYTKNGPAL |

## (B) List of 1,518 negative Lys-methylation sites and sequence

| Seqence # | Protein ID | Site | Sequences |
|---|---|---|---|
| 1 | P05141 | 49 | QITADKQYKGI |
| 2 | P05141 | 94 | FAFKDKYKQIF |
| 3 | P05141 | 96 | FKDKYKQIFLG |
| 4 | P05141 | 105 | LGGVDKRTQFW |
| 5 | P05141 | 245 | MQSGRKGTDIM |
| 6 | P05141 | 272 | GKAFFKGAWSN |
| 7 | Q9Y232 | 73 | DKRKNKKGKTE |
| 8 | Q9Y232 | 74 | KRKNKKGKTEY |
| 9 | Q9Y232 | 157 | ALVIGKDHESK |
| 10 | Q9Y232 | 162 | KDHESKNSQLF |
| 11 | P00042 | 11 | KGSEKKGATLF |
| 12 | P00042 | 17 | GATLFKTRCLQ |
| 13 | P00042 | 48 | GRQSGKAEGYS |
| 14 | P00042 | 60 | TDANIKKAVEW |
| 15 | P26358 | 58 | LQTEIKNQLCD |
| 16 | P26358 | 83 | YLAKVKSLLNK |
| 17 | P26358 | 177 | AKGPAKRKPQE |
| 18 | P26358 | 198 | IKEEDKDQDEK |

| | | | |
|---|---|---|---|
| 19 | Q5VTE0 | 41 | KRTIEKFEKEA |
| 20 | Q5VTE0 | 129 | EAGISKNGQTR |
| 21 | P0CE47 | 238 | ERGIIKVGEEV |
| 22 | O75367 | 117 | ELLAKKRGSKG |
| 23 | P62807 | 12 | SAPAPKKGSKK |
| 24 | P09988 | 123 | VTIQPKDMQLA |
| 25 | P69150 | 19 | AKAPRKQLASK |
| 26 | Q55BP0 | 30 | SSFPSKPVNEV |
| 27 | Q55BP0 | 65 | DLLIKKLPFQR |
| 28 | P61830 | 126 | QKKDIKLARRL |
| 29 | P62803 | 32 | IQGITKPAIRR |
| 30 | P04637 | 164 | AMAIYKQSQHM |
| 31 | P04637 | 386 | KKLMFKTEGPD |
| 32 | Q86L05 | 43 | KNYDTKKDKRF |
| 33 | Q86L05 | 53 | FSGQIKIGTVT |
| 34 | Q86L05 | 97 | IGPKNKKAIKK |
| 35 | Q86L05 | 105 | IKKLSKKYDAF |
| 36 | Q86L05 | 147 | EDMASKINDVK |
| 37 | Q86L05 | 152 | KINDVKSTVKF |
| 38 | P14909 | 34 | VEKTKKIKIID |
| 39 | P14909 | 80 | DELREKIAQYL |
| 40 | P14909 | 94 | YGTDVKKEEVI |
| 41 | P14909 | 105 | VTPGAKPALFL |
| 42 | P14909 | 142 | KLLGGKPIYAN |
| 43 | P14909 | 194 | PNDVKKIVDIS |
| 44 | P14909 | 257 | GYIVAKREIIQ |
| 45 | P14909 | 285 | QKAAVKAFDTF |
| 46 | P14909 | 313 | YDELTKVKGVE |
| 47 | P39462 | 136 | IVPHYKYMYKL |
| 48 | P39462 | 263 | PKALAKQGKYV |
| 49 | P22498 | 76 | QKMGLKIARLN |
| 50 | P22498 | 138 | IFKDLKSRGLY |
| 51 | P22498 | 256 | KSVSKKPVGII |
| 52 | P22498 | 445 | RFGLLKVDYNT |
| 53 | P0C835 | 53 | KYFRHKLPDDY |
| 54 | P80053 | 16 | YTQQVKKLYKV |
| 55 | P80053 | 54 | RGSDGKLKTFM |
| 56 | P80053 | 170 | IKITGKVDFAV |
| 57 | P13123 | 52 | EKDAPKELLDM |
| 58 | P61991 | 40 | DEGGGKTGRGA |
| 59 | P26358 | 284 | DEKDEKKHRSQ |
| 60 | P26358 | 291 | HRSQPKDLAAK |
| 61 | O75530 | 398 | VEDPHKAKCTT |
| 62 | O75530 | 400 | DPHKAKCTTLT |
| 63 | Q5VTE0 | 371 | AHIACKFAELK |
| 64 | P81542 | 20 | DHAGHKVYAPA |
| 65 | P81542 | 32 | PPVKEKALGIH |
| 66 | P00219 | 20 | DHSGHKIYGPV |
| 67 | O43524 | 242 | NPDGGKSGKAP |
| 68 | Q08369 | 311 | PLAMRKEGIQT |
| 69 | P16403 | 110 | FKLNKKAASGE |
| 70 | P16403 | 117 | ASGEAKPKVKK |

| | | | |
|---|---|---|---|
| 71 | P16403 | 130 | GTKPKKPVGAA |
| 72 | P16403 | 139 | AAKKPKKAAGG |
| 73 | P10412 | 22 | KTPVKKKARKS |
| 74 | P10412 | 34 | GAAKRKASGPP |
| 75 | P10412 | 121 | AKPKAKKAGAA |
| 76 | P10412 | 136 | PAGAAKKPKKA |
| 77 | P10412 | 148 | GAATPKKSAKK |
| 78 | P16401 | 21 | EKSPAKKKATK |
| 79 | P16401 | 23 | SPAKKKATKKA |
| 80 | P16401 | 35 | GAGAAKRKATG |
| 81 | P16401 | 154 | KKAVKKTPKKA |
| 82 | O75367 | 157 | KKGARKSKKKQ |
| 83 | O75367 | 236 | NTLEKKGGKEF |
| 84 | P40283 | 13 | KPAEKKPASEK |
| 85 | P40283 | 33 | KAPAEKKPKAG |
| 86 | P40283 | 52 | AGGDKKKKMKK |
| 87 | Q41811 | 60 | TRGVRKIFLEN |
| 88 | O14979 | 322 | QQQQQKGGRGA |
| 89 | Q14103 | 231 | CFITFKEEEPV |
| 90 | P16862 | 76 | VEAHLKELNSI |
| 91 | P16862 | 197 | LNRPQKAIAVM |
| 92 | P16862 | 525 | AVNENKIVRKP |
| 93 | P16862 | 548 | EAIQAKDFKRA |
| 94 | O00159 | 24 | PHRPCKLALGS |
| 95 | O00159 | 274 | KGQCAKVSSIN |
| 96 | O00159 | 285 | DKSDWKVVRKA |
| 97 | O00159 | 486 | DLVEEKFKGII |
| 98 | O00159 | 488 | VEEKFKGIISI |
| 99 | O00159 | 595 | ELSDKKRPETV |
| 100 | O00159 | 605 | VATQFKMSLLQ |
| 101 | O00159 | 618 | EILQSKEPAYV |
| 102 | P13538 | 85 | SMNPPKYDKIE |
| 103 | P13538 | 207 | ASGEKKKEEQS |
| 104 | P13538 | 260 | FGATGKLASAD |
| 105 | P13538 | 354 | KTAIYKLTGAV |
| 106 | Q96RI1 | 36 | SMKPAKGVLTE |
| 107 | Q96RI1 | 196 | ECRLRKCKEMG |
| 108 | Q96RI1 | 251 | VTSTTKSCREK |
| 109 | Q96RI1 | 289 | TNKILKEEFSA |
| 110 | Q96RI1 | 335 | QIALLKGSAVE |
| 111 | Q96RI1 | 474 | RVNDHKFTPLL |
| 112 | P11940 | 30 | AMLYEKFSPAG |
| 113 | P11940 | 113 | KSIDNKALYDT |
| 114 | P11940 | 157 | ERAIEKMNGML |
| 115 | P11940 | 277 | RQTELKRKFEQ |
| 116 | P06400 | 8 | PKTPRKTAATA |
| 117 | P06400 | 80 | WLTWEKVSSVD |
| 118 | P06400 | 122 | FTELQKNIEIS |
| 119 | P06400 | 143 | IDTSTKVDNAM |
| 120 | P06400 | 164 | FALFSKLERTC |
| 121 | P06400 | 228 | LDYFIKLSPPM |
| 122 | P06400 | 327 | EEIYLKNKDLD |

| | | | |
|---|---|---|---|
| 123 | P06400 | 341 | FLDHDKTLQTD |
| 124 | P0CX43 | 91 | SVDDLKKLNKN |
| 125 | P47914 | 47 | NMRFAKKHNKK |
| 126 | P47914 | 55 | NKKGLKKMQAN |
| 127 | Q969Q0 | 6 | MVNVPKTRRTF |
| 128 | Q969Q0 | 58 | KPIFRKKAKTT |
| 129 | Q969Q0 | 59 | PIFRKKAKTTK |
| 130 | Q969Q0 | 89 | AIKRCKHFELG |
| 131 | P0CX27 | 76 | RLECVKCKTRA |
| 132 | P0CX27 | 89 | TLKRCKHFELG |
| 133 | P02393 | 91 | GLKEAKDKVDG |
| 134 | P02393 | 114 | EAEEAKKQLVE |
| 135 | P07472 | 98 | APATLKEGMSK |
| 136 | Q12962 | 212 | YGINVKKPHYF |
| 137 | Q04207 | 122 | GIQCVKKRDLE |
| 138 | Q04207 | 221 | CDKVQKEDIEV |
| 139 | Q04207 | 301 | HRIEEKRKRTY |
| 140 | Q04207 | 425 | SAPVPKSTQAG |
| 141 | Q53688 | 12 | RLQLNKNFNFG |
| 142 | E5KWG9 | 29 | TASHDKVWPIL |
| 143 | E5KWG9 | 135 | VSELVKLPSIP |
| 144 | E5KWG9 | 278 | IASTPKGEYHK |
| 145 | E5KWG9 | 283 | KGEYHKIASYY |
| 146 | E5KWG9 | 292 | YYQWQKRVEKL |
| 147 | B6A879 | 93 | TTADVKPTENI |
| 148 | B6A879 | 173 | DGGGSKEEERG |
| 149 | B6A879 | 290 | LRELQKKAHAM |
| 150 | Q9L5X8 | 126 | FAHGLKPIVVI |
| 151 | Q9L5X8 | 438 | MAPDGKGRVRM |
| 152 | Q6NC90 | 9 | HIDPTKEVFAQ |
| 153 | Q6NEZ3 | 66 | YKFNQKSNFSA |
| 154 | Q6NEZ3 | 108 | GYKNAKLTKSS |
| 155 | Q6NEZ3 | 120 | RINVGKNVKLD |
| 156 | Q6NEZ3 | 178 | TFIPEKTILTN |
| 157 | Q6NEZ3 | 341 | VMPGEKVSVSA |
| 158 | Q9UKV3 | 103 | LVKRLKGALML |
| 159 | P18281 | 16 | QGAALKHAETV |
| 160 | P18281 | 34 | ENVTVKKVDRS |
| 161 | P18281 | 52 | KPHELKHAETV |
| 162 | Q9SJU4 | 246 | YDVAEKVWAEV |
| 163 | Q9ZU52 | 120 | LYQSTKDGKTF |
| 164 | Q9ZU52 | 123 | STKDGKTFVDC |
| 165 | Q9ZU52 | 142 | GIKVDKGLSPL |
| 166 | Q9ZU52 | 171 | SAEYYKQGARF |
| 167 | P62157 | 22 | FSLFDKDGDGT |
| 168 | P11118 | 76 | TLMSRKMHDTD |
| 169 | P07463 | 76 | SLMARKMKEQD |
| 170 | P04573 | 43 | MDMLTKLGQTY |
| 171 | P04573 | 133 | VEEAMKEADED |
| 172 | P00889 | 208 | MDLIAKLPCVA |
| 173 | P00063 | 16 | PAAGEKIFKTK |
| 174 | P00063 | 107 | LIAYLKQSTAA |

| | | | |
|---|---|---|---|
| 175 | P00078 | 19 | AARGEKLFKGR |
| 176 | P00077 | 19 | AAKGEKIFKGR |
| 177 | P00077 | 22 | GEKIFKGRAAQ |
| 178 | P00077 | 98 | FAGIKKPQERA |
| 179 | P00043 | 60 | YTDANKKKGVE |
| 180 | P00076 | 7 | DAERGKKLFES |
| 181 | P00076 | 65 | EETLHKFLENP |
| 182 | P00076 | 78 | YVPGTKMAFAG |
| 183 | P00047 | 13 | PGDASKGANLF |
| 184 | P00047 | 97 | GLKKNKDRNDL |
| 185 | Q71V39 | 179 | VSAYIKKIGYN |
| 186 | P02993 | 172 | FEEIKKEVSAY |
| 187 | P02994 | 177 | TSNFIKKVGYN |
| 188 | P02994 | 217 | WEKETKAGVVK |
| 189 | Q96KQ7 | 139 | MTGAGKSPPSV |
| 190 | P53991 | 49 | TTDMSKRTVPT |
| 191 | P53991 | 55 | RTVPTKLEEGE |
| 192 | P53991 | 69 | NTYSNKAPFKA |
| 193 | P53991 | 81 | VRSVEKITGPK |
| 194 | P53991 | 164 | DPETGKEDPAK |
| 195 | P11416 | 94 | FVCQDKSSGYH |
| 196 | P11416 | 166 | DRNKKKKEAPK |
| 197 | P11416 | 360 | LLEALKVYVRK |
| 198 | P27064 | 227 | AEAIFKSQAET |
| 199 | P0A7J7 | 92 | KSGSGKPNKDK |
| 200 | Q5SLP6 | 9 | VVAVVKLQLPA |
| 201 | Q5SLP6 | 16 | QLPAGKATPAP |
| 202 | Q5SLP6 | 70 | FTFVTKTPPAS |
| 203 | P0CX53 | 41 | GLSPKKVGEDI |
| 204 | P62986 | 114 | NCRKKKCGHTN |
| 205 | P55072 | 20 | AILKQKNRPNR |
| 206 | P55072 | 190 | EGEPIKREDEE |
| 207 | P55072 | 211 | IGGCRKQLAQI |
| 208 | P55072 | 231 | HPALFKAIGVK |
| 209 | P05547 | 21 | DIDRKKAEVRK |
| 210 | P05547 | 26 | KAEVRKRLEEQ |
| 211 | P05547 | 46 | MTPERKKKLRL |
| 212 | O95785 | 54 | YLPVTKEGPRD |
| 213 | O95785 | 128 | PRLLEKHAQGR |
| 214 | O95785 | 277 | CSIYFKQKEHL |
| 215 | O95785 | 517 | DFPLSKPLLHG |
| 216 | O95785 | 702 | KHEERKCPYCP |
| 217 | O95785 | 754 | FSFQKKKKKVA |
| 218 | P05141 | 10 | AVSFAKDFLAG |
| 219 | P26358 | 160 | PRITRKSTRQT |
| 220 | P26358 | 179 | GPAKRKPQEES |
| 221 | P26358 | 203 | KDQDEKRRRVT |
| 222 | O75530 | 89 | CVNSLKEDHNQ |
| 223 | O75530 | 184 | TMQCIKHYVGH |
| 224 | O75530 | 293 | PFISQKIHFPD |
| 225 | O75530 | 322 | DLILSKSCENA |
| 226 | Q5VTE0 | 146 | YTLGVKQLIVG |

| | | | |
|---|---|---|---|
| 227 | Q5VTE0 | 179 | VSTYIKKIGYN |
| 228 | Q5VTE0 | 215 | WFKGWKVTRKD |
| 229 | Q5VTE0 | 255 | LQDVYKIGGIG |
| 230 | P0CE47 | 249 | EIVGIKETQKS |
| 231 | P0CE47 | 300 | KPGTIKPHTKF |
| 232 | P0CE47 | 304 | IKPHTKFESEV |
| 233 | P0CE47 | 314 | VYILSKDEGGR |
| 234 | O75367 | 8 | SRGGKKKSTKT |
| 235 | P62807 | 13 | APAPKKGSKKA |
| 236 | P62807 | 24 | VTKAQKKDGKK |
| 237 | P62807 | 29 | KKDGKKRKRSR |
| 238 | P62807 | 31 | DGKKRKRSRKE |
| 239 | P62807 | 86 | LAHYNKRSTIT |
| 240 | Q55BN9 | 64 | SELLIKKLPFQ |
| 241 | Q55BP0 | 64 | SDLLIKKLPFQ |
| 242 | P62803 | 80 | EHAKRKTVTAM |
| 243 | Q86L05 | 90 | DIEALKKIGPK |
| 244 | Q86L05 | 95 | KKIGPKNKKAI |
| 245 | Q7M1B9 | 18 | AEKVLKSKTPV |
| 246 | P14909 | 36 | KTKKIKIIDFG |
| 247 | P14909 | 263 | REIIQKMGILA |
| 248 | P14909 | 301 | MVSLFKKRRDV |
| 249 | P14909 | 315 | ELTKVKGVEVS |
| 250 | P14909 | 321 | GVEVSKPNGAF |
| 251 | P22498 | 72 | HDNAQKMGLKI |
| 252 | P22498 | 102 | NFDESKQDVTE |
| 253 | Q9Y232 | 204 | PKSPVKSRTAV |
| 254 | Q9Y232 | 348 | DIVVRKQDGFT |
| 255 | Q9Y232 | 360 | ILLSTKSSENN |
| 256 | P80053 | 20 | VKKLYKVGELL |
| 257 | P80053 | 97 | MIMTWKNSLLL |
| 258 | P80053 | 109 | PYGGGKGGVRV |
| 259 | P80053 | 137 | IQAIYKYLGSE |
| 260 | P80053 | 313 | PKVKAKLIVEG |
| 261 | P61991 | 21 | DISKIKKVWRV |
| 262 | P26358 | 302 | RRPEEKEPEKV |
| 263 | O75530 | 335 | CWKPGKMEDDI |
| 264 | O75530 | 344 | DIDKIKPSESN |
| 265 | Q5VTE0 | 376 | KFAELKEKIDR |
| 266 | P55907 | 74 | HPASEKKADPV |
| 267 | P81543 | 20 | THEGHKVYGPV |
| 268 | P49949 | 20 | EHQGHKVYGPV |
| 269 | O43524 | 195 | CVPYFKDKGDS |
| 270 | Q08369 | 328 | NLNKSKTPAGP |
| 271 | Q08369 | 402 | SAVCSKLSPQG |
| 272 | P10412 | 17 | PAPAEKTPVKK |
| 273 | P10412 | 127 | KAGAAKAKKPA |
| 274 | P16401 | 22 | KSPAKKKATKK |
| 275 | P16401 | 26 | KKKATKKAAGA |
| 276 | P16401 | 37 | GAAKRKATGPP |
| 277 | P16401 | 143 | TPKKAKKAAGA |
| 278 | P16401 | 150 | AAGAKKAVKKT |

| | | | |
|---|---|---|---|
| 279 | O75367 | 235 | GNTLEKKGGKE |
| 280 | P40283 | 43 | GKKLPKEAGAG |
| 281 | P40283 | 111 | LARYNKKPTIT |
| 282 | Q41811 | 17 | GKGARKRHRKV |
| 283 | O14979 | 76 | GGAAIKGGRRR |
| 284 | O14979 | 142 | KINASKNQQDD |
| 285 | O14979 | 149 | QQDDGKMFIGG |
| 286 | Q14103 | 67 | ESEGAKIDASK |
| 287 | Q14103 | 111 | WDTTKKDLKDY |
| 288 | Q14103 | 129 | VDCTLKLDPIT |
| 289 | Q14103 | 146 | GFVLFKESESV |
| 290 | Q14103 | 176 | RAKAMKTKEPV |
| 291 | Q14103 | 178 | KAMKTKEPVKK |
| 292 | Q14103 | 255 | SKCEIKVAMSK |
| 293 | P16862 | 69 | DEKINKNVEAH |
| 294 | P16862 | 323 | IEELLKTNRIS |
| 295 | P16862 | 336 | QYERMKHLNIC |
| 296 | P16862 | 536 | LMESVKLTKAV |
| 297 | P16862 | 539 | SVKLTKAVAEA |
| 298 | P16862 | 588 | KDKRLKIAIVN |
| 299 | O00159 | 527 | HFLTHKLADQR |
| 300 | P13538 | 370 | LKFKQKQREEQ |
| 301 | Q96RI1 | 132 | SAGRIKGDELC |
| 302 | Q96RI1 | 172 | KNAVYKCKNGG |
| 303 | Q96RI1 | 353 | EIFNKKLPSGH |
| 304 | Q96RI1 | 434 | LQKLCKIHQPE |
| 305 | P11940 | 104 | GNIFIKNLDKS |
| 306 | P11940 | 213 | KDLFGKFGPAL |
| 307 | P11940 | 254 | DEMNGKELNGK |
| 308 | P06400 | 65 | LCQKLKIPDHV |
| 309 | P06400 | 130 | EISVHKFFNLL |
| 310 | P06400 | 192 | SALVLKVSWIT |
| 311 | P06400 | 279 | IEVLCKEHECN |
| 312 | P06400 | 319 | VENLSKRYEEI |
| 313 | P06400 | 427 | IGYIFKEKFAK |
| 314 | P06400 | 429 | YIFKEKFAKAV |
| 315 | Q86L05 | 156 | VKSTVKFQLKK |
| 316 | Q86L05 | 207 | KTLYVKTSMGP |
| 317 | P0CX43 | 39 | LQVGLKNYDPQ |
| 318 | P0CX43 | 97 | KLNKNKKLIKK |
| 319 | P0CX43 | 98 | LNKNKKLIKKL |
| 320 | P0CX43 | 102 | KKLIKKLSKKY |
| 321 | P47914 | 22 | HRNGIKKPRSQ |
| 322 | P47914 | 23 | RNGIKKPRSQR |
| 323 | P47914 | 48 | MRFAKKHNKKG |
| 324 | P47914 | 92 | KGVSRKLDRLA |
| 325 | P47914 | 124 | CRPKAKAKAKA |
| 326 | P47914 | 149 | PAQAPKRTQAP |
| 327 | Q969Q0 | 44 | RRYDRKQSGYG |
| 328 | Q969Q0 | 100 | GDKKRKGQVIQ |
| 329 | P0CX27 | 78 | ECVKCKTRAQL |
| 330 | P02393 | 6 | MSSITKEQVVE |

| | | | |
|---|---|---|---|
| 331 | P02393 | 72 | AAGANKIGVIK |
| 332 | P0A7K2 | 30 | SAMEEKFGVSA |
| 333 | P0A7K2 | 109 | AEALKKALEEA |
| 334 | P07472 | 110 | DGDEAKTKLEE |
| 335 | Q12962 | 42 | TAAENKASPAG |
| 336 | Q12962 | 213 | GINVKKPHYFT |
| 337 | Q04207 | 56 | STDTTKTHPTI |
| 338 | Q04207 | 93 | HELVGKDCRDG |
| 339 | Q53688 | 185 | TLMSWKNPPSY |
| 340 | Q53688 | 417 | DLRYYKISPDQ |
| 341 | E5KWG9 | 296 | QKRVEKLLRSD |
| 342 | B6A879 | 11 | TYTSSKAMSDI |
| 343 | B6A879 | 83 | ADEINKIGNPT |
| 344 | B6A879 | 374 | YVLLIKELRSA |
| 345 | B6A879 | 552 | LFSEAKGHFIS |
| 346 | Q9L5X8 | 119 | RFVTQKAFAHG |
| 347 | Q9L5X8 | 287 | GLGELKISDTI |
| 348 | Q9L5X8 | 341 | LERLEKELVHN |
| 349 | D0ZRV8 | 13 | LIELVKKVNRN |
| 350 | D0ZRV8 | 194 | WKAQQKMNEQR |
| 351 | Q6NEZ3 | 80 | MPESVKAGTGF |
| 352 | Q6NEZ3 | 105 | NLSGYKNAKLT |
| 353 | Q6NEZ3 | 111 | NAKLTKSSIRI |
| 354 | Q6NEZ3 | 202 | TTNADKPFATV |
| 355 | Q6NEZ3 | 369 | APEDVKYIDGT |
| 356 | Q6NEZ3 | 407 | WKNESKKPNES |
| 357 | Q6NEZ3 | 461 | YSNSLKDLVSI |
| 358 | D1BQI7 | 38 | YGRQLKTRIPP |
| 359 | Q9UKV3 | 143 | KQYLEKQQELL |
| 360 | Q9UKV3 | 223 | DEKPRKGERRS |
| 361 | Q9UKV3 | 315 | EIPRVKPEEMM |
| 362 | P18281 | 59 | AETVDKSGPAI |
| 363 | Q9SJU4 | 15 | KASPVKSDWVK |
| 364 | Q9SJU4 | 20 | KSDWVKGQSLL |
| 365 | Q9SJU4 | 131 | STTDGKKMVDV |
| 366 | Q9SJU4 | 132 | TTDGKKMVDVL |
| 367 | Q9SJU4 | 348 | QNTCLKTWGGK |
| 368 | Q9ZU52 | 271 | PGAEHKNKASP |
| 369 | Q9ZU52 | 348 | QGKPEKIEASQ |
| 370 | P62157 | 14 | QIAEFKEAFSL |
| 371 | P07463 | 14 | QIAEFKEAFAL |
| 372 | P07463 | 22 | FALFDKDGDGT |
| 373 | P07463 | 78 | MARKMKEQDSE |
| 374 | P04573 | 67 | GPKGDKKNIGP |
| 375 | P04573 | 68 | PKGDKKNIGPE |
| 376 | P04573 | 112 | DFDEFKFIMQK |
| 377 | P00889 | 14 | RLFGAKNASCL |
| 378 | P00889 | 34 | SSTNLKDILAD |
| 379 | P00889 | 233 | GAIDSKLDWSH |
| 380 | P00889 | 317 | LTQLQKEVGKD |
| 381 | P00889 | 327 | DVSDEKLRDYI |
| 382 | P00889 | 366 | REFALKHLPHD |

| | | | |
|---|---|---|---|
| 383 | P00889 | 393 | LLEQGKAKNPW |
| 384 | P00063 | 21 | KIFKTKCAQCH |
| 385 | P00078 | 61 | GYAYSKANAES |
| 386 | P00078 | 97 | SFAGMKKPQER |
| 387 | P00077 | 33 | CHTGAKGGANG |
| 388 | P00077 | 97 | SFAGIKKPQER |
| 389 | P00043 | 7 | PAPYEKGSEKK |
| 390 | P00076 | 8 | AERGKKLFESR |
| 391 | P00076 | 72 | LENPKKYVPGT |
| 392 | P00076 | 88 | GIKAKKDRQDI |
| 393 | P00047 | 87 | FIPGTKMAFGG |
| 394 | P02993 | 244 | SRPTEKPLRLP |
| 395 | P02994 | 129 | EAGISKDGQTR |
| 396 | P02994 | 154 | IVAVNKMDSVK |
| 397 | P02994 | 170 | FQEIVKETSNF |
| 398 | P02994 | 184 | VGYNPKTVPFV |
| 399 | Q96KQ7 | 179 | PEGQPKVHRAR |
| 400 | Q96KQ7 | 253 | EVTLTKGDPGS |
| 401 | Q96KQ7 | 288 | VDSDSKSEVEA |
| 402 | Q96KQ7 | 344 | RRKAKKKWRKD |
| 403 | P53991 | 101 | IETEGKIPFWE |
| 404 | P53991 | 282 | EQNNRKGGKMY |
| 405 | P10587 | 33 | DWSAKKLVWVP |
| 406 | P10587 | 67 | QENGKKVTLSK |
| 407 | P10587 | 82 | KMNPPKFSKVE |
| 408 | P10587 | 183 | ESGAGKTENTK |
| 409 | P10587 | 189 | TENTKKVIQYL |
| 410 | P11416 | 109 | ACEGCKGFFRR |
| 411 | P11416 | 128 | TCHRDKNCIIN |
| 412 | P11416 | 134 | NCIINKVTRNR |
| 413 | P11416 | 171 | KKEAPKPECSE |
| 414 | P11416 | 227 | IDLWDKFSELS |
| 415 | P11416 | 234 | SELSTKCIIKT |
| 416 | P27064 | 164 | RDKLNKYGRPL |
| 417 | P27064 | 316 | FRVLAKALRMS |
| 418 | P27064 | 466 | VWKEIKFEFEA |
| 419 | P04717 | 252 | CEEMLKRAVFA |
| 420 | P04717 | 356 | DDYIKKDRSRG |
| 421 | P04992 | 21 | GVKEYKLTYYT |
| 422 | P04992 | 252 | CEEMMKRAVFA |
| 423 | P04992 | 463 | ACEVWKEIRFN |
| 424 | Q5SLP6 | 39 | IMEFVKAFNAA |
| 425 | Q5SLP6 | 109 | VLEIAKQKMPD |
| 426 | P0CX53 | 96 | DRKKDKNVKHS |
| 427 | P0CX53 | 99 | KDKNVKHSGNI |
| 428 | P62986 | 27 | TIENVKAKIQD |
| 429 | P62986 | 29 | ENVKAKIQDKE |
| 430 | P62986 | 112 | AVNCRKKKCGH |
| 431 | P55072 | 136 | FEVYLKPYFLE |
| 432 | O95785 | 105 | IVRTMKPHAEL |
| 433 | O95785 | 373 | YVQHAKLHMRE |
| 434 | O95785 | 479 | VDYFGKAEPSL |

| | | | |
|---|---|---|---|
| 435 | P05141 | 33 | PIERVKLLLQV |
| 436 | P05141 | 92 | LNFAFKDKYKQ |
| 437 | P05141 | 166 | LVKIYKSDGIK |
| 438 | Q9Y232 | 17 | GKSRKKNWQYE |
| 439 | P00042 | 6 | PAPFKKGSEKK |
| 440 | P00042 | 28 | CHTVEKGGPHK |
| 441 | P00042 | 95 | GLKKEKDRNDL |
| 442 | P26358 | 29 | VRRRLKDLERD |
| 443 | P26358 | 45 | ECVKEKLNLLH |
| 444 | P26358 | 67 | CDLETKLRKEE |
| 445 | P26358 | 81 | EGYLAKVKSLL |
| 446 | P26358 | 130 | ANSPPKPLSKP |
| 447 | P26358 | 148 | SDGEAKPEPSP |
| 448 | O75530 | 70 | RKSWGKGKWKS |
| 449 | O75530 | 72 | SWGKGKWKSKK |
| 450 | O75530 | 77 | KWKSKKCKYSF |
| 451 | O75530 | 250 | DLLGEKIMSCG |
| 452 | Q5VTE0 | 20 | HVDSGKSTTTG |
| 453 | Q5VTE0 | 51 | AAEMGKGSFKY |
| 454 | Q5VTE0 | 84 | KFETSKYYVTI |
| 455 | Q5VTE0 | 154 | IVGVNKMDSTE |
| 456 | Q5VTE0 | 172 | YEEIVKEVSTY |
| 457 | P0CE47 | 137 | IVFLNKCDMVD |
| 458 | P0CE47 | 283 | LLRGIKREEIE |
| 459 | P0CE47 | 325 | HTPFFKGYRPQ |
| 460 | O75367 | 96 | LNQLLKGVTIA |
| 461 | O75367 | 142 | KSPSQKKPVSK |
| 462 | O75367 | 143 | SPSQKKPVSKK |
| 463 | O75367 | 147 | KKPVSKKAGGK |
| 464 | O75367 | 152 | KKAGGKKGARK |
| 465 | O75367 | 153 | KAGGKKGARKS |
| 466 | P62807 | 16 | PKKGSKKAVTK |
| 467 | P62807 | 17 | KKGSKKAVTKA |
| 468 | O15819 | 126 | VTIMPKDIHLA |
| 469 | Q55BN9 | 123 | VTIMVKDIQLA |
| 470 | P61830 | 38 | TGGVKKPHRYK |
| 471 | P04637 | 24 | FSDLWKLLPEN |
| 472 | P04637 | 139 | FCQLAKTCPVQ |
| 473 | Q86L05 | 46 | DTKKDKRFSGQ |
| 474 | Q86L05 | 78 | CDEATKIGAEF |
| 475 | P80322 | 37 | GDISTKELGTI |
| 476 | P14909 | 31 | ARNVEKTKKIK |
| 477 | P14909 | 137 | YAEVVKLLGGK |
| 478 | P39462 | 313 | LAEAGKVKPMI |
| 479 | Q9Y232 | 312 | GVTASKRKFID |
| 480 | Q9Y232 | 314 | TASKRKFIDDR |
| 481 | P0C835 | 6 | MSSGKKAVKVK |
| 482 | P0C835 | 48 | DPETGKYFRHK |
| 483 | P80053 | 45 | RIIQVKIQIRG |
| 484 | P80053 | 231 | GYYAGKFLSEM |
| 485 | P80053 | 265 | KAIEIKEKTGS |
| 486 | P13123 | 22 | TSKIKKVWRVG |

| | | | |
|---|---|---|---|
| 487 | P61991 | 13 | YKGEEKQVDIS |
| 488 | P61991 | 53 | EKDAPKELLQM |
| 489 | P39476 | 13 | YKGEEKEVDIS |
| 490 | P26358 | 240 | EERDEKEEKRL |
| 491 | P26358 | 243 | DEKEEKRLRSQ |
| 492 | P26358 | 255 | KEPTPKQKLKE |
| 493 | P26358 | 259 | PKQKLKEEPDR |
| 494 | P26358 | 281 | EDGDEKDEKKH |
| 495 | P26358 | 285 | EKDEKKHRSQP |
| 496 | P26358 | 296 | KDLAAKRRPEE |
| 497 | Q5VTE0 | 273 | ETGVLKPGMVV |
| 498 | P81539 | 13 | SLPVVKEEQGV |
| 499 | P81539 | 19 | EEQGVKIYGTY |
| 500 | P49949 | 73 | HPASEKKADPI |
| 501 | O43524 | 270 | GRAAKKKAALQ |
| 502 | O43524 | 455 | TIQENKPATFS |
| 503 | O43524 | 569 | SLGSAKHQQQS |
| 504 | Q08369 | 320 | QTRKRKPKNLN |
| 505 | Q08369 | 326 | PKNLNKSKTPA |
| 506 | P16403 | 26 | KKKAAKKAGGT |
| 507 | P16403 | 136 | PVGAAKKPKKA |
| 508 | P16403 | 152 | PKKSAKKTPKK |
| 509 | P16403 | 156 | AKKTPKKAKKP |
| 510 | P10412 | 32 | SAGAAKRKASG |
| 511 | P10412 | 139 | AAKKPKKATGA |
| 512 | P16401 | 67 | LAALKKALAAG |
| 513 | P16401 | 141 | GATPKKAKKAA |
| 514 | O75367 | 160 | ARKSKKKQGEV |
| 515 | O75367 | 196 | LFLGQKLNLIH |
| 516 | O75367 | 250 | VLELRKKNGPL |
| 517 | P40283 | 40 | PKAGKKLPKEA |
| 518 | P40283 | 58 | KKMKKKSVETY |
| 519 | P40283 | 98 | NDIFEKLAQEA |
| 520 | O14979 | 137 | FAEGSKINASK |
| 521 | O14979 | 212 | ELKEHKLDGKL |
| 522 | O14979 | 224 | DPKRAKALKGK |
| 523 | Q14103 | 242 | KKIMEKKYHNV |
| 524 | P16862 | 33 | IDFYTKFLSLE |
| 525 | P16862 | 106 | DILAVKDTLNA |
| 526 | P16862 | 144 | GVTSTKNAVST |
| 527 | P16862 | 276 | CMEFKKREGRL |
| 528 | P16862 | 551 | QAKDFKRAMSL |
| 529 | O00159 | 289 | WKVVRKALTVI |
| 530 | O00159 | 444 | NYCNEKLQQLF |
| 531 | O00159 | 627 | YVRCIKPNDAK |
| 532 | P13538 | 50 | KESFVKGTIQS |
| 533 | P13538 | 56 | GTIQSKEGGKV |
| 534 | P13538 | 206 | AASGEKKKEEQ |
| 535 | P13538 | 273 | TYLLEKSRVTF |
| 536 | P13538 | 366 | HYGNLKFKQKQ |
| 537 | Q96RI1 | 158 | TCEGCKGFFRR |
| 538 | Q96RI1 | 174 | AVYKCKNGGNC |

| | | | |
|---|---|---|---|
| 539 | Q96RI1 | 224 | SKRLRKNVKQH |
| 540 | Q96RI1 | 384 | MFSFYKSIGEL |
| 541 | Q96RI1 | 390 | SIGELKMTQEE |
| 542 | P11940 | 80 | DVIKGKPVRIM |
| 543 | P11940 | 129 | NILSCKVVCDE |
| 544 | P11940 | 229 | TDESGKSKGFG |
| 545 | P11940 | 231 | ESGKSKGFGFV |
| 546 | P11940 | 333 | EGGRSKGFGFV |
| 547 | P11940 | 348 | PEEATKAVTEM |
| 548 | P06400 | 154 | SRLLKKYDVLF |
| 549 | P06400 | 447 | GSQRYKLGVRL |
| 550 | P0CX43 | 18 | VKELLKYSNET |
| 551 | P0CX43 | 105 | IKKLSKKYNAF |
| 552 | P47914 | 15 | HNQSRKWHRNG |
| 553 | P47914 | 63 | QANNAKAMSAR |
| 554 | P47914 | 73 | RAEAIKALVKP |
| 555 | P47914 | 126 | PKAKAKAKAKD |
| 556 | P47914 | 130 | AKAKAKDQTKA |
| 557 | Q969Q0 | 13 | RRTFCKKCGKH |
| 558 | Q969Q0 | 14 | RTFCKKCGKHQ |
| 559 | Q969Q0 | 22 | KHQPHKVTQYK |
| 560 | Q969Q0 | 27 | KVTQYKKGKDS |
| 561 | Q969Q0 | 97 | ELGGDKKRKGQ |
| 562 | P0CX27 | 6 | MVNVPKTRKTY |
| 563 | P0CX27 | 9 | VPKTRKTYCKG |
| 564 | P0CX27 | 13 | RKTYCKGKTCR |
| 565 | P0CX27 | 29 | KVTQYKAGKAS |
| 566 | P02393 | 31 | KELEEKFGVSA |
| 567 | P02393 | 102 | APSTLKEAVSK |
| 568 | P0A7K2 | 66 | AAGANKVAVIK |
| 569 | P0A7K2 | 96 | APAALKEGVSK |
| 570 | Q04207 | 62 | THPTIKINGYT |
| 571 | Q53688 | 216 | QESHSKILDLD |
| 572 | Q53688 | 250 | SIIKNKIIIVE |
| 573 | Q53688 | 302 | NFTAEKISISE |
| 574 | Q53688 | 429 | HVFNQKRRGKI |
| 575 | E5KWG9 | 16 | YSIIEKTAQVV |
| 576 | E5KWG9 | 84 | HGLTPKTDHPV |
| 577 | E5KWG9 | 258 | FERLIKSAPYD |
| 578 | B6A879 | 148 | HYVGDKTKPKL |
| 579 | B6A879 | 152 | DKTKPKLSAYI |
| 580 | Q9L5X8 | 54 | SNDIEKERGIT |
| 581 | Q9L5X8 | 63 | ITILAKNTAIN |
| 582 | Q9L5X8 | 133 | IVVINKIDRPG |
| 583 | Q9L5X8 | 254 | KTRNGKVGTVM |
| 584 | Q9L5X8 | 427 | NIGLRKGELKD |
| 585 | Q9L5X8 | 545 | NALKGKQLTNV |
| 586 | D0ZRV8 | 14 | IELVKKVNRNK |
| 587 | Q6NEZ3 | 92 | ALIKIKDISVS |
| 588 | Q6NEZ3 | 208 | PFATVKVDPAD |
| 589 | Q6NEZ3 | 238 | ATVPEKLNEKM |
| 590 | Q6NEZ3 | 242 | EKLNEKMDGKV |

| | | | |
|---|---|---|---|
| 591 | Q6NEZ3 | 311 | TELDTKKPEDT |
| 592 | Q6NEZ3 | 403 | YDPEWKNESKK |
| 593 | Q9UKV3 | 58 | PSTSRKMAELE |
| 594 | Q9UKV3 | 70 | VTLDGKPLQAL |
| 595 | Q9UKV3 | 91 | QRGLAKSGQKS |
| 596 | P18281 | 71 | EDVHVKKVDRG |
| 597 | Q9SJU4 | 10 | TATMLKASPVK |
| 598 | Q9SJU4 | 58 | ADELVKTAKTI |
| 599 | Q9SJU4 | 150 | GIKVDKGLVPL |
| 600 | Q9SJU4 | 378 | LAQLGKYTGEG |
| 601 | Q9ZU52 | 238 | LEVAEKVWSEV |
| 602 | P11118 | 144 | YEEFVKMMMSK |
| 603 | P04573 | 81 | LTLCSKWVRQD |
| 604 | P04573 | 153 | FMDLIKKSKNA |
| 605 | P00044 | 11 | AGSAKKGATLF |
| 606 | P00063 | 35 | KGAGHKQGPNL |
| 607 | P00043 | 11 | EKGSEKKGANL |
| 608 | P00070 | 17 | PTTGEKIFKTK |
| 609 | P02993 | 84 | KFETAKYYVTI |
| 610 | P02993 | 171 | RFEEIKKEVSA |
| 611 | P02994 | 44 | IEKFEKEAAEL |
| 612 | P02994 | 178 | SNFIKKVGYNP |
| 613 | Q96KQ7 | 53 | EETLPKATPDS |
| 614 | Q96KQ7 | 122 | PSSPSKGGSCP |
| 615 | Q96KQ7 | 229 | LAKRRKLNSGG |
| 616 | Q96KQ7 | 348 | KKKWRKDSPWV |
| 617 | Q96KQ7 | 354 | DSPWVKPSRKR |
| 618 | Q96KQ7 | 361 | SRKRRKREPPR |
| 619 | P53991 | 73 | NKAPFKAKVRS |
| 620 | P53991 | 251 | GNSDAKLYDEE |
| 621 | P53991 | 262 | LQAIAKAYPGQ |
| 622 | P10587 | 85 | PPKFSKVEDMA |
| 623 | P10587 | 142 | IIDMYKGKKRH |
| 624 | P68696 | 81 | RSIYGKKGSAG |
| 625 | P68696 | 82 | SIYGKKGSAGV |
| 626 | P68696 | 94 | TVKTSKSILVG |
| 627 | P68696 | 116 | ANVVEKLADYL |
| 628 | P11416 | 155 | DVGMSKESVRN |
| 629 | P11416 | 207 | LCQLGKYTTNN |
| 630 | P27064 | 146 | PTAYIKTFQGP |
| 631 | P27064 | 463 | ACEVWKEIKFE |
| 632 | P04717 | 146 | PYAYVKTFQGP |
| 633 | P0A7J7 | 81 | AAVLLKKAAGI |
| 634 | P0A7J7 | 97 | KPNKDKVGKIS |
| 635 | P0A7J7 | 113 | EIAQTKAADMT |
| 636 | Q5SLP6 | 86 | AAGLEKGAHKP |
| 637 | P0CX53 | 48 | GEDIAKATKEF |
| 638 | P0CX53 | 57 | EFKGIKVTVQL |
| 639 | P62986 | 11 | KTLTGKTITLE |
| 640 | P62986 | 48 | LIFAGKQLEDG |
| 641 | P62986 | 93 | KYNCDKMICRK |
| 642 | P55072 | 8 | SGADSKGDDLS |

| | | | |
|---|---|---|---|
| 643 | P55072 | 18 | STAILKQKNRP |
| 644 | P55072 | 45 | SLSQPKMDELQ |
| 645 | P55072 | 60 | DTVLLKGKKRR |
| 646 | P55072 | 62 | VLLKGKKRREA |
| 647 | P55072 | 312 | DAIAPKREKTH |
| 648 | P05547 | 11 | AAEEAKKKQDD |
| 649 | P05547 | 34 | EEQSLKKQKKG |
| 650 | P05547 | 63 | AEELKKEQERK |
| 651 | O95785 | 339 | IEEIQKLKQVP |
| 652 | P05141 | 43 | VQHASKQITAD |
| 653 | P05141 | 163 | GDCLVKIYKSD |
| 654 | P05141 | 171 | KSDGIKGLYQG |
| 655 | P05141 | 206 | MLPDPKNTHIV |
| 656 | Q9Y232 | 116 | RRHTEKQKEST |
| 657 | P00042 | 33 | KGGPHKVGPNL |
| 658 | P26358 | 188 | ESERAKSDESI |
| 659 | P26358 | 233 | GTRTEKEEERD |
| 660 | Q5VTE0 | 100 | HRDFIKNMITG |
| 661 | P0CE47 | 38 | TTVLAKTYGGA |
| 662 | P0CE47 | 253 | IKETQKSTCTG |
| 663 | O75367 | 7 | SSRGGKKKSTK |
| 664 | O75367 | 121 | KKRGSKGKLEA |
| 665 | O75367 | 137 | PAKKAKSPSQK |
| 666 | P69150 | 54 | LREIRKYQKST |
| 667 | P69150 | 123 | VTIMTKDMQLA |
| 668 | P59226 | 57 | IRKYQKSTELL |
| 669 | P62803 | 60 | TRGVLKVFLEN |
| 670 | P62803 | 78 | YTEHAKRKTVT |
| 671 | P62803 | 92 | VVYALKRQGRT |
| 672 | P04637 | 319 | SSPQPKKKPLD |
| 673 | P04637 | 320 | SPQPKKKPLDG |
| 674 | P04637 | 357 | DAQAGKEPGGS |
| 675 | Q86L05 | 61 | TVTKPKLSVCV |
| 676 | Q86L05 | 133 | LNKVGKFPTLL |
| 677 | Q7M1B9 | 57 | RLTIAKVNTDD |
| 678 | P80322 | 6 | SDDYVKARVMF |
| 679 | P80322 | 12 | ARVMFKEEQIS |
| 680 | P14909 | 51 | DLPTFKRIRDA |
| 681 | P14909 | 58 | IRDAAKEALDQ |
| 682 | P14909 | 169 | QSKISKRTKMI |
| 683 | P14909 | 242 | VNGFSKTFSMT |
| 684 | P14909 | 354 | KLIEEKGVVTI |
| 685 | P39462 | 22 | EIGVPKPKGPQ |
| 686 | P39462 | 73 | HEIAGKIEEVG |
| 687 | P39462 | 239 | RITESKGVDAV |
| 688 | P39462 | 259 | LSVYPKALAKQ |
| 689 | P39462 | 315 | EAGKVKPMITK |
| 690 | P39462 | 338 | NLENFKAIGRQ |
| 691 | P22498 | 35 | NTDWYKWVHDP |
| 692 | P22498 | 251 | AYDGIKSVSKK |
| 693 | P0C835 | 9 | GKKAVKVKTPA |
| 694 | P80053 | 17 | TQQVKKLYKVG |

| | | | |
|---|---|---|---|
| 695 | P80053 | 207 | KEAANKFIGGV |
| 696 | P80053 | 304 | ENVINKFNAPK |
| 697 | P80053 | 415 | VVRAMKARGIL |
| 698 | P13123 | 13 | YKGEEKEVDTS |
| 699 | P13123 | 21 | DTSKIKKVWRV |
| 700 | P13123 | 39 | YDDNGKTGRGA |
| 701 | P13123 | 48 | GAVSEKDAPKE |
| 702 | P26358 | 250 | LRSQTKEPTPK |
| 703 | O75530 | 375 | MDFWQKMLALG |
| 704 | Q5VTE0 | 392 | LEDGPKFLKSG |
| 705 | P55907 | 75 | PASEKKADPVN |
| 706 | O43524 | 259 | MDNSNKYTKSR |
| 707 | Q08369 | 318 | GIQTRKRKPKN |
| 708 | P16403 | 21 | EKAPVKKKAAK |
| 709 | P16403 | 63 | SLAALKKALAA |
| 710 | P16403 | 97 | TLVQTKGTGAS |
| 711 | P16403 | 122 | KPKVKKAGGTK |
| 712 | P16403 | 127 | KAGGTKPKKPV |
| 713 | P16403 | 129 | GGTKPKKPVGA |
| 714 | P10412 | 23 | TPVKKKARKSA |
| 715 | P10412 | 129 | GAAKAKKPAGA |
| 716 | P16401 | 133 | AAKAKKPAGAT |
| 717 | O75367 | 226 | ADIDLKDDLGN |
| 718 | O75367 | 239 | EKKGGKEFVEA |
| 719 | P40283 | 36 | AEKKPKAGKKL |
| 720 | P40283 | 57 | KKKMKKKSVET |
| 721 | P40283 | 69 | KIYIFKVLKQV |
| 722 | P40283 | 142 | VSEGTKAVTKF |
| 723 | Q41811 | 80 | EHARRKTVTAM |
| 724 | O14979 | 209 | KVLELKEHKLD |
| 725 | O14979 | 250 | SEEQIKEYFGA |
| 726 | O14979 | 269 | LPMDTKTNERR |
| 727 | O14979 | 288 | DEEPVKKLLES |
| 728 | O14979 | 302 | QIGSGKCEIKV |
| 729 | Q14103 | 98 | QREEWKMFIGG |
| 730 | Q14103 | 153 | SESVDKVMDQK |
| 731 | Q14103 | 158 | KVMDQKEHKLN |
| 732 | Q14103 | 218 | LPMDNKTNKRR |
| 733 | Q14103 | 341 | QSGYGKVSRRG |
| 734 | P16862 | 58 | DSISLKILLRP |
| 735 | P16862 | 225 | RSAIFKGCRAF |
| 736 | P16862 | 248 | GPEYIKEFHWE |
| 737 | P16862 | 432 | CDIVSKHRSRG |
| 738 | P16862 | 585 | KLPKDKRLKIA |
| 739 | O00159 | 281 | SSINDKSDWKV |
| 740 | O00159 | 361 | RKIIAKGEELL |
| 741 | O00159 | 455 | IELTLKSEQEE |
| 742 | O00159 | 561 | TGFLDKNNDLL |
| 743 | O00159 | 578 | TMCSSKNPIMS |
| 744 | P13538 | 31 | IEAQNKPFDAK |
| 745 | P13538 | 88 | PPKYDKIEDMA |
| 746 | P13538 | 208 | SGEKKKEEQSG |

| | | | |
|---|---|---|---|
| 747 | P13538 | 297 | IMSNKKPELID |
| 748 | P13538 | 386 | TEVADKAAYLM |
| 749 | P13538 | 400 | SAELLKALCYP |
| 750 | P11940 | 108 | IKNLDKSIDNK |
| 751 | P11940 | 167 | LLNDRKVFVGR |
| 752 | P11940 | 221 | PALSVKVMTDE |
| 753 | P11940 | 246 | HEDAQKAVDEM |
| 754 | P11940 | 312 | DERLRKEFSPF |
| 755 | P11940 | 371 | ALAQRKEERQA |
| 756 | P11940 | 512 | TVPQYKYAAGV |
| 757 | P06400 | 94 | GGYIQKKKELW |
| 758 | P06400 | 236 | PPMLLKEPYKT |
| 759 | P06400 | 417 | KESILKRVKDI |
| 760 | Q86L05 | 195 | LVSLLKKGWQN |
| 761 | P0CX43 | 54 | FSGSLKLPNCP |
| 762 | P0CX43 | 92 | VDDLKKLNKNK |
| 763 | P0CX43 | 133 | LSKAGKFPTPV |
| 764 | P0CX43 | 160 | IKFQLKKVLCL |
| 765 | P0CX43 | 196 | VSLLKKNWQNV |
| 766 | P47914 | 87 | KPKIPKGVSRK |
| 767 | P47914 | 106 | HPKLGKRARAR |
| 768 | P47914 | 114 | RARIAKGLRLC |
| 769 | Q969Q0 | 30 | QYKKGKDSLYA |
| 770 | Q969Q0 | 64 | KAKTTKKIVLR |
| 771 | Q969Q0 | 65 | AKTTKKIVLRL |
| 772 | Q969Q0 | 80 | PNCRSKRMLAI |
| 773 | P0CX27 | 32 | QYKAGKASLFA |
| 774 | P0CX27 | 63 | FHKKAKTTKKV |
| 775 | P0CX27 | 66 | KAKTTKKVVLR |
| 776 | P0CX27 | 98 | LGGEKKQKGQA |
| 777 | P02393 | 26 | LSEFIKELEEK |
| 778 | P07472 | 103 | KEGMSKEDGDE |
| 779 | Q12962 | 175 | ALQHCKMKGTA |
| 780 | Q04207 | 37 | MRFRYKCEGRS |
| 781 | Q04207 | 123 | IQCVKKRDLEQ |
| 782 | Q04207 | 315 | KSIMKKSPFNG |
| 783 | Q53688 | 140 | DTVISKGLLKI |
| 784 | Q53688 | 144 | SKGLLKIVKDG |
| 785 | Q53688 | 265 | FQEELKLNSDG |
| 786 | Q53688 | 379 | PEAYTKLQQYM |
| 787 | E5KWG9 | 68 | FTMLPKGLDPY |
| 788 | E5KWG9 | 94 | VGSLLKEVHEN |
| 789 | E5KWG9 | 126 | FPSAEKLGKVS |
| 790 | E5KWG9 | 168 | GIDYSKRTMNL |
| 791 | B6A879 | 210 | GDIGNKSKKVQ |
| 792 | B6A879 | 298 | HAMGHKLDLAF |
| 793 | B6A879 | 402 | SGVKAKLKKSN |
| 794 | B6A879 | 565 | TPRTVKQKGEY |
| 795 | Q9L5X8 | 22 | HVDHGKTTLVD |
| 796 | Q9L5X8 | 165 | DQLDFKVVYAS |
| 797 | Q9L5X8 | 236 | TRGSVKANQQV |
| 798 | Q9L5X8 | 359 | TDDPDKFRVSG |

| | | | |
|---|---|---|---|
| 799 | D0ZRV8 | 76 | IETLQKSIDNE |
| 800 | Q6NEZ3 | 90 | FDALIKIKDIS |
| 801 | Q6NEZ3 | 280 | DTSGSKTITAR |
| 802 | Q6NEZ3 | 312 | ELDTKKPEDTD |
| 803 | Q6NEZ3 | 408 | KNESKKPNESY |
| 804 | Q9UKV3 | 41 | EGRWRKLPKMP |
| 805 | Q9UKV3 | 81 | RVTDLKAALEQ |
| 806 | Q9UKV3 | 113 | LENLQKHSTPH |
| 807 | Q9UKV3 | 138 | QNSFIKQYLEK |
| 808 | Q9UKV3 | 220 | DSDDEKPRKGE |
| 809 | Q9UKV3 | 349 | QEEARKSHLAR |
| 810 | P18281 | 23 | AETVDKSAPQI |
| 811 | Q9SJU4 | 358 | KEENVKAAQDI |
| 812 | Q9SJU4 | 369 | LLARAKANSLA |
| 813 | Q9SJU4 | 389 | ESEEAKEGMFV |
| 814 | Q9ZU52 | 53 | LVKTAKSIASP |
| 815 | Q9ZU52 | 273 | AEHKNKASPET |
| 816 | Q9ZU52 | 345 | RTWQGKPEKIE |
| 817 | Q9ZU52 | 354 | IEASQKALLVR |
| 818 | Q9ZU52 | 361 | LLVRAKANSLA |
| 819 | P62157 | 78 | MARKMKDTDSE |
| 820 | P04573 | 50 | GQTYTKRETEA |
| 821 | P04573 | 156 | LIKKSKNALKE |
| 822 | P00889 | 107 | QKMLPKAKGGE |
| 823 | P00889 | 321 | QKEVGKDVSDE |
| 824 | P00044 | 60 | TDANIKKNVLW |
| 825 | P00044 | 61 | DANIKKNVLWD |
| 826 | P00078 | 22 | GEKLFKGRAAQ |
| 827 | P00077 | 61 | GFAYSKANADS |
| 828 | P00043 | 96 | GLKKAKDRNDL |
| 829 | P00070 | 64 | AGNKNKAVIWE |
| 830 | P00047 | 61 | YTDANKQAGIT |
| 831 | Q71V39 | 215 | WFKGWKVERKE |
| 832 | P02993 | 222 | ERKEGKADGKT |
| 833 | P02994 | 84 | KFETPKYQVTV |
| 834 | Q96KQ7 | 28 | ALLLEKETRGA |
| 835 | Q96KQ7 | 200 | PPVPEKRPPEI |
| 836 | Q96KQ7 | 219 | VHSLGKVTSDL |
| 837 | Q96KQ7 | 226 | TSDLAKRRKLN |
| 838 | P53991 | 169 | KEDPAKKGLCS |
| 839 | P53991 | 194 | TGPTGKVLLLP |
| 840 | P10587 | 50 | EAASIKEEKGD |
| 841 | P10587 | 66 | LQENGKKVTLS |
| 842 | P10587 | 77 | KDDIQKMNPPK |
| 843 | P10587 | 136 | PIYSEKIIDMY |
| 844 | P11416 | 193 | IEKVRKAHQET |
| 845 | P11416 | 238 | TKCIIKTVEFA |
| 846 | P27064 | 21 | GVKDYKLTYYT |
| 847 | P27064 | 128 | NVFGFKALRAL |
| 848 | P04717 | 81 | SLDRYKGRCYE |
| 849 | P04717 | 316 | FRVLAKALRLS |
| 850 | P04992 | 305 | VIDRQKNHGIH |

| | | | |
|---|---|---|---|
| 851 | P0A7J7 | 45 | KAFNAKTDSIE |
| 852 | P0A7J7 | 82 | AVLLKKAAGIK |
| 853 | Q5SLP6 | 80 | SYLIRKAAGLE |
| 854 | Q5SLP6 | 111 | EIAKQKMPDLN |
| 855 | P0CX53 | 54 | ATKEFKGIKVT |
| 856 | P0CX53 | 63 | VTVQLKIQNRQ |
| 857 | P62986 | 63 | DYNIQKESTLH |
| 858 | P62986 | 113 | VNCRKKKCGHT |
| 859 | P55072 | 63 | LLKGKKRREAV |
| 860 | P55072 | 81 | TCSDEKIRMNR |
| 861 | P55072 | 164 | RAVEFKVVETD |
| 862 | P55072 | 277 | PEIMSKLAGES |
| 863 | P55072 | 288 | ESNLRKAFEEA |
| 864 | P05547 | 20 | DDIDRKKAEVR |
| 865 | P05547 | 55 | RLLLRKKAAEE |
| 866 | P05547 | 99 | LRAIIKEYFDH |
| 867 | O95785 | 341 | EIQKLKQVPGD |
| 868 | O95785 | 743 | SAEEVKAIERR |
| 869 | Q9Y232 | 27 | EGPTQKLFLKR |
| 870 | Q9Y232 | 84 | YLVRWKGYDSE |
| 871 | Q9Y232 | 118 | HTEKQKESTLT |
| 872 | Q9Y232 | 185 | SLSSRKNMDLA |
| 873 | P00042 | 10 | KKGSEKKGATL |
| 874 | P26358 | 88 | KSLLNKDLSLE |
| 875 | P26358 | 194 | SDESIKEEDKD |
| 876 | O75530 | 20 | MPAAKKQKLSS |
| 877 | O75530 | 79 | KSKKCKYSFKC |
| 878 | O75530 | 83 | CKYSFKCVNSL |
| 879 | O75530 | 275 | MMNAIKESYDY |
| 880 | Q5VTE0 | 30 | GHLIYKCGGID |
| 881 | Q5VTE0 | 62 | AWVLDKLKAER |
| 882 | P0CE47 | 188 | AEWEAKILELA |
| 883 | P0CE47 | 264 | VEMFRKLLDEG |
| 884 | O75367 | 73 | ARDNKKGRVTP |
| 885 | P62807 | 21 | KKAVTKAQKKD |
| 886 | P62807 | 28 | QKKDGKKRKRS |
| 887 | P61830 | 116 | AAIHAKRVTIQ |
| 888 | P61830 | 122 | RVTIQKKDIKL |
| 889 | P62803 | 9 | RGKGGKGLGKG |
| 890 | P62803 | 45 | RRGGVKRISGL |
| 891 | P04637 | 120 | HSGTAKSVTCT |
| 892 | P04637 | 351 | EALELKDAQAG |
| 893 | Q86L05 | 38 | LQINLKNYDTK |
| 894 | Q86L05 | 44 | NYDTKKDKRFS |
| 895 | Q86L05 | 59 | IGTVTKPKLSV |
| 896 | Q86L05 | 91 | IEALKKIGPKN |
| 897 | Q86L05 | 101 | NKKAIKKLSKK |
| 898 | Q86L05 | 106 | KKLSKKYDAFL |
| 899 | Q7M1B9 | 20 | KVLKSKTPVVV |
| 900 | Q7M1B9 | 44 | APILDKLAGEY |
| 901 | Q7M1B9 | 82 | TLVIFKDGREV |
| 902 | P80322 | 104 | FRVLDKNGDGF |

| | | | |
|---|---|---|---|
| 903 | P80322 | 112 | DGFIDKDEFRA |
| 904 | P14909 | 23 | TTLLYKEIARN |
| 905 | P14909 | 172 | ISKRTKMIVFN |
| 906 | P14909 | 302 | VSLFKKRRDVM |
| 907 | P22498 | 124 | DEYANKDALNH |
| 908 | P22498 | 328 | TRTVVKRTEKG |
| 909 | P22498 | 451 | VDYNTKRLYWR |
| 910 | P0C835 | 11 | KAVKVKTPAGK |
| 911 | P0C835 | 24 | ELVPEKVWALA |
| 912 | P80053 | 117 | VRVDPKKLTRE |
| 913 | P80053 | 166 | LDEYIKITGKV |
| 914 | P13123 | 19 | EVDTSKIKKVW |
| 915 | P61991 | 9 | VKFKYKGEEKQ |
| 916 | P61991 | 22 | ISKIKKVWRVG |
| 917 | P13125 | 9 | VRFKYKGEEKE |
| 918 | P13125 | 52 | EKDAPKELMDM |
| 919 | P39476 | 19 | EVDISKIKKVW |
| 920 | P26358 | 257 | PTPKQKLKEEP |
| 921 | O75530 | 342 | EDDIDKIKPSE |
| 922 | O75530 | 408 | TLTHHKCGAAI |
| 923 | Q5VTE0 | 290 | VTTEVKSVEMH |
| 924 | Q5VTE0 | 313 | VGFKVKNVSVK |
| 925 | Q5VTE0 | 330 | VAGDSKNDPPM |
| 926 | Q5VTE0 | 386 | RRSGKKLEDGP |
| 927 | P49949 | 74 | PASEKKADPIN |
| 928 | O43524 | 663 | NFTGAKQASSQ |
| 929 | Q08369 | 245 | CGLYHKMNGIN |
| 930 | Q08369 | 366 | EMRPIKTEPGL |
| 931 | P16403 | 64 | LAALKKALAAA |
| 932 | P16403 | 106 | ASGSFKLNKKA |
| 933 | P16403 | 119 | GEAKPKVKKAG |
| 934 | P16403 | 140 | AKKPKKAAGGA |
| 935 | P10412 | 21 | EKTPVKKKARK |
| 936 | P10412 | 119 | GEAKPKAKKAG |
| 937 | P10412 | 122 | KPKAKKAGAAK |
| 938 | P16401 | 17 | PAPVEKSPAKK |
| 939 | P16401 | 149 | KAAGAKKAVKK |
| 940 | P16401 | 153 | AKKAVKKTPKK |
| 941 | O75367 | 161 | RKSKKKQGEVS |
| 942 | O75367 | 167 | QGEVSKAASAD |
| 943 | P40283 | 8 | PKAEKKPAEKK |
| 944 | P40283 | 18 | KPASEKPVEEK |
| 945 | P40283 | 39 | KPKAGKKLPKE |
| 946 | P40283 | 54 | GDKKKKMKKKS |
| 947 | P40283 | 64 | SVETYKIYIFK |
| 948 | P40283 | 72 | IFKVLKQVHPD |
| 949 | P40283 | 105 | AQEASKLARYN |
| 950 | O14979 | 221 | KLIDPKRAKAL |
| 951 | O14979 | 234 | KEPPKKVFVGG |
| 952 | Q14103 | 72 | KIDASKNEEDE |
| 953 | Q14103 | 110 | SWDTTKKDLKD |
| 954 | Q14103 | 170 | KVIDPKRAKAM |

| | | | |
|---|---|---|---|
| 955 | Q14103 | 182 | TKEPVKKIFVG |
| 956 | Q14103 | 197 | DTPEEKIREYF |
| 957 | Q14103 | 221 | DNKTNKRRGFC |
| 958 | Q14103 | 237 | EEEPVKKIMEK |
| 959 | Q14103 | 243 | KIMEKKYHNVG |
| 960 | Q14103 | 251 | NVGLSKCEIKV |
| 961 | P16862 | 275 | RCMEFKKREGR |
| 962 | P16862 | 369 | LDRICKAIDYV |
| 963 | P16862 | 529 | NKIVRKPLMES |
| 964 | P16862 | 583 | EPKLPKDKRLK |
| 965 | O00159 | 151 | KTEATKRLLQF |
| 966 | O00159 | 224 | SYLLEKSRVVH |
| 967 | O00159 | 336 | TENQLKYLTRL |
| 968 | O00159 | 404 | RSLASKDVESP |
| 969 | O00159 | 477 | QYFNNKIICDL |
| 970 | O00159 | 571 | LFRNLKETMCS |
| 971 | P13538 | 23 | LRKSEKERIEA |
| 972 | P13538 | 108 | VLYNLKERYAA |
| 973 | P13538 | 214 | EEQSGKMQGTL |
| 974 | Q96RI1 | 188 | MYMRRKCQECR |
| 975 | Q96RI1 | 227 | LRKNVKQHADQ |
| 976 | Q96RI1 | 317 | LVEFTKKLPGF |
| 977 | Q96RI1 | 318 | VEFTKKLPGFQ |
| 978 | P11940 | 196 | TNVYIKNFGED |
| 979 | P11940 | 259 | KELNGKQIYVG |
| 980 | P11940 | 324 | TITSAKVMMEG |
| 981 | P11940 | 361 | RIVATKPLYVA |
| 982 | P06400 | 63 | TALCQKLKIPD |
| 983 | P06400 | 95 | GYIQKKKELWG |
| 984 | P06400 | 153 | MSRLLKKYDVL |
| 985 | P06400 | 265 | SARIAKQLEND |
| 986 | P06400 | 329 | IYLKNKDLDAR |
| 987 | P06400 | 412 | CTVNPKESILK |
| 988 | P06400 | 462 | MESMLKSEEER |
| 989 | P0CX43 | 78 | DVDRAKSCGVD |
| 990 | P0CX43 | 95 | LKKLNKNKKLI |
| 991 | P0CX43 | 118 | SEVLIKQVPRL |
| 992 | P0CX43 | 156 | VRSTIKFQLKK |
| 993 | P47914 | 52 | KKHNKKGLKKM |
| 994 | P47914 | 56 | KKGLKKMQANN |
| 995 | P47914 | 77 | IKALVKPKEVK |
| 996 | P47914 | 134 | AKDQTKAQAAA |
| 997 | Q969Q0 | 98 | LGGDKKRKGQV |
| 998 | P0CX27 | 24 | KHTQHKVTQYK |
| 999 | P0CX27 | 46 | RRYDRKQSGFG |
| 1000 | P0CX27 | 60 | KPVFHKKAKTT |
| 1001 | P02393 | 66 | FDVILKAAGAN |
| 1002 | P02393 | 115 | AEEAKKQLVEA |
| 1003 | P0A7K2 | 108 | DAEALKKALEE |
| 1004 | P07472 | 67 | TSAGEKKVNVI |
| 1005 | P07472 | 73 | KVNVIKVVREI |
| 1006 | Q12962 | 108 | ANGDVKPVVSS |

| | | | |
|---|---|---|---|
| 1007 | Q12962 | 177 | QHCKMKGTASG |
| 1008 | Q04207 | 79 | ISLVTKDPPHR |
| 1009 | Q53688 | 66 | ELGGEKEYRRL |
| 1010 | Q53688 | 176 | YDTLQKQNYTL |
| 1011 | Q53688 | 362 | VKECDKTNEIE |
| 1012 | Q53688 | 371 | IEEATKRNPEA |
| 1013 | Q53688 | 445 | STHDTKFSEDV |
| 1014 | E5KWG9 | 129 | AEKLGKVSELV |
| 1015 | B6A879 | 43 | VIFDGKVYTNT |
| 1016 | B6A879 | 531 | SLSMGKNGFVL |
| 1017 | Q9L5X8 | 431 | RKGELKDMAPD |
| 1018 | D0ZRV8 | 19 | KVNRNKVPTPM |
| 1019 | Q6NEZ3 | 123 | VGKNVKLDGNQ |
| 1020 | Q6NEZ3 | 352 | SLLPNKAPIRV |
| 1021 | Q6NEZ3 | 455 | QMGVYKYSNSL |
| 1022 | D1BQI7 | 90 | MASIVKALSRK |
| 1023 | Q9UKV3 | 95 | AKSGQKSALVK |
| 1024 | Q9UKV3 | 268 | ADRNLKTEEEE |
| 1025 | Q9UKV3 | 302 | EAPILKEFKEE |
| 1026 | Q9UKV3 | 325 | MDERPKTRSQE |
| 1027 | Q9SJU4 | 293 | ASYTLKLLRNR |
| 1028 | Q9ZU52 | 8 | SASFVKPNTLS |
| 1029 | Q9ZU52 | 370 | LAQLGKYSAEG |
| 1030 | Q9ZU52 | 382 | NEDAKKGMFVK |
| 1031 | P62157 | 76 | TMMARKMKDTD |
| 1032 | P04573 | 64 | EARGPKGDKKN |
| 1033 | P04573 | 154 | MDLIKKSKNAL |
| 1034 | P00889 | 43 | ADLIPKEQARI |
| 1035 | P00889 | 103 | IPECQKMLPKA |
| 1036 | P00889 | 352 | HAVLRKTDPRY |
| 1037 | P00889 | 450 | PLERPKSMSTD |
| 1038 | P00063 | 61 | YSAANKNMAVN |
| 1039 | P00078 | 84 | LENPKKFMPGT |
| 1040 | P00078 | 90 | FMPGTKMSFAG |
| 1041 | P00043 | 18 | GANLFKTRCLQ |
| 1042 | P00047 | 19 | GANLFKTRCAQ |
| 1043 | P00047 | 81 | LENPKKFIPGT |
| 1044 | Q71V39 | 84 | KFETTKYYITI |
| 1045 | Q71V39 | 172 | YDEIVKEVSAY |
| 1046 | P02994 | 51 | AAELGKGSFKY |
| 1047 | P02994 | 159 | KMDSVKWDESR |
| 1048 | Q96KQ7 | 131 | CPSRAKMSMTG |
| 1049 | P53991 | 122 | TKINSKGKEVP |
| 1050 | P53991 | 291 | MYIQDKVEEYA |
| 1051 | P53991 | 315 | YFCGLKGMMPG |
| 1052 | P07041 | 123 | VTIQSKDIQLA |
| 1053 | P10587 | 53 | SIKEEKGDEVT |
| 1054 | P10587 | 144 | DMYKGKKRHEM |
| 1055 | P10587 | 188 | KTENTKKVIQY |
| 1056 | P10587 | 204 | SSHKGKKDTSI |
| 1057 | P10587 | 205 | SHKGKKDTSIT |
| 1058 | P11416 | 262 | QITLLKAACLD |

| | | | |
|---|---|---|---|
| 1059 | P11416 | 365 | KVYVRKRRPSR |
| 1060 | P27064 | 32 | PEYETKDTDIL |
| 1061 | P27064 | 201 | GLDFTKDDENV |
| 1062 | P27064 | 252 | CEEMMKRAIFA |
| 1063 | P04717 | 32 | PDYQTKDTDIL |
| 1064 | P04717 | 450 | IREACKWSPEL |
| 1065 | P04992 | 81 | SLDRYKGHRYR |
| 1066 | P0A7J7 | 10 | VQAYVKLQVAA |
| 1067 | P0A7J7 | 51 | TDSIEKGLPIP |
| 1068 | P0A7J7 | 100 | KDKVGKISRAQ |
| 1069 | P0CX53 | 40 | LGLSPKKVGED |
| 1070 | P0CX53 | 86 | VITALKEPPRD |
| 1071 | P55072 | 217 | QLAQIKEMVEL |
| 1072 | P05547 | 35 | EQSLKKQKKGF |
| 1073 | P05547 | 37 | SLKKQKKGFMT |
| 1074 | P05547 | 38 | LKKQKKGFMTP |
| 1075 | P05547 | 47 | TPERKKKLRLL |
| 1076 | P05547 | 48 | PERKKKLRLLL |
| 1077 | P05547 | 74 | AGERRKIIDQR |
| 1078 | P05547 | 112 | QIESDKYDVEL |
| 1079 | P05547 | 122 | LEIIRKDYEIN |
| 1080 | P05547 | 139 | NDLRGKFIKPT |
| 1081 | O95785 | 279 | IYFKQKEHLLE |
| 1082 | O95785 | 332 | QASREKIIEEI |
| 1083 | O95785 | 357 | RLQCPKCVFGT |
| 1084 | O95785 | 756 | FQKKKKKVANF |
| 1085 | P05141 | 23 | AAAISKTAVAP |
| 1086 | Q9Y232 | 31 | QKLFLKRNNVS |
| 1087 | Q9Y232 | 69 | ERIVDKRKNKK |
| 1088 | Q9Y232 | 71 | IVDKRKNKKGK |
| 1089 | Q9Y232 | 147 | NSNFSKTSPKA |
| 1090 | Q9Y232 | 151 | SKTSPKALVIG |
| 1091 | Q9Y232 | 175 | SQKFRKNTAPS |
| 1092 | Q9Y232 | 191 | NMDLAKSGIKI |
| 1093 | Q9Y232 | 200 | KILVPKSPVKS |
| 1094 | P26358 | 39 | DSLTEKECVKE |
| 1095 | O75530 | 76 | GKWKSKKCKYS |
| 1096 | O75530 | 106 | FNWHSKEGDPL |
| 1097 | Q5VTE0 | 212 | NMPWFKGWKVT |
| 1098 | Q5VTE0 | 244 | TRPTDKPLRLP |
| 1099 | P0CE47 | 90 | HADYVKNMITG |
| 1100 | P0CE47 | 177 | RGSALKALEGD |
| 1101 | P0CE47 | 358 | PGDNIKMVVTL |
| 1102 | O75367 | 12 | KKKSTKTSRSA |
| 1103 | O75367 | 116 | PELLAKKRGSK |
| 1104 | O75367 | 134 | TPPPAKKAKSP |
| 1105 | O75367 | 148 | KPVSKKAGGKK |
| 1106 | P62807 | 25 | TKAQKKDGKKR |
| 1107 | P62807 | 44 | SVYVYKVLKQV |
| 1108 | P62807 | 117 | VSEGTKAVTKY |
| 1109 | P62807 | 121 | TKAVTKYTSSK |
| 1110 | P62803 | 6 | MSGRGKGGKGL |

| | | | |
|---|---|---|---|
| 1111 | P62803 | 17 | GKGGAKRHRKV |
| 1112 | P04637 | 291 | EENLRKKGEPH |
| 1113 | P04637 | 292 | ENLRKKGEPHH |
| 1114 | Q86L05 | 98 | GPKNKKAIKKL |
| 1115 | Q86L05 | 130 | GPGLNKVGKFP |
| 1116 | Q7M1B9 | 15 | SDFAEKVLKSK |
| 1117 | P14909 | 33 | NVEKTKKIKII |
| 1118 | P14909 | 95 | GTDVKKEEVIV |
| 1119 | P14909 | 193 | SPNDVKKIVDI |
| 1120 | P14909 | 220 | FVYEGKMRSTL |
| 1121 | P14909 | 337 | VSKILKTSGFD |
| 1122 | P14909 | 344 | SGFDVKSLAIK |
| 1123 | P39462 | 24 | GVPKPKGPQVL |
| 1124 | P39462 | 86 | VVGYSKGDLVA |
| 1125 | P39462 | 252 | LNNSEKTLSVY |
| 1126 | P39462 | 266 | LAKQGKYVMVG |
| 1127 | P22498 | 304 | TRGNEKIVRDD |
| 1128 | P22498 | 374 | YDVLTKYWNRY |
| 1129 | Q9Y232 | 219 | SESPEKLDPVE |
| 1130 | Q9Y232 | 284 | LAVNGKGTSPF |
| 1131 | P0C835 | 34 | APKGRKGVKIG |
| 1132 | P80053 | 56 | SDGKLKTFMGW |
| 1133 | P80053 | 118 | RVDPKKLTREE |
| 1134 | P80053 | 130 | EQLSRKYIQAI |
| 1135 | P80053 | 279 | YPEGRKVTNEE |
| 1136 | P80053 | 309 | KFNAPKVKAKL |
| 1137 | P80053 | 311 | NAPKVKAKLIV |
| 1138 | P80053 | 360 | EWANNKMGEII |
| 1139 | P80053 | 371 | SDEEAKKLIVD |
| 1140 | P13123 | 28 | VWRVGKMVSFT |
| 1141 | P61991 | 19 | QVDISKIKKVW |
| 1142 | P61991 | 28 | VWRVGKMISFT |
| 1143 | Q5VTE0 | 311 | DNVGFKVKNVS |
| 1144 | P55907 | 21 | EHSGHKVYGPV |
| 1145 | O43524 | 606 | VMGHEKFPSDL |
| 1146 | Q08369 | 255 | NRPLIKPQRRL |
| 1147 | Q08369 | 322 | RKRKPKNLNKS |
| 1148 | Q08369 | 421 | SQASSKQDSWN |
| 1149 | P16403 | 52 | AVAASKERSGV |
| 1150 | P16403 | 81 | NNSRIKLGLKS |
| 1151 | P16403 | 90 | KSLVSKGTLVQ |
| 1152 | P16403 | 149 | GATPKKSAKKT |
| 1153 | P16403 | 153 | KKSAKKTPKKA |
| 1154 | P10412 | 137 | AGAAKKPKKAT |
| 1155 | P10412 | 140 | AKKPKKATGAA |
| 1156 | P10412 | 149 | AATPKKSAKKT |
| 1157 | P16401 | 140 | AGATPKKAKKA |
| 1158 | P16401 | 144 | PKKAKKAAGAK |
| 1159 | P16401 | 157 | VKKTPKKAKKP |
| 1160 | O75367 | 159 | GARKSKKKQGE |
| 1161 | P40283 | 7 | APKAEKKPAEK |
| 1162 | P40283 | 23 | KPVEEKSKAEK |

| | | | |
|---|---|---|---|
| 1163 | P40283 | 25 | VEEKSKAEKAP |
| 1164 | P40283 | 28 | KSKAEKAPAEK |
| 1165 | O14979 | 216 | HKLDGKLIDPK |
| 1166 | O14979 | 233 | GKEPPKKVFVG |
| 1167 | O14979 | 289 | EEPVKKLLESR |
| 1168 | Q14103 | 173 | DPKRAKAMKTK |
| 1169 | P16862 | 83 | LNSITKTQDWR |
| 1170 | P16862 | 415 | IFIPEKPATSS |
| 1171 | P16862 | 580 | DHNEPKLPKDK |
| 1172 | P16862 | 641 | RSLNWKDMLGW |
| 1173 | O00159 | 200 | SSRFGKYMDVQ |
| 1174 | O00159 | 269 | YLYLVKGQCAK |
| 1175 | O00159 | 357 | ALTHRKIIAKG |
| 1176 | O00159 | 396 | TWLVGKINRSL |
| 1177 | O00159 | 512 | LTFLEKLEDTV |
| 1178 | O00159 | 632 | KPNDAKQPGRF |
| 1179 | P13538 | 20 | APYLRKSEKER |
| 1180 | P13538 | 60 | SKEGGKVTVKT |
| 1181 | P13538 | 64 | GKVTVKTEGGE |
| 1182 | P13538 | 74 | ETLTVKEDQVF |
| 1183 | P13538 | 191 | KTVNTKRVIQY |
| 1184 | P13538 | 237 | AFGNAKTVRND |
| 1185 | P13538 | 249 | SSRFGKFIRIH |
| 1186 | P13538 | 368 | GNLKFKQKQRE |
| 1187 | Q96RI1 | 286 | QEITNKILKEE |
| 1188 | Q96RI1 | 352 | AEIFNKKLPSG |
| 1189 | Q96RI1 | 431 | LDVLQKLCKIH |
| 1190 | P11940 | 78 | NFDVIKGKPVR |
| 1191 | P11940 | 95 | DPSLRKSGVGN |
| 1192 | P11940 | 138 | DENGSKGYGFV |
| 1193 | P11940 | 268 | VGRAQKKVERQ |
| 1194 | P11940 | 269 | GRAQKKVERQT |
| 1195 | P06400 | 96 | YIQKKKELWGI |
| 1196 | P06400 | 136 | FFNLLKEIDTS |
| 1197 | P06400 | 202 | TFLLAKGEVLQ |
| 1198 | P06400 | 289 | NIDEVKNVYFK |
| 1199 | P06400 | 294 | KNVYFKNFIPF |
| 1200 | P06400 | 359 | QRTPRKSNLDE |
| 1201 | Q86L05 | 161 | KFQLKKVLCLA |
| 1202 | Q86L05 | 202 | GWQNIKTLYVK |
| 1203 | P0CX43 | 14 | VREHVKELLKY |
| 1204 | P0CX43 | 207 | GSLVVKSSMGP |
| 1205 | P47914 | 38 | KGVDPKFLRNM |
| 1206 | P47914 | 82 | KPKEVKPKIPK |
| 1207 | P47914 | 103 | YIAHPKLGKRA |
| 1208 | P47914 | 122 | RLCRPKAKAKA |
| 1209 | Q969Q0 | 28 | VTQYKKGKDSL |
| 1210 | P0CX27 | 61 | PVFHKKAKTTK |
| 1211 | P0CX27 | 67 | AKTTKKVVLRL |
| 1212 | P0CX27 | 97 | ELGGEKKQKGQ |
| 1213 | P02393 | 58 | PAEEEKTEFDV |
| 1214 | P0A7K2 | 52 | EAAEEKTEFDV |

| | | | |
|---|---|---|---|
| 1215 | P0A7K2 | 85 | GLKEAKDLVES |
| 1216 | P07472 | 112 | DEAKTKLEEAG |
| 1217 | Q04207 | 28 | IIEQPKQRGMR |
| 1218 | Q04207 | 303 | IEEKRKRTYET |
| 1219 | Q53688 | 147 | LLKIVKDGDEY |
| 1220 | Q53688 | 158 | FLEYFKWKLPL |
| 1221 | Q53688 | 160 | EYFKWKLPLTE |
| 1222 | Q53688 | 248 | LRSIIKNKIII |
| 1223 | Q53688 | 358 | ANQIVKECDKT |
| 1224 | Q53688 | 390 | PAVYAKAYEDT |
| 1225 | E5KWG9 | 191 | GVFEEKGVRAI |
| 1226 | E5KWG9 | 211 | SEELLKFCERS |
| 1227 | E5KWG9 | 229 | SWDSPKINRFT |
| 1228 | B6A879 | 121 | PDVNFKEDGSQ |
| 1229 | B6A879 | 291 | RELQKKAHAMG |
| 1230 | B6A879 | 445 | NLYSPKDPGEQ |
| 1231 | Q9L5X8 | 28 | TTLVDKLLQQS |
| 1232 | Q9L5X8 | 480 | HYGPHKGGNIG |
| 1233 | Q9L5X8 | 499 | ANAAGKALTNA |
| 1234 | Q6NC90 | 132 | RLKPLKPGKGF |
| 1235 | Q6NC90 | 135 | PLKPGKGFGEI |
| 1236 | D0ZRV8 | 36 | RLRVRKYRDPQ |
| 1237 | D0ZRV8 | 53 | LPESLKALLAY |
| 1238 | D0ZRV8 | 190 | HLPQWKAQQKM |
| 1239 | Q6NEZ3 | 145 | INDHLKASLEG |
| 1240 | Q9UKV3 | 213 | SISEEKGDSDD |
| 1241 | Q9UKV3 | 305 | ILKEFKEEGEE |
| 1242 | Q9SJU4 | 61 | LVKTAKTIASP |
| 1243 | Q9SJU4 | 186 | GARFAKWRTVV |
| 1244 | Q9SJU4 | 203 | SALAVKEAAWG |
| 1245 | Q9ZU52 | 288 | TLTMLKRRVPP |
| 1246 | P62157 | 31 | GTITTKELGTV |
| 1247 | P04573 | 14 | LGPEEKDECMK |
| 1248 | P00889 | 49 | EQARIKTFRQQ |
| 1249 | P00889 | 109 | MLPKAKGGEEP |
| 1250 | P00889 | 139 | VSWLSKEWAKR |
| 1251 | P00889 | 382 | VAQLYKIVPNV |
| 1252 | P00078 | 98 | FAGMKKPQERA |
| 1253 | P00043 | 61 | TDANKKKGVEW |
| 1254 | P00043 | 62 | DANKKKGVEWS |
| 1255 | P00070 | 31 | CHTVEKGAGHK |
| 1256 | P00070 | 96 | FPGLKKPQERA |
| 1257 | P00047 | 35 | QGGANKIGPNL |
| 1258 | P00047 | 47 | GLFGRKTGSVE |
| 1259 | Q71V39 | 212 | NMPWFKGWKVE |
| 1260 | P02993 | 212 | RLPWYKGWNIE |
| 1261 | P02994 | 210 | NAPWYKGWEKE |
| 1262 | P02994 | 222 | KAGVVKGKTLL |
| 1263 | Q96KQ7 | 341 | SSGRRKAKKKW |
| 1264 | Q96KQ7 | 358 | VKPSRKRRKRE |
| 1265 | P53991 | 38 | PVAATKASTAV |
| 1266 | P53991 | 170 | EDPAKKGLCSN |

| | | | |
|---|---|---|---|
| 1267 | P07041 | 116 | CAIHAKRVTIQ |
| 1268 | P10587 | 11 | LSDDEKFLFVD |
| 1269 | P10587 | 72 | KVTLSKDDIQK |
| 1270 | P68696 | 91 | GVITVKTSKSI |
| 1271 | P11416 | 86 | LPRIYKPCFVC |
| 1272 | P11416 | 147 | YCRLQKCFDVG |
| 1273 | P11416 | 164 | RNDRNKKKKEA |
| 1274 | P27063 | 6 | PQTETKASVGF |
| 1275 | P27064 | 81 | SLDRYKGRCYG |
| 1276 | P27064 | 177 | CTIKPKLGLSA |
| 1277 | P27064 | 183 | LGLSAKNYGRA |
| 1278 | P27064 | 334 | GTVVGKLEGER |
| 1279 | P27064 | 356 | DDFVEKDRSRG |
| 1280 | P27064 | 450 | IREASKWSPEL |
| 1281 | P04717 | 8 | PQTETKAKVGF |
| 1282 | P04992 | 146 | PPAYVKTFQGP |
| 1283 | P0A7J7 | 72 | FTFVTKTPPAA |
| 1284 | Q5SLP6 | 90 | EKGAHKPGREK |
| 1285 | P0CX53 | 31 | AALAPKIGPLG |
| 1286 | P0CX53 | 93 | PPRDRKKDKNV |
| 1287 | P0CX53 | 94 | PRDRKKDKNVK |
| 1288 | P0CX53 | 119 | RQMRDKSFGRT |
| 1289 | P0CX53 | 146 | CRVDFKNPHDI |
| 1290 | P62986 | 6 | MQIFVKTLTGK |
| 1291 | P55072 | 112 | DVKYGKRIHVL |
| 1292 | P55072 | 148 | YRPIRKGDIFL |
| 1293 | P55072 | 295 | FEEAEKNAPAI |
| 1294 | P05547 | 12 | AEEAKKKQDDI |
| 1295 | P05547 | 68 | KEQERKAGERR |
| 1296 | P05547 | 84 | RCGQPKNLDGA |
| 1297 | O95785 | 179 | VEDTPKTLDMA |
| 1298 | O95785 | 385 | PGQTTKEPFGG |
| 1299 | O95785 | 610 | AALELKQAFRE |
| 1300 | O95785 | 641 | IVLVAKLGPQV |
| 1301 | O95785 | 697 | PAMALKHEERK |
| 1302 | P05141 | 63 | VVRIPKEQGVL |
| 1303 | P05141 | 147 | AADVGKAGAER |
| 1304 | P05141 | 268 | RDEGGKAFFKG |
| 1305 | Q9Y232 | 76 | KNKKGKTEYLV |
| 1306 | Q9Y232 | 195 | AKSGIKILVPK |
| 1307 | P00042 | 85 | YIPGTKMAFGG |
| 1308 | P00042 | 92 | AFGGLKKEKDR |
| 1309 | P26358 | 43 | EKECVKEKLNL |
| 1310 | P26358 | 134 | PKPLSKPRTPR |
| 1311 | O75530 | 22 | AAKKQKLSSDE |
| 1312 | O75530 | 74 | GKGKWKSKKCK |
| 1313 | O75530 | 211 | LLSVSKDHALR |
| 1314 | O75530 | 332 | AIVCWKPGKME |
| 1315 | Q5VTE0 | 44 | IEKFEKEAAEM |
| 1316 | Q5VTE0 | 64 | VLDKLKAERER |
| 1317 | P0CE47 | 25 | HVDHGKTTLTA |
| 1318 | P0CE47 | 295 | GQVLAKPGTIK |

| | | | |
|---|---|---|---|
| 1319 | O75367 | 9 | RGGKKKSTKTS |
| 1320 | O75367 | 34 | LRYIKKGHPKY |
| 1321 | O75367 | 72 | AARDNKKGRVT |
| 1322 | O75367 | 135 | PPPAKKAKSPS |
| 1323 | P62807 | 6 | MPEPAKSAPAP |
| 1324 | P68432 | 116 | CAIHAKRVTIM |
| 1325 | P09988 | 116 | CAIHGKRVTIQ |
| 1326 | Q55BN9 | 65 | ELLIKKLPFQR |
| 1327 | P61830 | 57 | IRRFQKSTELL |
| 1328 | P61830 | 123 | VTIQKKDIKLA |
| 1329 | P62803 | 13 | GKGLGKGGAKR |
| 1330 | P04637 | 101 | SVPSQKTYQGS |
| 1331 | P04637 | 132 | SPALNKMFCQL |
| 1332 | P04637 | 321 | PQPKKKPLDGE |
| 1333 | Q86L05 | 18 | VSQLFKEAQES |
| 1334 | Q86L05 | 24 | EAQESKRGFLE |
| 1335 | Q86L05 | 102 | KKAIKKLSKKY |
| 1336 | P80322 | 44 | LGTIMKRLGMS |
| 1337 | P80322 | 153 | DYEEWKEIIQE |
| 1338 | P14909 | 166 | DDLQSKISKRT |
| 1339 | P14909 | 281 | TSFVQKAAVKA |
| 1340 | P14909 | 334 | FPNVSKILKTS |
| 1341 | P14909 | 349 | KSLAIKLIEEK |
| 1342 | P39462 | 31 | PQVLIKVEAAG |
| 1343 | P39462 | 61 | EDLGVKLPVTL |
| 1344 | P39462 | 172 | SLDPTKTLLVV |
| 1345 | P39462 | 192 | AVQIAKAVSGA |
| 1346 | P39462 | 320 | KPMITKTMKLE |
| 1347 | P39462 | 323 | ITKTMKLEEAN |
| 1348 | P22498 | 219 | GYVGVKSGFPP |
| 1349 | P22498 | 255 | IKSVSKKPVGI |
| 1350 | Q9Y232 | 388 | AADDSKLVLLS |
| 1351 | Q9Y232 | 417 | LTDDRKRESTK |
| 1352 | Q9Y232 | 422 | KRESTKMAEAI |
| 1353 | P0C835 | 37 | GRKGVKIGLFK |
| 1354 | P0C835 | 42 | KIGLFKDPETG |
| 1355 | P80053 | 202 | VATIAKEAANK |
| 1356 | P80053 | 239 | SEMGAKIVGVS |
| 1357 | P80053 | 247 | GVSDSKGGVIN |
| 1358 | Q5VTE0 | 385 | DRRSGKKLEDG |
| 1359 | P00219 | 10 | NYRTSKPVVGD |
| 1360 | O43524 | 176 | ESSPDKRLTLS |
| 1361 | O43524 | 197 | PYFKDKGDSNS |
| 1362 | O43524 | 360 | SPSVSKPCTVE |
| 1363 | P16403 | 17 | APPAEKAPVKK |
| 1364 | P16403 | 22 | KAPVKKKAAKK |
| 1365 | P16403 | 23 | APVKKKAAKKA |
| 1366 | P16403 | 46 | SELITKAVAAS |
| 1367 | P16403 | 85 | IKLGLKSLVSK |
| 1368 | P16403 | 109 | SFKLNKKAASG |
| 1369 | P16403 | 121 | AKPKVKKAGGT |
| 1370 | P16403 | 137 | VGAAKKPKKAA |

| | | | |
|---|---|---|---|
| 1371 | P16403 | 148 | GGATPKKSAKK |
| 1372 | P16403 | 157 | KKTPKKAKKPA |
| 1373 | P10412 | 117 | ASGEAKPKAKK |
| 1374 | P16401 | 55 | AVAASKERNGL |
| 1375 | O75367 | 189 | TVLSTKSLFLG |
| 1376 | P40283 | 12 | KKPAEKKPASE |
| 1377 | P40283 | 34 | APAEKKPKAGK |
| 1378 | P40283 | 51 | GAGGDKKKKMK |
| 1379 | P40283 | 53 | GGDKKKKMKKK |
| 1380 | P40283 | 56 | KKKKMKKKSVE |
| 1381 | Q42681 | 64 | ELVIRKLPFQR |
| 1382 | Q41811 | 13 | GKGLGKGARKR |
| 1383 | O14979 | 180 | VDCTIKTDPVT |
| 1384 | O14979 | 204 | AASVDKVLELK |
| 1385 | O14979 | 227 | RAKALKGKEPP |
| 1386 | O14979 | 229 | KALKGKEPPKK |
| 1387 | O14979 | 306 | GKCEIKVAQPK |
| 1388 | O14979 | 311 | KVAQPKEVYRQ |
| 1389 | Q14103 | 114 | TKKDLKDYFSK |
| 1390 | Q14103 | 161 | DQKEHKLNGKV |
| 1391 | Q14103 | 165 | HKLNGKVIDPK |
| 1392 | Q14103 | 238 | EEPVKKIMEKK |
| 1393 | Q14103 | 260 | KVAMSKEQYQQ |
| 1394 | P16862 | 25 | SVQSYKAAIDF |
| 1395 | P16862 | 66 | LRPDEKINKNV |
| 1396 | P16862 | 150 | NAVSTKPTPPP |
| 1397 | P16862 | 168 | SGLSSKVHSYT |
| 1398 | P16862 | 438 | HRSRGKRTTIV |
| 1399 | O00159 | 146 | ESGAGKTEATK |
| 1400 | O00159 | 188 | AFGNAKTLRND |
| 1401 | O00159 | 209 | VQFDFKGAPVG |
| 1402 | O00159 | 535 | DQRTRKSLGRG |
| 1403 | O00159 | 594 | SELSDKKRPET |
| 1404 | P13538 | 147 | LAYRGKKRQEA |
| 1405 | P13538 | 148 | AYRGKKRQEAP |
| 1406 | P13538 | 186 | ESGAGKTVNTK |
| 1407 | P13538 | 296 | QIMSNKKPELI |
| 1408 | P13538 | 349 | FSADEKTAIYK |
| 1409 | Q96RI1 | 33 | EMMSMKPAKGV |
| 1410 | Q96RI1 | 120 | EMPVTKKPRMG |
| 1411 | Q96RI1 | 198 | RLRKCKEMGML |
| 1412 | Q96RI1 | 218 | TEIQCKSKRLR |
| 1413 | Q96RI1 | 256 | KSCREKTELTP |
| 1414 | Q96RI1 | 413 | DRQYIKDREAV |
| 1415 | Q96RI1 | 420 | REAVEKLQEPL |
| 1416 | P11940 | 177 | RFKSRKEREAE |
| 1417 | P11940 | 188 | LGARAKEFTNV |
| 1418 | P11940 | 208 | DDERLKDLFGK |
| 1419 | P11940 | 279 | TELKRKFEQMK |
| 1420 | P11940 | 284 | KFEQMKQDRIT |
| 1421 | P06400 | 240 | LKEPYKTAVIP |
| 1422 | P06400 | 420 | ILKRVKDIGYI |

| | | | |
|---|---|---|---|
| 1423 | Q86L05 | 160 | VKFQLKKVLCL |
| 1424 | Q86L05 | 196 | VSLLKKGWQNI |
| 1425 | P0CX43 | 24 | YSNETKKRNFL |
| 1426 | P0CX43 | 25 | SNETKKRNFLE |
| 1427 | P0CX43 | 101 | NKKLIKKLSKK |
| 1428 | P0CX43 | 106 | KKLSKKYNAFI |
| 1429 | P0CX43 | 147 | DDLYGKVTDVR |
| 1430 | P0CX43 | 195 | FVSLLKKNWQN |
| 1431 | P47914 | 33 | RYESLKGVDPK |
| 1432 | P47914 | 51 | AKKHNKKGLKK |
| 1433 | P47914 | 79 | ALVKPKEVKPK |
| 1434 | P47914 | 84 | KEVKPKIPKGV |
| 1435 | Q969Q0 | 17 | CKKCGKHQPHK |
| 1436 | Q969Q0 | 61 | FRKKAKTTKKI |
| 1437 | P0CX27 | 15 | TYCKGKTCRKH |
| 1438 | P0CX27 | 86 | AQLTLKRCKHF |
| 1439 | P0CX27 | 100 | GEKKQKGQALQ |
| 1440 | P02393 | 107 | KEAVSKEEAEE |
| 1441 | P0A7K2 | 71 | KVAVIKAVRGA |
| 1442 | P0A7K2 | 101 | KEGVSKDDAEA |
| 1443 | P07472 | 68 | SAGEKKVNVIK |
| 1444 | P07472 | 87 | GLKEAKAAVDG |
| 1445 | Q12962 | 161 | SLAAQKFISDI |
| 1446 | Q04207 | 195 | NTAELKICRVN |
| 1447 | Q04207 | 218 | FLLCDKVQKED |
| 1448 | Q04207 | 314 | FKSIMKKSPFN |
| 1449 | Q53688 | 256 | IIIVEKILGFQ |
| 1450 | B6A879 | 107 | ILVEDKYTEET |
| 1451 | B6A879 | 212 | IGNKSKKVQEV |
| 1452 | B6A879 | 213 | GNKSKKVQEVW |
| 1453 | B6A879 | 567 | RTVKQKGEYAA |
| 1454 | B6A879 | 573 | GEYAAKNKLGG |
| 1455 | Q9L5X8 | 8 | TPQIDKLRNIA |
| 1456 | Q9L5X8 | 543 | TVNALKGKQLT |
| 1457 | Q6NEZ3 | 41 | DTQAPKPVNTV |
| 1458 | Q6NEZ3 | 62 | AWGLYKFNQKS |
| 1459 | Q6NEZ3 | 226 | ESASIKQDVQI |
| 1460 | Q6NEZ3 | 332 | ATSLLKPAKVM |
| 1461 | Q9UKV3 | 44 | WRKLPKMPEAV |
| 1462 | Q9UKV3 | 238 | QARAAKLSEGS |
| 1463 | Q9UKV3 | 294 | DDEGQKSREAP |
| 1464 | P18281 | 47 | LEEVAKPHELK |
| 1465 | Q9SJU4 | 82 | NATCGKRLASI |
| 1466 | Q9SJU4 | 147 | IVPGIKVDKGL |
| 1467 | Q9SJU4 | 268 | EGILLKPSMVT |
| 1468 | Q9SJU4 | 353 | KTWGGKEENVK |
| 1469 | Q9ZU52 | 50 | SDELVKTAKSI |
| 1470 | P11118 | 87 | TEEEIKEAFRV |
| 1471 | P00889 | 76 | GMRGMKGLVYE |
| 1472 | P00889 | 193 | GIHRTKYWELI |
| 1473 | P00889 | 215 | PCVAAKIYRNL |
| 1474 | P00063 | 19 | GEKIFKTCAQ |

| | | | |
|---|---|---|---|
| 1475 | P00063 | 30 | CHTVDKGAGHK |
| 1476 | P00063 | 87 | YIPGTKMVFPG |
| 1477 | P00077 | 16 | PGDAAKGEKIF |
| 1478 | P00043 | 12 | KGSEKKGANLF |
| 1479 | P00043 | 94 | FGGLKKAKDRN |
| 1480 | P00076 | 22 | CHSAQKGVNST |
| 1481 | P00076 | 52 | YSNANKNAAIV |
| 1482 | P00076 | 87 | AGIKAKKDRQD |
| 1483 | P00070 | 62 | YSAGNKNKAVI |
| 1484 | P00047 | 95 | FGGLKKNKDRN |
| 1485 | Q71V39 | 219 | WKVERKEGNAS |
| 1486 | P02993 | 44 | IEKFEKEAQEM |
| 1487 | P02993 | 51 | AQEMGKGSFKY |
| 1488 | P02994 | 214 | YKGWEKETKAG |
| 1489 | P02994 | 224 | GVVKGKTLLEA |
| 1490 | Q96KQ7 | 343 | GRRKAKKKWRK |
| 1491 | Q96KQ7 | 345 | RKAKKKWRKDS |
| 1492 | Q96KQ7 | 368 | EPPRAKEPRGV |
| 1493 | P53991 | 6 | MASLRKPSNHA |
| 1494 | P53991 | 234 | NVPSYKFTGLF |
| 1495 | P53991 | 285 | NRKGGKMYIQD |
| 1496 | P10587 | 17 | FLFVDKNFVNN |
| 1497 | P10587 | 145 | MYKGKKRHEMP |
| 1498 | P10587 | 202 | VASSHKGKKDT |
| 1499 | P11416 | 118 | RRSIQKNMVYT |
| 1500 | P11416 | 165 | NDRNKKKKEAP |
| 1501 | P11416 | 167 | RNKKKKEAPKP |
| 1502 | P27064 | 161 | QVERDKLNKYG |
| 1503 | P27064 | 305 | VIDRQKNHGMH |
| 1504 | P04717 | 227 | AEAIYKSQAET |
| 1505 | P04717 | 355 | RDDYIKKDRSR |
| 1506 | P04992 | 236 | ETGEIKGHYLK |
| 1507 | P04992 | 241 | KGHYLKATAGT |
| 1508 | P0A7J7 | 87 | KAAGIKSGSGK |
| 1509 | P0A7J7 | 95 | SGKPNKDKVGK |
| 1510 | P0CX53 | 130 | LASVTKEILGT |
| 1511 | P62986 | 88 | RQLAQKYNCDK |
| 1512 | P55072 | 109 | PCPDVKYGKRI |
| 1513 | P05547 | 13 | EEAKKKQDDID |
| 1514 | P05547 | 56 | LLLRKKAAEEL |
| 1515 | P05547 | 62 | AAEELKKEQER |
| 1516 | O95785 | 550 | QLGRNKSTVHP |
| 1517 | O95785 | 753 | RFSFQKKKKKV |
| 1518 | O95785 | 755 | SFQKKKKVAN |
