# Supplementary material for: iMethyl-PseAAC: Identification of Protein Methylation Sites via a Pseudo Amino Acid Composition Approach
Source: Biomed Res Int. 2014 May 22;2014:947416. doi: 10.1155/2014/947416 (PMC4054830; doi:10.1155/2014/947416)
Supplement: Supplementary file 1 — S1: An analysis of the benchmark dataset used in Hu et al. (Biopolymers, 2011, 95, 763-771) S2: List of self-conflict samples in the benchmark dataset used in Hu et al. (Biopolymers, 2011, 95, 763-771) S3: Benchmark dataset used for studying the Arg-methylation. It contains 1,481 samples, of which 185 are positive and 1,296 negative. These data were extracted from UniProtKB/Swiss-Prot database (version UniProt release 2013_06). S4: Benchmark dataset used for studying the Lys-methylation. It contains 1,884 samples, of which 226 are positive and 1,518 negative. These data were extracted from UniProtKB/Swiss-Prot database (version UniProt release 2013_06). S5: Seven negative subsets for studying Arg-methylation. Each subset contains 185 negative samples randomly taken from the 1,296 negative samples in Online Supporting Information S3 except for the 6th subset, which contains 186 samples. None of the samples in one subset occurs in any other subset. S6: Seven negative subsets for studying Lys-methylation. Each subset contains 217 negative samples randomly taken from the 1,518 negative samples in Online Supporting Information S4 except for the 5th subset, which only contains 216 samples. None of the samples in one subset occurs in any other subset. S7: Independent dataset for studying the Arg-methylation. It contains 75 samples, of which 20 are positive and 55 negative. None of the samples listed here occurs in Online Supporting Information S3. S8: Independent dataset for studying the Lys-methylation. It contains 40 samples, of which 14 are positive and 26 negative. None of the samples listed here occurs in Online Supporting Information S4. S9: The code for encoding the peptides investigated in this paper. [file 947416.f1.zip › S5.pdf]

Online Supporting Information S5. Seven negative subsets for studying Arg-methylation. Each subset contains 185 negative samples randomly taken from the 1,296 negative samples in Online Supporting Information S3 except for the 6th subset, which contains 186 samples. None of the samples in one subset occurs in any other subset.

(1) Subset 1 (185 samples)

Sample #	Protein ID	Site	Sequences
1	Q9NR22	229	VAIEDRQYKDF
2	Q9UBL3	324	LFSAQRLPPHG
3	Q92934	72	VEIRSRHSSYP
4	Q7Q2B7	222	IDLPERVDVII
5	P11911	9	GLEALRALPLL
6	P11911	118	CGTYLRVRNPV
7	Q09472	86	LSELLRSGSSP
8	Q09472	354	HKCQRREQANG
9	Q09472	408	WKNCTRHDCPV
10	Q09472	580	ITQDLRNHLVH
11	Q09472	604	ALKDRRMENLV
12	Q09472	649	LEEKRRTRLQK
13	Q09472	1015	TETEERSTELK
14	Q09472	1076	ESLPFRQPVDP
15	Q09472	1324	GEVTVRVVHAS
16	Q09472	1410	RPKCLRTAVYH
17	Q09472	1462	IPKPKRLQEWY
18	P03372	28	LEPLNRPQLKI
19	P03372	158	NSDNRRQGGRE
20	P03372	234	IDKNRRKSCQA
21	P03372	256	MKGGIRKDRRG
22	P03372	503	QQQHQRLAQLL
23	P03372	515	ILSHIRHMSNK
24	Q01844	614	GFGGGRRGGPG
25	Q01844	632	EQMGGRRGGRG
26	P22509	76	GGGRGRGGGRG
27	P22509	224	VIEDARHPHKY
28	P35637	213	YGQQDRGGRGR
29	P35637	269	KFGGPRDQGSR
30	P35637	274	RDQGSRHDSEQ
31	P35637	328	NLYTDRETGKL
32	P03347	299	RDYVDRFYKTL
33	P03347	305	FYKTLRAEQAS
34	P03347	429	KDCTERQANFL
35	P03347	452	NFLQSRPEPTA
36	P03347	502	PLTSLRSLFGN
37	P68431	54	LREIRRYQKST
38	P68431	73	FQRLVREIAQD
39	P68431	117	AIHAKRVTIMP
40	P68431	130	IQLARRIRGER
41	P62805	24	HRKVLRDNIQG

42	P62805	68	LENVIRDAVTY
43	P62805	96	LKRQGRTLYGF
44	P17096	84	PGRKPRGRPKK
45	P31942	19	SDGTVRLRGLP
46	P31942	21	GTVRLRGLPFG
47	P31942	105	YDPPRRLLGQR
48	P31942	222	PLNPIRVHIDI
49	O60506	303	TAAQARRRLMS
50	O60506	305	AQARRRLMSGK
51	O60506	446	PPTRGRGRGGR
52	Q07666	13	AARMSRSSGRS
53	Q07666	43	PPLPHRSRGGG
54	Q07666	291	VPVRGRGAAPP
55	Q07666	331	GATVTRGVPPP
56	P08199	394	KVRAARTLLAK
57	P08199	604	RIVTDRETGSS
58	O75925	63	KELYRRRFPQK
59	O75925	257	ITSLVRLSTTV
60	O75925	579	LLHSSRFFPYT
61	Q92804	101	GSQGGRAPSYD
62	Q92804	164	HTQDDRRDVSR
63	Q92804	165	TQDDRRDVSRY
64	Q92804	185	SQGGGRGRGGY
65	Q92804	321	SFATRRPEFMR
66	Q92804	372	NMNFARRNSCN
67	Q92804	415	GRGGDRGGYGG
68	Q92804	431	GYGGDRSSGGG
69	Q92804	459	GYGGDRGGGYG
70	Q92804	580	GKMGGRNDYRN
71	Q92804	584	GRNDYRNDQRN
72	P09867	82	HKVDGRVVEPK
73	P09867	88	VVEPKRAVSRE
74	O00267	17	EEDSERSSDGE
75	O00267	30	EVDEERRSAAG
76	O00267	130	LQNLWRDQREE
77	O00267	368	GNRYSRKGFLF
78	O00267	571	KVVTVRHQAVT
79	O00267	641	FVCKTRHLVLA
80	O00267	652	GGSKPRDVTNF
81	O00267	783	YGSGSRTPMYG
82	P69697	7	EPVDPRLEPWK
83	P69697	56	KRRQRRRPPQG
84	Q15554	28	SGRARRGRHEP
85	Q15554	30	RARRGRHEPGL
86	Q15554	47	GAGEARLEEAV
87	Q15554	69	ALRAFRGSRYG
88	Q15554	72	AFRGSRYGDFR
89	Q15554	192	TTQKLRNDLLN
90	Q15554	356	QPKNKRMTISR
91	P15056	188	KALMMRGLIPE
92	P15056	260	LFQGFRCQTCG
93	P15056	347	IPQPFRPADED

94	P15056	360	NQFGQRDRSSS
95	P15056	426	GPQRERKSSSS
96	Q5TAQ9	161	PALRERELGSS
97	Q96EY1	187	PEELFRKIFGE
98	Q96EY1	272	GPFVMRSTCRR
99	Q96EY1	276	MRSTCRRCGGR
100	Q96EY1	281	RRCGGRGSIII
101	Q96EY1	303	AKQKKRVMIPV
102	Q96EY1	364	LGGTARAQGLY
103	P06746	89	KLEKIRQDDTS
104	P06746	126	TLEDLRKNEDK
105	Q92997	215	ASRLMRRHKRR
106	Q92997	220	RRHKRRRRKQK
107	Q54G21	660	KMMESRNEAIT
108	Q54G21	708	INQLKRDYNTL
109	Q54G21	827	TLETERSALSS
110	Q54G21	841	LVENTRLETES
111	Q9R1E0	19	FEPLPRQRSCT
112	Q9R1E0	313	STFRPRTSSNA
113	Q9R1E0	551	TSAMNRLTPVK
114	Q9R1E0	586	CNGYGRMGVLH
115	Q13283	106	NNQALRRFMQT
116	Q13283	107	NQALRRFMQTF
117	Q13283	283	PASQPRPESKP
118	P31943	230	GAGFERMRRGA
119	P04264	30	INYQRRTTSSS
120	P04264	65	GGFGSRSLVNL
121	P04264	199	FIDKVRFLEQQ
122	P04264	343	SMDNNRSLDLD
123	P08729	48	GASRPRVAVRS
124	P08729	305	DLRNTRNEISE
125	P15720	10	KRSSFRHGSKM
126	P15720	48	FFGGDRHVPRR
127	P15720	96	NIVSPRTPPPM
128	P15720	162	GGSGSRPGSRS
129	P25274	128	FGYGGRAADYK
130	Q8IZS5	61	EKKVIRHDMPD
131	Q8IZS5	67	HDMPDRTLAAH
132	Q8IZS5	77	HQQKFRLPASA
133	P11940	153	QEAAERAIEKM
134	P11940	166	MLLNDRKVFVG
135	P11940	186	AELGARAKEFT
136	P11940	287	QMKQDRITRYQ
137	P11940	331	MMEGGRSKGFG
138	Q9JMB7	6	MTGRARARARG
139	Q9JMB7	36	GYIPPRPQQSP
140	Q9JMB7	51	LVGRGRQRGMV
141	Q9JMB7	84	ERGGRRRDFHD
142	Q9JMB7	147	EARRLRSALLF
143	Q8CDG1	177	FGLTARDPPRL
144	Q86WR7	140	AGKEHRKQDAE
145	Q86WR7	204	QKISERMAGNE

146	Q86WR7	221	PFREGRPGEWR
147	P09651	265	YSGGSRGYGSG
148	P22626	185	HNAEVRKALSR
149	P51991	239	GGNFGRGGNFG
150	Q99729	30	EASRGRGWTGA
151	P14678	228	PPPGMRGPPPP
152	Q14524	179	VKILARGFCLH
153	Q14524	340	CPEGYRCLKAG
154	Q14524	367	FLALFRLMTQD
155	P23246	399	FGPIERAVVIV
156	P68363	105	ANNYARGHYTI
157	P68363	264	LVPYPRIHFPL
158	P68363	320	CCLLYRGDVVP
159	P07437	77	TMDSVRSGPFG
160	P07437	162	EEYPDRIMNTF
161	P07437	282	GSQQYRALTVP
162	P07437	359	CDIPPRGLKMA
163	Q86V81	20	LNRSQRGGRGG
164	Q86V81	174	VPLDGRPMNIQ
165	Q86V81	197	AQSVNRGGMTR
166	Q86V81	202	RGGMTRNRGAG
167	O08583	56	GGGPMRNRPAI
168	O08583	203	GMTRNRGSGGF
169	P39748	70	MGMFYRTIRMM
170	P31269	207	HARSTRKKRCP
171	P31269	233	NMYLTRDRRYE
172	P31269	241	RYEVARLLNLT
173	P04049	100	CCAVFRLLHEH
174	P04049	143	THNFARKTFLK
175	P04049	231	VSSQHRYSTPH
176	P04049	282	IEDAIRSHSES
177	P04049	318	PAQRERAPVSG
178	P04049	333	NKIRPRGQRDS
179	P46783	8	MPKKNRIAIYE
180	P46783	80	GIQYLRDYLHL
181	P46783	96	PATLRRSRPET
182	P46783	105	ETGRPRPKGLE
183	P46783	116	GERPARLTRGE
184	P46783	128	DRDTYRRSAVP
185	O76922	87	SVRGRRLITDL

(2) Subset 2 (185 samples)

Sample #	Protein ID	Site	Sequences
1	Q9NR22	13	RCLLLRRKMAE
2	Q9NR22	14	CLLLRRKMAEN
3	Q9UBL3	211	ECMTTRQRPGK
4	Q9UBL3	231	TMSKERDVFLV
5	Q9UBL3	315	TTKKARSDPLF
6	Q9UBL3	374	PGDLYRACLYE
7	Q9UBL3	411	GYSMVRASHGV

8	Q9UBL3	592	KDLTYRPMSDM
9	Q92934	116	GRELRRMSDEF
10	Q92934	131	KKGLPRPKSAG
11	Q7Q2B7	84	ELVLFRFASRG
12	Q7Q2B7	88	FRFASRGDCQQ
13	Q7Q2B7	95	DCQQFRSLLHK
14	Q7Q2B7	341	EKDLHRIQIDV
15	Q7Q2B7	447	KNPYFRYTGAP
16	P11911	47	LGEEARLTCEN
17	P11911	96	VNKNHRGLYWC
18	P11911	137	EGTKNRIITAE
19	Q09472	1104	LSTIKRKLDTG
20	Q09472	1133	AWLYNRKTSRV
21	Q09472	1197	YSYQNRYHFCE
22	Q09472	1392	PPNQRRVYISY
23	Q09472	1494	QATEDRLTSAK
24	P03372	37	KIPLERPLGEV
25	P03372	142	SGYTVREAGPP
26	P03372	241	SCQACRLRKCY
27	P03372	436	TSSRFRMMNLQ
28	Q01844	392	VKMNKRTGQPM
29	P22509	56	GGGGFRGRGGG
30	P22509	84	GRGGKRGNQSG
31	P35637	521	EHRQDRRERPY
32	P03347	275	LNKIVRMYSPT
33	P03347	403	EGHTARNCRAP
34	P68431	129	DIQLARRIRGE
35	P62805	40	IRRLARRGGVK
36	P62805	79	TEHAKRKTVTA
37	P31942	93	IFRSSRSEIKG
38	P31942	129	YYGAGRGSMYD
39	P31942	261	NNMQHRYIELF
40	P31942	301	YGSVGRMGMGN
41	P31942	323	LGGYGRGGGGS
42	O60506	103	VMKTYRQREKQ
43	O60506	229	NNHEIRSGKHI
44	O60506	451	RGRGGRGGYGY
45	O60506	504	GGRGARGAAPS
46	O60506	557	GARGGRGGNVG
47	O60506	565	NVGGKRKADGY
48	Q07666	10	DDPAARMSRSS
49	Q07666	204	GKGSMRDKAKE
50	Q07666	436	VKGAYREHPYG
51	P08199	343	AVVDVRTGTNR
52	P08199	421	DALEIRLVSQD
53	P08199	474	GQRQERTGKNS
54	Q28165	17	GAAGGRGSGPG
55	Q28165	25	GPGRRRHLVPG
56	Q28165	78	EPPRPRAPPGA
57	Q28165	200	CGSVNRVTILC
58	Q28165	248	KVIPKRTNRPG
59	O75925	276	TAEIGRNYSMA

60	O75925	528	LIQDYRHPFHM
61	Q96PU8	106	RILGPRGLTAK
62	Q92804	221	RDYGPRTDADS
63	Q92804	320	VSFATRRPEFM
64	Q92804	341	GRGGYRGRGGF
65	Q92804	349	GGFQGRGGDPK
66	Q92804	388	RPEDSRPSGGD
67	Q92804	406	GERGYRGRGGR
68	Q92804	422	GYGGDRSGGGY
69	P09867	31	TDESLRSHFEQ
70	P09867	196	SSQRGRSGSGN
71	P09867	300	YFAKPRNQGGY
72	O00267	31	VDEERRSAAGS
73	O00267	65	EEDDDRPPKKP
74	O00267	319	DRIKARMSLKD
75	O00267	346	DAEKIRSLGGD
76	O00267	516	LKVLPRDLQLC
77	O00267	582	RKKDNRFAVAL
78	O00267	711	IGQTVRISQGP
79	O00267	798	LQDGSRTPHYG
80	O00267	831	PNTPSRAEEEY
81	O00267	1049	ILGEDREATGV
82	O00267	1066	EDGIVRMDLDE
83	P69697	78	PTSQSRGDPTG
84	Q15554	13	SDGSGRAAGRR
85	Q15554	77	RYGDFRQIRDI
86	Q15554	102	VSRLLRVMQCL
87	P15056	239	THNFVRKTFFT
88	Q5TAQ9	11	SSTDGRTDLAN
89	Q5TAQ9	151	ALPRPRWQALP
90	Q5TAQ9	177	EACGARVFVQR
91	Q5TAQ9	182	RVFVQRFRLQH
92	Q96EY1	102	ILGVPRNASQK
93	Q96EY1	332	IFITFRVQKSP
94	P06746	102	INFLTRVSGIG
95	P06746	137	LNHHQRIGLKY
96	P06746	258	RRIDIRLIPKD
97	Q92997	145	SAQRERPRRRD
98	Q92997	167	AKGERRREPGG
99	Q60487	105	MKEDGRLLASK
100	Q54G21	180	VANGDRVSIKY
101	Q54G21	230	GVIGMRKSAKR
102	Q54G21	793	EESLKRLKLDM
103	Q54G21	1425	IDNSPRQTTTE
104	Q9R1E0	249	GKSPRRRAASM
105	Q9R1E0	487	AQPNSRVLGQN
106	Q9R1E0	534	ASVNGRTLPHV
107	Q13283	159	EEPEERQQTPE
108	Q13283	296	QIPPQRPQRDQ
109	P31943	16	FVVKVRGLPWS
110	P31943	52	YTREGRPSGEA
111	P31943	192	SRAEVRTHYDP

112	P31943	217	RPGAGRGYNSI
113	P04264	37	TSSSTRRSGGG
114	P04264	386	QITAGRHGDSV
115	P08729	30	RLSSARPGGLG
116	P08729	46	GLGASRPRVAV
117	P08729	52	PRVAVRSAYGG
118	P08729	64	VGAGIREVTIN
119	P08729	187	DEINHRTAAEN
120	P08729	276	MAKCSRAEAEA
121	P08729	313	ISEMNRAIQRL
122	P08729	317	NRAIQRLQAEI
123	Q36736	14	SAKLDRLDQEF
124	P02687	41	LDSLGRFFGSD
125	P15720	78	RSQHGRPGDDN
126	P15720	166	SRPGSRSGSPV
127	P25274	9	KRPSQRHGSKY
128	P25274	49	FFSSDRGAPKR
129	Q8IZS5	31	MVKEDREATEG
130	Q8IZS5	142	QDESGREETLN
131	Q8IZS5	177	LEEFQRSQKTI
132	P11940	89	IMWSQRDPSLR
133	P11940	206	DMDDERLKDLF
134	P11940	278	QTELKRKFEQM
135	P11940	311	DDERLRKEFSP
136	Q9JMB7	83	AERGGRRRDFH
137	Q9JMB7	145	LMEARRLRSAL
138	Q9JMB7	160	EDLIGRCHAFD
139	Q8CDG1	9	VRPLFRGPTPV
140	Q8CDG1	88	RPPSKREVPPL
141	Q8CDG1	111	MVRKDREEPRS
142	Q8CDG1	156	LLPLGRAASSI
143	Q86WR7	6	MPVTHRKSDAS
144	Q86WR7	22	TSPSCRLRAFS
145	Q86WR7	173	PDPPRRELRAP
146	Q86WR7	176	PRRELRAPSPP
147	Q86WR7	265	TNGAAREPRRT
148	P22626	15	LERKKREKEQF
149	P22626	60	DPASKRSRGFG
150	P22626	228	PGSNFRGGSDG
151	P22626	238	GYGSGRGFGDG
152	P51991	9	KPPPGRPQPDS
153	P51991	113	KRAVSREDSVK
154	P51991	143	EEYNLRDYFEK
155	P51991	226	GNFMGRGGNFG
156	Q99729	248	GGRGNRNRGNR
157	Q99729	250	RGNRNRGNRGS
158	P14678	221	PPPGMRPPPPG
159	P23246	7	SRDRFRSRGGG
160	P23246	18	GGGFHRRGGGG
161	P23246	25	GGGGGRGGLHD
162	P68363	215	YDICRRNLDIE
163	P68363	243	ITASLRFDGAL

164	P68363	308	VKCDPRHGKYM
165	P07437	213	YDICFRTLKLT
166	P07437	241	VTTCLRFPGQL
167	P07437	276	APLTSRGSQQY
168	P07437	306	AACDPRHGRYL
169	P07437	380	QELFKRISEQF
170	Q86V81	63	RPAIARGAAGG
171	Q86V81	79	PAPYSRPKQLP
172	Q86V81	144	YDRSGRSLGTA
173	O08583	189	IDTQRRPAQSI
174	P39748	211	EFHLSRILQEL
175	P39748	239	YCESIRGIGPK
176	P39748	332	RLSKSRQGSTQ
177	P39748	355	LSSAKRKEPEP
178	P31269	128	GLPSSRPYGIK
179	P31269	141	PLSARRGDCPT
180	P04049	59	TSNTIRVFLPN
181	P04049	223	RESVSRMPVSS
182	P46783	55	QSLKSRGYVKE
183	O76922	86	GSVRGRRLITD
184	O76922	123	FKVLKRPNWTI
185	O76922	146	ATRLRRSFLYE

(3) Subset 3 (185 samples)

Sample #	Protein ID	Site	Sequences
1	Q9NR22	368	NAKNVRDLDFT
2	Q9UBL3	181	LTWQSRTQDEH
3	Q9UBL3	380	ACLYERVLLAL
4	Q9UBL3	417	ASHGVRKGAWY
5	Q9UBL3	437	PDTAARLGWSQ
6	Q9UBL3	622	EVDGRRSPPWE
7	Q92934	112	AQRYGRELRRM
8	Q92934	142	TATQMRQSSSW
9	Q92934	149	SSSWTRVFQSW
10	Q92934	157	QSWWDRNLGRG
11	Q7Q2B7	40	QGMAVRLLQEG
12	Q7Q2B7	67	TTHLSRGHTSH
13	Q7Q2B7	299	NLVALRDAAMK
14	P11911	162	LLFRKRWQNEK
15	Q09472	15	PPSAKRPKLSS
16	Q09472	370	NLPHCRTMKNV
17	Q09472	504	SPQGMRPMSNM
18	Q09472	626	ESANNRAEYYH
19	Q09472	1187	LCTIPRDATYY
20	Q09472	1297	RLPSTRLGTFL
21	Q09472	1342	PGMKARFVDSG
22	P03372	183	SAKETRYCAVC
23	P03372	269	MLKHKRQRDDG
24	Q01844	486	GGPMGRMGGRG
25	P22509	38	GFGGGRGGFGG

26	P22509	58	GGFRGRGGGGG
27	P22509	74	QSGGGRGRGGG
28	P22509	106	GVFICRGKEDA
29	P35637	216	QDRGGRGRGGS
30	P35637	234	GGGYNRSSGGY
31	P35637	372	SFATRRADFNR
32	P35637	518	SRGEHRQDRRE
33	P03347	20	RWEKIRLRPGG
34	P03347	22	EKIRLRPGGKK
35	P68431	41	VKKPHRYRPGT
36	P68431	43	KPHRYRPGTVA
37	P68431	50	GTVALREIRRY
38	P68431	64	TELLIRKLPFQ
39	P68431	84	FKTDLRFQSSA
40	P62805	18	KGGAKRHRKVL
41	P62805	37	KPAIRRLARRG
42	P62805	46	RGGVKRISGLI
43	P17096	24	DGTEKRGRGRP
44	P17096	28	KRGRGRPRKQP
45	P31942	174	RMRDGRGMGGH
46	P31942	205	RGLPFRATEND
47	O60506	381	IHFDERDGAVK
48	O60506	526	AGYSQRGGPGS
49	O60506	554	GVRGARGGRGG
50	O60506	577	QPDSKRRQTNN
51	O60506	578	PDSKRRQTNNQ
52	Q07666	31	AHPSVRQTPSR
53	Q07666	282	VPEPSRGRGVP
54	Q07666	284	EPSRGRGVPVR
55	Q07666	346	GAPAPRARTAG
56	P08199	348	RTGTNRKFGYV
57	P08199	554	EGRTIRLELQG
58	P08199	599	GSVRARIVTDR
59	Q28165	23	GSGPGRRRHLV
60	O75925	248	PKRPSRPINIT
61	O75925	347	LTIPCRALTCS
62	O75925	417	TWAPMRSKKEV
63	O75925	507	ASPVSRTPSLP
64	Q96PU8	53	EISRVRKDMYN
65	Q96PU8	139	KEEQNRGKPNW
66	Q96PU8	205	LNGTYRDANIK
67	Q96PU8	256	IMPLIRQIQTA
68	Q96PU8	320	ATKVRRHDMRV
69	Q96PU8	324	RRHDMRVHPYQ
70	Q92804	216	NFGGHRDYGPR
71	Q92804	334	GSGGGRRGRGG
72	Q92804	335	SGGGRRGRGGY
73	Q92804	411	RGRGGRGGDRG
74	P09867	122	EEHHLRDYFEQ
75	P09867	140	EIMTDRGSGKK
76	P09867	284	GNFGGRSSGPY
77	O00267	71	PPKKPRHGGFI

78	O00267	190	KIGEERATAIS
79	O00267	198	AISLMRKFIAY
80	O00267	610	GPHSGREGEIR
81	O00267	615	REGEIRHLFRS
82	O00267	619	IRHLFRSFAFL
83	O00267	767	TSTYGRTPMYG
84	O00267	989	IQVKVRDTYLD
85	P69697	49	GISYGRKKRRQ
86	Q15554	41	GGPAERGAGEA
87	Q15554	274	PREPARQLRNP
88	P15056	199	CCAVYRIQDGE
89	P15056	362	FGQRDRSSSAP
90	P15056	384	IDDLIRDQGFR
91	P15056	435	SSSEDRNRMKT
92	Q5TAQ9	65	TSTESRGTDTE
93	Q5TAQ9	94	INDENRVHDRS
94	Q5TAQ9	117	EQPRRRVQRKR
95	Q5TAQ9	125	RKRANRDQDSS
96	Q5TAQ9	134	SSDDERALEDW
97	Q5TAQ9	149	TSALPRPRWQA
98	Q5TAQ9	159	ALPALRERELG
99	Q96EY1	25	PAISGRGARPP
100	Q96EY1	340	KSPVFRRDGAD
101	Q96EY1	341	SPVFRRDGADI
102	P06746	182	VCGSFRRGAES
103	Q92997	73	PCFNGRVVSWL
104	Q92997	105	PPPMERTGGIG
105	Q92997	200	DDSTSRFSSST
106	Q92997	219	MRRHKRRRRKQ
107	Q60487	16	AAAARREPGGA
108	Q60487	96	GVCANRYLAMK
109	Q60487	144	YVALKRTGQYK
110	Q54G21	235	RKSAKRILVIP
111	Q54G21	613	VQTLHRLVEDN
112	Q54G21	673	VAQMRRQIIDL
113	Q54G21	746	YALKIRAFKKS
114	Q54G21	1031	KDVIKRVTVEY
115	Q9R1E0	222	HSKFIRVQNEG
116	Q9R1E0	264	KFAKSRGRAAK
117	Q13283	32	PDMLHRFYGKN
118	Q13283	78	CHTKIRHVDAH
119	Q13283	302	PQRDQRVREQR
120	Q13283	304	RDQRVREQRIN
121	Q13283	317	PQRGPRPIREA
122	Q13283	331	GDIEPRRMVRH
123	Q13283	332	DIEPRRMVRHP
124	Q13283	370	NVVELRINSGG
125	P31943	49	RFIYTREGRPS
126	P31943	199	HYDPPRKLMAM
127	P04264	12	SRSGYRSGGGF
128	P04264	29	IINYQRRTTSS
129	P04264	239	FINNLRRRVDQ

130	P08729	13	PVFTSRSAAFS
131	P02687	129	FGYGGRASDYK
132	P02687	158	FKLGGRDSRSG
133	P15720	52	DRHVPRRGFGK
134	P15720	112	GLSLTRFSWGG
135	P25274	111	GTVLSRFSWGA
136	P11940	49	RDMITRRSLGY
137	P11940	67	PADAERALDTM
138	P11940	83	KGKPVRIMWSQ
139	P11940	94	RDPSLRKSGVG
140	P11940	240	FVSFERHEDAQ
141	Q9JMB7	8	GRARARARGRA
142	Q9JMB7	10	ARARARGRARG
143	Q9JMB7	53	GRGRQRGMVVG
144	Q9JMB7	95	LGVNTRQNLDH
145	Q9JMB7	193	NGEHVRITITL
146	Q8CDG1	21	PSQCVRMPGCW
147	Q8CDG1	31	WPQAPRPLEPA
148	Q8CDG1	50	RGLVFRKPEDS
149	Q8CDG1	206	VLTMERKEKEL
150	Q86WR7	186	PVEHPRLLRSV
151	P22626	62	ASKRSRGFGFV
152	P22626	99	KRAVAREESGK
153	P22626	147	EIITDRQSGKK
154	P51991	167	QSGKKRGFAFV
155	Q99729	28	VPEASRGRGWT
156	P14678	16	QHIDYRMRCIL
157	P14678	73	GLVLLRGENLV
158	P14678	108	GPGIGRAAGRG
159	P14678	209	GIPPGRGTPMG
160	Q14524	34	AEKQARGSTTL
161	Q14524	43	TLQESREGLPE
162	Q14524	222	ALRTFRVLRAL
163	Q14524	225	TFRVLRALKTI
164	Q14524	282	RHKCVRNFTAL
165	P23246	9	DRFRSRGGGGG
166	P23246	267	GGPGGRSEEKI
167	P23246	315	EDEFKRLFAKY
168	P23246	342	IKLESRALAEI
169	P68363	221	NLDIERPTYTN
170	P07437	262	MVPFPRLHFFM
171	Q86V81	29	GGGRGRGRAGS
172	Q86V81	73	GGGRNRPAPYS
173	Q86V81	155	DVHFERKADAL
174	Q86V81	204	GMTRNRGAGGF
175	O08583	38	GSQGGRGGAVQ
176	O08583	188	QIDTQRRPAQS
177	O08583	196	AQSINRGGMTR
178	P31269	106	AAPDGRYMRSW
179	P31269	109	DGRYMRSWLEP
180	P31269	248	LNLTERQVKIW
181	P31269	258	WFQNRRMKMKK

182	P04049	316	PVPAQRERAPV
183	O76922	11	NPVIARGRGRG
184	O76922	26	NVEANRGFAPS
185	O76922	189	LVTKSRAGENI

(4) Subset 4 (185 samples)

Sample #	Protein ID	Site	Sequences
1	Q9NR22	95	LKDEVRTLTYR
2	Q9NR22	204	TVIFARDKWLK
3	Q9NR22	218	LMFPDRAALYV
4	Q9NR22	300	CLQIQRNDYVH
5	Q9UBL3	108	DEENGRQLGEV
6	Q9UBL3	160	NTYFLRKQANL
7	Q92934	109	LWAAQRYGREL
8	Q7Q2B7	261	KMYPSRGDLHV
9	P11911	125	RNPVPRPFLDM
10	P11911	160	TLLLFRKRWQN
11	Q09472	237	GQTGLRGPQPL
12	Q09472	353	AHKCQRREQAN
13	Q09472	466	PSSIERAYAAL
14	Q09472	754	MGYGPRMQQPS
15	Q09472	883	LHPPPRQTPTP
16	Q09472	915	PQQQPRSQQST
17	Q09472	1055	KPEELRQALMP
18	Q09472	1137	NRKTSRVYKYC
19	Q09472	1166	GYCCGRKLEFS
20	P03372	162	RRQGGRERLAS
21	P03372	243	QACRLRKCYEV
22	P03372	259	GIRKDRRGGRM
23	P03372	277	DDGEGRGEVGS
24	P03372	300	PLMIKRSKKNS
25	P03372	394	IGLVWRSMEHP
26	P03372	434	LATSSRFRMMN
27	P03372	548	MLDAHRLHAPT
28	Q01844	138	ATAPTRPQDGN
29	Q01844	461	GGLPPREGRGM
30	Q01844	518	GNVQHRAGDWQ
31	P22509	80	GRGGGRGGKRG
32	P22509	213	NLAKKRTNIIP
33	P22509	250	QPDQTRIVALN
34	P35637	371	VSFATRRADFN
35	P35637	472	NYGDDRRGGRG
36	P35637	514	GKMDSRGEHRQ
37	P03347	91	YCVHQRIEIKD
38	P03347	150	QAISPRTLNAW
39	P03347	380	TIMMQRGNFRN
40	P03347	464	PFLQSRPEPTA
41	P03347	476	PEESFRSGVET
42	P68431	70	KLPFQRLVREI
43	P62805	36	TKPAIRRLARR

44	P62805	41	RRLARRGGVKR
45	P62805	56	IYEETRGVLKV
46	P17096	73	GAAKTRKTTTT
47	P17096	86	RKPRGRPKKLE
48	P31942	116	PGPYDRPIGGR
49	P31942	137	MYDRMRRGGDG
50	P31942	232	IGADGRATGEA
51	O60506	60	SDLDERAIEAL
52	O60506	286	DKKKNRGFCFL
53	O60506	343	KVLFVRNLANT
54	O60506	520	APPRGRAGYSQ
55	Q07666	17	SRSSGRSGSMD
56	Q07666	213	KEEELRKGGDP
57	Q07666	289	RGVPVRGRGAA
58	Q07666	354	TAGIQRIPLPP
59	P08199	281	PAPGKRKKEMT
60	P08199	471	GEKGQRQERTG
61	P08199	551	MEIEGRTIRLE
62	P08199	597	FEGSVRARIVT
63	Q28165	24	SGPGRRRHLVP
64	Q28165	125	EAIKARVREME
65	O75925	28	LGYAGRNKHGR
66	O75925	163	SDNSQRFRETC
67	O75925	296	TVLLQRLRAKG
68	O75925	330	ATTSLRVSLLC
69	O75925	632	HTVTNRSSTDT
70	Q96PU8	43	FNHLERLLDEE
71	Q96PU8	101	FNFVGRILGPR
72	Q96PU8	330	VHPYQRIVTAD
73	Q96PU8	336	IVTADRAATGN
74	Q92804	153	SYHSQRENYSH
75	Q92804	169	RRDVSRYGEDN
76	Q92804	187	GGGRGRGGYDK
77	Q92804	195	YDKDGRGPMTG
78	Q92804	326	RPEFMRGGGSG
79	Q92804	373	MNFARRNSCNQ
80	Q92804	395	SGGDFRGRGYG
81	Q92804	441	GYSGDRSGGGY
82	Q92804	526	GYGGDRSRGGY
83	P09867	53	DPNTKRSRGFG
84	P09867	178	HNCEVRKALSK
85	P09867	218	NDNFGRGGNFS
86	O00267	123	DRSGARRLQNL
87	O00267	133	LWRDQREEELG
88	O00267	246	GVGNLRLGYWN
89	O00267	334	RKKFKRPPQRL
90	O00267	416	STGKEREHNFQ
91	O00267	747	TISVDRQRLTT
92	Q15554	25	SRSSGRARRGR
93	Q15554	66	FHEALRAFRGS
94	Q15554	89	QALLVRPLGKE
95	Q15554	200	LLNIIREKNLA

96	Q15554	270	VEKPPREPARQ
97	Q15554	361	RMTISRLVLEE
98	Q15554	490	VMIKDRWRTMK
99	Q5TAQ9	116	EEQPRRRVQRK
100	Q96EY1	8	ARCSTRWLLVV
101	Q96EY1	28	SGRGARPPREG
102	Q96EY1	319	DGQTVRMPVGK
103	Q96EY1	325	MPVGKREIFIT
104	P06746	40	KYNAYRKAASV
105	P06746	112	GPSAARKFVDE
106	P06746	253	KEYPHRRIDIR
107	P06746	299	NEYTIRPLGVT
108	P06746	328	IQWKYREPKDR
109	Q92997	231	VSRIERSSSFS
110	Q60487	63	IHPDGRVDGVR
111	Q60487	84	LQAEDRGVVSI
112	Q60487	131	NYNTYRSRKYS
113	Q9R1E0	21	PLPRQRSCTWP
114	Q9R1E0	98	AAAASRGLCGD
115	Q9R1E0	154	TSSSRRNAWGN
116	Q9R1E0	266	AKSRGRAAKKK
117	Q9R1E0	311	NWSTFRPRTSS
118	Q13283	299	PQRPQRDQRVR
119	Q13283	307	RVREQRINIPP
120	Q13283	335	PRRMVRHPDSH
121	P31943	81	ETMGHRYVEVF
122	P31943	114	NDGFVRLRGLP
123	P31943	224	YNSIGRGAGFE
124	P31943	232	GFERMRRGAYG
125	P31943	262	SDRFGRDLNYC
126	P04264	179	QKVKSREREQI
127	P04264	240	INNLRRRVDQL
128	P04264	241	NNLRRRVDQLK
129	P04264	403	ISELNRVIQRL
130	P08729	25	RGAQVRLSSAR
131	P08729	88	SLQRVRQEESE
132	P08729	161	QVDGGRLEAEL
133	P08729	226	EINFLRTLNET
134	Q36736	21	DQEFNRKMQEQ
135	P02687	9	KRPSQRSKYLA
136	P02687	63	GHHAARTTHYG
137	P15720	53	RHVPRRGFGKD
138	P25274	63	KDHAARTTHYG
139	P25274	77	QKSGHRPQDEN
140	Q8IZS5	91	GNEYGRNYFDP
141	Q8IZS5	118	REDDDRIFYNR
142	Q8IZS5	221	VTAVIRSRRYY
143	Q8IZS5	223	AVIRSRRYYRE
144	P11940	44	SIRVCRDMITR
145	P11940	172	KVFVGRFKSRK
146	Q9JMB7	125	FRLTSRPQWAL
147	Q8CDG1	39	EPAWGRAGPAG

148	Q8CDG1	108	SANMVRKDREE
149	Q8CDG1	115	DREEPRSSLPD
150	Q86WR7	231	RTPAARGPRSG
151	Q86WR7	268	AAREPRRTLSR
152	P51991	16	QPDSGRRRRRR
153	P51991	19	SGRRRRRRGEE
154	P51991	20	GRRRRRRGEEG
155	Q99729	194	PKLNKRRGFVF
156	Q99729	322	GKSQRRGGHQN
157	P14678	94	DTGIARVPLAG
158	P14678	112	GRAAGRGIPAG
159	Q14524	219	NVSALRTFRVL
160	Q14524	376	QDCWERLYQQT
161	Q14524	433	EEKEKRFQEAM
162	P23246	32	GLHDFRSPPPG
163	P23246	287	LSLLRRPGEKT
164	P23246	298	YTQRCRLFVGN
165	P23246	407	VIVDDRGRSTG
166	P07437	62	GKYVPRAILVD
167	P07437	390	FTAMFRRKAFL
168	Q86V81	31	GRGRGRAGSQG
169	Q86V81	47	AQAAARVNRGG
170	O08583	50	AARVNRGGGPM
171	P39748	29	KSYFGRKVAID
172	P39748	47	FLIAVRQGGDV
173	P39748	261	IEEIVRRLDPN
174	P39748	320	QFSEERIRSGV
175	P31269	210	STRKKRCPYTK
176	P31269	257	IWFQNRRMKMK
177	P04049	73	TVVNVRNGMSL
178	P04049	111	KGKKARLDWNT
179	P04049	218	TMRRMRESVSR
180	P04049	256	LSQRQRSTSTP
181	P46783	65	EQFAWRHFYWY
182	P46783	119	PARLTRGEADR
183	O76922	96	DLVYSRPPGMT
184	O76922	248	NLENFRMQLWP
185	O76922	291	LSDAIRDSDDY

(5) Subset 5 (185 samples)

Sample #	Protein ID	Site	Sequences
1	Q9NR22	251	DMTCIRDVAMK
2	Q9UBL3	213	MTTRQRPGKMT
3	Q9UBL3	621	TEVDGRRSPPW
4	Q92934	20	SSSAERGLGPS
5	Q92934	115	YGRELRRMSDE
6	Q7Q2B7	146	TSTYQRAIYNN
7	Q7Q2B7	239	MLYNERMLETY
8	Q7Q2B7	308	MKEYFRQPIVD
9	Q7Q2B7	326	MAKSIRHTTNF

10	P11911	30	GCQALRVEGGP
11	P11911	55	CENNGRNPNIT
12	P11911	120	TYLRVRNPVPR
13	Q09472	200	SIGAGRGRQNM
14	Q09472	362	ANGEVRQCNLP
15	Q09472	424	NAGDKRNQQPI
16	Q09472	613	LVAYARKVEGD
17	Q09472	648	ELEEKRRTRLQ
18	Q09472	651	EKRRTRLQKQN
19	Q09472	695	DPSMIRGSVPN
20	Q09472	728	PPIVPRQTPPL
21	Q09472	838	SPVPSRTPTPH
22	Q09472	1067	LEALYRQDPES
23	Q09472	1234	EQFSKRKNDTL
24	Q09472	1281	LKKSARTRKEN
25	Q09472	1305	TFLENRVNDFL
26	Q09472	1311	VNDFLRRQNHP
27	Q09472	1312	NDFLRRQNHPE
28	Q09472	1391	PPPNQRRVYIS
29	Q09472	1405	SVHFFRPKCLR
30	P03372	211	KAFFKRSIQGH
31	P03372	233	TIDKNRRKSCQ
32	P03372	412	NLLLDRNQGKC
33	P03372	477	KDHIHRVLDKI
34	P03372	555	HAPTSRGGASV
35	Q01844	446	KVSLARKKPPM
36	Q01844	648	DKGEHRQERRD
37	Q01844	651	EHRQERRDRPY
38	P22509	64	GGGGGRGGGFQ
39	P22509	128	VYGEKRVSISE
40	P22509	204	SHRSGRDLINL
41	P22509	310	LEPYERDHAVV
42	P03347	15	GGELDRWEKIR
43	P03347	58	TSEGCRQILGQ
44	P03347	361	PGHKARVLAEA
45	P03347	406	TARNCRAPRKK
46	P03347	444	PSYKGRPGNFL
47	P17096	30	GRGRPRKQPPV
48	P31942	80	GKHKERIGHRY
49	P31942	200	HFVHMRGLPFR
50	O60506	142	VTTGQRKYGGP
51	O60506	192	PIWDLRLMMDP
52	O60506	203	LTGLNRGYAFV
53	O60506	366	FGKLERVKKLK
54	O60506	448	TRGRGRGGRGG
55	O60506	501	RGRGGRGARGA
56	O60506	546	GAQQQRGRGVR
57	Q07666	186	GNTIKRLQEET
58	Q07666	302	PPPVPRGRGVG
59	P08199	561	ELQGPRGSPNA
60	P08199	567	GSPNARSQPSK
61	Q28165	227	DKESVRTSLAL

62	Q28165	240	SLFRGRQIKVI
63	O75925	165	NSQRFRETCFA
64	Q96PU8	51	DEEISRVRKDM
65	Q96PU8	68	GSTEKRSAELP
66	Q96PU8	130	GKGSMRDKKKE
67	Q92804	337	GGRRGRGGYRG
68	Q92804	343	GGYRGRGGFQG
69	Q92804	545	GYGGDRSGGYG
70	P09867	75	AAMNARPHKVD
71	P09867	97	REDSQRPGAHL
72	P09867	146	GSGKKRGFAFV
73	O00267	119	VLDEDRSGARR
74	O00267	469	PAQELRKYFKM
75	O00267	552	VGVIVRLERET
76	O00267	555	IVRLERETFQV
77	O00267	668	APMSPRISSPM
78	O00267	700	SRGRGRRDNEL
79	O00267	701	RGRGRRDNELI
80	O00267	757	TVGSRRPGGMT
81	O00267	1005	QTGVIRSVTGG
82	P69697	55	KKRRQRRRPPQ
83	Q15554	27	SSGRARRGRHE
84	Q15554	99	EHTVSRLLRVM
85	Q15554	109	MQCLSRIEEGE
86	Q15554	492	IKDRWRTMKRL
87	P15056	95	DALQQREQQLL
88	P15056	178	CGVTVRDSLKK
89	P15056	443	MKTLGRRDSSD
90	Q5TAQ9	98	NRVHDRSEEEE
91	Q5TAQ9	122	RVQRKRANRDQ
92	Q5TAQ9	225	WDWVRRQPVLD
93	Q96EY1	41	GAWLSRKLSVP
94	Q96EY1	64	ALLTLRPGVSL
95	Q96EY1	151	SDEVKRKQYDA
96	Q96EY1	277	RSTCRRCGGRG
97	P06746	149	GDFEKRIPREE
98	P06746	254	EYPHRRIDIRL
99	Q92997	113	GIGDSRPPSFH
100	Q92997	149	ERPRRRDGPEH
101	Q92997	168	KGERRREPGGY
102	Q92997	216	SRLMRRHKRRR
103	Q92997	221	RHKRRRRKQKV
104	Q60487	121	CFFFERLESNN
105	Q60487	133	NTYRSRKYSSW
106	Q54G21	194	LENNQRVGSLF
107	Q54G21	210	SETPFRFVVGE
108	Q9R1E0	29	TWPLPRPEFNQ
109	Q9R1E0	606	GMFIERLDCDM
110	Q13283	17	GREFVRQYYTL
111	Q13283	132	HNDIFRYQDEV
112	Q13283	247	VQEDLRTFSWA
113	Q13283	314	NIPPQRGPRPI

114	P31943	75	ALKKDRETMGH
115	P31943	175	KKHKERIGHRY
116	P04264	8	RQFSSRSGYRS
117	P04264	38	SSSTRRSGGGG
118	P04264	181	VKSREREQIKS
119	P04264	223	VDTSTRTHNLE
120	P04264	267	MVEDYRNKYED
121	P04264	277	DEINKRTNAEN
122	P08729	136	SAKSSRLPDIF
123	P08729	302	HGDDLRNTRNE
124	P02687	31	FLPRHRDTGIL
125	P02687	96	NIVTPRTPPPS
126	P15720	30	RHGSPRHRDSG
127	P15720	42	LDSLGRFFGGD
128	P25274	150	QGTLSRLFKLG
129	Q8IZS5	132	LFDESRQGEPQ
130	Q8IZS5	195	LEQEIRSTSLH
131	P11940	265	QIYVGRAQKKV
132	Q9JMB7	80	LSLAERGGRRR
133	Q9JMB7	85	RGGRRRDFHDL
134	Q9JMB7	121	STNHFRLTSRP
135	Q9JMB7	174	LFLPKRLQHKV
136	Q86WR7	24	PSCRLRAFSRG
137	Q86WR7	40	RSSSSRSRSFT
138	Q86WR7	42	SSSRSRSFTLD
139	Q86WR7	172	TPDPPRRELRA
140	Q86WR7	234	AARGPRSGDPG
141	Q86WR7	269	AREPRRTLSRA
142	Q86WR7	273	RRTLSRAAVSV
143	P22626	38	TEESLRNYYEQ
144	P22626	89	HSIDGRVVEPK
145	P51991	18	DSGRRRRRRGE
146	P51991	68	DCVVMRDPQTK
147	P51991	74	DPQTKRSRGFG
148	P51991	246	GNFGGRGGYGG
149	Q99729	137	HRLDGRVIDPK
150	Q99729	171	TEEKIREYFGE
151	Q99729	321	YGKSQRRGGHQ
152	P14678	25	ILQDGRIFIGT
153	P14678	61	SKQAEREEKRV
154	P14678	65	EREEKRVLGLV
155	P14678	132	LAGPVRGVGGP
156	Q14524	104	GKTIFRFSATN
157	Q14524	190	AFTFLRDPWNW
158	Q14524	383	YQQTLRSAGKI
159	Q14524	451	EALTIRGVDTV
160	P23246	358	DDTPMRGRQLR
161	P68363	79	VIDEVRTGTYR
162	P68363	123	VLDRIRKLADQ
163	P68363	214	IYDICRRNLDI
164	P68363	402	LMYAKRAFVHW
165	P07437	156	LISKIREEYPD

166	P07437	320	AVFRGRMSMKE
167	Q86V81	58	GPIRNRPAIAR
168	Q86V81	71	AGGGGRNRPAP
169	Q86V81	141	AVHYDRSGRSL
170	Q86V81	190	IDAQRRPAQSV
171	O08583	70	AAGGGRNRPAP
172	O08583	201	RGGMTRNRGSG
173	P39748	103	AKRSERRAEAE
174	P39748	129	EKFTKRLVKVT
175	P39748	262	EEIVRRLDPNK
176	P31269	235	YLTRDRRYEVA
177	P04049	215	PSLTMRRMRES
178	P04049	216	SLTMRRMRESV
179	P46783	124	RGEADRDTYRR
180	P46783	153	TEFQFRGGFGR
181	O76922	80	SEGDPRGSVRG
182	O76922	132	TIYQYRVDFTP
183	O76922	145	EATRLRRSFLY
184	O76922	221	NLILRRAMEGL
185	O76922	233	LKLVSRYYYDP

(6) Subset 6 (186 samples)

Sample #	Protein ID	Site	Sequences
1	Q9NR22	100	RTLTYRNSMYH
2	Q9UBL3	300	GRGAKRKQQDG
3	Q9UBL3	342	NKDGYRYILAE
4	Q9UBL3	388	LALHDRAPQLK
5	Q92934	39	SGKHHRQAPGL
6	Q92934	70	GAVEIRSRHSS
7	Q92934	98	FRGRSRSAPPN
8	Q92934	161	DRNLGRGSSAP
9	Q7Q2B7	112	SVFNLRTEDSS
10	Q7Q2B7	140	MQDFVRTSTYQ
11	Q09472	202	GAGRGRQNMQY
12	Q09472	397	HCASSRQIISH
13	Q09472	568	STTGIRKQWHE
14	Q09472	603	AALKDRRMENL
15	Q09472	705	NQMMPRITPQS
16	Q09472	1356	ESFPYRTKALF
17	P03372	157	PNSDNRRQGGR
18	P03372	271	KHKRQRDDGEG
19	P03372	287	SAGDMRAANLW
20	P03372	335	EYDPTRPFSEA
21	Q01844	292	GPGENRSMSGP
22	P22509	141	DKIEYRAWNPF
23	P22509	201	VEFSHRSGRDL
24	P22509	261	AHTFLRNGGHF
25	P22509	320	VVGVYRPPPKA
26	P35637	218	RGGRGRGGSGG
27	P35637	441	MNFSWRNECNQ

28	P03347	39	IVWASRELERF
29	P03347	43	SRELERFAVNP
30	P03347	76	GSEELRSLYNT
31	P03347	214	AAEWDRVHPVH
32	P03347	229	APGQMREPRGS
33	P03347	232	QMREPRGSDIA
34	P62805	20	GAKRHRKVLRD
35	P62805	93	VYALKRQGRTL
36	P17096	81	TTTPGRKPRGR
37	P31942	55	MDYQGRSTGEA
38	P31942	104	FYDPPRRLLGQ
39	P31942	110	RLLGQRPGPYD
40	P31942	169	DGFDDRMRDGR
41	O60506	130	KALLERTGYTL
42	O60506	171	VGKIPRDLFED
43	O60506	244	SVANNRLFVGS
44	O60506	304	AAQARRRLMSG
45	O60506	413	PPDQKRKERKA
46	O60506	478	DYHNYRGGYED
47	O60506	548	QQQRGRGVRGA
48	O60506	551	RGRGVRGARGG
49	Q07666	36	RQTPSRQPPLP
50	Q07666	159	MKLKERVLIPV
51	Q07666	348	PAPRARTAGIQ
52	Q07666	420	DWNGTRPSLKA
53	Q07666	429	KAPPARPVKGA
54	P08199	385	EKPKGRDSKKV
55	Q28165	76	EEEPPRPRAPP
56	Q28165	127	IKARVREMEEE
57	Q28165	251	PKRTNRPGIST
58	O75925	15	MVMSLRVSELQ
59	O75925	201	VQVQLRFCLSE
60	O75925	245	GVEPKRPSRPI
61	O75925	298	LLQRLRAKGIR
62	O75925	309	NPDHSRALIKE
63	O75925	341	PLGKMRLTIPC
64	O75925	622	SSNSLRESHSH
65	Q96PU8	170	EIKLKRAVEEV
66	Q96PU8	319	VATKVRRHDMR
67	Q92804	403	GYGGERGYRGR
68	Q92804	408	RGYRGRGGRGG
69	P09867	14	EPEQLRKLFIG
70	P09867	55	NTKRSRGFGFV
71	P09867	92	KRAVSREDSQR
72	P09867	232	GFGGSRGGGGY
73	O00267	310	LKMIPRIDYDR
74	O00267	365	IFEGNRYSRKG
75	O00267	484	KVIAGRFEGDT
76	O00267	733	TESTARVELHS
77	O00267	749	SVDRQRLTTVG
78	O00267	756	TTVGSRRPGGM
79	P69697	57	RRQRRRPPQGS

80	Q15554	21	GRRASRSSGRA
81	Q15554	80	DFRQIRDIMQA
82	Q15554	223	QQKMLRFLESH
83	Q15554	277	PARQLRNPPTT
84	Q15554	332	KNKRPRKDENE
85	P15056	146	PTDVARSNPKS
86	P15056	252	FCDFCRKLLFQ
87	P15056	271	YKFHQRCSTEV
88	P15056	354	ADEDHRNQFGQ
89	P15056	437	SEDRNRMKTLG
90	Q5TAQ9	115	EEEQPRRRVQR
91	Q5TAQ9	168	LGSSARFVYEA
92	P06746	183	CGSFRRGAESS
93	P06746	283	FNKNMRAHALE
94	Q92997	39	KGVLQRPSYKF
95	Q92997	143	LVSAQRERPRR
96	Q92997	147	QRERPRRRDGP
97	Q92997	157	PEHATRLNGTA
98	Q92997	166	TAKGERRREPG
99	Q92997	222	HKRRRRKQKVS
100	Q92997	228	KQKVSRIERSS
101	Q60487	57	GGFFLRIHPDG
102	Q54G21	339	NESNSRNSHSN
103	Q54G21	631	VEKENRMVTLK
104	Q9R1E0	147	LAGQPRKTSSS
105	Q9R1E0	153	KTSSSRRNAWG
106	Q9R1E0	177	SSAEKRLTLSQ
107	Q9R1E0	211	WKNSIRHNLSL
108	Q13283	398	KVLSNRPIMFR
109	P31943	29	ADEVQRFFSDC
110	P31943	188	IFKSSRAEVRT
111	P04264	45	GGGGGRFSSCG
112	P04264	251	KSDQSRLDSEL
113	P04264	392	HGDSVRNSKIE
114	P08729	253	SMDNSRSLDLD
115	P02687	23	TMDHARHGFLP
116	P02687	52	RGAPKRGSGKD
117	P02687	78	KAQGHRPQDEN
118	P02687	161	GGRDSRSGSPM
119	P15720	6	MASQKRSSFRH
120	P15720	32	GSPRHRDSGLL
121	P15720	63	DIHAARASHVG
122	P15720	73	GSIPQRSQHGR
123	P11940	41	PILSIRVCRDM
124	P11940	176	GRFKSRKEREA
125	P11940	179	KSRKEREAELG
126	P11940	272	QKKVERQTELK
127	P11940	309	GIDDERLRKEF
128	Q9JMB7	12	ARARGRARGQE
129	Q8CDG1	45	AGPAGRGLVFR
130	Q8CDG1	293	ELKSQRKTDDA
131	Q86WR7	28	LRAFSRGGSLE

132	Q86WR7	35	GSLESRSSSSR
133	Q86WR7	218	PTSPFREGRPG
134	Q86WR7	226	RPGEWRTPAAR
135	P22626	12	TVPLERKKREK
136	P22626	82	AAMAARPHSID
137	P22626	129	EEHHLRDYFEE
138	P22626	153	QSGKKRGFGFV
139	P22626	190	RKALSRQEMQE
140	P51991	17	PDSGRRRRRRG
141	P51991	76	QTKRSRGFGFV
142	P51991	161	EVMEDRQSGKK
143	Q99729	110	NTGRSRGFGFI
144	Q99729	133	DQKEHRLDGRV
145	P14678	49	DCDEFRKIKPK
146	P14678	181	PPPMGRGAPPP
147	P14678	196	PPPGMRPPMGP
148	Q14524	8	NFLLPRGTSSF
149	Q14524	14	GTSSFRRFTRE
150	Q14524	15	TSSFRRFTRES
151	Q14524	122	FHPIRRAAVKI
152	P23246	236	PKPPHRGGGEP
153	P23246	286	NLSLLRRPGEK
154	P23246	296	KTYTQRCRLFV
155	P23246	360	TPMRGRQLRVR
156	P23246	363	RGRQLRVRFAT
157	P23246	376	AALSVRNLSPY
158	P68363	84	RTGTYRQLFHP
159	P68363	121	DLVLDRIRKLA
160	P68363	156	SLLMERLSVDY
161	P68363	229	YTNLNRLISQI
162	P68363	422	EFSEAREDMAA
163	P07437	121	VLDVVRKEAES
164	P07437	251	LNADLRKLAVN
165	P07437	309	DPRHGRYLTVA
166	P07437	391	TAMFRRKAFLH
167	Q86V81	17	IIKLNRSQRGG
168	Q86V81	23	SQRGGRGGGRG
169	Q86V81	27	GRGGGRGRGRA
170	Q86V81	38	GSQGGRGGGAQ
171	Q86V81	50	AARVNRGGGPI
172	Q86V81	56	GGGPIRNRPAI
173	O08583	47	VQAAARVNRGG
174	P39748	327	RSGVKRLSKSR
175	P31269	40	LGQPPRQAATL
176	P31269	204	NWLHARSTRKK
177	P31269	236	LTRDRRYEVAR
178	P04049	40	QFGYQRRASDD
179	P04049	41	FGYQRRASDDG
180	P04049	89	KALKVRGLQPE
181	P04049	191	DWSNIRQLLLF
182	P04049	254	GSLSQRQRSTS
183	P46783	95	VPATLRRSRPE

184	P46783	103	RPETGRPRPKG
185	P46783	129	RDTYRRSAVPP
186	O76922	17	GRGRGRKPNNV

(7) Subset 7 (185 samples)

Sample #	Protein ID	Site	Sequences
1	Q9NR22	8	MKHSSRCLLLR
2	Q9NR22	349	DYLTVRRGEEI
3	Q9NR22	350	YLTVRRGEEIY
4	Q9UBL3	398	KISDDRLTVVG
5	Q9UBL3	461	FSYSWRSKKGT
6	Q7Q2B7	206	NNLTDRIIVIA
7	Q7Q2B7	318	DTFDIRICMAK
8	Q7Q2B7	391	HWYQVRCLLQT
9	Q7Q2B7	417	LAANQRQSYDV
10	Q7Q2B7	535	ISSTGRQQQQQ
11	P11911	111	NNILKRSCGTY
12	Q09472	335	ADPEKRKLIQQ
13	Q09472	1252	CTECGRKMHQI
14	Q09472	1283	KSARTRKENKF
15	Q09472	1292	KFSAKRLPSTR
16	Q09472	1478	KAVSERIVHDY
17	P03372	151	PPAFYRPNSDN
18	P03372	164	QGGRERLASTN
19	P03372	263	DRRGGRMLKHK
20	P03372	352	TNLADRELVHM
21	P03372	363	INWAKRVPGFV
22	Q01844	268	QQSSFRQDHPS
23	Q01844	330	GSAGERGGFNK
24	Q01844	537	QNFAWRTECNQ
25	P22509	46	FGGGGRGRGGG
26	P22509	48	GGGRGRGGGGG
27	P22509	98	MVEPHRHEGVF
28	P22509	147	AWNPFRSKLAA
29	P22509	230	HPHKYRMLIAM
30	P35637	422	GGGQQRAGDWK
31	P03347	264	GEIYKRWIILG
32	P03347	286	SILDIRQGPKE
33	P03347	294	PKEPFRDYVDR
34	P03347	384	QRGNFRNQRKM
35	P68431	27	ATKAARKSAPA
36	P68431	53	ALREIRRYQKS
37	P17096	56	VPTPKRPRGRP
38	P31942	84	ERIGHRYIEIF
39	P31942	90	YIEIFRSSRSE
40	P31942	135	GSMYDRMRRGG
41	P31942	138	YDRMRRGGDGY
42	P31942	171	FDDRMRDGRGM
43	O60506	105	KTYRQREKQGT

44	O60506	416	QKRKERKAQRQ
45	O60506	420	ERKAQRQAAKN
46	O60506	498	VGARGRGGRGA
47	O60506	512	APSRGRGAAPP
48	O60506	533	GPGSARGVRGA
49	Q07666	56	SRGGARASPAT
50	P08199	391	DSKKVRAARTL
51	P08199	458	AEIDGRSVSLY
52	Q28165	172	MEADARSIYVG
53	O75925	33	RNKHGRKHELL
54	O75925	62	IKELYRRRFPQ
55	O75925	64	ELYRRRFPQKI
56	O75925	479	EPSAKRTCPSL
57	Q96PU8	124	CKIMVRGKGSM
58	Q96PU8	163	EDAQNRAEIKL
59	Q96PU8	227	AQAAPRIITGP
60	Q92804	175	YGEDNRGYGGS
61	Q92804	383	QCNEPRPEDSR
62	Q92804	397	GDFRGRGYGGE
63	P09867	47	DCVVMRDPNTK
64	O00267	124	RSGARRLQNLW
65	O00267	280	PKSWVRLKRGI
66	O00267	283	WVRLKRGIYKD
67	O00267	315	RIDYDRIKARM
68	O00267	329	DWFAKRKKFKR
69	O00267	338	KRPPQRLFDAE
70	O00267	494	TGLIVRVEENF
71	O00267	577	HQAVTRKKDNR
72	O00267	813	LHDGSRTPAQS
73	Q15554	54	EEAVNRWVLKF
74	Q15554	154	VVESSRKLVKE
75	Q15554	330	ALKNKRPRKDE
76	Q15554	482	YPFVNRTAVMI
77	P15056	158	QKPIVRVFLPN
78	P15056	166	LPNKQRTVVPA
79	P15056	172	TVVPARCGVTV
80	P15056	389	RDQGFRGDGGS
81	P15056	424	SPGPQRERKSS
82	P15056	444	KTLGRRDSSDD
83	Q5TAQ9	33	GAEEGRETSSG
84	Q5TAQ9	59	DGGPNRTSTES
85	Q5TAQ9	120	RRRVQRKRANR
86	Q5TAQ9	184	FVQRFRLQHGL
87	Q5TAQ9	224	VWDWVRRQPVL
88	Q96EY1	18	VVGTPRLPAIS
89	Q96EY1	31	GARPPREGVVG
90	Q92997	148	RERPRRRDGPE
91	Q60487	15	TAAAARREPGG
92	Q60487	68	RVDGVREKTDP
93	Q54G21	12	EEYSNRNAASS
94	Q54G21	320	VAKLGRATGFT
95	Q54G21	672	NVAQMRRQIID

96	Q54G21	759	DEKNARKSDIE
97	Q54G21	800	KLDMVRIKKRN
98	Q54G21	804	VRIKKRNDDKV
99	Q9R1E0	83	SEDFARAPGCV
100	Q9R1E0	324	STISGRLSPIM
101	Q13283	13	PLLVGREFVRQ
102	Q13283	63	QKEIHRKVMSQ
103	Q13283	320	GPRPIREAGEQ
104	P31943	44	GAQGIRFIYTR
105	P31943	116	GFVRLRGLPFG
106	P31943	150	VDFQGRSTGEA
107	P31943	206	LMAMQRPGPYD
108	P31943	212	PGPYDRPGAGR
109	P31943	259	GFGSDRFGRDL
110	P04264	86	ARGGGRGSGFG
111	P08729	77	LLAPLRLDADP
112	P08729	86	DPSLQRVRQEE
113	P08729	149	QIAGLRGQLEA
114	P08729	167	LEAELRSMQDV
115	Q36736	53	YEKFERMIKEH
116	P02687	29	HGFLPRHRDTG
117	P02687	47	FFGSDRGAPKR
118	P02687	112	GLSLSRFSWGA
119	P15720	25	TTDHARHGSPR
120	P25274	43	LDSIGRFFSSD
121	Q8IZS5	105	EEINPRQCATE
122	Q8IZS5	123	RIFYNRLTKLF
123	Q8IZS5	224	VIRSRRYYREQ
124	P11940	50	DMITRRSLGYA
125	P11940	290	QDRITRYQGVN
126	Q9JMB7	144	PLMEARRLRSA
127	Q9JMB7	187	VFSQTRNGEHV
128	Q9JMB7	218	YNIIFRRLLKI
129	Q8CDG1	83	LDTAFRPPSKR
130	Q8CDG1	140	SVGWSRMLGRG
131	Q8CDG1	144	SRMLGRGSSEV
132	Q8CDG1	181	ARDPPRLPQPP
133	Q8CDG1	255	ECKSMRFGMLK
134	Q8CDG1	326	YNVVFRRVMKL
135	Q86WR7	189	HPRLLRSVPTP
136	P22626	21	EKEQFRKLFIG
137	P22626	54	DCVVMRDPASK
138	P22626	95	VVEPKRAVARE
139	P22626	200	EVQSSRSGRGG
140	P22626	266	GYGGGRGGYGG
141	P22626	325	NFGGSRNMGGP
142	P51991	21	RRRRRRGEEGH
143	P51991	96	AAMCARPHKVD
144	P51991	214	SAGSQRGRGGG
145	P51991	216	GSQRGRGGGSG
146	Q99729	108	DPNTGRSRGFG
147	Q99729	195	KLNKRRGFVFI

148	Q99729	253	RNRGNRGSGGG
149	P14678	18	IDYRMRCILQD
150	P14678	172	QYPPGRGGPPP
151	Q14524	18	FRRFTRESLAA
152	Q14524	27	AAIEKRMAEKQ
153	Q14524	53	EEEAPRPQLDL
154	Q14524	121	PFHPIRRAAVK
155	Q14524	277	FMGNLRHKCVR
156	P23246	19	GGFHRRGGGGG
157	P23246	44	GLNQNRGPMGP
158	P23246	242	GGGEPRGGRQH
159	P23246	245	EPRGGRQHHPP
160	P23246	365	RQLRVRFATHA
161	P68363	64	GKHVPRAVFVD
162	P68363	373	LAKVQRAVCML
163	P68363	390	AEAWARLDHKF
164	P07437	46	DLQLDRISVYY
165	P07437	86	FGQIFRPDNFV
166	Q86V81	189	QIDAQRRPAQS
167	O08583	58	GPMRNRPAIAR
168	P39748	73	FYRTIRMMENG
169	P39748	245	GIGPKRAVDLI
170	P39748	322	SEERIRSGVKR
171	P39748	339	GSTQGRLDDFF
172	P31269	29	ELSVGRYAPGT
173	P31269	166	SPPVDREKQPS
174	P04049	67	LPNKQRTVVNV
175	P04049	164	LLNGFRCQTCG
176	P04049	275	LPVDSRMIEDA
177	P04049	331	EKNKIRPRGQR
178	P46783	98	TLRRSRPETGR
179	P46783	113	GLEGERPARLT
180	O76922	84	PRGSVRGRRLI
181	O76922	143	DVEATRLRRSF
182	O76922	220	LNLILRRAMEG
183	O76922	260	YQTSIRQHEND
184	O76922	278	CHKVMRTETLY
185	O76922	302	QSTFKRAVMGM
