# Supplementary material for: iMethyl-PseAAC: Identification of Protein Methylation Sites via a Pseudo Amino Acid Composition Approach
Source: Biomed Res Int. 2014 May 22;2014:947416. doi: 10.1155/2014/947416 (PMC4054830; doi:10.1155/2014/947416)
Supplement: Supplementary file 1 — S1: An analysis of the benchmark dataset used in Hu et al. (Biopolymers, 2011, 95, 763-771) S2: List of self-conflict samples in the benchmark dataset used in Hu et al. (Biopolymers, 2011, 95, 763-771) S3: Benchmark dataset used for studying the Arg-methylation. It contains 1,481 samples, of which 185 are positive and 1,296 negative. These data were extracted from UniProtKB/Swiss-Prot database (version UniProt release 2013_06). S4: Benchmark dataset used for studying the Lys-methylation. It contains 1,884 samples, of which 226 are positive and 1,518 negative. These data were extracted from UniProtKB/Swiss-Prot database (version UniProt release 2013_06). S5: Seven negative subsets for studying Arg-methylation. Each subset contains 185 negative samples randomly taken from the 1,296 negative samples in Online Supporting Information S3 except for the 6th subset, which contains 186 samples. None of the samples in one subset occurs in any other subset. S6: Seven negative subsets for studying Lys-methylation. Each subset contains 217 negative samples randomly taken from the 1,518 negative samples in Online Supporting Information S4 except for the 5th subset, which only contains 216 samples. None of the samples in one subset occurs in any other subset. S7: Independent dataset for studying the Arg-methylation. It contains 75 samples, of which 20 are positive and 55 negative. None of the samples listed here occurs in Online Supporting Information S3. S8: Independent dataset for studying the Lys-methylation. It contains 40 samples, of which 14 are positive and 26 negative. None of the samples listed here occurs in Online Supporting Information S4. S9: The code for encoding the peptides investigated in this paper. [file 947416.f1.zip › S6.pdf]

Online Supporting Information S6. Seven negative subsets for studying Lys-methylation. Each subset contains 217 negative samples randomly taken from the 1,518 negative samples in Online Supporting Information S4 except for the 5th subset, which only contains 216 samples. None of the samples in one subset occurs in any other subset.

(1) Subset 1 (217 samples)

Sample #	Protein ID	Site	Sequences
1	P05141	49	QITADKQYKGI
2	P05141	94	FAFKDKYKQIF
3	P05141	96	FKDKYKQIFLG
4	P05141	105	LGGVDKRTQFW
5	P05141	245	MQSGRKGTDIM
6	P05141	272	GKAFFKGAWSN
7	Q9Y232	73	DKRKNKKGKTE
8	Q9Y232	74	KRKNKKGKTEY
9	Q9Y232	157	ALVIGKDHESK
10	Q9Y232	162	KDHESKNSQLF
11	P00042	11	KGSEKKGATLF
12	P00042	17	GATLFKTRCLQ
13	P00042	48	GRQSGKAEGYS
14	P00042	60	TDANIKKAVEW
15	P26358	58	LQTEIKNQLCD
16	P26358	83	YLAKVKSLLNK
17	P26358	177	AKGPAKRKPQE
18	P26358	198	IKEEDKDQDEK
19	Q5VTE0	41	KRTIEKFEKEA
20	Q5VTE0	129	EAGISKNGQTR
21	P0CE47	238	ERGIIKVGEEV
22	O75367	117	ELLAKKRGSKG
23	P62807	12	SAPAPKKGSKK
24	P09988	123	VTIQPKDMQLA
25	P69150	19	AKAPRKQLASK
26	Q55BP0	30	SSFPSKPVNEV
27	Q55BP0	65	DLLIKKLPFQR
28	P61830	126	QKKDIKLARRL
29	P62803	32	IQGITKPAIRR
30	P04637	164	AMAIYKQSQHM
31	P04637	386	KKLMFKTEGPD
32	Q86L05	43	KNYDTKKDKRF
33	Q86L05	53	FSGQIKIGTVT
34	Q86L05	97	IGPKNKKAIKK
35	Q86L05	105	IKKLSKKYDAF
36	Q86L05	147	EDMASKINDVK
37	Q86L05	152	KINDVKSTVKF
38	P14909	34	VEKTKKIKIID
39	P14909	80	DELREKIAQYL
40	P14909	94	YGTDVKKEEVI
41	P14909	105	VTPGAKPALFL

42	P14909	142	KLLGGKPIYAN
43	P14909	194	PNDVKKIVDIS
44	P14909	257	GYIVAKREIIQ
45	P14909	285	QKAAVKAFDTF
46	P14909	313	YDELTKVKGVE
47	P39462	136	IVPHYKYMYKL
48	P39462	263	PKALAKQGKYV
49	P22498	76	QKMGLKIARLN
50	P22498	138	IFKDLKSRGLY
51	P22498	256	KSVSKKPVGII
52	P22498	445	RFGLLKVDYNT
53	P0C835	53	KYFRHKLPDDY
54	P80053	16	YTQQVKKLYKV
55	P80053	54	RGSDGKLKTFM
56	P80053	170	IKITGKVDFAV
57	P13123	52	EKDAPKELLDM
58	P61991	40	DEGGGKTGRGA
59	P26358	284	DEKDEKKHRSQ
60	P26358	291	HRSQPKDLAAK
61	O75530	398	VEDPHKAKCTT
62	O75530	400	DPHKAKCTTLT
63	Q5VTE0	371	AHIACKFAELK
64	P81542	20	DHAGHKVYAPA
65	P81542	32	PPVKEKALGIH
66	P00219	20	DHSGHKIYGPV
67	O43524	242	NPDGGKSGKAP
68	Q08369	311	PLAMRKEGIQT
69	P16403	110	FKLNKKAASGE
70	P16403	117	ASGEAKPKVKK
71	P16403	130	GTKPKKPVGAA
72	P16403	139	AAKKPKKAAGG
73	P10412	22	KTPVKKKARKS
74	P10412	34	GAAKRKASGPP
75	P10412	121	AKPKAKKAGAA
76	P10412	136	PAGAAKKPKKA
77	P10412	148	GAATPKKSAKK
78	P16401	21	EKSPAKKKATK
79	P16401	23	SPAKKKATKKA
80	P16401	35	GAGAAKRKATG
81	P16401	154	KKAVKKTPKKA
82	O75367	157	KKGARKSKKKQ
83	O75367	236	NTLEKKGGKEF
84	P40283	13	KPAEKKPASEK
85	P40283	33	KAPAEKKPKAG
86	P40283	52	AGGDKKKKMKK
87	Q41811	60	TRGVRKIFLEN
88	O14979	322	QQQQQKGGRGA
89	Q14103	231	CFITFKEEEPV
90	P16862	76	VEAHLKELNSI
91	P16862	197	LNRPQKAIAVM
92	P16862	525	AVNENKIVRKP
93	P16862	548	EAIQAKDFKRA

94	O00159	24	PHRPCKLALGS
95	O00159	274	KGQCAKVSSIN
96	O00159	285	DKSDWKVVRKA
97	O00159	486	DLVEEKFKGII
98	O00159	488	VEEKFKGIISI
99	O00159	595	ELSDKKRPETV
100	O00159	605	VATQFKMSLLQ
101	O00159	618	EILQSKEPAYV
102	P13538	85	SMNPPKYDKIE
103	P13538	207	ASGEKKKEEQS
104	P13538	260	FGATGKLASAD
105	P13538	354	KTAIYKLTGAV
106	Q96RI1	36	SMKPAKGVLTE
107	Q96RI1	196	ECRLRKCKEMG
108	Q96RI1	251	VTSTTKSCREK
109	Q96RI1	289	TNKILKEEFSA
110	Q96RI1	335	QIALLKGSAVE
111	Q96RI1	474	RVNDHKFTPLL
112	P11940	30	AMLYEKFSPAG
113	P11940	113	KSIDNKALYDT
114	P11940	157	ERAIEKMNGML
115	P11940	277	RQTELKRKFEQ
116	P06400	8	PKTPRKTAATA
117	P06400	80	WLTWEKVSSVD
118	P06400	122	FTELQKNIEIS
119	P06400	143	IDTSTKVDNAM
120	P06400	164	FALFSKLERTC
121	P06400	228	LDYFIKLSPPM
122	P06400	327	EEIYLKNKDLD
123	P06400	341	FLDHDKTLQTD
124	P0CX43	91	SVDDLKKLNKN
125	P47914	47	NMRFAKKHNKK
126	P47914	55	NKKGLKKMQAN
127	Q969Q0	6	MVNVPKTRRTF
128	Q969Q0	58	KPIFRKKAKTT
129	Q969Q0	59	PIFRKKAKTTK
130	Q969Q0	89	AIKRCKHFELG
131	P0CX27	76	RLECVKCKTRA
132	P0CX27	89	TLKRCKHFELG
133	P02393	91	GLKEAKDKVDG
134	P02393	114	EAEEAKKQLVE
135	P07472	98	APATLKEGMSK
136	Q12962	212	YGINVKKPHYF
137	Q04207	122	GIQCVKKRDLE
138	Q04207	221	CDKVQKEDIEV
139	Q04207	301	HRIEEKRKRTY
140	Q04207	425	SAPVPKSTQAG
141	Q53688	12	RLQLNKNFNFG
142	E5KWG9	29	TASHDKVWPIL
143	E5KWG9	135	VSELVKLPSIP
144	E5KWG9	278	IASTPKGEYHK
145	E5KWG9	283	KGEYHKIASYY

146	E5KWG9	292	YYQWQKRVEKL
147	B6A879	93	TTADVKPTENI
148	B6A879	173	DGGGSKEEERG
149	B6A879	290	LRELQKKAHAM
150	Q9L5X8	126	FAHGLKPIVVI
151	Q9L5X8	438	MAPDGKGRVRM
152	Q6NC90	9	HIDPTKEVFAQ
153	Q6NEZ3	66	YKFNQKSNFSA
154	Q6NEZ3	108	GYKNAKLTKSS
155	Q6NEZ3	120	RINVGKNVKLD
156	Q6NEZ3	178	TFIPEKTILTN
157	Q6NEZ3	341	VMPGEKVSVSA
158	Q9UKV3	103	LVKRLKGALML
159	P18281	16	QGAALKHAETV
160	P18281	34	ENVTVKKVDRS
161	P18281	52	KPHELKHAETV
162	Q9SJU4	246	YDVAEKVWAEV
163	Q9ZU52	120	LYQSTKDGKTF
164	Q9ZU52	123	STKDGKTFVDC
165	Q9ZU52	142	GIKVDKGLSPL
166	Q9ZU52	171	SAEYYKQGARF
167	P62157	22	FSLFDKDGDGT
168	P11118	76	TLMSRKMHDTD
169	P07463	76	SLMARKMKEQD
170	P04573	43	MDMLTKLGQTY
171	P04573	133	VEEAMKEADED
172	P00889	208	MDLIAKLPCVA
173	P00063	16	PAAGEKIFKTK
174	P00063	107	LIAYLKQSTAA
175	P00078	19	AARGEKLFKGR
176	P00077	19	AAKGEKIFKGR
177	P00077	22	GEKIFKGRAAQ
178	P00077	98	FAGIKKPQERA
179	P00043	60	YTDANKKKGVE
180	P00076	7	DAERGKKLFES
181	P00076	65	EETLHKFLENP
182	P00076	78	YVPGTKMAFAG
183	P00047	13	PGDASKGANLF
184	P00047	97	GLKKNKDRNDL
185	Q71V39	179	VSAYIKKIGYN
186	P02993	172	FEEIKKEVSAY
187	P02994	177	TSNFIKKVGYN
188	P02994	217	WEKETKAGVVK
189	Q96KQ7	139	MTGAGKSPPSV
190	P53991	49	TTDMSKRTVPT
191	P53991	55	RTVPTKLEEGE
192	P53991	69	NTYSNKAPFKA
193	P53991	81	VRSVEKITGPK
194	P53991	164	DPETGKEDPAK
195	P11416	94	FVCQDKSSGYH
196	P11416	166	DRNKKKKEAPK
197	P11416	360	LLEALKVYVRK

198	P27064	227	AEAIFKSQAET
199	P0A7J7	92	KSGSGKPNKDK
200	Q5SLP6	9	VVAVVKLQLPA
201	Q5SLP6	16	QLPAGKATPAP
202	Q5SLP6	70	FTFVTKTPPAS
203	P0CX53	41	GLSPKKVGEDI
204	P62986	114	NCRKKKCGHTN
205	P55072	20	AILKQKNRPNR
206	P55072	190	EGEPIKREDEE
207	P55072	211	IGGCRKQLAQI
208	P55072	231	HPALFKAIGVK
209	P05547	21	DIDRKKAEVRK
210	P05547	26	KAEVRKRLEEQ
211	P05547	46	MTPERKKKLRL
212	O95785	54	YLPVTKEGPRD
213	O95785	128	PRLLEKHAQGR
214	O95785	277	CSIYFKQKEHL
215	O95785	517	DFPLSKPLLHG
216	O95785	702	KHEERKCPYCP
217	O95785	754	FSFQKKKKKVA

(2) Subset 2 (217 samples)

Sample #	Protein ID	Site	Sequences
1	P05141	10	AVSFAKDFLAG
2	P26358	160	PRITRKSTRQT
3	P26358	179	GPAKRKPQEES
4	P26358	203	KDQDEKRRRVT
5	O75530	89	CVNSLKEDHNQ
6	O75530	184	TMQCIKHYVGH
7	O75530	293	PFISQKIHFPD
8	O75530	322	DLILSKSCENA
9	Q5VTE0	146	YTLGVKQLIVG
10	Q5VTE0	179	VSTYIKKIGYN
11	Q5VTE0	215	WFKGWKVTRKD
12	Q5VTE0	255	LQDVYKIGGIG
13	P0CE47	249	EIVGIKETQKS
14	P0CE47	300	KPGTIKPHTKF
15	P0CE47	304	IKPHTKFESEV
16	P0CE47	314	VYILSKDEGGR
17	O75367	8	SRGGKKKSTKT
18	P62807	13	APAPKKGSKKA
19	P62807	24	VTKAQKKDGKK
20	P62807	29	KKDGKKRKRSR
21	P62807	31	DGKKRKRSRKE
22	P62807	86	LAHYNKRSTIT
23	Q55BN9	64	SELLIKKLPFQ
24	Q55BP0	64	SDLLIKKLPFQ
25	P62803	80	EHAKRKTVTAM
26	Q86L05	90	DIEALKKIGPK
27	Q86L05	95	KKIGPKNKKAI

28	Q7M1B9	18	AEKVLKSKTPV
29	P14909	36	KTKKIKIIDFG
30	P14909	263	REIIQKMGILA
31	P14909	301	MVSLFKKRRDV
32	P14909	315	ELTKVKGVEVS
33	P14909	321	GVEVSKPNGAF
34	P22498	72	HDNAQKMGLKI
35	P22498	102	NFDESKQDVTE
36	Q9Y232	204	PKSPVKSRTAV
37	Q9Y232	348	DIVVRKQDGFT
38	Q9Y232	360	ILLSTKSSENN
39	P80053	20	VKKLYKVGELL
40	P80053	97	MIMTWKNSLLL
41	P80053	109	PYGGGKGGVRV
42	P80053	137	IQAIYKYLGSE
43	P80053	313	PKVKAKLIVEG
44	P61991	21	DISKIKKVWRV
45	P26358	302	RRPEEKEPEKV
46	O75530	335	CWKPGKMEDDI
47	O75530	344	DIDKIKPSESN
48	Q5VTE0	376	KFAELKEKIDR
49	P55907	74	HPASEKKADPV
50	P81543	20	THEGHKVYGPV
51	P49949	20	EHQGHKVYGPV
52	O43524	195	CVPYFKDKGDS
53	Q08369	328	NLNKSKTPAGP
54	Q08369	402	SAVCSKLSPQG
55	P10412	17	PAPAEKTPVKK
56	P10412	127	KAGAAKAKKPA
57	P16401	22	KSPAKKKATKK
58	P16401	26	KKKATKKAAGA
59	P16401	37	GAAKRKATGPP
60	P16401	143	TPKKAKKAAGA
61	P16401	150	AAGAKKAVKKT
62	O75367	235	GNTLEKKGGKE
63	P40283	43	GKKLPKEAGAG
64	P40283	111	LARYNKKPTIT
65	Q41811	17	GKGARKRHRKV
66	O14979	76	GGAAIKGGRRR
67	O14979	142	KINASKNQQDD
68	O14979	149	QQDDGKMFIGG
69	Q14103	67	ESEGAKIDASK
70	Q14103	111	WDTTKKDLKDY
71	Q14103	129	VDCTLKLDPIT
72	Q14103	146	GFVLFKESESV
73	Q14103	176	RAKAMKTKEPV
74	Q14103	178	KAMKTKEPVKK
75	Q14103	255	SKCEIKVAMSK
76	P16862	69	DEKINKNVEAH
77	P16862	323	IEELLKTNRIS
78	P16862	336	QYERMKHLNIC
79	P16862	536	LMESVKLTKAV

80	P16862	539	SVKLTKAVAEA
81	P16862	588	KDKRLKIAIVN
82	O00159	527	HFLTHKLADQR
83	P13538	370	LKFKQKQREEQ
84	Q96RI1	132	SAGRIKGDELC
85	Q96RI1	172	KNAVYKCKNGG
86	Q96RI1	353	EIFNKKLPSGH
87	Q96RI1	434	LQKLCKIHQPE
88	P11940	104	GNIFIKNLDKS
89	P11940	213	KDLFGKFGPAL
90	P11940	254	DEMNGKELNGK
91	P06400	65	LCQKLKIPDHV
92	P06400	130	EISVHKFFNLL
93	P06400	192	SALVLKVSWIT
94	P06400	279	IEVLCKEHECN
95	P06400	319	VENLSKRYEEI
96	P06400	427	IGYIFKEKFAK
97	P06400	429	YIFKEKFAKAV
98	Q86L05	156	VKSTVKFQLKK
99	Q86L05	207	KTLYVKTSMGP
100	P0CX43	39	LQVGLKNYDPQ
101	P0CX43	97	KLNKNKKLIKK
102	P0CX43	98	LNKNKKLIKKL
103	P0CX43	102	KKLIKKLSKKY
104	P47914	22	HRNGIKKPRSQ
105	P47914	23	RNGIKKPRSQR
106	P47914	48	MRFAKKHNKKG
107	P47914	92	KGVSRKLDRLA
108	P47914	124	CRPKAKAKAKA
109	P47914	149	PAQAPKRTQAP
110	Q969Q0	44	RRYDRKQSGYG
111	Q969Q0	100	GDKKRKGQVIQ
112	P0CX27	78	ECVKCKTRAQL
113	P02393	6	MSSITKEQVVE
114	P02393	72	AAGANKIGVIK
115	P0A7K2	30	SAMEEKFGVSA
116	P0A7K2	109	AEALKKALEEA
117	P07472	110	DGDEAKTKLEE
118	Q12962	42	TAAENKASPAG
119	Q12962	213	GINVKKPHYFT
120	Q04207	56	STDTTKTHPTI
121	Q04207	93	HELVGKDCRDG
122	Q53688	185	TLMSWKNPPSY
123	Q53688	417	DLRYYKISPDQ
124	E5KWG9	296	QKRVEKLLRSD
125	B6A879	11	TYTSSKAMSDI
126	B6A879	83	ADEINKIGNPT
127	B6A879	374	YVLLIKELRSA
128	B6A879	552	LFSEAKGHFIS
129	Q9L5X8	119	RFVTQKAFAHG
130	Q9L5X8	287	GLGELKISDTI
131	Q9L5X8	341	LERLEKELVHN

132	D0ZRV8	13	LIELVKKVNRN
133	D0ZRV8	194	WKAQQKMNEQR
134	Q6NEZ3	80	MPESVKAGTGF
135	Q6NEZ3	105	NLSGYKNAKLT
136	Q6NEZ3	111	NAKLTKSSIRI
137	Q6NEZ3	202	TTNADKPFATV
138	Q6NEZ3	369	APEDVKYIDGT
139	Q6NEZ3	407	WKNESKKPNES
140	Q6NEZ3	461	YSNSLKDLVSI
141	D1BQI7	38	YGRQLKTRIPP
142	Q9UKV3	143	KQYLEKQQELL
143	Q9UKV3	223	DEKPRKGERRS
144	Q9UKV3	315	EIPRVKPEEMM
145	P18281	59	AETVDKSGPAI
146	Q9SJU4	15	KASPVKSDWVK
147	Q9SJU4	20	KSDWVKGQSLL
148	Q9SJU4	131	STTDGKKMVDV
149	Q9SJU4	132	TTDGKKMVDVL
150	Q9SJU4	348	QNTCLKTWGGK
151	Q9ZU52	271	PGAEHKNKASP
152	Q9ZU52	348	QGKPEKIEASQ
153	P62157	14	QIAEFKEAFSL
154	P07463	14	QIAEFKEAFAL
155	P07463	22	FALFDKDGDGT
156	P07463	78	MARKMKEQDSE
157	P04573	67	GPKGDKKNIGP
158	P04573	68	PKGDKKNIGPE
159	P04573	112	DFDEFKFIMQK
160	P00889	14	RLFGAKNASCL
161	P00889	34	SSTNLKDILAD
162	P00889	233	GAIDSKLDWSH
163	P00889	317	LTQLQKEVGKD
164	P00889	327	DVSDEKLRDYI
165	P00889	366	REFALKHLPHD
166	P00889	393	LLEQGKAKNPW
167	P00063	21	KIFKTKCAQCH
168	P00078	61	GYAYSKANAES
169	P00078	97	SFAGMKKPQER
170	P00077	33	CHTGAKGGANG
171	P00077	97	SFAGIKKPQER
172	P00043	7	PAPYEKGSEKK
173	P00076	8	AERGKKLFESR
174	P00076	72	LENPKKYVPGT
175	P00076	88	GIKAKKDRQDI
176	P00047	87	FIPGTKMAFGG
177	P02993	244	SRPTEKPLRLP
178	P02994	129	EAGISKDGQTR
179	P02994	154	IVAVNKMDSVK
180	P02994	170	FQEIVKETSNF
181	P02994	184	VGYNPKTVPFV
182	Q96KQ7	179	PEGQPKVHRAR
183	Q96KQ7	253	EVTLTKGDPGS

184	Q96KQ7	288	VDSDSKSEVEA
185	Q96KQ7	344	RRKAKKKWRKD
186	P53991	101	IETEGKIPFWE
187	P53991	282	EQNNRKGGKMY
188	P10587	33	DWSAKKLVWVP
189	P10587	67	QENGKKVTLSK
190	P10587	82	KMNPPKFSKVE
191	P10587	183	ESGAGKTENTK
192	P10587	189	TENTKKVIQYL
193	P11416	109	ACEGCKGFFRR
194	P11416	128	TCHRDKNCIIN
195	P11416	134	NCIINKVTRNR
196	P11416	171	KKEAPKPECSE
197	P11416	227	IDLWDKFSELS
198	P11416	234	SELSTKCIIKT
199	P27064	164	RDKLNKYGRPL
200	P27064	316	FRVLAKALRMS
201	P27064	466	VWKEIKFEFEA
202	P04717	252	CEEMLKRAVFA
203	P04717	356	DDYIKKDRSRG
204	P04992	21	GVKEYKLTYYT
205	P04992	252	CEEMMKRAVFA
206	P04992	463	ACEVWKEIRFN
207	Q5SLP6	39	IMEFVKAFNAA
208	Q5SLP6	109	VLEIAKQKMPD
209	P0CX53	96	DRKKDKNVKHS
210	P0CX53	99	KDKNVKHSGNI
211	P62986	27	TIENVKAKIQD
212	P62986	29	ENVKAKIQDKE
213	P62986	112	AVNCRKKKCGH
214	P55072	136	FEVYLKPYFLE
215	O95785	105	IVRTMKPHAEL
216	O95785	373	YVQHAKLHMRE
217	O95785	479	VDYFGKAEPSL

(3) Subset 3 (217 samples)

Sample #	Protein ID	Site	Sequences
1	P05141	33	PIERVKLLLQV
2	P05141	92	LNFAFKDKYKQ
3	P05141	166	LVKIYKSDGIK
4	Q9Y232	17	GKSRKKNWQYE
5	P00042	6	PAPFKKGSEKK
6	P00042	28	CHTVEKGGPHK
7	P00042	95	GLKKEKDRNDL
8	P26358	29	VRRRLKDLERD
9	P26358	45	ECVKEKLNLLH
10	P26358	67	CDLETKLRKEE
11	P26358	81	EGYLAKVKSLL

12	P26358	130	ANSPPKPLSKP
13	P26358	148	SDGEAKPEPSP
14	O75530	70	RKSWGKGKWKS
15	O75530	72	SWGKGKWKSKK
16	O75530	77	KWKSKKCKYSF
17	O75530	250	DLLGEKIMSCG
18	Q5VTE0	20	HVDSGKSTTTG
19	Q5VTE0	51	AAEMGKGSFKY
20	Q5VTE0	84	KFETSKYYVTI
21	Q5VTE0	154	IVGVNKMDSTE
22	Q5VTE0	172	YEEIVKEVSTY
23	P0CE47	137	IVFLNKCDMVD
24	P0CE47	283	LLRGIKREEIE
25	P0CE47	325	HTPFFKGYRPQ
26	O75367	96	LNQLLKGVTIA
27	O75367	142	KSPSQKKPVSK
28	O75367	143	SPSQKKPVSKK
29	O75367	147	KKPVSKKAGGK
30	O75367	152	KKAGGKKGARK
31	O75367	153	KAGGKKGARKS
32	P62807	16	PKKGSKKAVTK
33	P62807	17	KKGSKKAVTKA
34	O15819	126	VTIMPKDIHLA
35	Q55BN9	123	VTIMVKDIQLA
36	P61830	38	TGGVKKPHRYK
37	P04637	24	FSDLWKLLPEN
38	P04637	139	FCQLAKTCPVQ
39	Q86L05	46	DTKKDKRFSGQ
40	Q86L05	78	CDEATKIGAEF
41	P80322	37	GDISTKELGTI
42	P14909	31	ARNVEKTKKIK
43	P14909	137	YAEVVKLLGGK
44	P39462	313	LAEAGKVKPMI
45	Q9Y232	312	GVTASKRKFID
46	Q9Y232	314	TASKRKFIDDR
47	P0C835	6	MSSGKKAVKVK
48	P0C835	48	DPETGKYFRHK
49	P80053	45	RIIQVKIQIRG
50	P80053	231	GYYAGKFLSEM
51	P80053	265	KAIEIKEKTGS
52	P13123	22	TSKIKKVWRVG
53	P61991	13	YKGEEKQVDIS
54	P61991	53	EKDAPKELLQM
55	P39476	13	YKGEEKEVDIS
56	P26358	240	EERDEKEEKRL
57	P26358	243	DEKEEKRLRSQ
58	P26358	255	KEPTPKQKLKE
59	P26358	259	PKQKLKEEPDR
60	P26358	281	EDGDEKDEKKH
61	P26358	285	EKDEKKHRSQP
62	P26358	296	KDLAAKRRPEE
63	Q5VTE0	273	ETGVLKPGMVV

64	P81539	13	SLPVVKEEQGV
65	P81539	19	EEQGVKIYGTY
66	P49949	73	HPASEKKADPI
67	O43524	270	GRAAKKKAALQ
68	O43524	455	TIQENKPATFS
69	O43524	569	SLGSAKHQQQS
70	Q08369	320	QTRKRKPKNLN
71	Q08369	326	PKNLNKSKTPA
72	P16403	26	KKKAAKKAGGT
73	P16403	136	PVGAAKKPKKA
74	P16403	152	PKKSAKKTPKK
75	P16403	156	AKKTPKKAKKP
76	P10412	32	SAGAAKRKASG
77	P10412	139	AAKKPKKATGA
78	P16401	67	LAALKKALAAG
79	P16401	141	GATPKKAKKAA
80	O75367	160	ARKSKKKQGEV
81	O75367	196	LFLGQKLNLIH
82	O75367	250	VLELRKKNGPL
83	P40283	40	PKAGKKLPKEA
84	P40283	58	KKMKKKSVETY
85	P40283	98	NDIFEKLAQEA
86	O14979	137	FAEGSKINASK
87	O14979	212	ELKEHKLDGKL
88	O14979	224	DPKRAKALKGK
89	Q14103	242	KKIMEKKYHNV
90	P16862	33	IDFYTKFLSLE
91	P16862	106	DILAVKDTLNA
92	P16862	144	GVTSTKNAVST
93	P16862	276	CMEFKKREGRL
94	P16862	551	QAKDFKRAMSL
95	O00159	289	WKVVRKALTVI
96	O00159	444	NYCNEKLQQLF
97	O00159	627	YVRCIKPNDAK
98	P13538	50	KESFVKGTIQS
99	P13538	56	GTIQSKEGGKV
100	P13538	206	AASGEKKKEEQ
101	P13538	273	TYLLEKSRVTF
102	P13538	366	HYGNLKFKQKQ
103	Q96RI1	158	TCEGCKGFFRR
104	Q96RI1	174	AVYKCKNGGNC
105	Q96RI1	224	SKRLRKNVKQH
106	Q96RI1	384	MFSFYKSIGEL
107	Q96RI1	390	SIGELKMTQEE
108	P11940	80	DVIKGKPVRIM
109	P11940	129	NILSCKVVCDE
110	P11940	229	TDESGKSKGFG
111	P11940	231	ESGKSKGFGFV
112	P11940	333	EGGRSKGFGFV
113	P11940	348	PEEATKAVTEM
114	P06400	154	SRLLKKYDVLF
115	P06400	447	GSQRYKLGVRL

116	P0CX43	18	VKELLKYSNET
117	P0CX43	105	IKKLSKKYNAF
118	P47914	15	HNQSRKWHRNG
119	P47914	63	QANNAKAMSAR
120	P47914	73	RAEAIKALVKP
121	P47914	126	PKAKAKAKAKD
122	P47914	130	AKAKAKDQTKA
123	Q969Q0	13	RRTFCKKCGKH
124	Q969Q0	14	RTFCKKCGKHQ
125	Q969Q0	22	KHQPHKVTQYK
126	Q969Q0	27	KVTQYKKGKDS
127	Q969Q0	97	ELGGDKKRKGQ
128	P0CX27	6	MVNVPKTRKTY
129	P0CX27	9	VPKTRKTYCKG
130	P0CX27	13	RKTYCKGKTCR
131	P0CX27	29	KVTQYKAGKAS
132	P02393	31	KELEEKFGVSA
133	P02393	102	APSTLKEAVSK
134	P0A7K2	66	AAGANKVAVIK
135	P0A7K2	96	APAALKEGVSK
136	Q04207	62	THPTIKINGYT
137	Q53688	216	QESHSKILDLD
138	Q53688	250	SIIKNKIIIVE
139	Q53688	302	NFTAEKISISE
140	Q53688	429	HVFNQKRRGKI
141	E5KWG9	16	YSIIEKTAQVV
142	E5KWG9	84	HGLTPKTDHPV
143	E5KWG9	258	FERLIKSAPYD
144	B6A879	148	HYVGDKTKPKL
145	B6A879	152	DKTKPKLSAYI
146	Q9L5X8	54	SNDIEKERGIT
147	Q9L5X8	63	ITILAKNTAIN
148	Q9L5X8	133	IVVINKIDRPG
149	Q9L5X8	254	KTRNGKVGTVM
150	Q9L5X8	427	NIGLRKGELKD
151	Q9L5X8	545	NALKGKQLTNV
152	D0ZRV8	14	IELVKKVNRNK
153	Q6NEZ3	92	ALIKIKDISVS
154	Q6NEZ3	208	PFATVKVDPAD
155	Q6NEZ3	238	ATVPEKLNEKM
156	Q6NEZ3	242	EKLNEKMDGKV
157	Q6NEZ3	311	TELDTKKPEDT
158	Q6NEZ3	403	YDPEWKNESKK
159	Q9UKV3	58	PSTSRKMAELE
160	Q9UKV3	70	VTLDGKPLQAL
161	Q9UKV3	91	QRGLAKSGQKS
162	P18281	71	EDVHVKKVDRG
163	Q9SJU4	10	TATMLKASPVK
164	Q9SJU4	58	ADELVKTAKTI
165	Q9SJU4	150	GIKVDKGLVPL
166	Q9SJU4	378	LAQLGKYTGEG
167	Q9ZU52	238	LEVAEKVWSEV

168	P11118	144	YEEFVKMMMSK
169	P04573	81	LTLCSKWVRQD
170	P04573	153	FMDLIKKSKNA
171	P00044	11	AGSAKKGATLF
172	P00063	35	KGAGHKQGPNL
173	P00043	11	EKGSEKKGANL
174	P00070	17	PTTGEKIFKTK
175	P02993	84	KFETAKYYVTI
176	P02993	171	RFEEIKKEVSA
177	P02994	44	IEKFEKEAAEL
178	P02994	178	SNFIKKVGYNP
179	Q96KQ7	53	EETLPKATPDS
180	Q96KQ7	122	PSSPSKGGSCP
181	Q96KQ7	229	LAKRRKLNSGG
182	Q96KQ7	348	KKKWRKDSPWV
183	Q96KQ7	354	DSPWVKPSRKR
184	Q96KQ7	361	SRKRRKREPPR
185	P53991	73	NKAPFKAKVRS
186	P53991	251	GNSDAKLYDEE
187	P53991	262	LQAIAKAYPGQ
188	P10587	85	PPKFSKVEDMA
189	P10587	142	IIDMYKGKKRH
190	P68696	81	RSIYGKKGSAG
191	P68696	82	SIYGKKGSAGV
192	P68696	94	TVKTSKSILVG
193	P68696	116	ANVVEKLADYL
194	P11416	155	DVGMSKESVRN
195	P11416	207	LCQLGKYTTNN
196	P27064	146	PTAYIKTFQGP
197	P27064	463	ACEVWKEIKFE
198	P04717	146	PYAYVKTFQGP
199	P0A7J7	81	AAVLLKKAAGI
200	P0A7J7	97	KPNKDKVGKIS
201	P0A7J7	113	EIAQTKAADMT
202	Q5SLP6	86	AAGLEKGAHKP
203	P0CX53	48	GEDIAKATKEF
204	P0CX53	57	EFKGIKVTVQL
205	P62986	11	KTLTGKTITLE
206	P62986	48	LIFAGKQLEDG
207	P62986	93	KYNCDKMICRK
208	P55072	8	SGADSKGDDLS
209	P55072	18	STAILKQKNRP
210	P55072	45	SLSQPKMDELQ
211	P55072	60	DTVLLKGKKRR
212	P55072	62	VLLKGKKRREA
213	P55072	312	DAIAPKREKTH
214	P05547	11	AAEEAKKKQDD
215	P05547	34	EEQSLKKQKKG
216	P05547	63	AEELKKEQERK
217	O95785	339	IEEIQKLKQVP

(4) Subset 4 (217 samples)

Sample #	Protein ID	Site	Sequences
1	P05141	43	VQHASKQITAD
2	P05141	163	GDCLVKIYKSD
3	P05141	171	KSDGIKGLYQG
4	P05141	206	MLPDPKNTHIV
5	Q9Y232	116	RRHTEKQKEST
6	P00042	33	KGGPHKVGPNL
7	P26358	188	ESERAKSDESI
8	P26358	233	GTRTEKEEERD
9	Q5VTE0	100	HRDFIKNMITG
10	P0CE47	38	TTVLAKTYGGA
11	P0CE47	253	IKETQKSTCTG
12	O75367	7	SSRGGKKKSTK
13	O75367	121	KKRGSKGKLEA
14	O75367	137	PAKKAKSPSQK
15	P69150	54	LREIRKYQKST
16	P69150	123	VTIMTKDMQLA
17	P59226	57	IRKYQKSTELL
18	P62803	60	TRGVLKVFLEN
19	P62803	78	YTEHAKRKTVT
20	P62803	92	VVYALKRQGRT
21	P04637	319	SSPQPKKKPLD
22	P04637	320	SPQPKKKPLDG
23	P04637	357	DAQAGKEPGGS
24	Q86L05	61	TVTKPKLSVCV
25	Q86L05	133	LNKVGKFPTLL
26	Q7M1B9	57	RLTIAKVNTDD
27	P80322	6	SDDYVKARVMF
28	P80322	12	ARVMFKEEQIS
29	P14909	51	DLPTFKRIRDA
30	P14909	58	IRDAAKEALDQ
31	P14909	169	QSKISKRTKMI
32	P14909	242	VNGFSKTFSMT
33	P14909	354	KLIEEKGVVTI
34	P39462	22	EIGVPKPKGPQ
35	P39462	73	HEIAGKIEEVG
36	P39462	239	RITESKGVDAV
37	P39462	259	LSVYPKALAKQ
38	P39462	315	EAGKVKPMITK
39	P39462	338	NLENFKAIGRQ
40	P22498	35	NTDWYKWVHDP
41	P22498	251	AYDGIKSVSKK
42	P0C835	9	GKKAVKVKTPA
43	P80053	17	TQQVKKLYKVG
44	P80053	207	KEAANKFIGGV
45	P80053	304	ENVINKFNAPK
46	P80053	415	VVRAMKARGIL
47	P13123	13	YKGEEKEVDTS
48	P13123	21	DTSKIKKVWRV

49	P13123	39	YDDNGKTGRGA
50	P13123	48	GAVSEKDAPKE
51	P26358	250	LRSQTKEPTPK
52	O75530	375	MDFWQKMLALG
53	Q5VTE0	392	LEDGPKFLKSG
54	P55907	75	PASEKKADPVN
55	O43524	259	MDNSNKYTKSR
56	Q08369	318	GIQTRKRKPKN
57	P16403	21	EKAPVKKKAAK
58	P16403	63	SLAALKKALAA
59	P16403	97	TLVQTKGTGAS
60	P16403	122	KPKVKKAGGTK
61	P16403	127	KAGGTKPKKPV
62	P16403	129	GGTKPKKPVGA
63	P10412	23	TPVKKKARKSA
64	P10412	129	GAAKAKKPAGA
65	P16401	133	AAKAKKPAGAT
66	O75367	226	ADIDLKDDLGN
67	O75367	239	EKKGGKEFVEA
68	P40283	36	AEKKPKAGKKL
69	P40283	57	KKKMKKKSVET
70	P40283	69	KIYIFKVLKQV
71	P40283	142	VSEGTKAVTKF
72	Q41811	80	EHARRKTVTAM
73	O14979	209	KVLELKEHKLD
74	O14979	250	SEEQIKEYFGA
75	O14979	269	LPMDTKTNERR
76	O14979	288	DEEPVKKLLES
77	O14979	302	QIGSGKCEIKV
78	Q14103	98	QREEWKMFIGG
79	Q14103	153	SESVDKVMDQK
80	Q14103	158	KVMDQKEHKLN
81	Q14103	218	LPMDNKTNKRR
82	Q14103	341	QSGYGKVSRRG
83	P16862	58	DSISLKILLRP
84	P16862	225	RSAIFKGCRAF
85	P16862	248	GPEYIKEFHWE
86	P16862	432	CDIVSKHRSRG
87	P16862	585	KLPKDKRLKIA
88	O00159	281	SSINDKSDWKV
89	O00159	361	RKIIAKGEELL
90	O00159	455	IELTLKSEQEE
91	O00159	561	TGFLDKNNDLL
92	O00159	578	TMCSSKNPIMS
93	P13538	31	IEAQNKPFDAK
94	P13538	88	PPKYDKIEDMA
95	P13538	208	SGEKKKEEQSG
96	P13538	297	IMSNKKPELID
97	P13538	386	TEVADKAAYLM
98	P13538	400	SAELLKALCYP
99	P11940	108	IKNLDKSIDNK
100	P11940	167	LLNDRKVFVGR

101	P11940	221	PALSVKVMTDE
102	P11940	246	HEDAQKAVDEM
103	P11940	312	DERLRKEFSPF
104	P11940	371	ALAQRKEERQA
105	P11940	512	TVPQYKYAAGV
106	P06400	94	GGYIQKKKELW
107	P06400	236	PPMLLKEPYKT
108	P06400	417	KESILKRVKDI
109	Q86L05	195	LVSLLKKGWQN
110	P0CX43	54	FSGSLKLPNCP
111	P0CX43	92	VDDLKKLNKNK
112	P0CX43	133	LSKAGKFPTPV
113	P0CX43	160	IKFQLKKVLCL
114	P0CX43	196	VSLLKKNWQNV
115	P47914	87	KPKIPKGVSRK
116	P47914	106	HPKLGKRARAR
117	P47914	114	RARIAKGLRLC
118	Q969Q0	30	QYKKGKDSLYA
119	Q969Q0	64	KAKTTKKIVLR
120	Q969Q0	65	AKTTKKIVLRL
121	Q969Q0	80	PNCRSKRMLAI
122	P0CX27	32	QYKAGKASLFA
123	P0CX27	63	FHKKAKTTKKV
124	P0CX27	66	KAKTTKKVVLR
125	P0CX27	98	LGGEKKQKGQA
126	P02393	26	LSEFIKELEEK
127	P07472	103	KEGMSKEDGDE
128	Q12962	175	ALQHCKMKGTA
129	Q04207	37	MRFRYKCEGRS
130	Q04207	123	IQCVKKRDLEQ
131	Q04207	315	KSIMKKSPFNG
132	Q53688	140	DTVISKGLLKI
133	Q53688	144	SKGLLKIVKDG
134	Q53688	265	FQEELKLNSDG
135	Q53688	379	PEAYTKLQQYM
136	E5KWG9	68	FTMLPKGLDPY
137	E5KWG9	94	VGSLLKEVHEN
138	E5KWG9	126	FPSAEKLGKVS
139	E5KWG9	168	GIDYSKRTMNL
140	B6A879	210	GDIGNKSKKVQ
141	B6A879	298	HAMGHKLDLAF
142	B6A879	402	SGVKAKLKKSN
143	B6A879	565	TPRTVKQKGEY
144	Q9L5X8	22	HVDHGKTTLVD
145	Q9L5X8	165	DQLDFKVVYAS
146	Q9L5X8	236	TRGSVKANQQV
147	Q9L5X8	359	TDDPDKFRVSG
148	D0ZRV8	76	IETLQKSIDNE
149	Q6NEZ3	90	FDALIKIKDIS
150	Q6NEZ3	280	DTSGSKTITAR
151	Q6NEZ3	312	ELDTKKPEDTD
152	Q6NEZ3	408	KNESKKPNESY

153	Q9UKV3	41	EGRWRKLPKMP
154	Q9UKV3	81	RVTDLKAALEQ
155	Q9UKV3	113	LENLQKHSTPH
156	Q9UKV3	138	QNSFIKQYLEK
157	Q9UKV3	220	DSDDEKPRKGE
158	Q9UKV3	349	QEEARKSHLAR
159	P18281	23	AETVDKSAPQI
160	Q9SJU4	358	KEENVKAAQDI
161	Q9SJU4	369	LLARAKANSLA
162	Q9SJU4	389	ESEEAKEGMFV
163	Q9ZU52	53	LVKTAKSIASP
164	Q9ZU52	273	AEHKNKASPET
165	Q9ZU52	345	RTWQGKPEKIE
166	Q9ZU52	354	IEASQKALLVR
167	Q9ZU52	361	LLVRAKANSLA
168	P62157	78	MARKMKDTDSE
169	P04573	50	GQTYTKRETEA
170	P04573	156	LIKKSKNALKE
171	P00889	107	QKMLPKAKGGE
172	P00889	321	QKEVGKDVSDE
173	P00044	60	TDANIKKNVLW
174	P00044	61	DANIKKNVLWD
175	P00078	22	GEKLFKGRAAQ
176	P00077	61	GFAYSKANADS
177	P00043	96	GLKKAKDRNDL
178	P00070	64	AGNKNKAVIWE
179	P00047	61	YTDANKQAGIT
180	Q71V39	215	WFKGWKVERKE
181	P02993	222	ERKEGKADGKT
182	P02994	84	KFETPKYQVTV
183	Q96KQ7	28	ALLLEKETRGA
184	Q96KQ7	200	PPVPEKRPPEI
185	Q96KQ7	219	VHSLGKVTSDL
186	Q96KQ7	226	TSDLAKRRKLN
187	P53991	169	KEDPAKKGLCS
188	P53991	194	TGPTGKVLLLP
189	P10587	50	EAASIKEEKGD
190	P10587	66	LQENGKKVTLS
191	P10587	77	KDDIQKMNPPK
192	P10587	136	PIYSEKIIDMY
193	P11416	193	IEKVRKAHQET
194	P11416	238	TKCIIKTVEFA
195	P27064	21	GVKDYKLTYYT
196	P27064	128	NVFGFKALRAL
197	P04717	81	SLDRYKGRCYE
198	P04717	316	FRVLAKALRLS
199	P04992	305	VIDRQKNHGIH
200	P0A7J7	45	KAFNAKTDSIE
201	P0A7J7	82	AVLLKKAAGIK
202	Q5SLP6	80	SYLIRKAAGLE
203	Q5SLP6	111	EIAKQKMPDLN
204	P0CX53	54	ATKEFKGIKVT

205	P0CX53	63	VTVQLKIQNRQ
206	P62986	63	DYNIQKESTLH
207	P62986	113	VNCRKKKCGHT
208	P55072	63	LLKGKKRREAV
209	P55072	81	TCSDEKIRMNR
210	P55072	164	RAVEFKVVETD
211	P55072	277	PEIMSKLAGES
212	P55072	288	ESNLRKAFEEA
213	P05547	20	DDIDRKKAEVR
214	P05547	55	RLLLRKKAAEE
215	P05547	99	LRAIIKEYFDH
216	O95785	341	EIQKLKQVPGD
217	O95785	743	SAEEVKAIERR

(5) Subset 5 (216 samples)

Sample #	Protein ID	Site	Sequences
1	Q9Y232	27	EGPTQKLFLKR
2	Q9Y232	84	YLVRWKGYDSE
3	Q9Y232	118	HTEKQKESTLT
4	Q9Y232	185	SLSSRKNMDLA
5	P00042	10	KKGSEKKGATL
6	P26358	88	KSLLNKDLSLE
7	P26358	194	SDESIKEEDKD
8	O75530	20	MPAAKKQKLSS
9	O75530	79	KSKKCKYSFKC
10	O75530	83	CKYSFKCVNSL
11	O75530	275	MMNAIKESYDY
12	Q5VTE0	30	GHLIYKCGGID
13	Q5VTE0	62	AWVLDKLKAER
14	P0CE47	188	AEWEAKILELA
15	P0CE47	264	VEMFRKLLDEG
16	O75367	73	ARDNKKGRVTP
17	P62807	21	KKAVTKAQKKD
18	P62807	28	QKKDGKKRKRS
19	P61830	116	AAIHAKRVTIQ
20	P61830	122	RVTIQKKDIKL
21	P62803	9	RGKGGKGLGKG
22	P62803	45	RRGGVKRISGL
23	P04637	120	HSGTAKSVTCT
24	P04637	351	EALELKDAQAG
25	Q86L05	38	LQINLKNYDTK
26	Q86L05	44	NYDTKKDKRFS
27	Q86L05	59	IGTVTKPKLSV
28	Q86L05	91	IEALKKIGPKN
29	Q86L05	101	NKKAIKKLSKK
30	Q86L05	106	KKLSKKYDAFL
31	Q7M1B9	20	KVLKSKTPVVV
32	Q7M1B9	44	APILDKLAGEY
33	Q7M1B9	82	TLVIFKDGREV

34	P80322	104	FRVLDKNGDGF
35	P80322	112	DGFIDKDEFRA
36	P14909	23	TTLLYKEIARN
37	P14909	172	ISKRTKMIVFN
38	P14909	302	VSLFKKRRDVM
39	P22498	124	DEYANKDALNH
40	P22498	328	TRTVVKRTEKG
41	P22498	451	VDYNTKRLYWR
42	P0C835	11	KAVKVKTPAGK
43	P0C835	24	ELVPEKVWALA
44	P80053	117	VRVDPKKLTRE
45	P80053	166	LDEYIKITGKV
46	P13123	19	EVDTSKIKKVW
47	P61991	9	VKFKYKGEEKQ
48	P61991	22	ISKIKKVWRVG
49	P13125	9	VRFKYKGEEKE
50	P13125	52	EKDAPKELMDM
51	P39476	19	EVDISKIKKVW
52	P26358	257	PTPKQKLKEEP
53	O75530	342	EDDIDKIKPSE
54	O75530	408	TLTHHKCGAAI
55	Q5VTE0	290	VTTEVKSVEMH
56	Q5VTE0	313	VGFKVKNVSVK
57	Q5VTE0	330	VAGDSKNDPPM
58	Q5VTE0	386	RRSGKKLEDGP
59	P49949	74	PASEKKADPIN
60	O43524	663	NFTGAKQASSQ
61	Q08369	245	CGLYHKMNGIN
62	Q08369	366	EMRPIKTEPGL
63	P16403	64	LAALKKALAAA
64	P16403	106	ASGSFKLNKKA
65	P16403	119	GEAKPKVKKAG
66	P16403	140	AKKPKKAAGGA
67	P10412	21	EKTPVKKKARK
68	P10412	119	GEAKPKAKKAG
69	P10412	122	KPKAKKAGAAK
70	P16401	17	PAPVEKSPAKK
71	P16401	149	KAAGAKKAVKK
72	P16401	153	AKKAVKKTPKK
73	O75367	161	RKSKKKQGEVS
74	O75367	167	QGEVSKAASAD
75	P40283	8	PKAEKKPAEKK
76	P40283	18	KPASEKPVEEK
77	P40283	39	KPKAGKKLPKE
78	P40283	54	GDKKKKMKKKS
79	P40283	64	SVETYKIYIFK
80	P40283	72	IFKVLKQVHPD
81	P40283	105	AQEASKLARYN
82	O14979	221	KLIDPKRAKAL
83	O14979	234	KEPPKKVFVGG
84	Q14103	72	KIDASKNEEDE
85	Q14103	110	SWDTTKKDLKD

86	Q14103	170	KVIDPKRAKAM
87	Q14103	182	TKEPVKKIFVG
88	Q14103	197	DTPEEKIREYF
89	Q14103	221	DNKTNKRRGFC
90	Q14103	237	EEEPVKKIMEK
91	Q14103	243	KIMEKKYHNVG
92	Q14103	251	NVGLSKCEIKV
93	P16862	275	RCMEFKKREGR
94	P16862	369	LDRICKAIDYV
95	P16862	529	NKIVRKPLMES
96	P16862	583	EPKLPKDKRLK
97	O00159	151	KTEATKRLLQF
98	O00159	224	SYLLEKSRVVH
99	O00159	336	TENQLKYLTRL
100	O00159	404	RSLASKDVESP
101	O00159	477	QYFNNKIICDL
102	O00159	571	LFRNLKETMCS
103	P13538	23	LRKSEKERIEA
104	P13538	108	VLYNLKERYAA
105	P13538	214	EEQSGKMQGTL
106	Q96RI1	188	MYMRRKCQECR
107	Q96RI1	227	LRKNVKQHADQ
108	Q96RI1	317	LVEFTKKLPGF
109	Q96RI1	318	VEFTKKLPGFQ
110	P11940	196	TNVYIKNFGED
111	P11940	259	KELNGKQIYVG
112	P11940	324	TITSAKVMMEG
113	P11940	361	RIVATKPLYVA
114	P06400	63	TALCQKLKIPD
115	P06400	95	GYIQKKKELWG
116	P06400	153	MSRLLKKYDVL
117	P06400	265	SARIAKQLEND
118	P06400	329	IYLKNKDLDAR
119	P06400	412	CTVNPKESILK
120	P06400	462	MESMLKSEEER
121	P0CX43	78	DVDRAKSCGVD
122	P0CX43	95	LKKLNKNKKLI
123	P0CX43	118	SEVLIKQVPRL
124	P0CX43	156	VRSTIKFQLKK
125	P47914	52	KKHNKKGLKKM
126	P47914	56	KKGLKKMQANN
127	P47914	77	IKALVKPKEVK
128	P47914	134	AKDQTKAQAAA
129	Q969Q0	98	LGGDKKRKGQV
130	P0CX27	24	KHTQHKVTQYK
131	P0CX27	46	RRYDRKQSGFG
132	P0CX27	60	KPVFHKKAKTT
133	P02393	66	FDVILKAAGAN
134	P02393	115	AEEAKKQLVEA
135	P0A7K2	108	DAEALKKALEE
136	P07472	67	TSAGEKKVNVI
137	P07472	73	KVNVIKVVREI

138	Q12962	108	ANGDVKPVVSS
139	Q12962	177	QHCKMKGTASG
140	Q04207	79	ISLVTKDPPHR
141	Q53688	66	ELGGEKEYRRL
142	Q53688	176	YDTLQKQNYTL
143	Q53688	362	VKECDKTNEIE
144	Q53688	371	IEEATKRNPEA
145	Q53688	445	STHDTKFSEDV
146	E5KWG9	129	AEKLGKVSELV
147	B6A879	43	VIFDGKVYTNT
148	B6A879	531	SLSMGKNGFVL
149	Q9L5X8	431	RKGELKDMAPD
150	D0ZRV8	19	KVNRNKVPTPM
151	Q6NEZ3	123	VGKNVKLDGNQ
152	Q6NEZ3	352	SLLPNKAPIRV
153	Q6NEZ3	455	QMGVYKYSNSL
154	D1BQI7	90	MASIVKALSRK
155	Q9UKV3	95	AKSGQKSALVK
156	Q9UKV3	268	ADRNLKTEEEE
157	Q9UKV3	302	EAPILKEFKEE
158	Q9UKV3	325	MDERPKTRSQE
159	Q9SJU4	293	ASYTLKLLRNR
160	Q9ZU52	8	SASFVKPNTLS
161	Q9ZU52	370	LAQLGKYSAEG
162	Q9ZU52	382	NEDAKKGMFVK
163	P62157	76	TMMARKMKDTD
164	P04573	64	EARGPKGDKKN
165	P04573	154	MDLIKKSKNAL
166	P00889	43	ADLIPKEQARI
167	P00889	103	IPECQKMLPKA
168	P00889	352	HAVLRKTDPRY
169	P00889	450	PLERPKSMSTD
170	P00063	61	YSAANKNMAVN
171	P00078	84	LENPKKFMPGT
172	P00078	90	FMPGTKMSFAG
173	P00043	18	GANLFKTRCLQ
174	P00047	19	GANLFKTRCAQ
175	P00047	81	LENPKKFIPGT
176	Q71V39	84	KFETTKYYITI
177	Q71V39	172	YDEIVKEVSAY
178	P02994	51	AAELGKGSFKY
179	P02994	159	KMDSVKWDESR
180	Q96KQ7	131	CPSRAKMSMTG
181	P53991	122	TKINSKGKEVP
182	P53991	291	MYIQDKVEEYA
183	P53991	315	YFCGLKGMMPG
184	P07041	123	VTIQSKDIQLA
185	P10587	53	SIKEEKGDEVT
186	P10587	144	DMYKGKKRHEM
187	P10587	188	KTENTKKVIQY
188	P10587	204	SSHKGKKDTSI
189	P10587	205	SHKGKKDTSIT

190	P11416	262	QITLLKAACLD
191	P11416	365	KVYVRKRRPSR
192	P27064	32	PEYETKDTDIL
193	P27064	201	GLDFTKDDENV
194	P27064	252	CEEMMKRAIFA
195	P04717	32	PDYQTKDTDIL
196	P04717	450	IREACKWSPEL
197	P04992	81	SLDRYKGHRYR
198	P0A7J7	10	VQAYVKLQVAA
199	P0A7J7	51	TDSIEKGLPIP
200	P0A7J7	100	KDKVGKISRAQ
201	P0CX53	40	LGLSPKKVGED
202	P0CX53	86	VITALKEPPRD
203	P55072	217	QLAQIKEMVEL
204	P05547	35	EQSLKKQKKGF
205	P05547	37	SLKKQKKGFMT
206	P05547	38	LKKQKKGFMTP
207	P05547	47	TPERKKKLRLL
208	P05547	48	PERKKKLRLLL
209	P05547	74	AGERRKIIDQR
210	P05547	112	QIESDKYDVEL
211	P05547	122	LEIIRKDYEIN
212	P05547	139	NDLRGKFIKPT
213	O95785	279	IYFKQKEHLLE
214	O95785	332	QASREKIIEEI
215	O95785	357	RLQCPKCVFGT
216	O95785	756	FQKKKKKVANF

(6) Subset 6 (217 samples)

Sample #	Protein ID	Site	Sequences
1	P05141	23	AAAISKTAVAP
2	Q9Y232	31	QKLFLKRNNVS
3	Q9Y232	69	ERIVDKRKNKK
4	Q9Y232	71	IVDKRKNKKGK
5	Q9Y232	147	NSNFSKTSPKA
6	Q9Y232	151	SKTSPKALVIG
7	Q9Y232	175	SQKFRKNTAPS
8	Q9Y232	191	NMDLAKSGIKI
9	Q9Y232	200	KILVPKSPVKS
10	P26358	39	DSLTEKECVKE
11	O75530	76	GKWKSKKCKYS
12	O75530	106	FNWHSKEGDPL
13	Q5VTE0	212	NMPWFKGWKVT
14	Q5VTE0	244	TRPTDKPLRLP
15	P0CE47	90	HADYVKNMITG
16	P0CE47	177	RGSALKALEGD
17	P0CE47	358	PGDNIKMVVTL
18	O75367	12	KKKSTKTSRSA
19	O75367	116	PELLAKKRGSK

20	O75367	134	TPPPAKKAKSP
21	O75367	148	KPVSKKAGGKK
22	P62807	25	TKAQKKDGKKR
23	P62807	44	SVYVYKVLKQV
24	P62807	117	VSEGTKAVTKY
25	P62807	121	TKAVTKYTSSK
26	P62803	6	MSGRGKGGKGL
27	P62803	17	GKGGAKRHRKV
28	P04637	291	EENLRKKGEPH
29	P04637	292	ENLRKKGEPHH
30	Q86L05	98	GPKNKKAIKKL
31	Q86L05	130	GPGLNKVGKFP
32	Q7M1B9	15	SDFAEKVLKSK
33	P14909	33	NVEKTKKIKII
34	P14909	95	GTDVKKEEVIV
35	P14909	193	SPNDVKKIVDI
36	P14909	220	FVYEGKMRSTL
37	P14909	337	VSKILKTSGFD
38	P14909	344	SGFDVKSLAIK
39	P39462	24	GVPKPKGPQVL
40	P39462	86	VVGYSKGDLVA
41	P39462	252	LNNSEKTLSVY
42	P39462	266	LAKQGKYVMVG
43	P22498	304	TRGNEKIVRDD
44	P22498	374	YDVLTKYWNRY
45	Q9Y232	219	SESPEKLDPVE
46	Q9Y232	284	LAVNGKGTSPF
47	P0C835	34	APKGRKGVKIG
48	P80053	56	SDGKLKTFMGW
49	P80053	118	RVDPKKLTREE
50	P80053	130	EQLSRKYIQAI
51	P80053	279	YPEGRKVTNEE
52	P80053	309	KFNAPKVKAKL
53	P80053	311	NAPKVKAKLIV
54	P80053	360	EWANNKMGEII
55	P80053	371	SDEEAKKLIVD
56	P13123	28	VWRVGKMVSFT
57	P61991	19	QVDISKIKKVW
58	P61991	28	VWRVGKMISFT
59	Q5VTE0	311	DNVGFKVKNVS
60	P55907	21	EHSGHKVYGPV
61	O43524	606	VMGHEKFPSDL
62	Q08369	255	NRPLIKPQRRL
63	Q08369	322	RKRKPKNLNKS
64	Q08369	421	SQASSKQDSWN
65	P16403	52	AVAASKERSGV
66	P16403	81	NNSRIKLGLKS
67	P16403	90	KSLVSKGTLVQ
68	P16403	149	GATPKKSAKKT
69	P16403	153	KKSAKKTPKKA
70	P10412	137	AGAAKKPKKAT
71	P10412	140	AKKPKKATGAA

72	P10412	149	AATPKKSAKKT
73	P16401	140	AGATPKKAKKA
74	P16401	144	PKKAKKAAGAK
75	P16401	157	VKKTPKKAKKP
76	O75367	159	GARKSKKKQGE
77	P40283	7	APKAEKKPAEK
78	P40283	23	KPVEEKSKAEK
79	P40283	25	VEEKSKAEKAP
80	P40283	28	KSKAEKAPAEK
81	O14979	216	HKLDGKLIDPK
82	O14979	233	GKEPPKKVFVG
83	O14979	289	EEPVKKLLESR
84	Q14103	173	DPKRAKAMKTK
85	P16862	83	LNSITKTQDWR
86	P16862	415	IFIPEKPATSS
87	P16862	580	DHNEPKLPKDK
88	P16862	641	RSLNWKDMLGW
89	O00159	200	SSRFGKYMDVQ
90	O00159	269	YLYLVKGQCAK
91	O00159	357	ALTHRKIIAKG
92	O00159	396	TWLVGKINRSL
93	O00159	512	LTFLEKLEDTV
94	O00159	632	KPNDAKQPGRF
95	P13538	20	APYLRKSEKER
96	P13538	60	SKEGGKVTVKT
97	P13538	64	GKVTVKTEGGE
98	P13538	74	ETLTVKEDQVF
99	P13538	191	KTVNTKRVIQY
100	P13538	237	AFGNAKTVRND
101	P13538	249	SSRFGKFIRIH
102	P13538	368	GNLKFKQKQRE
103	Q96RI1	286	QEITNKILKEE
104	Q96RI1	352	AEIFNKKLPSG
105	Q96RI1	431	LDVLQKLCKIH
106	P11940	78	NFDVIKGKPVR
107	P11940	95	DPSLRKSGVGN
108	P11940	138	DENGSKGYGFV
109	P11940	268	VGRAQKKVERQ
110	P11940	269	GRAQKKVERQT
111	P06400	96	YIQKKKELWGI
112	P06400	136	FFNLLKEIDTS
113	P06400	202	TFLLAKGEVLQ
114	P06400	289	NIDEVKNVYFK
115	P06400	294	KNVYFKNFIPF
116	P06400	359	QRTPRKSNLDE
117	Q86L05	161	KFQLKKVLCLA
118	Q86L05	202	GWQNIKTLYVK
119	P0CX43	14	VREHVKELLKY
120	P0CX43	207	GSLVVKSSMGP
121	P47914	38	KGVDPKFLRNM
122	P47914	82	KPKEVKPKIPK
123	P47914	103	YIAHPKLGKRA

124	P47914	122	RLCRPKAKAKA
125	Q969Q0	28	VTQYKKGKDSL
126	P0CX27	61	PVFHKKAKTTK
127	P0CX27	67	AKTTKKVVLRL
128	P0CX27	97	ELGGEKKQKGQ
129	P02393	58	PAEEEKTEFDV
130	P0A7K2	52	EAAEEKTEFDV
131	P0A7K2	85	GLKEAKDLVES
132	P07472	112	DEAKTKLEEAG
133	Q04207	28	IIEQPKQRGMR
134	Q04207	303	IEEKRKRTYET
135	Q53688	147	LLKIVKDGDEY
136	Q53688	158	FLEYFKWKLPL
137	Q53688	160	EYFKWKLPLTE
138	Q53688	248	LRSIIKNKIII
139	Q53688	358	ANQIVKECDKT
140	Q53688	390	PAVYAKAYEDT
141	E5KWG9	191	GVFEEKGVRAI
142	E5KWG9	211	SEELLKFCERS
143	E5KWG9	229	SWDSPKINRFT
144	B6A879	121	PDVNFKEDGSQ
145	B6A879	291	RELQKKAHAMG
146	B6A879	445	NLYSPKDPGEQ
147	Q9L5X8	28	TTLVDKLLQQS
148	Q9L5X8	480	HYGPHKGGNIG
149	Q9L5X8	499	ANAAGKALTNA
150	Q6NC90	132	RLKPLKPGKGF
151	Q6NC90	135	PLKPGKGFGEI
152	D0ZRV8	36	RLRVRKYRDPQ
153	D0ZRV8	53	LPESLKALLAY
154	D0ZRV8	190	HLPQWKAQQKM
155	Q6NEZ3	145	INDHLKASLEG
156	Q9UKV3	213	SISEEKGDSDD
157	Q9UKV3	305	ILKEFKEEGEE
158	Q9SJU4	61	LVKTAKTIASP
159	Q9SJU4	186	GARFAKWRTVV
160	Q9SJU4	203	SALAVKEAAWG
161	Q9ZU52	288	TLTMLKRRVPP
162	P62157	31	GTITTKELGTV
163	P04573	14	LGPEEKDECMK
164	P00889	49	EQARIKTFRQQ
165	P00889	109	MLPKAKGGEEP
166	P00889	139	VSWLSKEWAKR
167	P00889	382	VAQLYKIVPNV
168	P00078	98	FAGMKKPQERA
169	P00043	61	TDANKKKGVEW
170	P00043	62	DANKKKGVEWS
171	P00070	31	CHTVEKGAGHK
172	P00070	96	FPGLKKPQERA
173	P00047	35	QGGANKIGPNL
174	P00047	47	GLFGRKTGSVE
175	Q71V39	212	NMPWFKGWKVE

176	P02993	212	RLPWYKGWNIE
177	P02994	210	NAPWYKGWEKE
178	P02994	222	KAGVVKGKTLL
179	Q96KQ7	341	SSGRRKAKKKW
180	Q96KQ7	358	VKPSRKRRKRE
181	P53991	38	PVAATKASTAV
182	P53991	170	EDPAKKGLCSN
183	P07041	116	CAIHAKRVTIQ
184	P10587	11	LSDDEKFLFVD
185	P10587	72	KVTLSKDDIQK
186	P68696	91	GVITVKTSKSI
187	P11416	86	LPRIYKPCFVC
188	P11416	147	YCRLQKCFDVG
189	P11416	164	RNDRNKKKKEA
190	P27063	6	PQTETKASVGF
191	P27064	81	SLDRYKGRCYG
192	P27064	177	CTIKPKLGLSA
193	P27064	183	LGLSAKNYGRA
194	P27064	334	GTVVGKLEGER
195	P27064	356	DDFVEKDRSRG
196	P27064	450	IREASKWSPEL
197	P04717	8	PQTETKAKVGF
198	P04992	146	PPAYVKTFQGP
199	P0A7J7	72	FTFVTKTPPAA
200	Q5SLP6	90	EKGAHKPGREK
201	P0CX53	31	AALAPKIGPLG
202	P0CX53	93	PPRDRKKDKNV
203	P0CX53	94	PRDRKKDKNVK
204	P0CX53	119	RQMRDKSFGRT
205	P0CX53	146	CRVDFKNPHDI
206	P62986	6	MQIFVKTLTGK
207	P55072	112	DVKYGKRIHVL
208	P55072	148	YRPIRKGDIFL
209	P55072	295	FEEAEKNAPAI
210	P05547	12	AEEAKKKQDDI
211	P05547	68	KEQERKAGERR
212	P05547	84	RCGQPKNLDGA
213	O95785	179	VEDTPKTLDMA
214	O95785	385	PGQTTKEPFGG
215	O95785	610	AALELKQAFRE
216	O95785	641	IVLVAKLGPQV
217	O95785	697	PAMALKHEERK

(7) Subset 7 (217 samples)

Sample #	Protein ID	Site	Sequences
1	P05141	63	VVRIPKEQGVL
2	P05141	147	AADVGKAGAER
3	P05141	268	RDEGGKAFFKG
4	Q9Y232	76	KNKKGKTEYLV

5	Q9Y232	195	AKSGIKILVPK
6	P00042	85	YIPGTKMAFGG
7	P00042	92	AFGGLKKEKDR
8	P26358	43	EKECVKEKLNL
9	P26358	134	PKPLSKPRTPR
10	O75530	22	AAKKQKLSSDE
11	O75530	74	GKGKWKSKKCK
12	O75530	211	LLSVSKDHALR
13	O75530	332	AIVCWKPGKME
14	Q5VTE0	44	IEKFEKEAAEM
15	Q5VTE0	64	VLDKLKAERER
16	P0CE47	25	HVDHGKTTLTA
17	P0CE47	295	GQVLAKPGTIK
18	O75367	9	RGGKKKSTKTS
19	O75367	34	LRYIKKGHPKY
20	O75367	72	AARDNKKGRVT
21	O75367	135	PPPAKKAKSPS
22	P62807	6	MPEPAKSAPAP
23	P68432	116	CAIHAKRVTIM
24	P09988	116	CAIHGKRVTIQ
25	Q55BN9	65	ELLIKKLPFQR
26	P61830	57	IRRFQKSTELL
27	P61830	123	VTIQKKDIKLA
28	P62803	13	GKGLGKGGAKR
29	P04637	101	SVPSQKTYQGS
30	P04637	132	SPALNKMFCQL
31	P04637	321	PQPKKKPLDGE
32	Q86L05	18	VSQLFKEAQES
33	Q86L05	24	EAQESKRGFLE
34	Q86L05	102	KKAIKKLSKKY
35	P80322	44	LGTIMKRLGMS
36	P80322	153	DYEEWKEIIQE
37	P14909	166	DDLQSKISKRT
38	P14909	281	TSFVQKAAVKA
39	P14909	334	FPNVSKILKTS
40	P14909	349	KSLAIKLIEEK
41	P39462	31	PQVLIKVEAAG
42	P39462	61	EDLGVKLPVTL
43	P39462	172	SLDPTKTLLVV
44	P39462	192	AVQIAKAVSGA
45	P39462	320	KPMITKTMKLE
46	P39462	323	ITKTMKLEEAN
47	P22498	219	GYVGVKSGFPP
48	P22498	255	IKSVSKKPVGI
49	Q9Y232	388	AADDSKLVLLS
50	Q9Y232	417	LTDDRKRESTK
51	Q9Y232	422	KRESTKMAEAI
52	P0C835	37	GRKGVKIGLFK
53	P0C835	42	KIGLFKDPETG
54	P80053	202	VATIAKEAANK
55	P80053	239	SEMGAKIVGVS
56	P80053	247	GVSDSKGGVIN

57	Q5VTE0	385	DRRSGKKLEDG
58	P00219	10	NYRTSKPVVGD
59	O43524	176	ESSPDKRLTLS
60	O43524	197	PYFKDKGDSNS
61	O43524	360	SPSVSKPCTVE
62	P16403	17	APPAEKAPVKK
63	P16403	22	KAPVKKKAAKK
64	P16403	23	APVKKKAAKKA
65	P16403	46	SELITKAVAAS
66	P16403	85	IKLGLKSLVSK
67	P16403	109	SFKLNKKAASG
68	P16403	121	AKPKVKKAGGT
69	P16403	137	VGAAKKPKKAA
70	P16403	148	GGATPKKSAKK
71	P16403	157	KKTPKKAKKPA
72	P10412	117	ASGEAKPKAKK
73	P16401	55	AVAASKERNGL
74	O75367	189	TVLSTKSLFLG
75	P40283	12	KKPAEKKPASE
76	P40283	34	APAEKKPKAGK
77	P40283	51	GAGGDKKKKMK
78	P40283	53	GGDKKKKMKKK
79	P40283	56	KKKKMKKKSVE
80	Q42681	64	ELVIRKLPFQR
81	Q41811	13	GKGLGKGARKR
82	O14979	180	VDCTIKTDPVT
83	O14979	204	AASVDKVLELK
84	O14979	227	RAKALKGKEPP
85	O14979	229	KALKGKEPPKK
86	O14979	306	GKCEIKVAQPK
87	O14979	311	KVAQPKEVYRQ
88	Q14103	114	TKKDLKDYFSK
89	Q14103	161	DQKEHKLNGKV
90	Q14103	165	HKLNGKVIDPK
91	Q14103	238	EEPVKKIMEKK
92	Q14103	260	KVAMSKEQYQQ
93	P16862	25	SVQSYKAAIDF
94	P16862	66	LRPDEKINKNV
95	P16862	150	NAVSTKPTPPP
96	P16862	168	SGLSSKVHSYT
97	P16862	438	HRSRGKRTTIV
98	O00159	146	ESGAGKTEATK
99	O00159	188	AFGNAKTLRND
100	O00159	209	VQFDFKGAPVG
101	O00159	535	DQRTRKSLGRG
102	O00159	594	SELSDKKRPET
103	P13538	147	LAYRGKKRQEA
104	P13538	148	AYRGKKRQEAP
105	P13538	186	ESGAGKTVNTK
106	P13538	296	QIMSNKKPELI
107	P13538	349	FSADEKTAIYK
108	Q96RI1	33	EMMSMKPAKGV

109	Q96RI1	120	EMPVTKKPRMG
110	Q96RI1	198	RLRKCKEMGML
111	Q96RI1	218	TEIQCKSKRLR
112	Q96RI1	256	KSCREKTELTP
113	Q96RI1	413	DRQYIKDREAV
114	Q96RI1	420	REAVEKLQEPL
115	P11940	177	RFKSRKEREAE
116	P11940	188	LGARAKEFTNV
117	P11940	208	DDERLKDLFGK
118	P11940	279	TELKRKFEQMK
119	P11940	284	KFEQMKQDRIT
120	P06400	240	LKEPYKTAVIP
121	P06400	420	ILKRVKDIGYI
122	Q86L05	160	VKFQLKKVLCL
123	Q86L05	196	VSLLKKGWQNI
124	P0CX43	24	YSNETKKRNFL
125	P0CX43	25	SNETKKRNFLE
126	P0CX43	101	NKKLIKKLSKK
127	P0CX43	106	KKLSKKYNAFI
128	P0CX43	147	DDLYGKVTDVR
129	P0CX43	195	FVSLLKKNWQN
130	P47914	33	RYESLKGVDPK
131	P47914	51	AKKHNKKGLKK
132	P47914	79	ALVKPKEVKPK
133	P47914	84	KEVKPKIPKGV
134	Q969Q0	17	CKKCGKHQPHK
135	Q969Q0	61	FRKKAKTTKKI
136	P0CX27	15	TYCKGKTCRKH
137	P0CX27	86	AQLTLKRCKHF
138	P0CX27	100	GEKKQKGQALQ
139	P02393	107	KEAVSKEEAEE
140	P0A7K2	71	KVAVIKAVRGA
141	P0A7K2	101	KEGVSKDDAEA
142	P07472	68	SAGEKKVNVIK
143	P07472	87	GLKEAKAAVDG
144	Q12962	161	SLAAQKFISDI
145	Q04207	195	NTAELKICRVN
146	Q04207	218	FLLCDKVQKED
147	Q04207	314	FKSIMKKSPFN
148	Q53688	256	IIIVEKILGFQ
149	B6A879	107	ILVEDKYTEET
150	B6A879	212	IGNKSKKVQEV
151	B6A879	213	GNKSKKVQEVW
152	B6A879	567	RTVKQKGEYAA
153	B6A879	573	GEYAAKNKLGG
154	Q9L5X8	8	TPQIDKLRNIA
155	Q9L5X8	543	TVNALKGKQLT
156	Q6NEZ3	41	DTQAPKPVNTV
157	Q6NEZ3	62	AWGLYKFNQKS
158	Q6NEZ3	226	ESASIKQDVQI
159	Q6NEZ3	332	ATSLLKPAKVM
160	Q9UKV3	44	WRKLPKMPEAV

161	Q9UKV3	238	QARAAKLSEGS
162	Q9UKV3	294	DDEGQKSREAP
163	P18281	47	LEEVAKPHELK
164	Q9SJU4	82	NATCGKRLASI
165	Q9SJU4	147	IVPGIKVDKGL
166	Q9SJU4	268	EGILLKPSMVT
167	Q9SJU4	353	KTWGGKEENVK
168	Q9ZU52	50	SDELVKTAKSI
169	P11118	87	TEEEIKEAFRV
170	P00889	76	GMRGMKGLVYE
171	P00889	193	GIHRTKYWELI
172	P00889	215	PCVAAKIYRNL
173	P00063	19	GEKIFKTKCAQ
174	P00063	30	CHTVDKGAGHK
175	P00063	87	YIPGTKMVFPG
176	P00077	16	PGDAAKGEKIF
177	P00043	12	KGSEKKGANLF
178	P00043	94	FGGLKKAKDRN
179	P00076	22	CHSAQKGVNST
180	P00076	52	YSNANKNAAIV
181	P00076	87	AGIKAKKDRQD
182	P00070	62	YSAGNKNKAVI
183	P00047	95	FGGLKKNKDRN
184	Q71V39	219	WKVERKEGNAS
185	P02993	44	IEKFEKEAQEM
186	P02993	51	AQEMGKGSFKY
187	P02994	214	YKGWEKETKAG
188	P02994	224	GVVKGKTLLEA
189	Q96KQ7	343	GRRKAKKKWRK
190	Q96KQ7	345	RKAKKKWRKDS
191	Q96KQ7	368	EPPRAKEPRGV
192	P53991	6	MASLRKPSNHA
193	P53991	234	NVPSYKFTGLF
194	P53991	285	NRKGGKMYIQD
195	P10587	17	FLFVDKNFVNN
196	P10587	145	MYKGKKRHEMP
197	P10587	202	VASSHKGKKDT
198	P11416	118	RRSIQKNMVYT
199	P11416	165	NDRNKKKKEAP
200	P11416	167	RNKKKKEAPKP
201	P27064	161	QVERDKLNKYG
202	P27064	305	VIDRQKNHGMH
203	P04717	227	AEAIYKSQAET
204	P04717	355	RDDYIKKDRSR
205	P04992	236	ETGEIKGHYLK
206	P04992	241	KGHYLKATAGT
207	P0A7J7	87	KAAGIKSGSGK
208	P0A7J7	95	SGKPNKDKVGK
209	P0CX53	130	LASVTKEILGT
210	P62986	88	RQLAQKYNCDK
211	P55072	109	PCPDVKYGKRI
212	P05547	13	EEAKKKQDDID

213	P05547	56	LLLRKKAAEEL
214	P05547	62	AAEELKKEQER
215	O95785	550	QLGRNKSTVHP
216	O95785	753	RFSFQKKKKKV
217	O95785	755	SFQKKKKKVAN
