# Supplementary material for: iMethyl-PseAAC: Identification of Protein Methylation Sites via a Pseudo Amino Acid Composition Approach
Source: Biomed Res Int. 2014 May 22;2014:947416. doi: 10.1155/2014/947416 (PMC4054830; doi:10.1155/2014/947416)
Supplement: Supplementary file 1 — S1: An analysis of the benchmark dataset used in Hu et al. (Biopolymers, 2011, 95, 763-771) S2: List of self-conflict samples in the benchmark dataset used in Hu et al. (Biopolymers, 2011, 95, 763-771) S3: Benchmark dataset used for studying the Arg-methylation. It contains 1,481 samples, of which 185 are positive and 1,296 negative. These data were extracted from UniProtKB/Swiss-Prot database (version UniProt release 2013_06). S4: Benchmark dataset used for studying the Lys-methylation. It contains 1,884 samples, of which 226 are positive and 1,518 negative. These data were extracted from UniProtKB/Swiss-Prot database (version UniProt release 2013_06). S5: Seven negative subsets for studying Arg-methylation. Each subset contains 185 negative samples randomly taken from the 1,296 negative samples in Online Supporting Information S3 except for the 6th subset, which contains 186 samples. None of the samples in one subset occurs in any other subset. S6: Seven negative subsets for studying Lys-methylation. Each subset contains 217 negative samples randomly taken from the 1,518 negative samples in Online Supporting Information S4 except for the 5th subset, which only contains 216 samples. None of the samples in one subset occurs in any other subset. S7: Independent dataset for studying the Arg-methylation. It contains 75 samples, of which 20 are positive and 55 negative. None of the samples listed here occurs in Online Supporting Information S3. S8: Independent dataset for studying the Lys-methylation. It contains 40 samples, of which 14 are positive and 26 negative. None of the samples listed here occurs in Online Supporting Information S4. S9: The code for encoding the peptides investigated in this paper. [file 947416.f1.zip › S7.pdf]

Online Supporting Information S7. Independent dataset for studying the Arg-methylation. It contains 75 samples, of which 20 are positive and 55 negative. None of the samples listed here occurs in Online Supporting Information S3.

Sample #	Protein ID	Site	Sequences
1	P62805	4	MMMSGRGKGGK
2	Q28009	211	QDRGGRGRGGG
3	Q28009	213	RGGRGRGGGGG
4	Q28009	229	GGYEPRGRGGG
5	Q28009	231	YEPRGRGGGRG
6	Q28009	235	GRGGGRGGRGG
7	Q28009	238	GGRGGRGGMGG
8	Q28009	246	MGGSDRGGFNK
9	Q28009	460	YGDDRRGGRGG
10	Q28009	463	DRRGGRGGYDR
11	Q28009	468	RGGYDRGGYRG
12	Q28009	472	DRGGYRGRGGD
13	Q28009	474	GGYRGRGGDRG
14	Q28009	478	GRGGDRGGFRG
15	Q28009	482	DRGGFRGGRGG
16	Q28009	370	GGGNGRGGRGR
17	Q28009	373	NGRGGRGRGGP
18	Q28009	375	RGGRGRGGPMG
19	Q28009	381	GGPMGRGGYGG
20	Q28009	394	SGGGGRGGFPS
21	Q28009	409	GGGQQRAGDWK
22	Q28009	428	MNFSWRNECNQ
23	P62805	18	KGGAKRHRKVL
24	P62805	20	GAKRHRKVLRD
25	P62805	24	HRKVLRDNIQG
26	P62805	36	TKPAIRRLARR
27	P62805	37	KPAIRRLARRG
28	P62805	40	IRRLARRGGVK
29	P62805	46	RGGVKRISGLI
30	P62805	56	IYEETRGVLKV
31	P62805	68	LENVIRDAVTY
32	P62805	79	TEHAKRKTVTA
33	P62805	93	VYALKRQGRTL
34	P62805	96	LKRQGRTLYGF
35	P10651	9	TKQTARKSTGG
36	P10651	27	ASKAARKAAPA
37	P10651	41	VKKPHRYRPPT
38	P10651	43	KPHRYRPPTVA
39	P10651	50	PTVALREIRRY
40	P10651	53	ALREIRRYQKS
41	P10651	54	LREIRRYQKST
42	P10651	64	TELLIRKLPFQ
43	P10651	70	KLPFQRLVREI
44	P10651	73	FQRLVREIAQD
45	P10651	84	FKTDLRFQSSA

46	P10651	117	AIHGKRVTIQP
47	P10651	129	DMQLARRLRGE
48	P10651	130	MQLARRLRGER
49	P10651	132	LARRLRGERSS
50	P10651	135	RLRGERSSSSS
51	P68429	9	TKQTARKSTGG
52	P68429	27	ATKAARKSAPA
53	P68429	41	VKKPHRFRPGT
54	P68429	43	KPHRFRPGTVA
55	P68429	50	GTVALREIRKY
56	P68429	53	ALREIRKYQKS
57	P68429	64	TELLIRKLPFQ
58	P68429	70	KLPFQRLVREI
59	P68429	73	FQRLVREIAQD
60	P68429	84	FKTDLRFQSSA
61	P68429	117	AIHAKRVTIMP
62	P68429	129	DIQLARRIRGE
63	P68429	130	IQLARRIRGER
64	P68429	132	LARRIRGERAA
65	P68429	135	RIRGERAAAAA
66	Q28009	501	GKMDSRGEHRQ
67	Q28009	505	SRGEHRQDRRE
68	Q28009	508	EHRQDRRERPY
69	Q28009	509	HRQDRRERPYY
70	Q28009	511	QDRRERPYYYY
71	Q28009	256	KFGGPRDQGSR
72	Q28009	261	RDQGSRHDSEQ
73	Q28009	315	NLYTDRETGKL
74	Q28009	358	VSFATRRADFN
75	Q28009	359	SFATRRADFNR
