# Supplementary material for: iMethyl-PseAAC: Identification of Protein Methylation Sites via a Pseudo Amino Acid Composition Approach
Source: Biomed Res Int. 2014 May 22;2014:947416. doi: 10.1155/2014/947416 (PMC4054830; doi:10.1155/2014/947416)
Supplement: Supplementary file 1 — S1: An analysis of the benchmark dataset used in Hu et al. (Biopolymers, 2011, 95, 763-771) S2: List of self-conflict samples in the benchmark dataset used in Hu et al. (Biopolymers, 2011, 95, 763-771) S3: Benchmark dataset used for studying the Arg-methylation. It contains 1,481 samples, of which 185 are positive and 1,296 negative. These data were extracted from UniProtKB/Swiss-Prot database (version UniProt release 2013_06). S4: Benchmark dataset used for studying the Lys-methylation. It contains 1,884 samples, of which 226 are positive and 1,518 negative. These data were extracted from UniProtKB/Swiss-Prot database (version UniProt release 2013_06). S5: Seven negative subsets for studying Arg-methylation. Each subset contains 185 negative samples randomly taken from the 1,296 negative samples in Online Supporting Information S3 except for the 6th subset, which contains 186 samples. None of the samples in one subset occurs in any other subset. S6: Seven negative subsets for studying Lys-methylation. Each subset contains 217 negative samples randomly taken from the 1,518 negative samples in Online Supporting Information S4 except for the 5th subset, which only contains 216 samples. None of the samples in one subset occurs in any other subset. S7: Independent dataset for studying the Arg-methylation. It contains 75 samples, of which 20 are positive and 55 negative. None of the samples listed here occurs in Online Supporting Information S3. S8: Independent dataset for studying the Lys-methylation. It contains 40 samples, of which 14 are positive and 26 negative. None of the samples listed here occurs in Online Supporting Information S4. S9: The code for encoding the peptides investigated in this paper. [file 947416.f1.zip › S8.pdf]

Online Supporting Information S8. Independent dataset for studying the Lys-methylation. It contains 40 samples, of which 14 are positive and 26 negative. None of the samples listed here occurs in Online Supporting Information S4.

Sample #	Protein ID	Site	Sequences
1	P62805	21	AKRHRKVLRDN
2	P10651	5	MMARTKQTARK
3	P10651	10	KQTARKSTGGK
4	P10651	15	KSTGGKAPRKQ
5	P10651	19	GKAPRKQLASK
6	P10651	24	KQLASKAARKA
7	P10651	28	SKAARKAAPAT
8	P10651	37	ATGGVKKPHRY
9	P10651	80	IAQDFKTDLRF
10	P68429	5	MMARTKQTARK
11	P68429	10	KQTARKSTGGK
12	P68429	15	KSTGGKAPRKQ
13	P68429	19	GKAPRKQLATK
14	P68429	28	TKAARKSAPAT
15	P62805	6	MSGRGKGGKGL
16	P62805	9	RGKGGKGLGKG
17	P62805	32	IQGITKPAIRR
18	P62805	45	RRGGVKRISGL
19	P62805	60	TRGVLKVFLEN
20	P62805	78	YTEHAKRKTVT
21	P62805	80	EHAKRKTVTAM
22	P62805	92	VVYALKRQGRT
23	P10651	57	IRRYQKSTELL
24	P10651	65	ELLIRKLPFQR
25	P10651	116	CAIHGKRVTIQ
26	P10651	123	VTIQPKDMQLA
27	P68429	24	KQLATKAARKS
28	P68429	37	ATGGVKKPHRF
29	P68429	38	TGGVKKPHRFR
30	P68429	65	ELLIRKLPFQR
31	P68429	80	IAQDFKTDLRF
32	P68429	116	CAIHAKRVTIM
33	P68429	123	VTIMPKDIQLA
34	Q28009	299	QIGIIKTNKKT
35	Q28009	303	IKTNKKTGQPM
36	Q28009	319	DRETGKLKGEA
37	Q28009	321	ETGKLKGEATV
38	Q28009	335	DPPSAKAAIDW
39	Q28009	352	SGNPIKVSFAT
40	Q28009	435	ECNQCKAPKPD
