# Supplementary material for: iMethyl-PseAAC: Identification of Protein Methylation Sites via a Pseudo Amino Acid Composition Approach
Source: Biomed Res Int. 2014 May 22;2014:947416. doi: 10.1155/2014/947416 (PMC4054830; doi:10.1155/2014/947416)
Supplement: Supplementary file 1 — S1: An analysis of the benchmark dataset used in Hu et al. (Biopolymers, 2011, 95, 763-771) S2: List of self-conflict samples in the benchmark dataset used in Hu et al. (Biopolymers, 2011, 95, 763-771) S3: Benchmark dataset used for studying the Arg-methylation. It contains 1,481 samples, of which 185 are positive and 1,296 negative. These data were extracted from UniProtKB/Swiss-Prot database (version UniProt release 2013_06). S4: Benchmark dataset used for studying the Lys-methylation. It contains 1,884 samples, of which 226 are positive and 1,518 negative. These data were extracted from UniProtKB/Swiss-Prot database (version UniProt release 2013_06). S5: Seven negative subsets for studying Arg-methylation. Each subset contains 185 negative samples randomly taken from the 1,296 negative samples in Online Supporting Information S3 except for the 6th subset, which contains 186 samples. None of the samples in one subset occurs in any other subset. S6: Seven negative subsets for studying Lys-methylation. Each subset contains 217 negative samples randomly taken from the 1,518 negative samples in Online Supporting Information S4 except for the 5th subset, which only contains 216 samples. None of the samples in one subset occurs in any other subset. S7: Independent dataset for studying the Arg-methylation. It contains 75 samples, of which 20 are positive and 55 negative. None of the samples listed here occurs in Online Supporting Information S3. S8: Independent dataset for studying the Lys-methylation. It contains 40 samples, of which 14 are positive and 26 negative. None of the samples listed here occurs in Online Supporting Information S4. S9: The code for encoding the peptides investigated in this paper. [file 947416.f1.zip › S9.pdf]

Online Supporting Information S9. The code for encoding the peptides investigated in this paper.

```
%A function for converting the sequence to discrete vector
for lysine
function [DataM]=Encode4PMeS_AAI_disorder_PSSM(Seqp)
wideth=11;
AAindex=[-0.591 -1.302 -0.733 1.570 -0.146;
 -1.343 0.465 -0.862 -1.020 -0.255;
 1.050 0.302 -3.656 -0.259 -3.242;
 1.357 -1.453 1.477 0.113 -0.837;
 -1.006 -0.590 1.891 -0.397 0.412;
 -0.384 1.652 1.330 1.045 2.064;
 0.336 -0.417 -1.673 -1.474 -0.078;
 -1.239 -0.547 2.131 0.393 0.816;
 1.831 -0.561 0.533 -0.277 1.648;
 -1.019 -0.987 -1.505 1.266 -0.912;
 -0.663 -1.524 2.219 -1.005 1.212;
 0.945 0.828 1.299 -0.169 0.933;
 0.189 2.081 -1.628 0.421 -1.392;
 0.931 -0.179 -3.005 -0.503 -1.853;
 1.538 -0.055 1.502 0.440 2.897;
 -0.228 1.399 -4.760 0.670 -2.647;
 -0.032 0.326 2.213 0.908 1.313;
 -1.337 -0.279 -0.544 1.242 -1.262;
 -0.595 0.009 0.672 -2.128 -0.184
 0.260 0.830 3.097 -0.838 1.512];
AAindexset=(AAindex-ones(20,1)*min(AAindex))./(ones(20,1)
*max(AAindex)-ones(20,1)*min(AAindex));
OSet='ACDEFGHIKLMNPQRSTVWY';
LastVMar=[];
    %%%%converting the seqence to disorder seqence
    [orderans,temLastVMar1]=getdisorder(Seqp,11);
    %%%Amino Acid Factors
    temLastVMar2=zeros(1,5*wideth);
    %%%%Obtaining the AAindex of AA in the seqence
    temLastVMar3=zeros(1,20*wideth);
    db='E:/blast/swissprot/swissprot.fasta';
    pssm=getPSSM(Seqp,db);
    newpssm=pssm';
    for i=1:length(temsequence)
        Pointsite=find(OSet==temsequence(i));
        temLastVMar2=AAindexset(Pointsite,:);
        temLastVMar3=1+exp(-newpssm(i,:));
```

```
    end
DataM=[temLastVMar1,temLastVMar2,temLastVMar3];
return


%inputfile: fasta 格式的文件
%使用灰色模型 GM(2,1)
function GreyPSSM=Grey_PSSM_2(inputFile)

[heads,seqs]=fastaread(inputFile);
pssm=blastpssm(inputFile,'E:/NCBI/blast-2.2.25+/db/swissp
rot');
if ~ischar(heads)
    len = length(heads);
    GreyPSSM = zeros(len,60);
    for i = 1 : len
        matrix = 1./(1+exp(pssm{i}(:,1:20)));
        v = AAVector(seqs{i});
        for j = 1 : 20
            p = GM21Param(matrix(:,j));
            GreyPSSM(i,-2+j*3:j*3)  =  [abs(p(1))  abs(p(2))
abs(p(3))]*v(j);
        end
    end
else
   GreyPSSM = zeros(1,60);
   matrix = 1./(1+exp(pssm(:,1:20)));
   v = AAVector(seqs);
   for j = 1 : 20
        p = GM21Param(matrix(:,j));
        GreyPSSM(-2+j*3:j*3)    =    [abs(p(1))    abs(p(2))
abs(p(3))]*v(j);
   end
end




function [orderans,orderscores]=getdisorder(seq,wideth)
%函数作用：获得蛋白质序列中各氨基酸的 disorder 的得分
seqfile='E:/XXuan/PTM/VSL2DIR/VSL2/tmpseqf.txt';
fid=fopen(seqfile,'w');
fprintf(fid,'%s',seq);
fclose(fid);
```

```
%cmd=sprintf('cd E:/XXuan/PTM/VSL2DIR/VSL2');
%system(cmd);
cmd=sprintf('java    -jar    vsl2.jar    -s:%s    -w:%d
>testseq.pred',seqfile,wideth);
[oderans,status]=system(cmd);
fid=fopen('E:/XXuan/PTM/VSL2DIR/VSL2/testseq.pred','r');
flag=0;
while flag==0
  line=fgetl(fid);
  if  strcmp(line,'NO.          RES.         PREDICTION
DISORDER')==1
  flag=1;
  line=fgetl(fid);
  end
end
orderans=[];
orderscores=[];
while ~feof(fid)
    flag2=1;
  line=fgetl(fid);
  row=sscanf(line,'%d %s %f \n',3);
  if
strcmp(line,'=======================================')==
1
      flag2=0;
  end
  if length(row)==3&flag2>0
      numth=size(orderans,2)+1;
      orderans(numth).No=row(1);
      orderans(numth).AA=char(row(2));
      orderans(numth).Score=row(3);
      orderscores=[orderscores;row(3)];
  end
end
fclose(fid);
```

```
function [pssm]=getPSSM(seq,swiss)
%函数作用：获得蛋白质序列 seq，相对 swiss 数据库进行 psi-blast 搜索，
生成的 PSSM 矩阵
%seq：蛋白质序列,
%swiss：SwissProt 数据库所在位置
```

%pssm:返回的20*L矩阵,其中L表示序列长度,20对应20种氨基酸:A R N D C Q E G H I L K M F P S T W Y V
%注意：要在本机调用本函数，本机必须安装有NCBI Blast程序包以及对应的swiss数据库文件

```
seqFile='c:/tmpseq.txt';
fid=fopen(seqFile,'w');
    fprintf(fid,'>%s\r\n%s','MyProtein',seq);
fclose(fid);



cmd=sprintf('blastpgp    -j    3    -i    %s    -d    %s    -Q
C:/pssm.txt',seqFile,swiss);
[result,status]=system(cmd);
fid=fopen('C:/pssm.txt','r');
for i=1:3
  line=fgetl(fid);
end
pssm=zeros(0,20);
while ~feof(fid)
  line=fgetl(fid);
  row=sscanf(line,'%d %s %d %d %d %d %d
```
